# Confidence-Guided Local Structure Prediction with HHfrag

**DOI:** 10.1371/journal.pone.0076512

**Published:** 2013-10-16

**Authors:** Ivan Kalev, Michael Habeck

**Affiliations:** 1 Department of Protein Evolution, Max Planck Institute for Developmental Biology, Tübingen, Germany; 2 Institute for Mathematical Stochastics, Georg-August-University of Göttingen, Göttingen, Germany; University of Michigan, United States of America

## Abstract

We present a method to assess the reliability of local structure prediction from sequence. We introduce a greedy algorithm for filtering and enrichment of dynamic fragment libraries, compiled with remote-homology detection methods such as HHfrag. After filtering false hits at each target position, we reduce the fragment library to a minimal set of representative fragments, which are guaranteed to have correct local structure in regions of detectable conservation. We demonstrate that the location of conserved motifs in a protein sequence can be predicted by examining the recurrence and structural homogeneity of detected fragments. The resulting confidence score correlates with the local RMSD of the representative fragments and allows us to predict torsion angles from sequence with better accuracy compared to existing machine learning methods.

## Introduction

Deciphering the protein folding problem remains a fundamental challenge. Although experimental and theoretical studies have improved our understanding of the process, there are still many open problems, one of which is to reliably predict the native structure from sequence only. It has become clear that protein sequences do not adopt unlimited varieties of global and local shapes. Proteins that fold do not explore the complete conformational space [1]. Rather, the local structure of each polypeptide segment is biased by the geometrical and chemical properties of its constituent amino acids [2]. This observation prompted the development of structural alphabets in an attempt to partition known protein structures into a dictionary of discrete motif prototypes [3]. It has been reported that such fragment libraries may be sufficient to describe all protein folds in terms of recurrent building blocks [4], [5].

One of the first efforts to systematically study the amino acid preferences of known structural motifs is the I-Sites fragment library [6], [7], which was later adapted for use in *ab initio* fragment assembly [8] with Rosetta [9]. This has proven to be a successful strategy for local structure prediction from sequence. However, not all motifs have identifiable sequence preferences. A fixed set of sequence-based prototypes is generally insufficient to detect all structural elements in existing protein structures [10]. Although the sensitivity of sequence-based fragment detection can be pushed to higher levels by dynamic fragment selection [9], our studies show that protein structures are not simple combinations of conserved sequence motifs. Rather, we observe an alternating pattern of easily detectable elements (often matching to known I-Sites), connected by highly variable regions with no detectable sequence conservation (typically flexible coils and linkers) [10]. The ability to discriminate between these high- and low-precision regions is important, since local structure prediction in non-conserved regions is unreliable and should not be trusted. Conversely, protocols that assemble fragments should be able to obtain information about the locations of the conserved motif instances in a given protein sequence and utilize the corresponding torsion angle predictions with higher precedence.

In this study, we introduce a reliable algorithm for blind prediction of local high- and low-precision regions in protein sequences. By analysing the structural consistency and recurrence of motifs in dynamic HHfrag libraries, this method quantifies the quality of fragment assignment at each query position and nominates representative fragments, which are most likely to match the local structure of the query closely. We show that the confidence score of local structure prediction correlates well with the local RMSD and torsion angle error of representative fragments and can be used as a reliable predictor for the presence of high- or low-precision regions. Finally, we illustrate how this property can be used to predict torsion angles from sequence with higher accuracy than existing machine learning methods [11], [12].

## Methods

To predict the torsion angles of a target protein of unknown structure, we first build a dynamic fragment library using the HHfrag method for fragment detection from sequence [10]. The reliability of fragment detection at each target position is then analyzed by clustering and filtering all fragments, covering a given target residue. If the obtained confidence score for this position is indicative of a local region of high precision, our algorithm proceeds by selecting a representative fragment (the centroid) and extracts the torsion angles of the centroid. The final list of predicted torsion angles for the entire target sequence is compiled from the set of all nominated centroids.

### Dynamic Fragment Selection

For a given target sequence, we compute a dynamic library of variable-length fragments using the standard HHfrag protocol [10]. HHfrag is a sensitive and accurate fragment detection method, which uses internally the HHsearch [13] algorithm for local alignment of pairs of profile hidden Markov models (HMM).

For this purpose, both the query sequence and all template proteins are represented as profile HMMs with secondary structure information incorporated into the HHM files [14]. Sequence profiles are generated with PSI-BLAST [15]. Secondary structure information is calculated with DSSP [16] from the experimental structures of all templates or predicted with PSIPRED [17] for the query. The database of template structures (PDBS25) is a non-redundant subset of PDB [18], derived from the April 2010 build of PDBselect25 [19] (4824 protein chains, filtered at 25% sequence identity).

All detected fragments are directly excised by HHfrag from their corresponding experimental structures. The resulting fragment library is a position-specific, ordered set of structural motifs, ranging from 6 to 21 residues in length [10]. Each fragment is described by its matching query/subject positions, (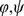) torsion angle pairs and backbone coordinate trace.

### Fragment Clusters

To eliminate outliers in the raw dynamic fragment libraries and select representative fragments for each target position, we propose a greedy *outlier rejection* algorithm.

For every position 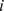 in the target sequence, we build a cluster of all fragments, covering this position. Each *fragment cluster* is represented by a graph whose nodes are fragments connected by weighted edges. The edge weights are the 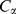-RMSDs between each given pair of fragments. Since all HHfrag libraries are composed of motifs of varying length and start positions, some fragments in a given cluster may overlap by less than 6 residues. In such cases, the RMSD cannot be a meaningful indicator for the structural divergence between fragments. Therefore, we do not connect these pairs with an edge.

Each fragment cluster has two key properties:

1. *Recurrence* (r) – refers to the sequence conservation of a structural motif. The recurrence of a given motif is measured by counting the number of its instances in the non-redundant database of profiles (PDBS25). We compute the recurrence of a query position and its associated cluster by simply counting the number of assigned fragments, covering this position, which is the number of vertices in the cluster.
2. *Consistency* (c) – characterizes the structural homogeneity of the fragments. We measure the consistency of a cluster by calculating the subset of structurally similar pairs of fragments. Two vertices are considered similar if the weight of their connecting edge, measured by the *C_α_*-RMSD between the two fragments, does not exceed a critical threshold of 1.5 Å.

### Filtering

**Figure 1:**
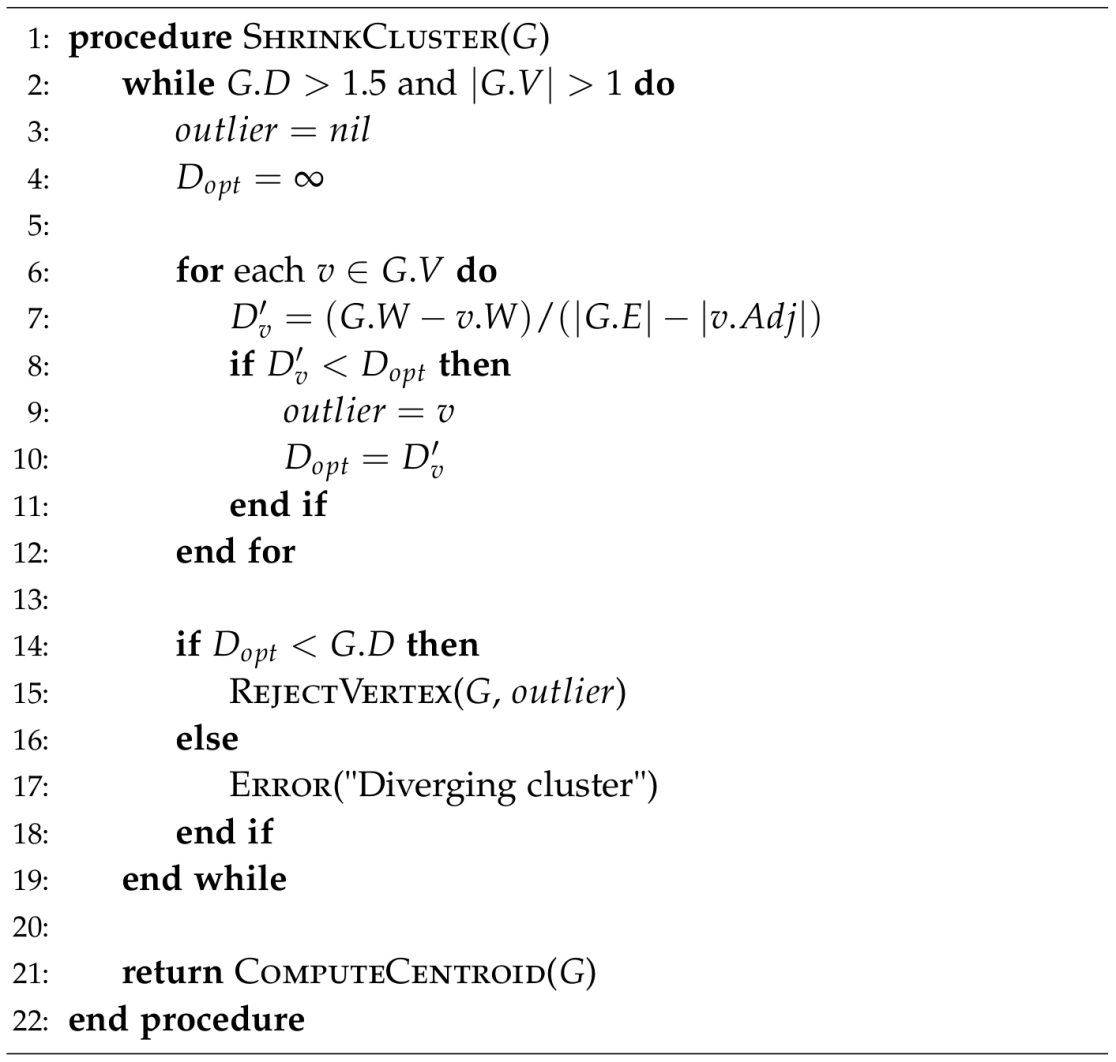
The outlier rejection algorithm. The *ShrinkCluster* procedure operates on an undirected graph 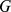. The algorithm keeps track of the total sum of all edge weights in the graph (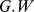) and their average (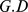). 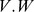 denotes the total weight of all edges incident to a given vertex 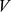.

The goal of the outlier rejection algorithm is to improve the structural consistency of a given cluster by performing a minimum number of node deletions (Figure 1).

Every cluster (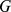) keeps track of the total sum of all of its pairwise RMSDs (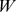). Each cluster vertex (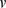) also maintains an up-to-date sum of the weights of all edges incident to it 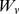. A fragment cluster is said to be *stable*, when the average RMSD 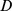 between all adjacent vertices is lower than the threshold of 1.5 Å:
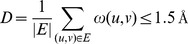
(1)where 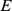 is the set of all edges and 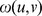 is the RMSD between a pair of fragments 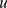 and 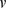. The algorithm performs iterative rejections, until the cluster stability criterion is satisfied. On each iteration, we probe all nodes by calculating the resulting average RMSD 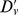 if vertex 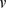 is excluded. This is given by the following greedy criterion:
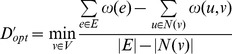
(2)where 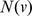 is the adjacency set of vertex 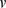. The fragment, whose exclusion from the cluster would produce the most significant drop in 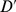 towards stability (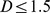
Å), is selected for deletion and removed.

**Figure 2:**
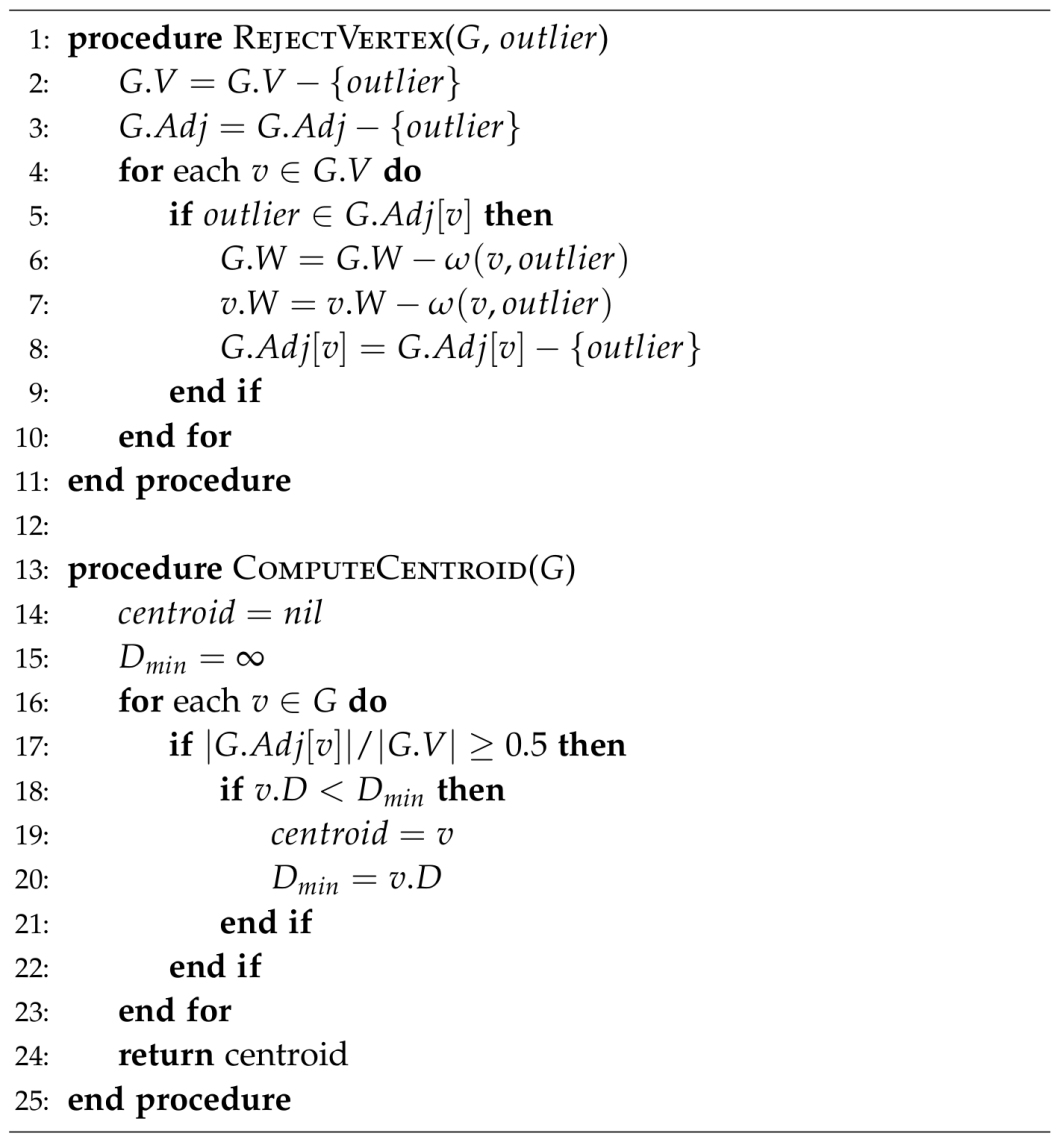
Supporting procedures.

When the graph is implemented using an adjacency sets data structure, each removal requires linear time of 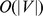 (Figure 2), needed to update all adjacency sets (linear complexity) and recalculate the cached sums of weights 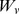 of affected nodes (constant time per fragment). If no fragment removal results in decrease of the average RMSD 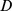, this cluster is not able to shrink further. Such clusters are considered *diverging*, which indicates heterogeneous aggregates of false positive fragments. In such cases we terminate the filtering procedure and the corresponding target position remains unassigned, additionally marked to be part of a low-precision region. The same result is also obtained if all cluster nodes are rejected before stability has been reached.

The maximum number of iterations equals the number of fragments 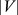 in a cluster. The amount of work performed on each iteration 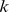 is equal to the current number of nodes 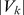 on iteration 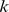 (to identify the candidate for rejection) plus additional 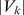 (to remove the candidate and update all adjacency sets and cached weight sums). The worst-case running time of the filtering algorithm is thus given by:
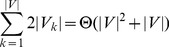
(3)


However, most clusters reach stability much earlier than 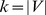 number of iterations, so the typical running time is in practice much better.

### Representative Fragments and Confidence

We define the *representative* fragment of a *stable cluster* to be its centroid, which is the node with the minimum average RMSD to its adjacent vertices (Figure 2). Since the number of edges per node may vary, we consider only fragments connected to a significant number of cluster elements (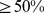).

The *confidence* score 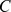, assigned to a given target position 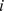 and its representative fragment, we derive from the recurrence (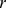) and structural consistency values (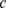) of the corresponding filtered (stable) cluster:
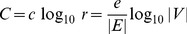
(4)where 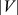 is the number of fragments in the filtered cluster, 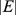 is its total number of edges and 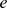 is the number of pairwise RMSDs not greater than 1.5 Å.

### Prediction of Torsion Angles

We use the filtered fragment libraries and their associated representative fragments for direct prediction of torsion angles from sequence – a strategy, which bears resemblance to earlier approaches [20]. For each position 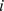 in a given target protein, we build a fragment cluster and compute the centroid fragment, as outlined above. The pair of torsion angle values (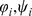) of the representative fragment at target position 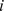 is extracted from the centroid’s experimental structure and directly reported as the final prediction at that position. A confidence value of 0.8 or higher indicates a reliable prediction within a local region of high precision.

### Performance Evaluation

We used a set of 106 protein targets from the CASP9 competition [21] to benchmark the accuracy of torsion angle prediction. The PDBS25 database of template structures, used for fragment extraction by HHfrag, contains only older entries and no homologous chains. For each target, we obtain a prediction for its torsion angles with the procedure, described above. The prediction accuracy was measured by the mean absolute error (MAE) between the predicted (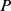) and experimental (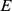) torsion angle values:
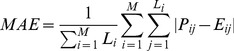
(5)where 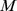 is the number of proteins and 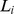 is the number of residues in protein 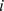 of confidence greater than a chosen cutoff (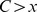). All predicted and experimental torsion angles are computed in degrees within the 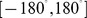 range. To keep the error values in that range as well, we apply the following rule when calculating the absolute angular errors 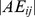:



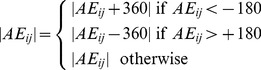
(6)Predicted torsion angles for the same set of proteins were also obtained with ANGLOR [11] and TANGLE [12] using their web server interfaces. MAE values were calculated using an identical procedure.

The quality of the fragment library is also evaluated in terms of local *precision* and *coverage* (see [10]). The *accuracy* of an assigned fragment is assessed by the local 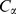-RMSD of the fragment to the native structure. If this RMSD is below 1.5 Å, the fragment is considered a positive (compatible) hit. The percentage of correctly assigned fragments that cover the same residue is the local precision of the fragment library. The coverage is the percentage of residues that are covered by at least one compatible fragment.

## Results and Discussion

In an earlier study, we discussed the precision of popular methods for local structure prediction by sequence-based remote homology detection [10]. We have shown that the precision of fragment detection with this strategy is relatively low (40% on average for Rosetta NNmake [9], [22] and 70% for HHfrag). Additionally, the precision of dynamic fragment libraries is never uniform along the target sequences. Local zones of high precision emerge in regions, containing detectable, well-known structural motifs [10]. These motifs were found to exhibit a certain degree of sequence profile conservation and can be observed in a wide range of evolutionary unrelated proteins, thus serving the purpose of structural design patterns [4], [6]. The quality of fragment libraries however rapidly decreases as we move away from the high-precision regions and enter areas of very high variability, such as loops and linkers.

In this study, we propose an intuitive model for the prediction of local high- and low-precision zones and demonstrate how this method can be applied to increase the reliability of local structure prediction.

### The Confidence Score

**Figure 3:**
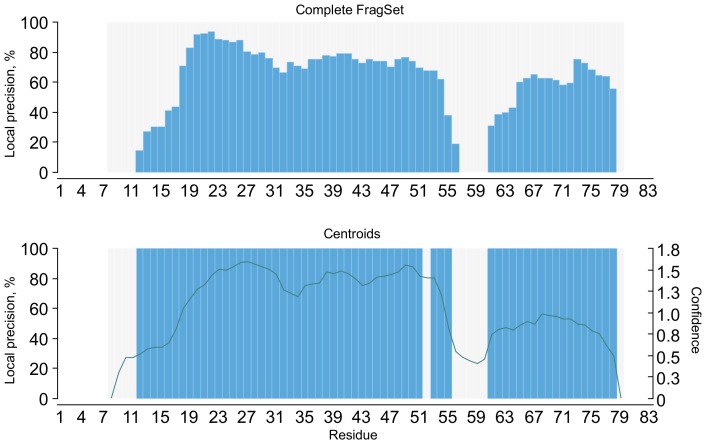
Local precision of filtered fragment libraries. The local precision of the complete HHfrag library for benchmark target 3Å 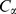-RMSD to the native structure for each target residue (blue bars; see [10]). The library was then filtered using the outlier rejection algorithm, described in Methods. The precision of the resulting library of representative fragments was measured in a similar way, except that only one fragment per target position (its associated centroid) was taken into account. The green curve shows the corresponding confidence values for each target residue.

A key property of locally conserved motifs is that they usually have good local sequence-structure correlation [6]. Such query sequence regions generate lists of matching fragment instances of higher *structural consistency* (Figure 3). Additionally, the most conserved motifs, such as the *GD box* [23], can be highly ubiquitous and often contain hundreds of detectable instances in a non-redundant structural database. The *recurrence* of a given motif was determined to be an equally strong indicator for reliable local structure prediction and this observation already plays an integral role in the HHfrag fragment detection method [14].

The *confidence* score of fragment assignment (Equation 4) integrates these two properties. The recurrence term in the expression is a weighting factor for the structural consistency of all instances of a given motif. Highly conserved motifs have a recurrence of 50–100 or more, which increases confidence because the structural consistency amplified by a factor greater than one. Clusters of size greater than 10 are up-weighted because 10 is the critical number of HHsearch hits, below which the program switches to a less strict, greedy hit-ranking mode [10], [13]. At 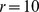, the logarithm of the recurrence is 1 and the confidence is determined entirely by the degree of structural consistency. Clusters of size less than 10 are associated with increasing uncertainty and thus severely penalized. We can follow the same intuition to define natural thresholds for the confidence score:

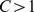
: credible local structure prediction, which is guaranteed to be accurate. For example, a confidence value of 1.5 can be obtained for a highly homogeneous cluster (75%) of large size (100 fragment instances).

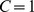
: transitional zone. Confidence value of 1 corresponds to a rare motif (10 instances) with maximum structural conservation or a highly abundant motif (100 instances) at moderate consistency of 50%.

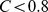
: uncertainty. This confidence threshold is equivalent to a small fragment cluster (10 instances) at consistency equal to the average precision of HHfrag for ordinary I-Sites (80%) or a highly recurrent motif (100 instances) at low precision (40%).

### Filtered Fragment Libraries

To maximise the precision of fragment-based local structure prediction in conserved regions, we propose a filtering algorithm used to compile reduced fragment libraries of low complexity and very high local precision. For each query position, we build a fragment cluster, as outlined in Methods. Inconsistent fragments in each cluster are iteratively rejected until a sufficient level of structural consistency is reached. A single, representative fragment is then selected out of the pool of surviving cluster members. After removing the outliers in all clusters, the entire library is enriched with high-quality fragments. In regions of local conservation, this always results in local centroid precision of 100%, i.e. representative fragments in those regions are guaranteed to have a low RMSD to the native structure (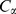-RMSD 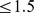 Å).

This is illustrated in Figure 3. After filtering the raw HHfrag library for target 3 nzl, we obtain a list of position-specific representative fragments (one fragment per query position). The local precision of the resulting filtered library of centroids is 100% for all high-accuracy regions, observed on the original plot (see Figure 3 for details). The confidence curve correlates well with the observed local precision pattern, dropping rapidly in regions where inaccurate centroids have been selected. Similar results were obtained after filtering all remaining CASP9 targets from the standard HHfrag benchmark [10] (see supplementary material).

### Confidence-guided Local Structure Prediction

To study the reliability of the confidence score as an indicator for local motif conservation, we measured the local precision of calculated centroids for all targets in the standard HHfrag benchmark [10].

**Figure 4:**
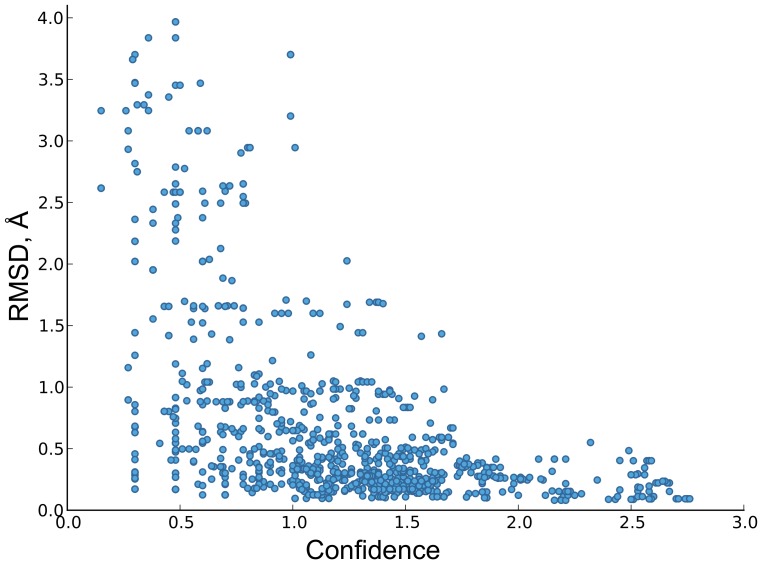
Reliability of the confidence score. Shown is the correlation between the confidence scores of all residue-wise centroids in our CASP9 benchmark and their 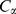-RMSD similarity to the corresponding native structures. The RMSD values were calculated over the entire lengths of the representative fragments.

Figure 4 shows the overall correlation between local accuracy of cluster centroids and confidence in our benchmark. A weak confidence value (0.1–0.6) is a clear signal for the presence of a low-accuracy region. Higher confidence values (0.8–1.0) indicate generally conserved motifs, which sometimes cannot be predicted reliably. The overall centroid precision in this confidence interval is 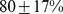 with an average RMSD to native structures of 1.0
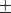
0.9 Å. Confidence greater than 1.0 guarantees an accurate and reliable local structure prediction with a very low chance of an error. The overall precision in such regions reaches 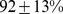 with an average RMSD to native structures as low as 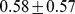 Å. These results confirm the expected confidence thresholds derived in the previous section.

Based on these observations, we propose a context-aware method for prediction of torsion angles from sequence (see Methods). In query regions of high confidence (0.8 or better), we rely on the corresponding centroids as a source of highly accurate torsion angle predictions. This allows client methods to consume predicted torsion angles in order of decreasing confidence. The confidence score brings contextual perspective to the local structure prediction, allowing fragment assembly applications to decide at runtime whether a given prediction should be trusted or rather replaced by exhaustive sampling of a generic structural alphabet.

### Benchmark

We examined the performance of our centroid-based torsion angle predictor on 106 protein targets from the CASP9 competition [21]. The mean absolute error (MAE) of predicted 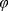 and 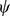 angles was compared against the values, obtained with two popular methods for torsion angle prediction from sequence: ANGLOR [11] and TANGLE [12]. The overall precision of HHFrag in comparison with these methods is summarized in Table 1.

**Table 1:** Torsion angle prediction performance.

Method	Confidence	MAE (*φ*)	MAE (*ψ*)
TANGLE	0.8	31.9±34.9°	90.7±30.6°
ANGLOR	0.8	18.7±25.8°	86.4±43.0°
HHfrag	0.8	18.6±27.0°	22.5±36.2°
TANGLE	0.0	34.2±36.4°	87.4±32.3°
ANGLOR	0.0	23.5±30.0°	84.7±47.6°
HHfrag	0.0	25.4±34.7°	34.9±48.9°

Mean absolute error (MAE) of 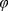 and 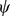 torsion angle prediction for high-confidence (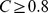) and all residues (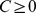) in our benchmark.

**Figure 5:**
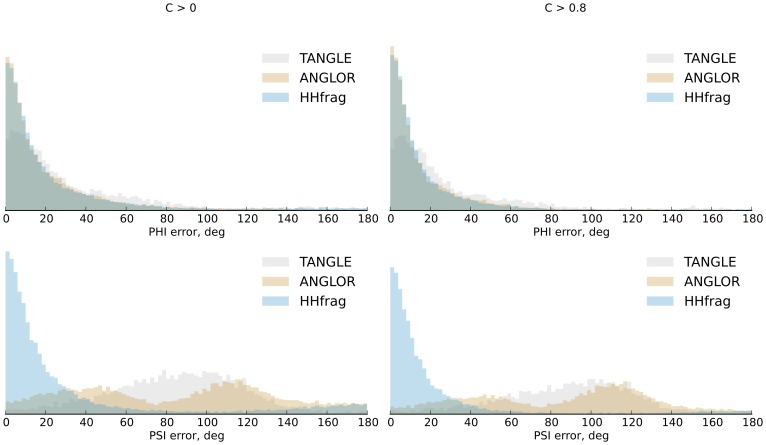
Distributions of the absolute errors of predicted torsion angles. Shown are the distributions of 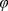 and 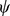 prediction errors for high-confidence (right) and all target residues (left) in our benchmark.

When regions of any confidence are considered, our method predicts 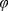 angles with slightly lower accuracy than ANGLOR (2° higher MAE), but better than TANGLE. For 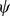 angles however, HHfrag is significantly more accurate, improving on both ANGLOR and TANGLE by a 50° lower MAE (Figure 5). The observed MAE of HHFrag is 25.4° for 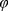 and 34.9° for 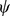 angles on average.

**Figure 6:**
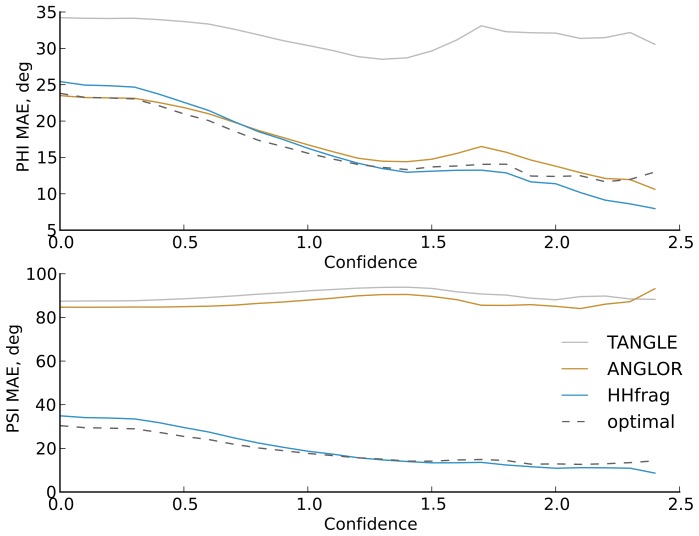
Torsion angle prediction accuracy at increasing confidence cutoffs. We measured the mean absolute error (MAE) of 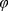 and 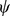 angle prediction at increasing confidence cutoffs. For each cutoff, we computed the 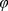 and 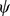 MAE for all target residues in our benchmark, having a confidence greater or equal to the cutoff. The optimal curve represents the lowest possible MAE, which our filtering algorithm could achieve by always picking a centroid identical to the best-fitting fragment at each position (the fragment with the lowest 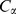-RMSD).

As expected, the quality of torsion angle prediction with HHfrag improves further when the confidence score of each query position is taken into account (Figure 6. In target regions of 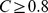, the average MAE drops by 6.8° and 12.4° for 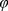 and 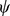 angles respectively. Generally, the MAE of HHfrag predictions gradually decreases as we discard regions of lower confidence (Figure 6). Such tendency is less pronounced for 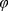 angle predictions with ANGLOR or TANGLE and completely lacking when these methods are used to predict 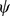 angles (Figure 6). HHfrag does not always select optimal centroids in low-confidence regions (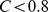) as the lack of sufficient recurrence and consistency of such clusters hinders the filtering algorithm. However, in transitional zones (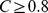), the deviation from the optimal MAE becomes negligible and for high-confidence regions (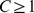) our method is guaranteed to extract torsion angles from the best-fitting fragment at each position. These results highlight the importance of taking the local conservation into account and confirm the utility of our confidence-guided prediction strategy.

### Availability

The fragment filtering algorithm and the confidence-guided torsion angle predictor are implemented as an HHfrag extension in version 1.2 of the CSB open-source SDK [24]. An HHfrag web server is available at http://toolkit.tuebingen.mpg.de/hhfrag. The standalone executable, source code and Python API are freely available for download at http://csb.codeplex.com/releases.

## Conclusion

We discussed the correlation between the quality of local structure prediction from sequence and the degree of local motif conservation. We introduced a greedy algorithm for fragment filtering, which can be used to decrease the complexity of dynamic, sequence-based fragment libraries. This algorithm takes a central part of our confidence-guided framework for prediction of local conservation, which captures the structural homogeneity and recurrence of detected fragments. Protein sequence regions, containing instances of ubiquitous and structurally consistent motifs, generally correspond to zones of very high local accuracy. We showed that this information can be used for reliable prediction of torsion angles from sequence with better accuracy compared to existing machine learning methods.

## Supporting Information

Benchmark S1 **Local centroid precision for each target in the benchmark set and a breakdown of the torsion angle prediction performance by residue type and secondary structure.**
(ZIP)
