# Supplementary material for: Confidence-Guided Local Structure Prediction with HHfrag
Source: PLoS One. 2013 Oct 16;8(10):e76512. doi: 10.1371/journal.pone.0076512 (PMC3797814; doi:10.1371/journal.pone.0076512)
Supplement: Benchmark S1 — Local centroid precision for each target in the benchmark set and a breakdown of the torsion angle prediction performance by residue type and secondary structure. (ZIP) [file pone.0076512.s001.zip › Benchmark_S1.html]

xml version="1.0" encoding="utf-8"?


Supplementary Data: TOC


1. Local precision of centroid fragment libraries

   Here we provide a comprehensive list of all local precision diagrams
   for filtered fragment libraries in our benchmark (CASP9 targets). The local
   precision was calculated as described in the manuscript: 100% if the corresponding
   centroid at position i has compatible structure (RMSD≤1.5Å); 0% otherwise.
   The confidence values for every target position/cluster are also shown.

   | Target | CASP 9 Category | Length |
   | --- | --- | --- |
   | 2l09A | TBM | 62 |
   | 2kjxA | FM | 65 |
   | 3nmdA | TBM | 72 |
   | 2l01A | TBM | 77 |
   | 3nrlA | FM | 81 |
   | 2l02A | TBM | 82 |
   | 3obhA | TBM | 82 |
   | 3nzlA | TBM | 83 |
   | 2kywA | TBM | 87 |
   | 3nppA | TBM | 87 |
   | 2l0bA | TBM | 91 |
   | 2l0cA | TBM | 97 |
   | 2kxyA | TBM | 100 |
   | 3nrtA | FM/TBM | 103 |
   | 3nrfA | TBM | 106 |
   | 3nbmA | TBM | 108 |
   | 3o0lA | TBM | 112 |
   | 2l0dA | TBM | 114 |
   | 3nnqA | TBM | 114 |
   | 3nrwA | TBM | 117 |
   | 3npdA | FM | 118 |
   | 3mqoA | TBM | 120 |
   | 3nkzA | TBM | 123 |
   | 3neuA | TBM | 125 |
   | 2kytA | TBM | 125 |
   | 3njaA | TBM | 125 |
   | 3nymA | FM | 128 |
   | 2l3bA | TBM | 130 |
   | 2kzxA | TBM | 131 |
   | 2ky9A | TBM | 132 |
   | 3nrdA | TBM | 135 |
   | 3n6yA | TBM | 137 |
   | 3nohA | TBM | 139 |
   | 3n53A | TBM | 140 |
   | 3nklA | TBM | 141 |
   | 3mr0A | TBM | 142 |
   | 3nhvA | TBM | 144 |
   | 3n70A | TBM | 145 |
   | 2kzwA | TBM | 145 |
   | 3nrvA | TBM | 148 |
   | 2ky4A | FM | 149 |
   | 3mxqA | TBM | 152 |
   | 2kyyA | TBM | 153 |
   | 2l06A | FM | 155 |
   | 3ni8A | TBM | 158 |
   | 3njcA | TBM | 158 |
   | 3n72A | TBM | 164 |
   | 3natA | FM | 164 |
   | 2l3fA | TBM | 166 |
   | 3na2A | TBM | 172 |
   | 3nkgA | FM | 174 |
   | 3nwzA | TBM | 176 |
   | 3nqwA | TBM | 179 |
   | 3mseB | TBM | 180 |
   | 3nrhA | FM | 182 |
   | 3ot2A | TBM | 187 |
   | 3mr7A | TBM | 189 |
   | 3n1uA | TBM | 191 |
   | 3ngwA | TBM | 208 |
   | 3ni7A | TBM | 213 |
   | 3mqzA | TBM | 215 |
   | 3nrgA | TBM | 217 |
   | 3o14A | TBM | 223 |
   | 3nnrA | TBM | 228 |
   | 3oruA | TBM | 234 |
   | 3ne8A | TBM | 234 |
   | 3no3A | TBM | 238 |
   | 2xgfA | TBM | 242 |
   | 3nkhA | TBM | 244 |
   | 3no6A | TBM | 248 |
   | 3nywA | TBM | 250 |
   | 3nyyA | TBM | 252 |
   | 3nmbA | TBM | 260 |
   | 3oqlA | TBM | 262 |
   | 3nxhA | TBM | 269 |
   | 3no2A | TBM | 276 |
   | 3on7A | TBM | 280 |
   | 3obiA | TBM | 288 |
   | 3nreA | TBM | 291 |
   | 3nuwA | TBM | 295 |
   | 3nyiA | TBM | 297 |
   | 3o1lA | TBM | 302 |
   | 3nkdA | TBM | 305 |
   | 3npfA | TBM | 306 |
   | 3ooxA | TBM | 312 |
   | 3mwbA | TBM | 313 |
   | 3nr8A | TBM | 316 |
   | 3nqkA | FM/TBM | 319 |
   | 3n91A | FM/TBM | 323 |
   | 3mwxA | TBM | 326 |
   | 3p1tA | TBM | 337 |
   | 3os7A | TBM | 341 |
   | 3nf2A | TBM | 352 |
   | 3n8uA | FM | 361 |
   | 3n6zA | FM | 363 |
   | 3mt1A | TBM | 365 |
   | 3n0xA | TBM | 374 |
   | 3nfvA | TBM | 382 |
   | 3os6A | TBM | 399 |
   | 3nraA | TBM | 407 |
   | 3nieA | TBM | 429 |
   | 3netA | TBM | 465 |
   | 3pfeA | TBM | 472 |
   | 3mx3A | TBM | 490 |
   | 3nlcA | TBM | 549 |
   | 3mwtA | TBM | 577 |
   | 3n05A | TBM | 590 |
   | 3nzpA | TBM | 619 |
   |  | | |
2. Torsion angle prediction accuracy (MAE)

   Here we provide a more detailed breakdown of the torsion angle prediction
   accuracy (MAE) by secondary structure and residue type. The MAE values shown
   in the table were calculated for high-confidence regions (C>0.8). For detailed
   MAE-to-confidence correlation reports please follow the links in the first column.

   | Category | HHfrag, φ | HHfrag, ψ | ANGLOR, φ | ANGLOR, ψ | TANGLE, φ | TANGLE, ψ |
   | --- | --- | --- | --- | --- | --- | --- |
   | Coil | 33.0 ± 39.9° | 40.7 ± 49.3° | 33.5 ± 35.5° | 78.2 ± 53.0° | 46.6 ± 50.2° | 74.5 ± 35.5° |
   | Helix | 8.8 ± 13.7° | 11.8 ± 22.6° | 7.7 ± 11.9° | 112.8 ± 19.8° | 14.0 ± 11.4° | 101.2 ± 23.2° |
   | Strand | 22.2 ± 21.7° | 24.2 ± 33.9° | 24.4 ± 24.0° | 46.4 ± 25.0° | 50.2 ± 28.2° | 87.2 ± 29.5° |
   | Buried | 18.6 ± 27.0° | 22.5 ± 36.2° | 18.7 ± 25.8° | 86.4 ± 43.0° | 31.9 ± 34.9° | 90.7 ± 30.6° |
   | Exposed | 20.0 ± 31.3° | 24.6 ± 39.3° | 19.1 ± 27.7° | 95.9 ± 42.7° | 31.9 ± 40.1° | 87.2 ± 32.5° |
   | ALA | 14.3 ± 22.2° | 17.9 ± 31.8° | 13.8 ± 20.6° | 92.3 ± 39.7° | 21.9 ± 23.4° | 96.0 ± 27.3° |
   | CYS | 24.0 ± 33.0° | 25.7 ± 37.9° | 24.9 ± 31.8° | 81.0 ± 45.4° | 39.7 ± 33.3° | 90.1 ± 36.4° |
   | ASP | 19.7 ± 28.8° | 24.2 ± 36.6° | 20.8 ± 29.6° | 93.3 ± 42.9° | 27.7 ± 30.3° | 84.3 ± 36.0° |
   | GLU | 14.6 ± 21.6° | 19.6 ± 33.3° | 13.9 ± 19.3° | 95.1 ± 40.2° | 23.3 ± 23.7° | 93.8 ± 30.6° |
   | PHE | 19.9 ± 24.4° | 22.3 ± 31.9° | 18.6 ± 22.6° | 81.0 ± 45.6° | 33.4 ± 29.5° | 94.2 ± 30.2° |
   | GLY | 40.7 ± 51.9° | 35.0 ± 46.3° | 55.0 ± 50.0° | 88.3 ± 46.0° | 96.7 ± 66.3° | 89.3 ± 42.3° |
   | HIS | 23.6 ± 33.1° | 28.0 ± 38.9° | 26.5 ± 32.6° | 86.9 ± 45.3° | 35.7 ± 34.3° | 83.1 ± 34.6° |
   | ILE | 13.9 ± 16.3° | 17.3 ± 28.8° | 13.3 ± 13.7° | 79.9 ± 38.8° | 29.2 ± 25.2° | 92.4 ± 26.0° |
   | LYS | 18.0 ± 26.6° | 22.5 ± 38.1° | 16.6 ± 22.3° | 92.7 ± 42.2° | 25.1 ± 27.3° | 90.2 ± 29.9° |
   | LEU | 14.6 ± 17.0° | 18.6 ± 31.8° | 13.2 ± 15.2° | 88.6 ± 38.6° | 24.7 ± 23.0° | 92.0 ± 26.4° |
   | MET | 17.1 ± 21.8° | 24.2 ± 38.9° | 15.8 ± 22.6° | 89.3 ± 41.5° | 25.6 ± 26.5° | 93.7 ± 30.1° |
   | ASN | 26.9 ± 39.3° | 29.0 ± 41.1° | 25.9 ± 33.3° | 91.0 ± 42.0° | 35.1 ± 39.8° | 78.1 ± 35.0° |
   | PRO | 17.8 ± 25.9° | 35.6 ± 51.7° | 15.3 ± 18.6° | 75.3 ± 53.0° | 16.3 ± 11.8° | 85.5 ± 29.1° |
   | GLN | 16.5 ± 21.9° | 21.5 ± 37.1° | 14.1 ± 18.1° | 92.5 ± 39.2° | 24.1 ± 24.9° | 90.5 ± 29.9° |
   | ARG | 16.4 ± 24.2° | 18.9 ± 31.9° | 15.2 ± 19.9° | 89.2 ± 40.5° | 26.2 ± 26.6° | 91.1 ± 27.8° |
   | SER | 21.6 ± 29.5° | 29.0 ± 43.8° | 20.2 ± 25.6° | 79.0 ± 49.3° | 28.0 ± 27.2° | 89.8 ± 29.5° |
   | THR | 17.1 ± 21.1° | 21.5 ± 34.5° | 17.1 ± 19.9° | 77.5 ± 46.7° | 30.8 ± 25.9° | 91.2 ± 28.6° |
   | VAL | 14.5 ± 15.2° | 19.5 ± 32.8° | 14.2 ± 13.1° | 77.8 ± 40.4° | 35.7 ± 26.5° | 91.7 ± 26.7° |
   | TRP | 17.4 ± 22.4° | 19.3 ± 30.4° | 17.0 ± 19.3° | 79.6 ± 43.7° | 32.6 ± 29.7° | 91.9 ± 26.5° |
   | TYR | 18.3 ± 21.6° | 20.1 ± 29.0° | 17.9 ± 19.4° | 80.7 ± 44.7° | 32.5 ± 27.1° | 91.4 ± 29.5° |
   |  | | | | | | | |
3. Ramachandran plots

   Here we show the Ramachandran distributions for all predicted and native torsion
   angles in regions of any confidence (C≥0).

   |  |
   | --- |
   |  |
   |  |
4. Correlation plots

   Shown is the correlation between native and predicted torsion angles
   in regions of high confidence (C>0.8).

   |  |
   | --- |
   | Since torsion angles are circular quantities, we have applied a correction similar to the one used in the calculation of MAE. Whenever the difference between a predicted and native angle was outside of the [-180, +180°] range, we subtracted or added 360° before measuring the correlation. |
   |  |
5. The HHsearch score

   We demonstrate the lack of reliable correlation between the HHseach probability
   score and the structural compatibility of short fragments (L≤12).

   |  |
   | --- |
   |  |
   |  |
