# Supplementary figures and images for: Confidence-Guided Local Structure Prediction with HHfrag (part 2 of 2)
Source: PLoS One. 2013 Oct 16;8(10):e76512. doi: 10.1371/journal.pone.0076512 (PMC3797814; doi:10.1371/journal.pone.0076512)

# 3n6zA

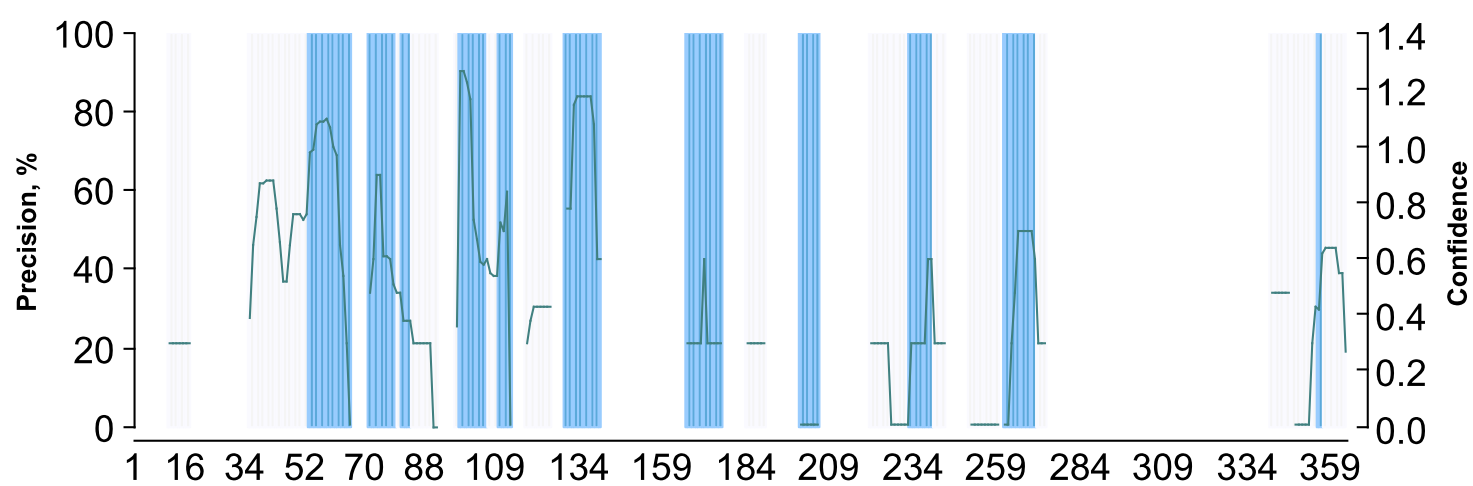

Supplement: Benchmark S1 — Local centroid precision for each target in the benchmark set and a breakdown of the torsion angle prediction performance by residue type and secondary structure. (ZIP) [file pone.0076512.s001.zip › Filtering/3n6zA.pdf]

### 3nr1A

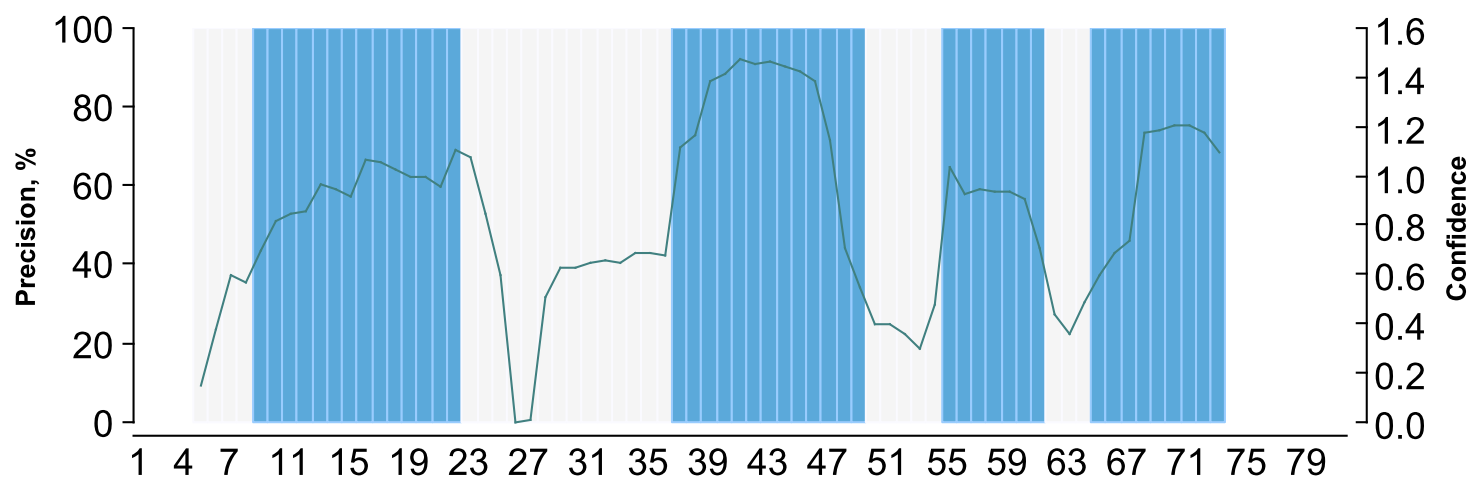

Supplement: Benchmark S1 — Local centroid precision for each target in the benchmark set and a breakdown of the torsion angle prediction performance by residue type and secondary structure. (ZIP) [file pone.0076512.s001.zip › Filtering/3nrlA.pdf]

3n8uA

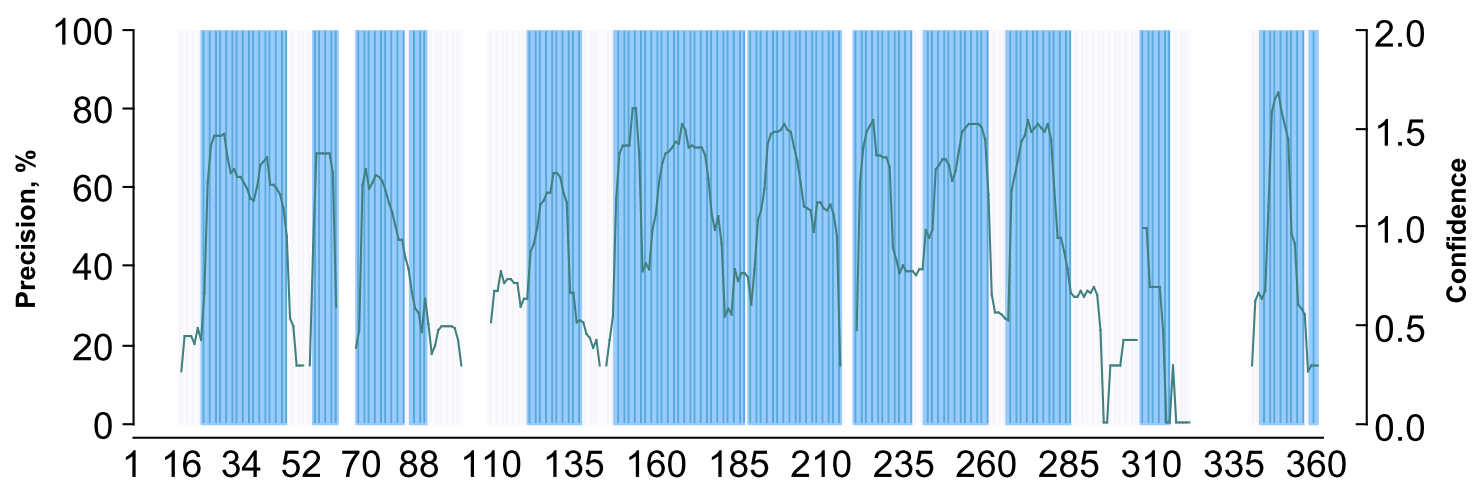

Supplement: Benchmark S1 — Local centroid precision for each target in the benchmark set and a breakdown of the torsion angle prediction performance by residue type and secondary structure. (ZIP) [file pone.0076512.s001.zip › Filtering/3n8uA.pdf]

3mwta

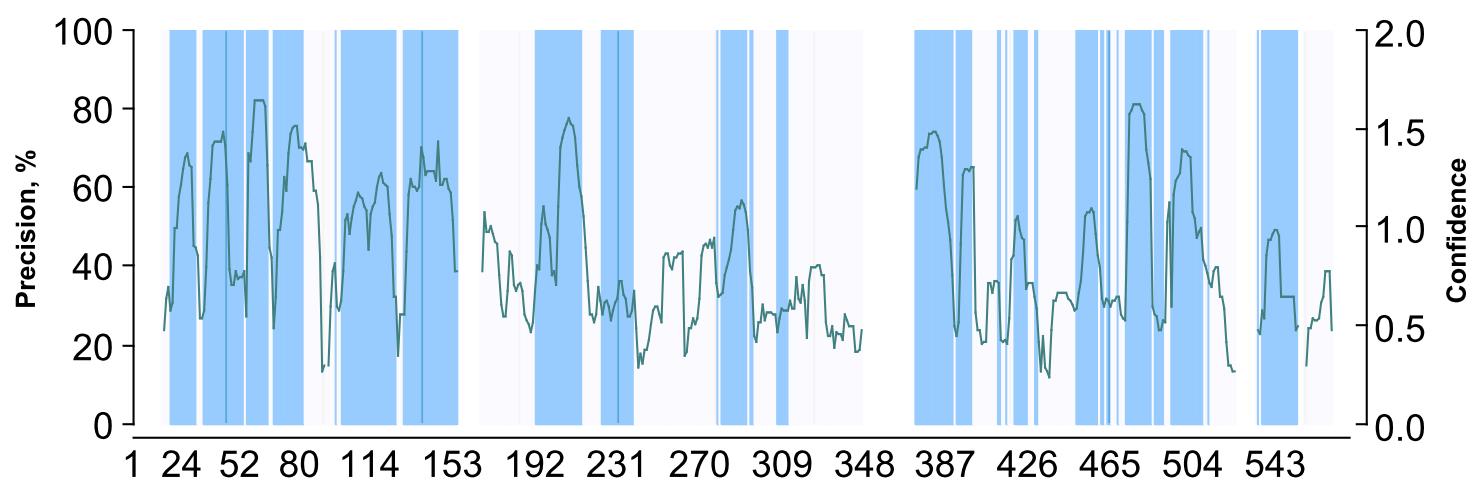

Supplement: Benchmark S1 — Local centroid precision for each target in the benchmark set and a breakdown of the torsion angle prediction performance by residue type and secondary structure. (ZIP) [file pone.0076512.s001.zip › Filtering/3mwtA.pdf]

### 3natA

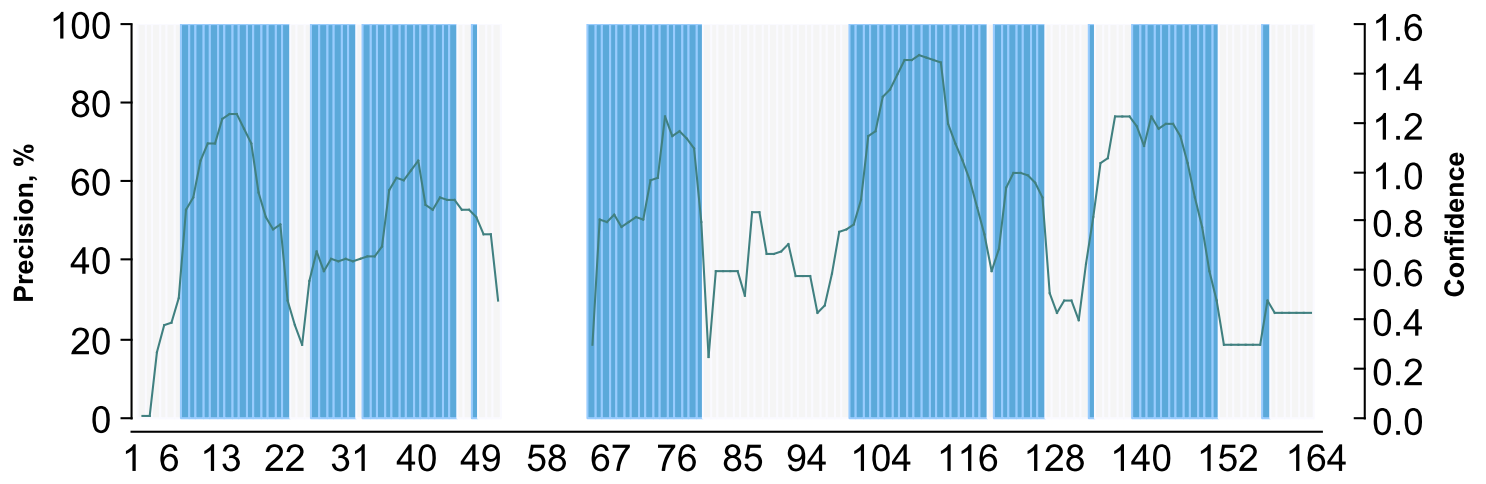

Supplement: Benchmark S1 — Local centroid precision for each target in the benchmark set and a breakdown of the torsion angle prediction performance by residue type and secondary structure. (ZIP) [file pone.0076512.s001.zip › Filtering/3natA.pdf]

# 300xA

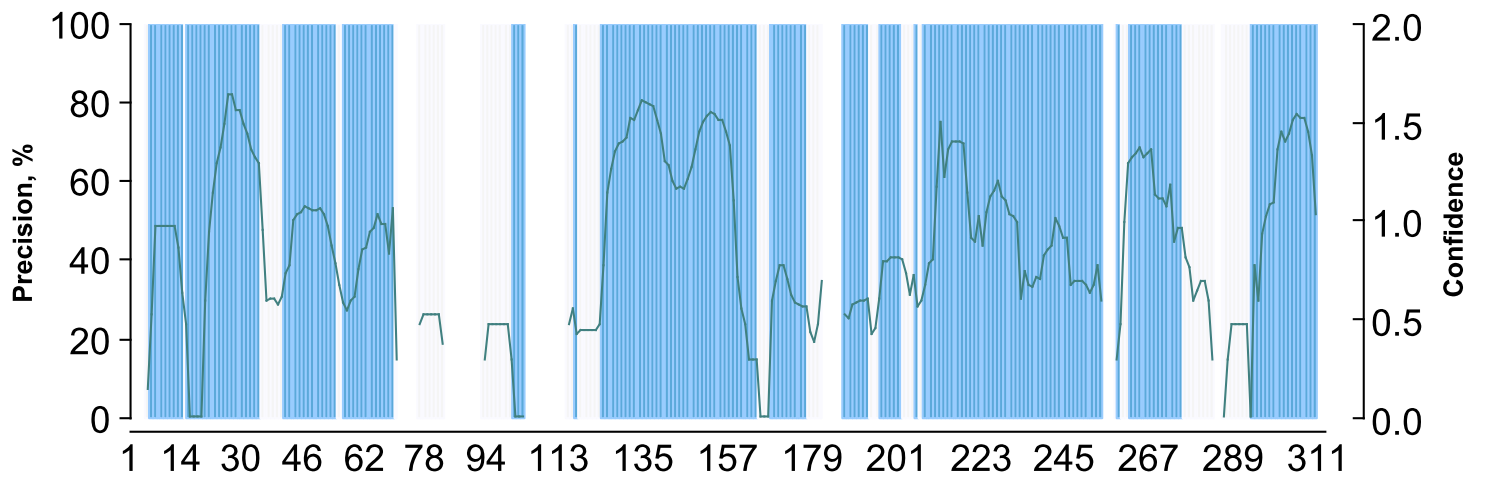

Supplement: Benchmark S1 — Local centroid precision for each target in the benchmark set and a breakdown of the torsion angle prediction performance by residue type and secondary structure. (ZIP) [file pone.0076512.s001.zip › Filtering/3ooxA.pdf]

3nppA

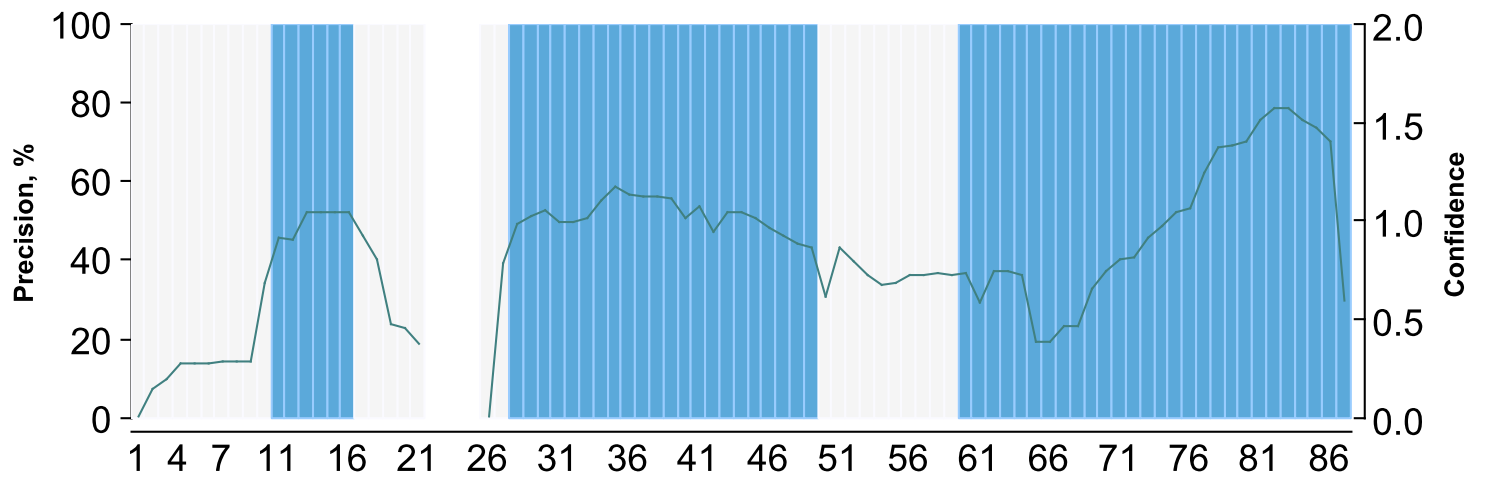

Supplement: Benchmark S1 — Local centroid precision for each target in the benchmark set and a breakdown of the torsion angle prediction performance by residue type and secondary structure. (ZIP) [file pone.0076512.s001.zip › Filtering/3nppA.pdf]

# 3pfeA

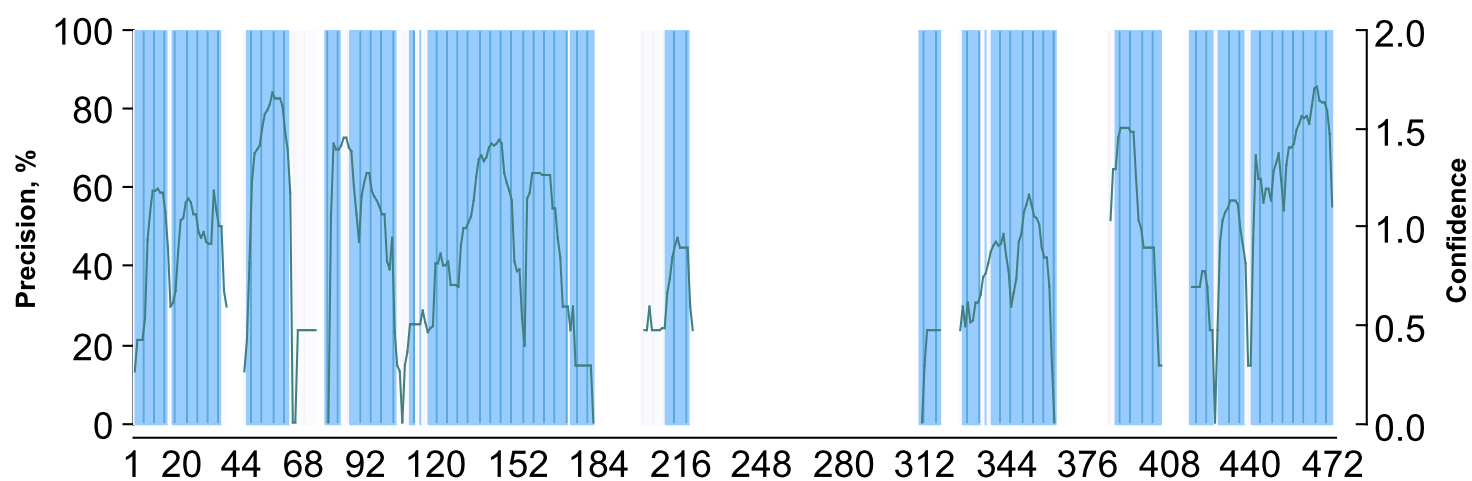

Supplement: Benchmark S1 — Local centroid precision for each target in the benchmark set and a breakdown of the torsion angle prediction performance by residue type and secondary structure. (ZIP) [file pone.0076512.s001.zip › Filtering/3pfeA.pdf]

2kjaA

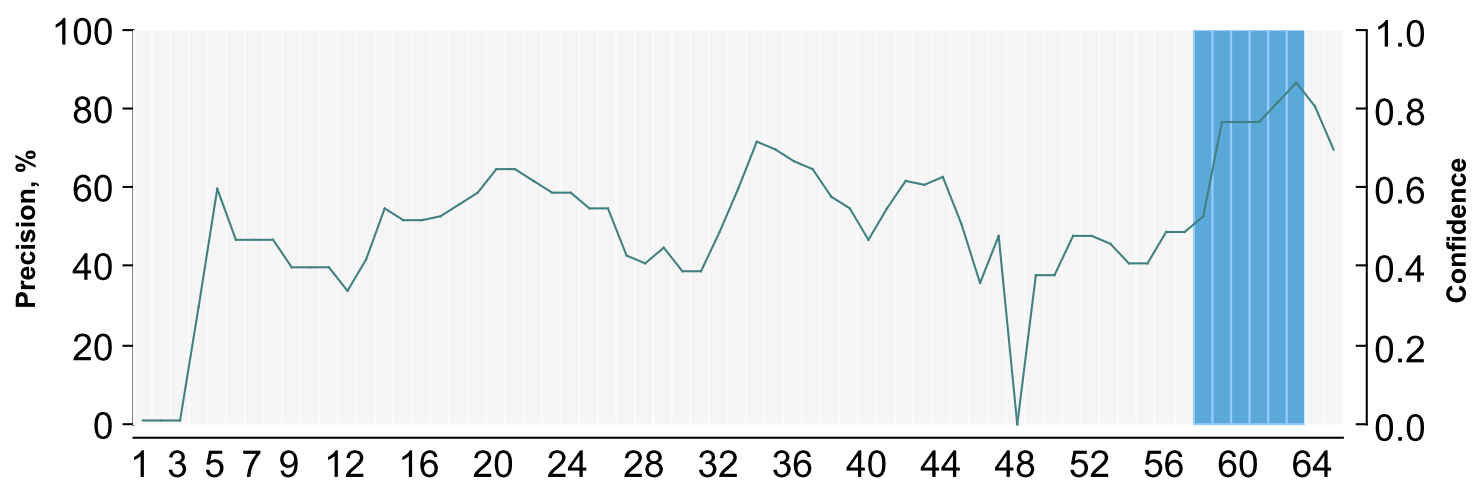

Supplement: Benchmark S1 — Local centroid precision for each target in the benchmark set and a breakdown of the torsion angle prediction performance by residue type and secondary structure. (ZIP) [file pone.0076512.s001.zip › Filtering/2kjxA.pdf]

# 3nymA

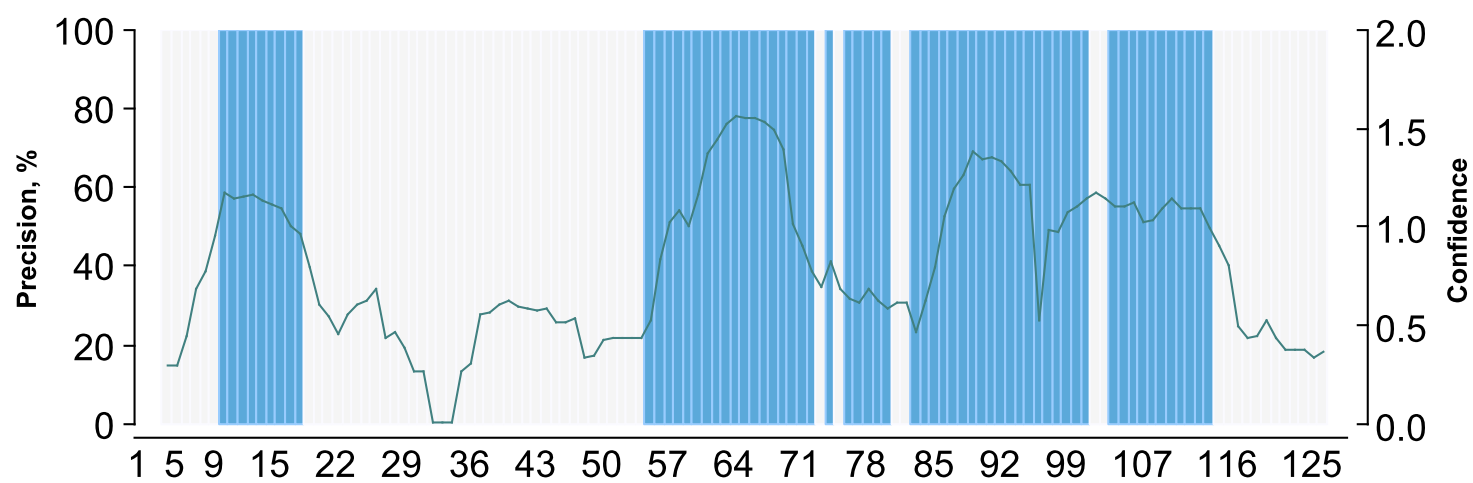

Supplement: Benchmark S1 — Local centroid precision for each target in the benchmark set and a breakdown of the torsion angle prediction performance by residue type and secondary structure. (ZIP) [file pone.0076512.s001.zip › Filtering/3nymA.pdf]

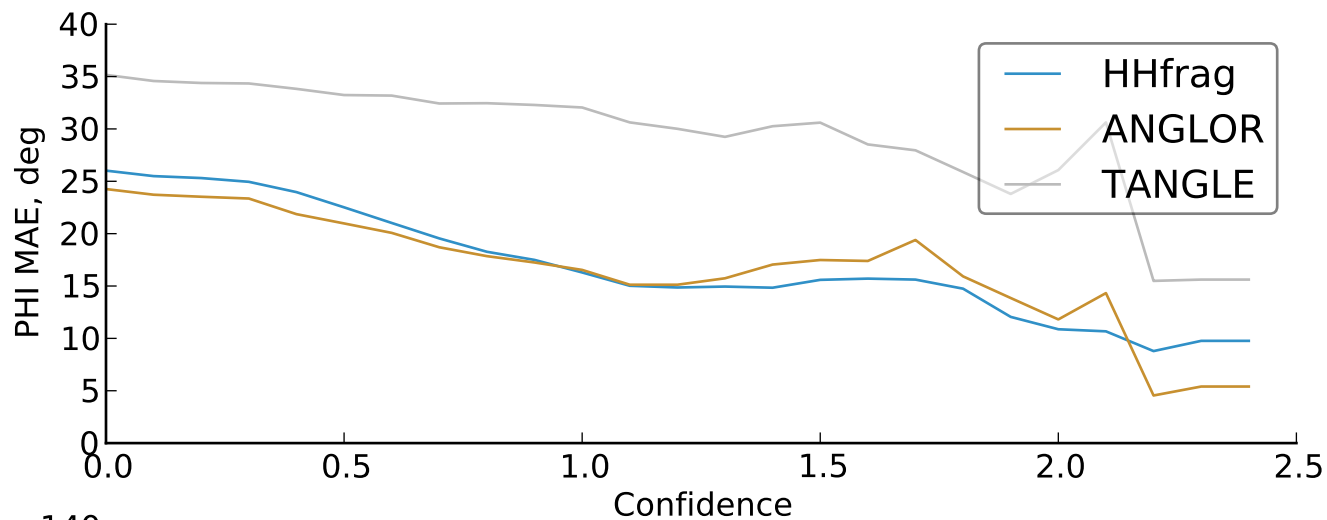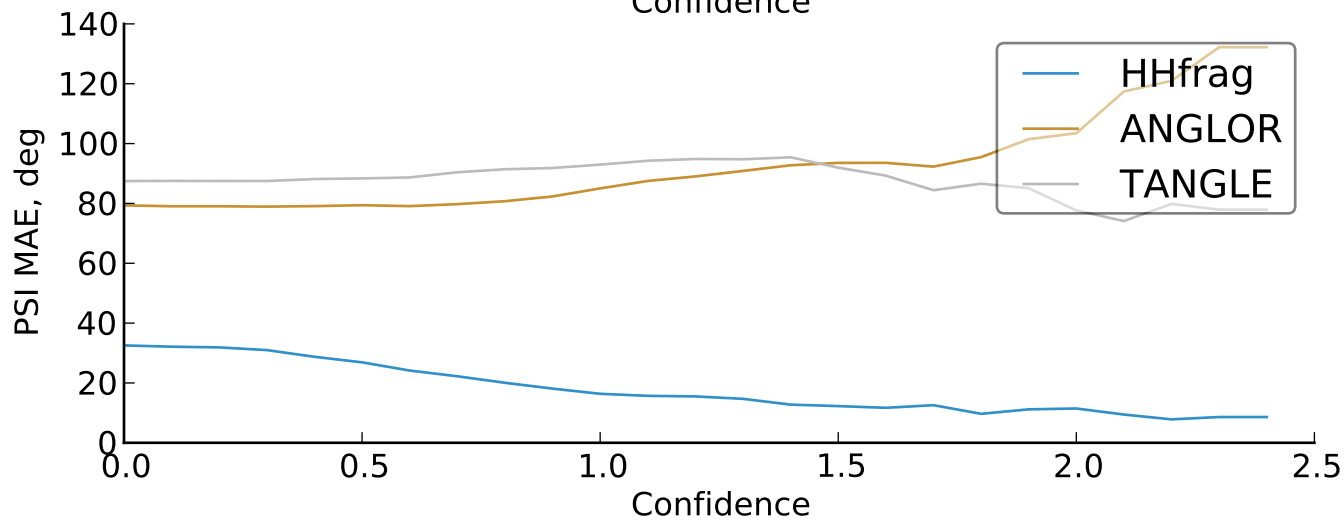

Supplement: Benchmark S1 — Local centroid precision for each target in the benchmark set and a breakdown of the torsion angle prediction performance by residue type and secondary structure. (ZIP) [file pone.0076512.s001.zip › MAE/TYR.pdf]

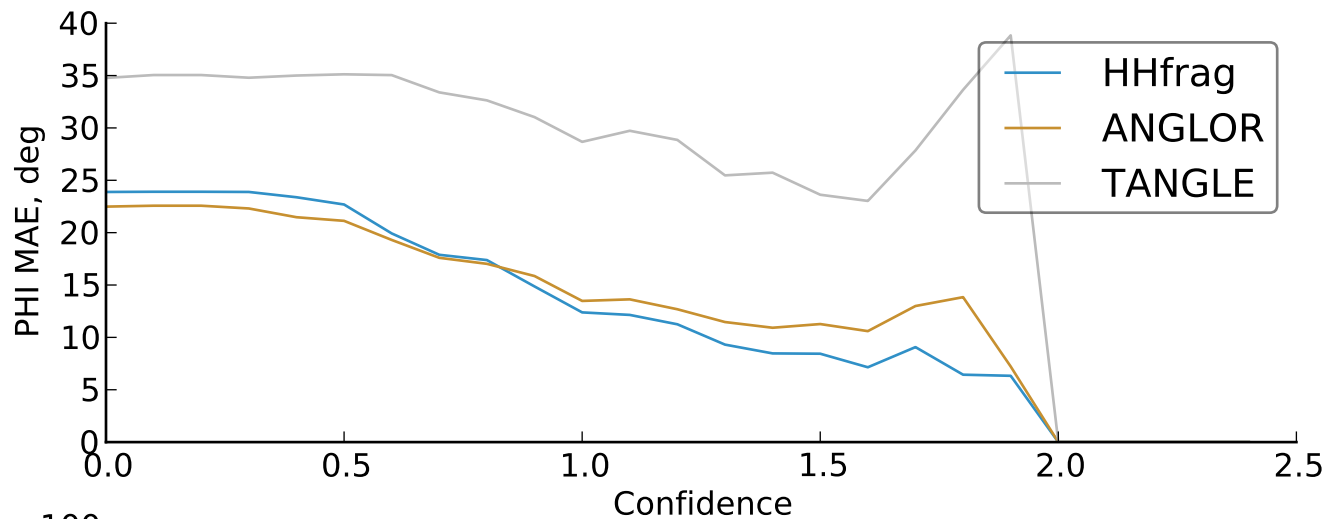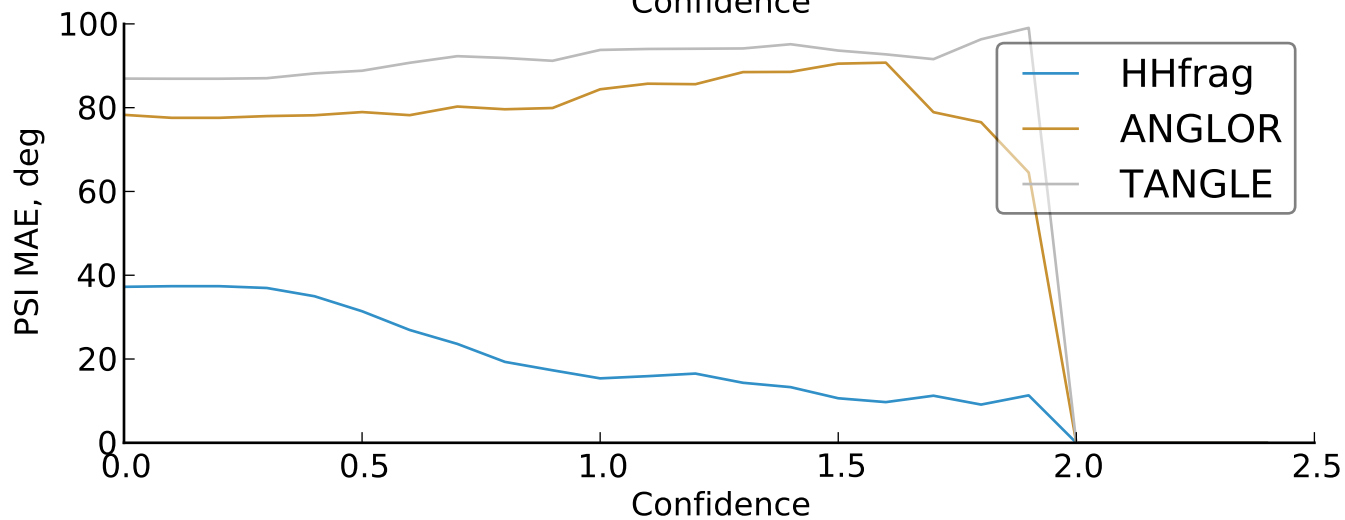

Supplement: Benchmark S1 — Local centroid precision for each target in the benchmark set and a breakdown of the torsion angle prediction performance by residue type and secondary structure. (ZIP) [file pone.0076512.s001.zip › MAE/TRP.pdf]

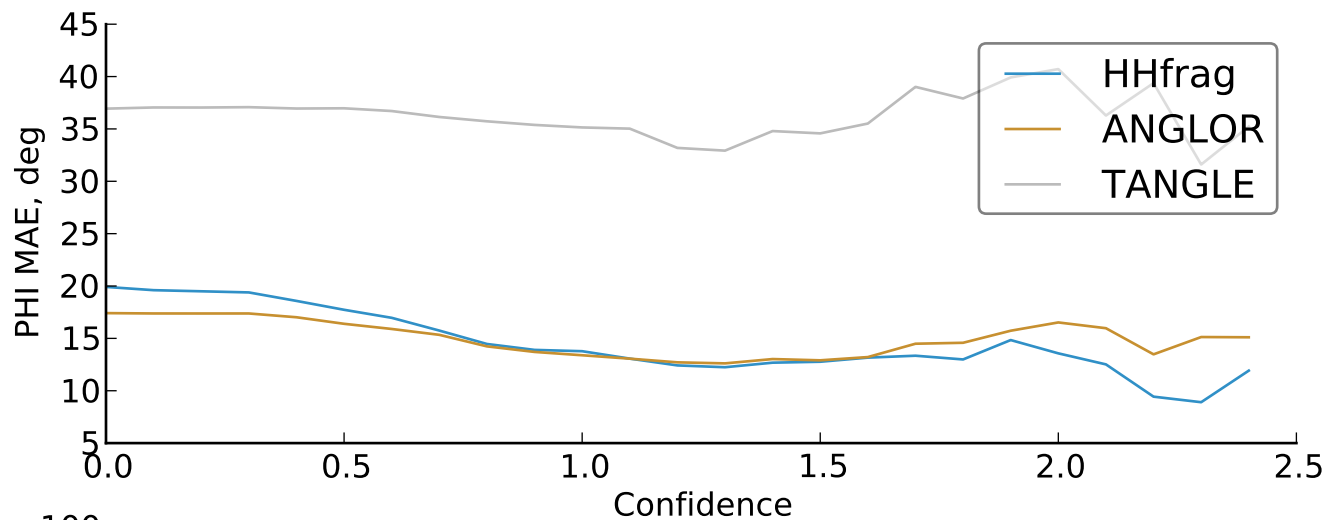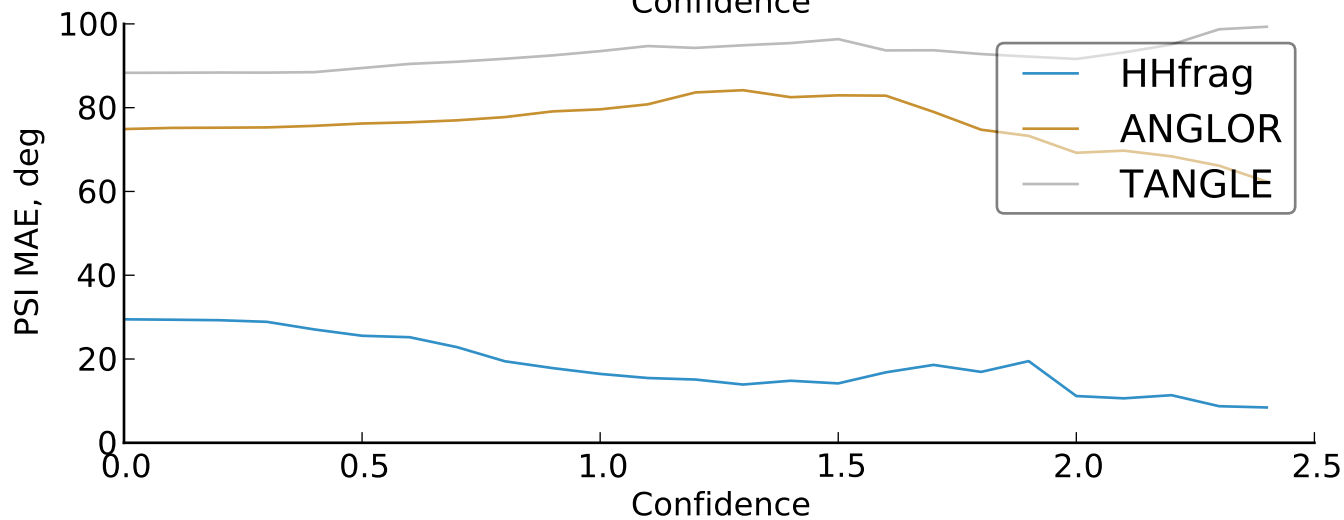

Supplement: Benchmark S1 — Local centroid precision for each target in the benchmark set and a breakdown of the torsion angle prediction performance by residue type and secondary structure. (ZIP) [file pone.0076512.s001.zip › MAE/VAL.pdf]

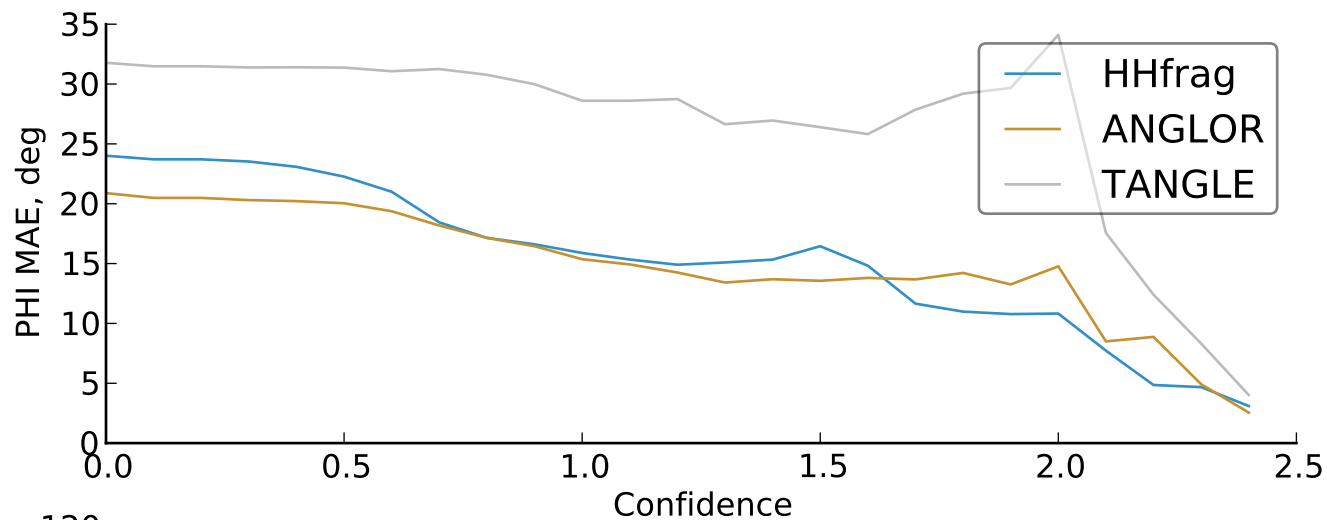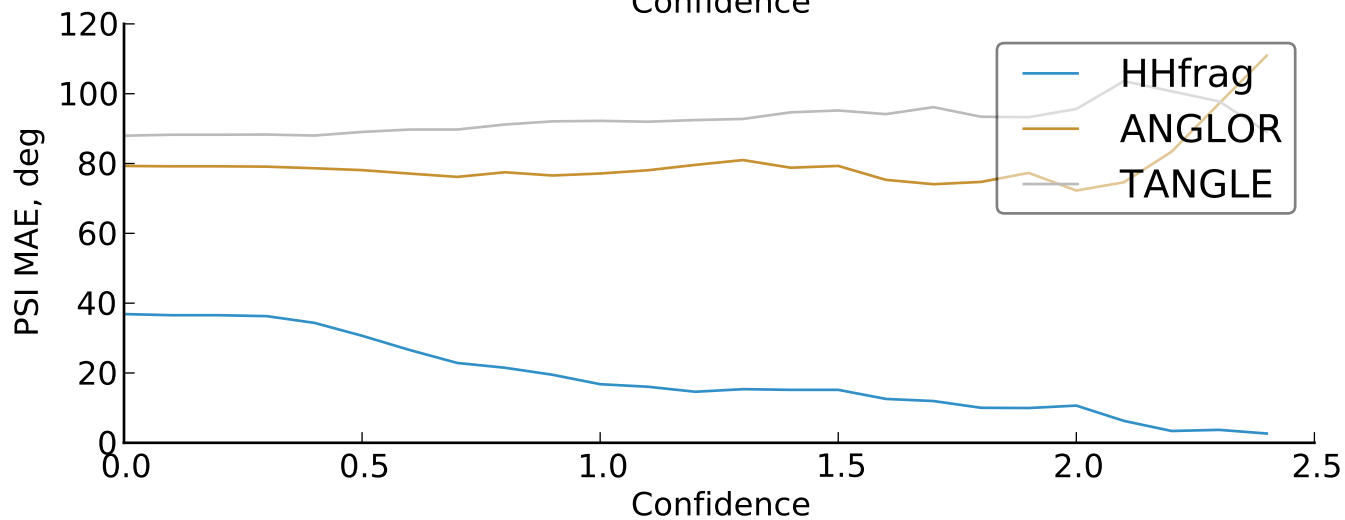

Supplement: Benchmark S1 — Local centroid precision for each target in the benchmark set and a breakdown of the torsion angle prediction performance by residue type and secondary structure. (ZIP) [file pone.0076512.s001.zip › MAE/THR.pdf]

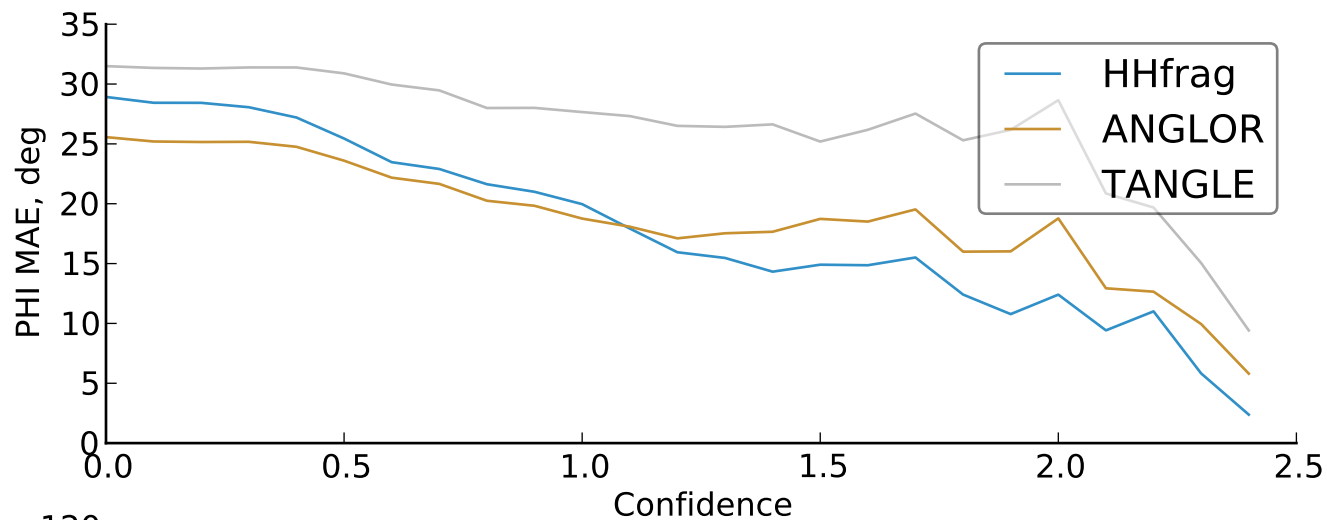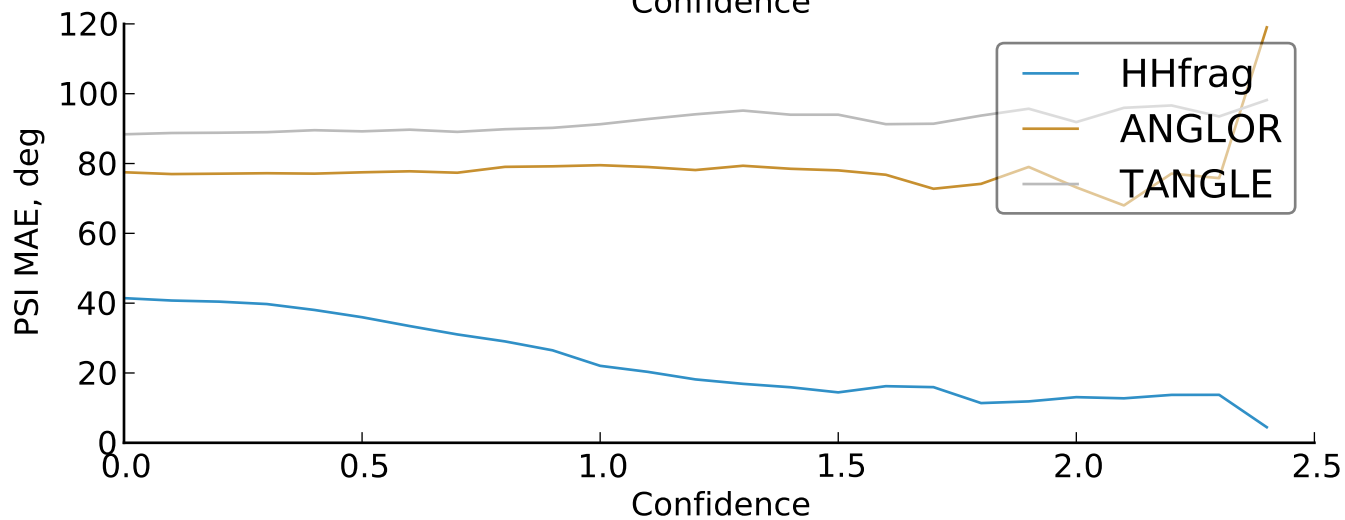

Supplement: Benchmark S1 — Local centroid precision for each target in the benchmark set and a breakdown of the torsion angle prediction performance by residue type and secondary structure. (ZIP) [file pone.0076512.s001.zip › MAE/SER.pdf]

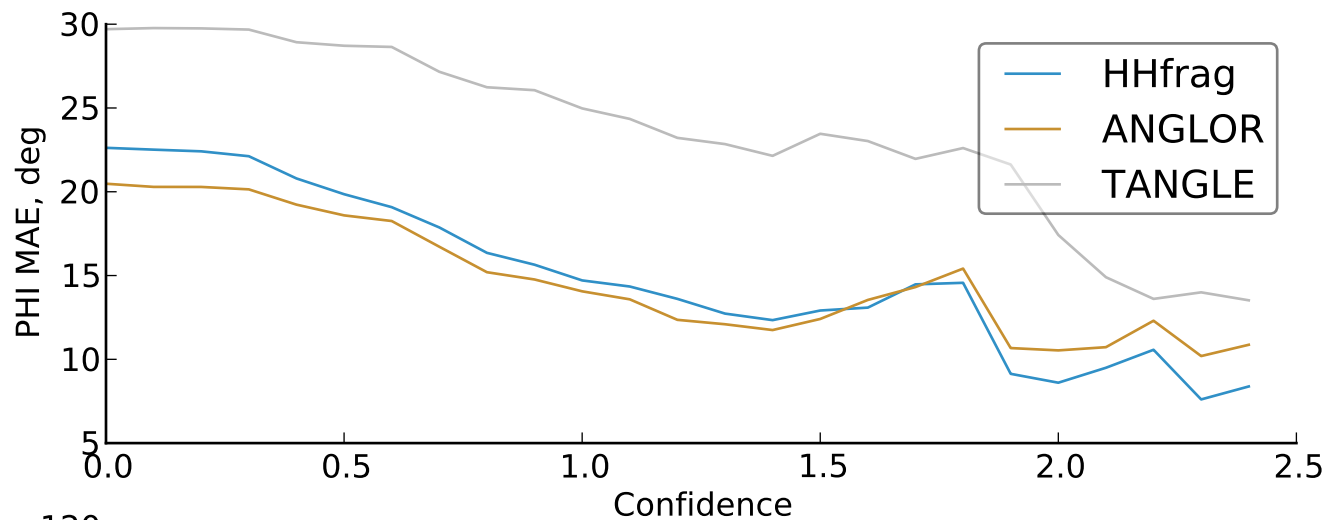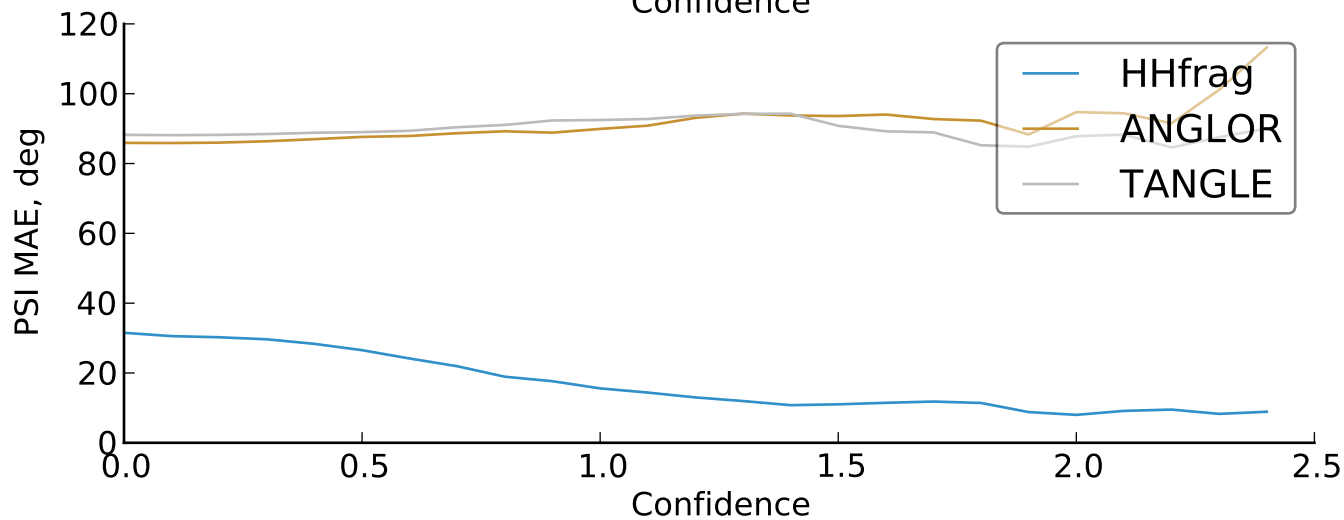

Supplement: Benchmark S1 — Local centroid precision for each target in the benchmark set and a breakdown of the torsion angle prediction performance by residue type and secondary structure. (ZIP) [file pone.0076512.s001.zip › MAE/ARG.pdf]

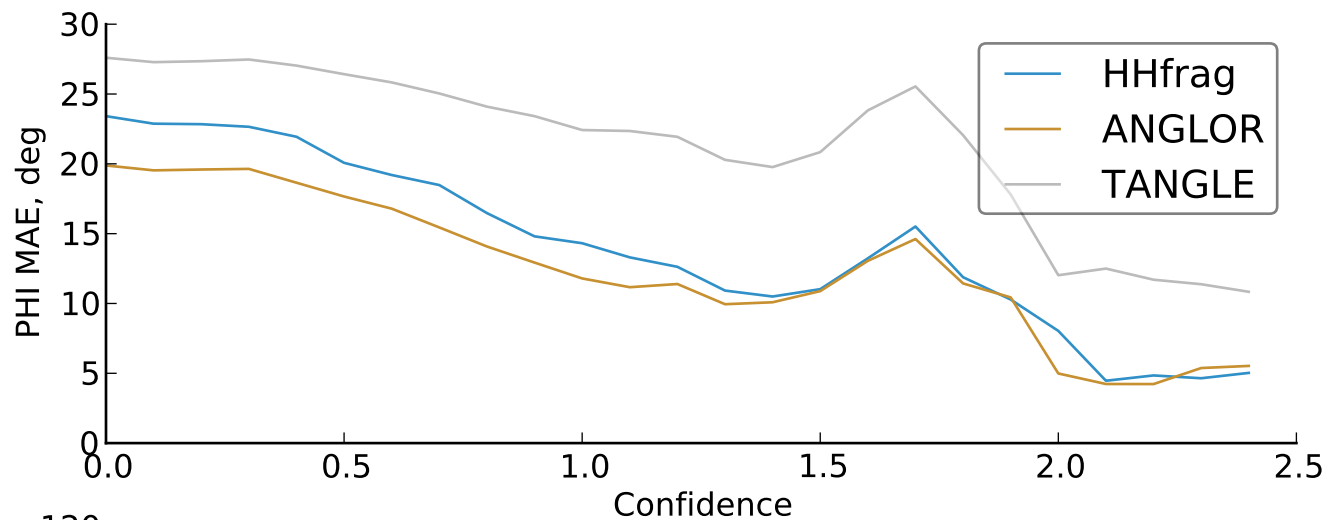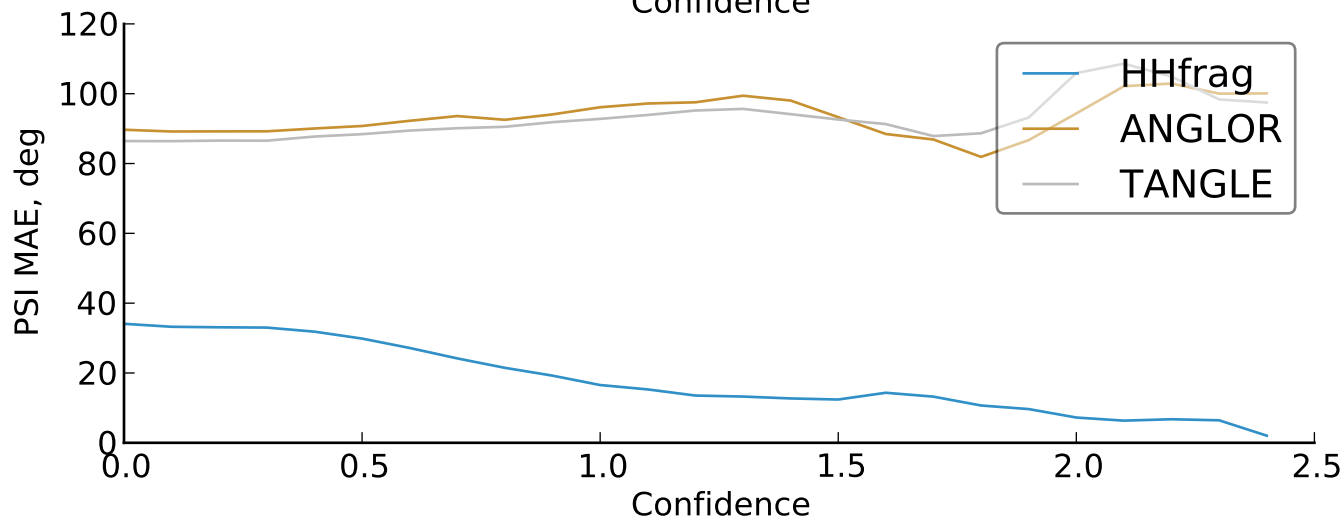

Supplement: Benchmark S1 — Local centroid precision for each target in the benchmark set and a breakdown of the torsion angle prediction performance by residue type and secondary structure. (ZIP) [file pone.0076512.s001.zip › MAE/GLN.pdf]

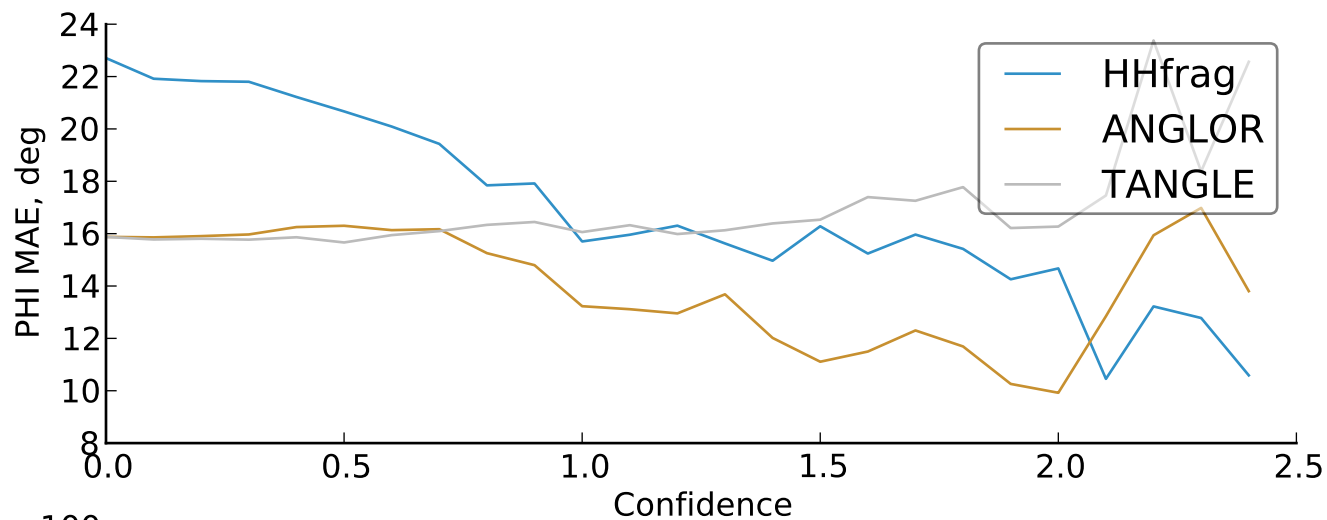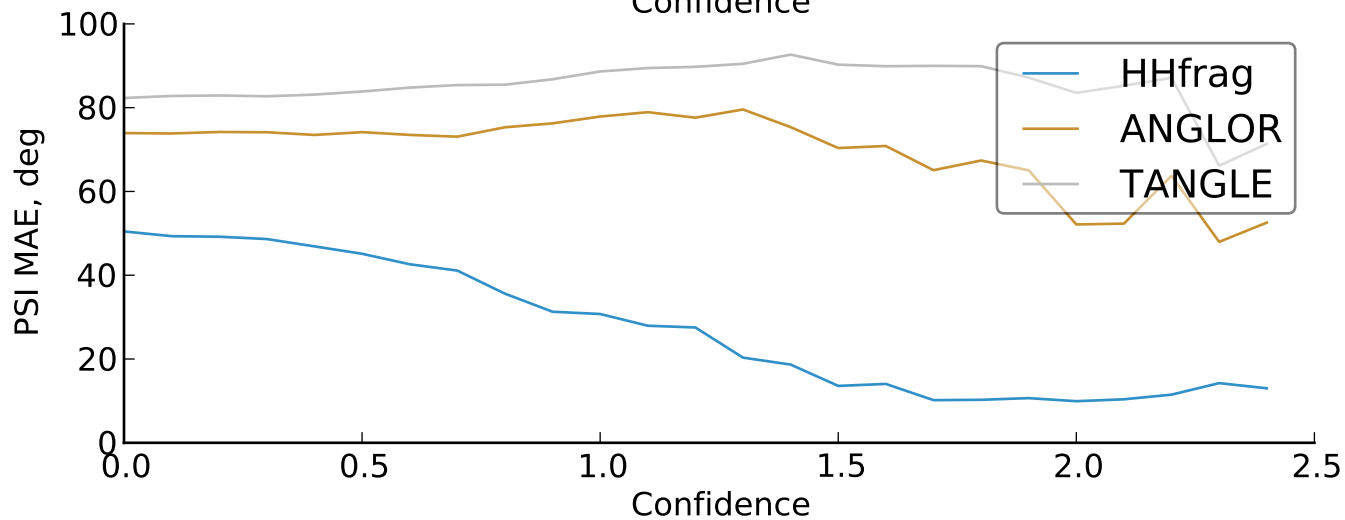

Supplement: Benchmark S1 — Local centroid precision for each target in the benchmark set and a breakdown of the torsion angle prediction performance by residue type and secondary structure. (ZIP) [file pone.0076512.s001.zip › MAE/PRO.pdf]

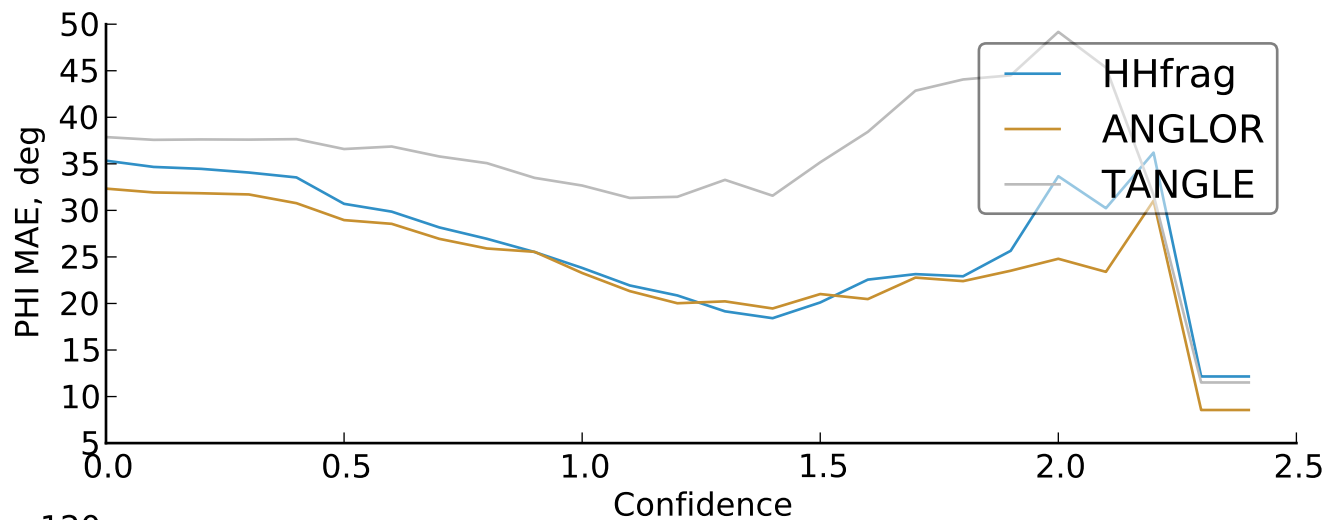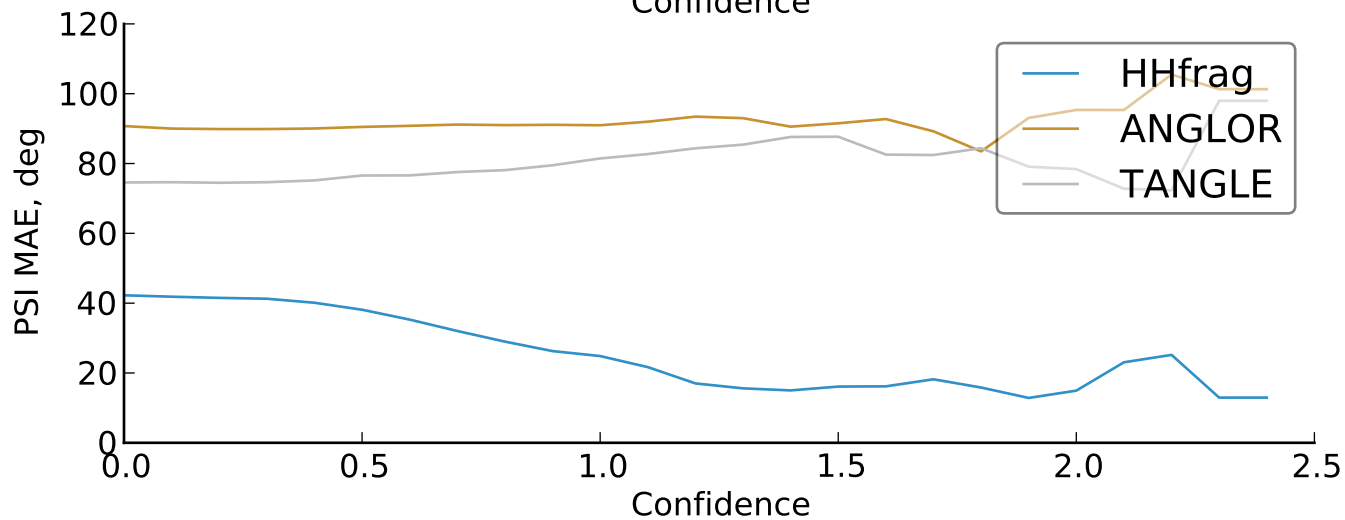

Supplement: Benchmark S1 — Local centroid precision for each target in the benchmark set and a breakdown of the torsion angle prediction performance by residue type and secondary structure. (ZIP) [file pone.0076512.s001.zip › MAE/ASN.pdf]

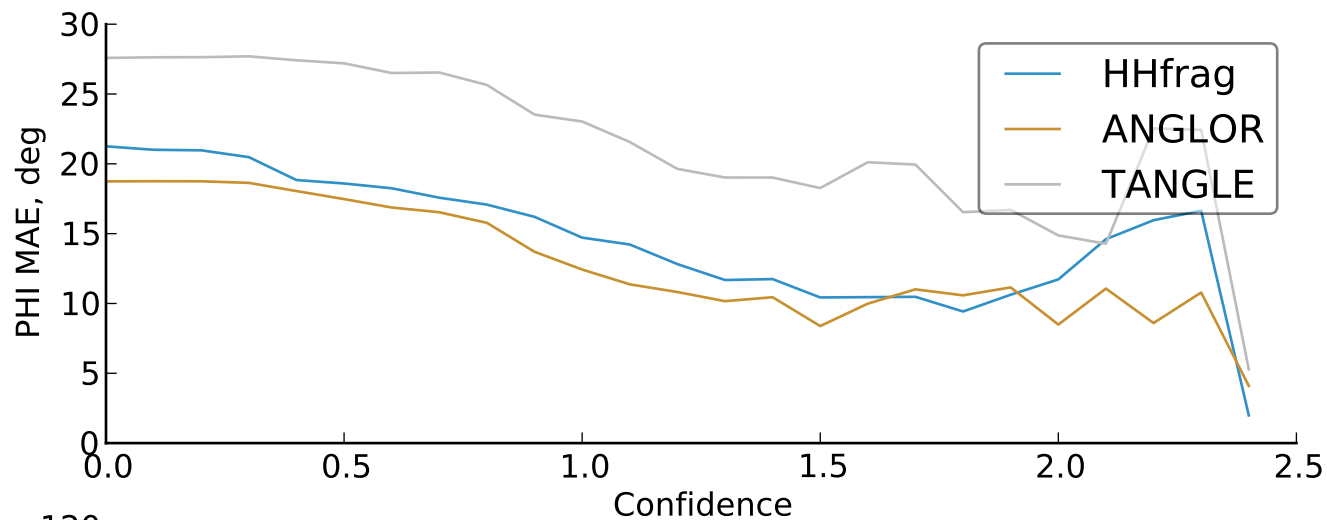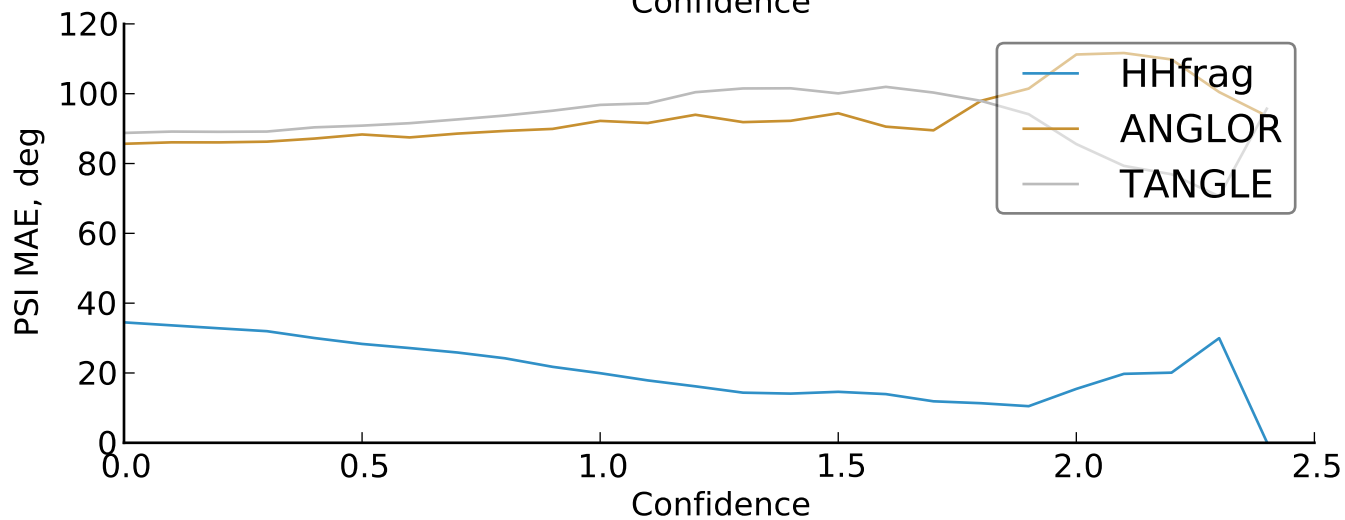

Supplement: Benchmark S1 — Local centroid precision for each target in the benchmark set and a breakdown of the torsion angle prediction performance by residue type and secondary structure. (ZIP) [file pone.0076512.s001.zip › MAE/MET.pdf]

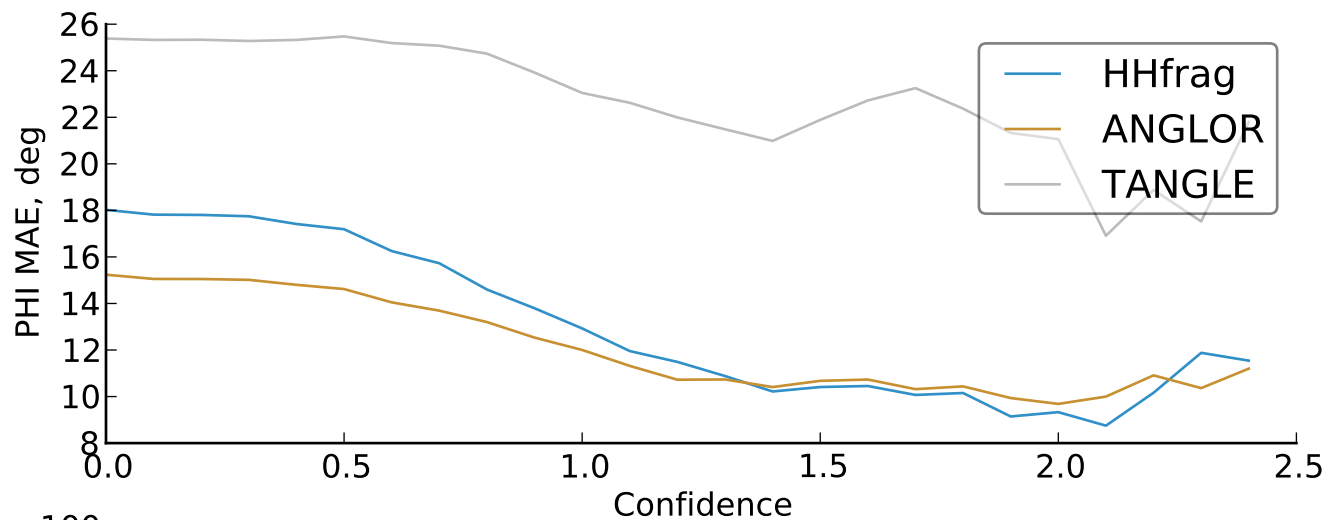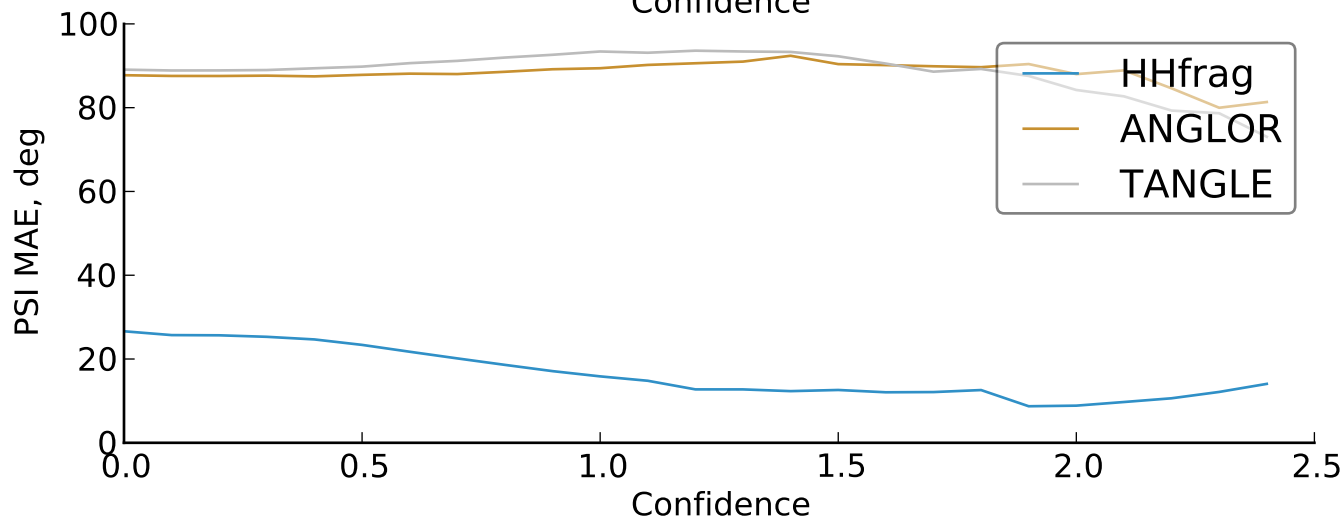

Supplement: Benchmark S1 — Local centroid precision for each target in the benchmark set and a breakdown of the torsion angle prediction performance by residue type and secondary structure. (ZIP) [file pone.0076512.s001.zip › MAE/LEU.pdf]

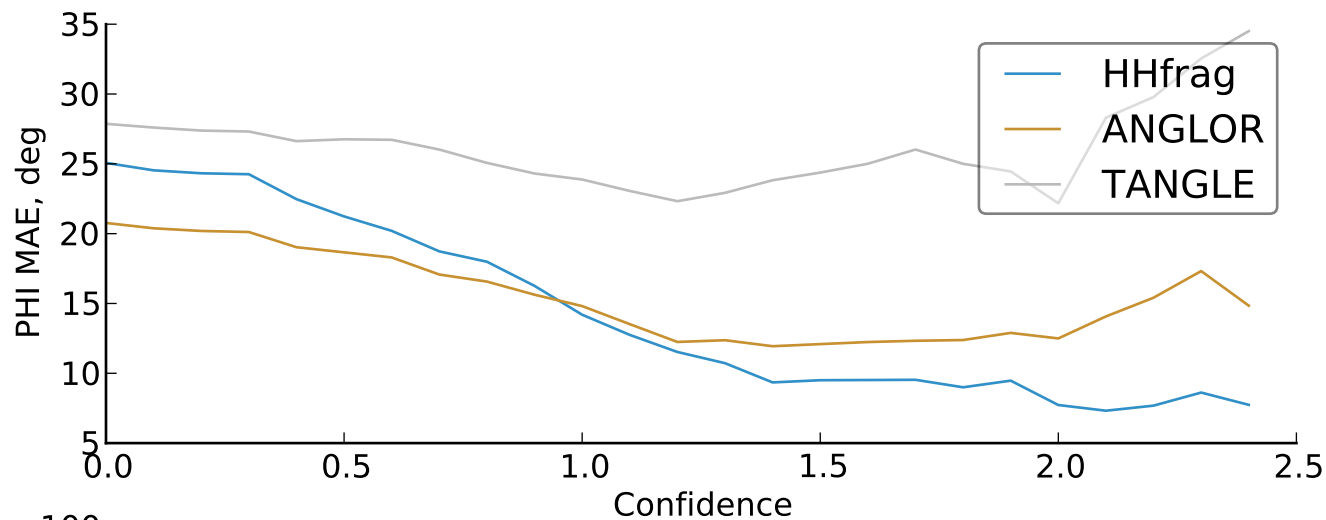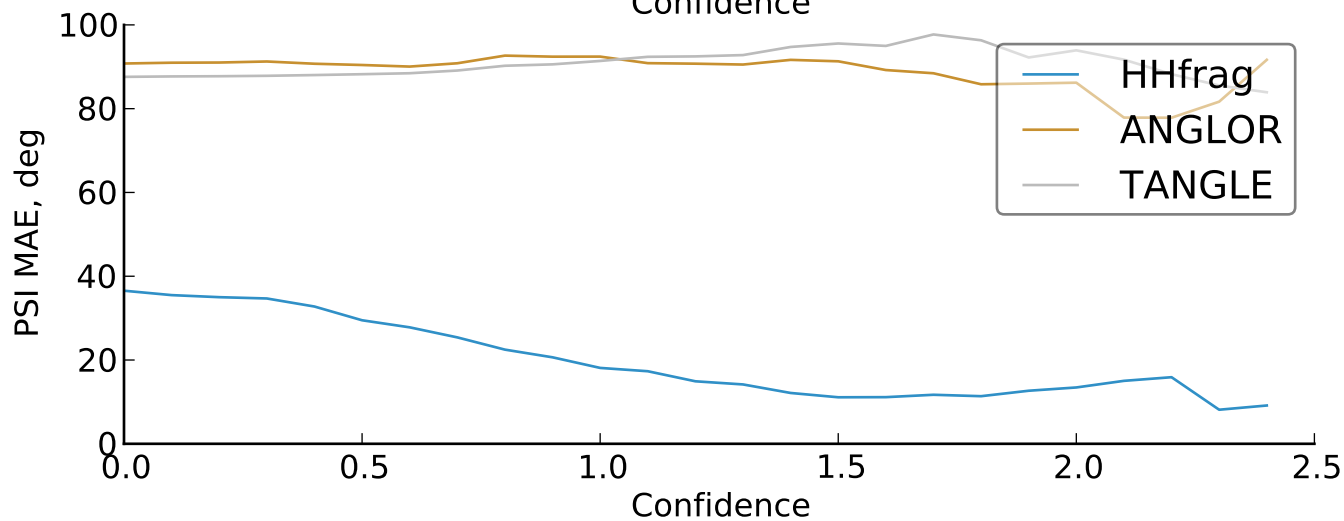

Supplement: Benchmark S1 — Local centroid precision for each target in the benchmark set and a breakdown of the torsion angle prediction performance by residue type and secondary structure. (ZIP) [file pone.0076512.s001.zip › MAE/LYS.pdf]

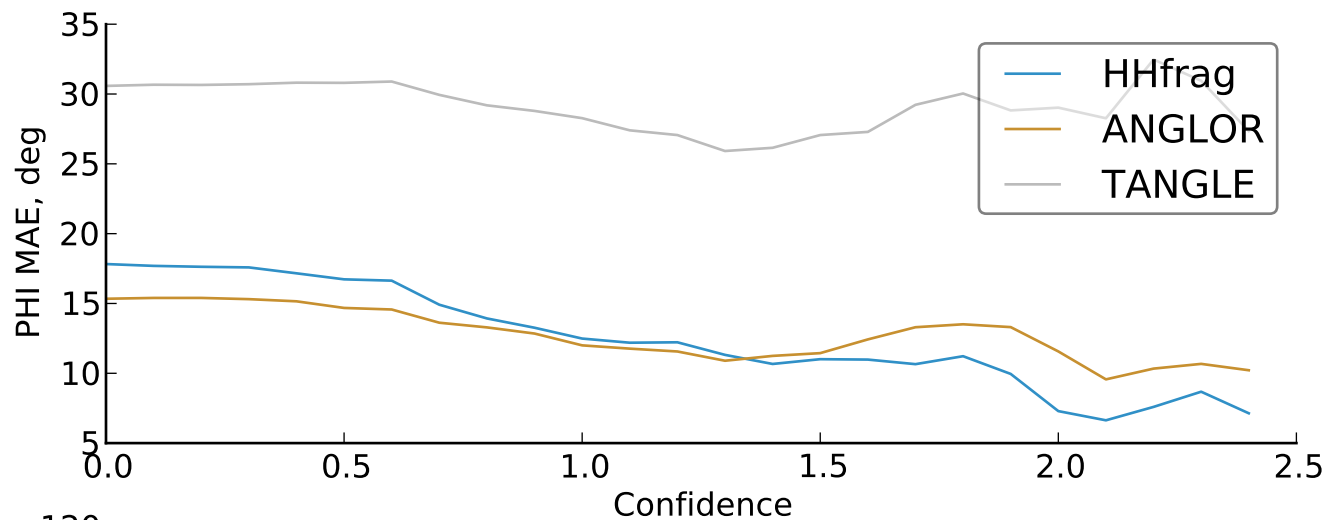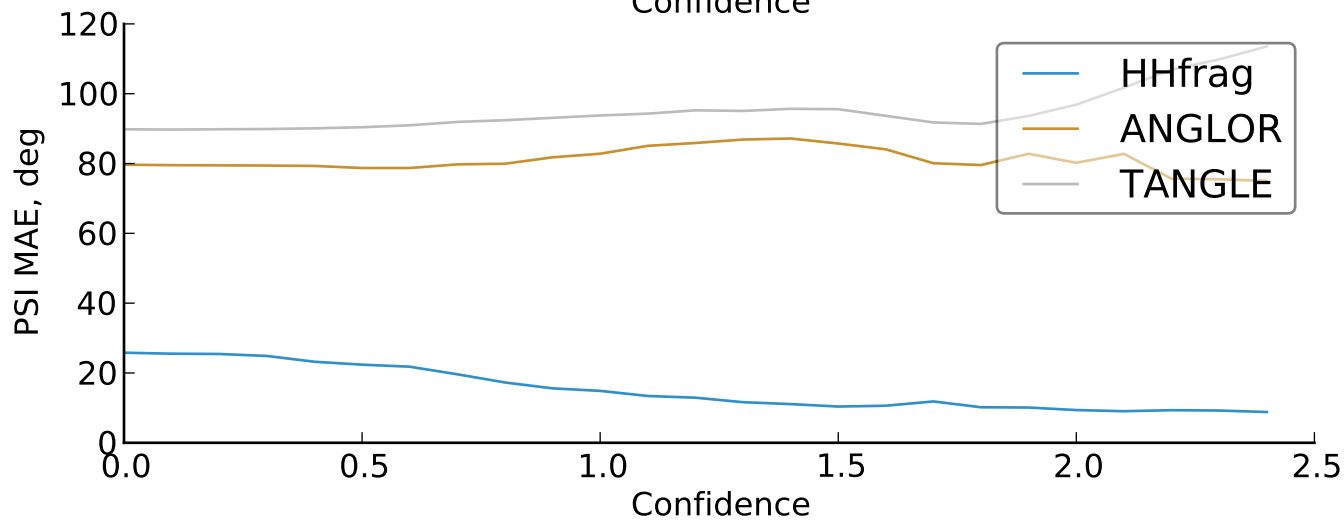

Supplement: Benchmark S1 — Local centroid precision for each target in the benchmark set and a breakdown of the torsion angle prediction performance by residue type and secondary structure. (ZIP) [file pone.0076512.s001.zip › MAE/ILE.pdf]

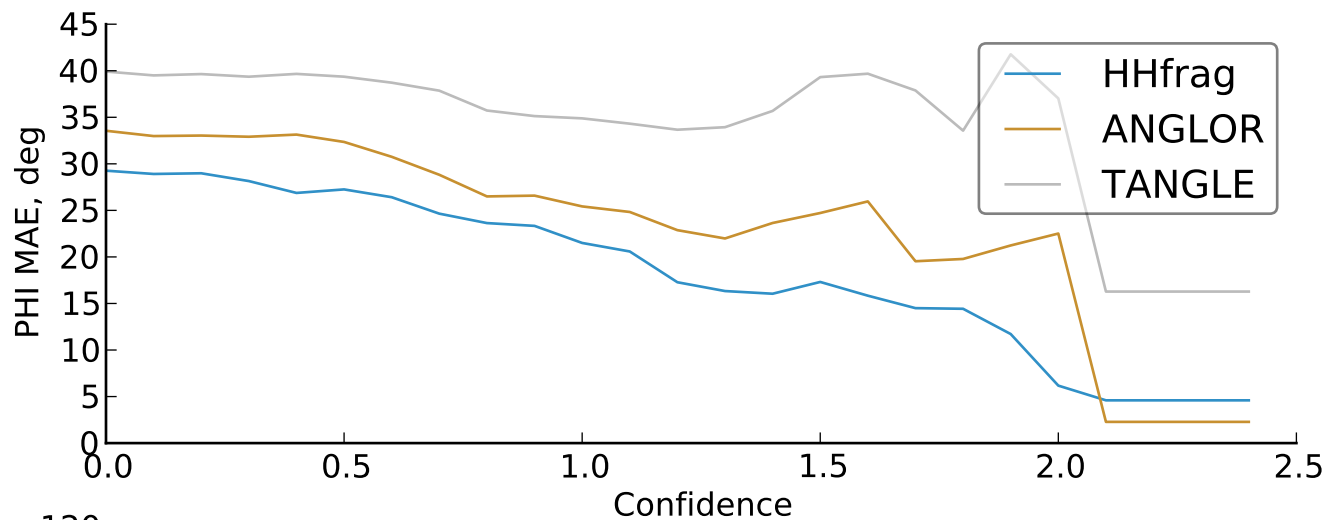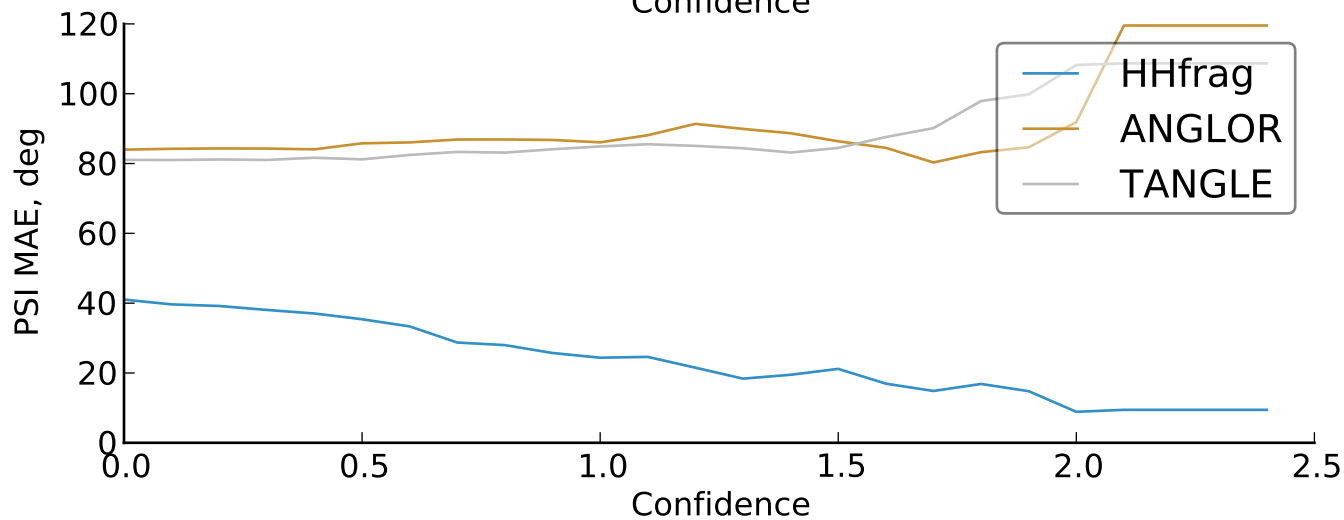

Supplement: Benchmark S1 — Local centroid precision for each target in the benchmark set and a breakdown of the torsion angle prediction performance by residue type and secondary structure. (ZIP) [file pone.0076512.s001.zip › MAE/HIS.pdf]

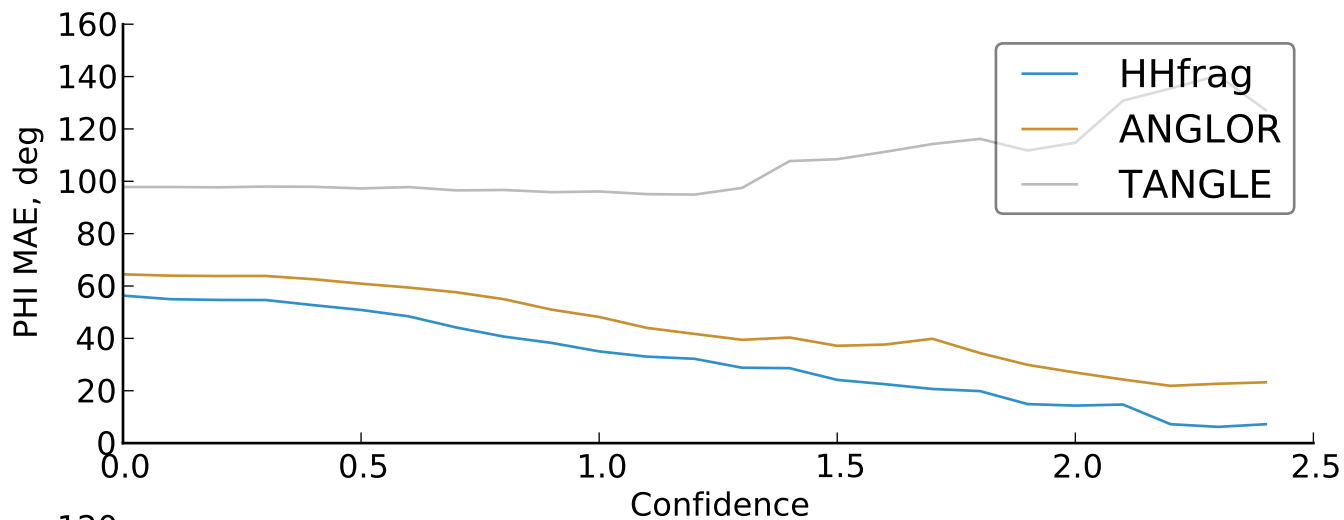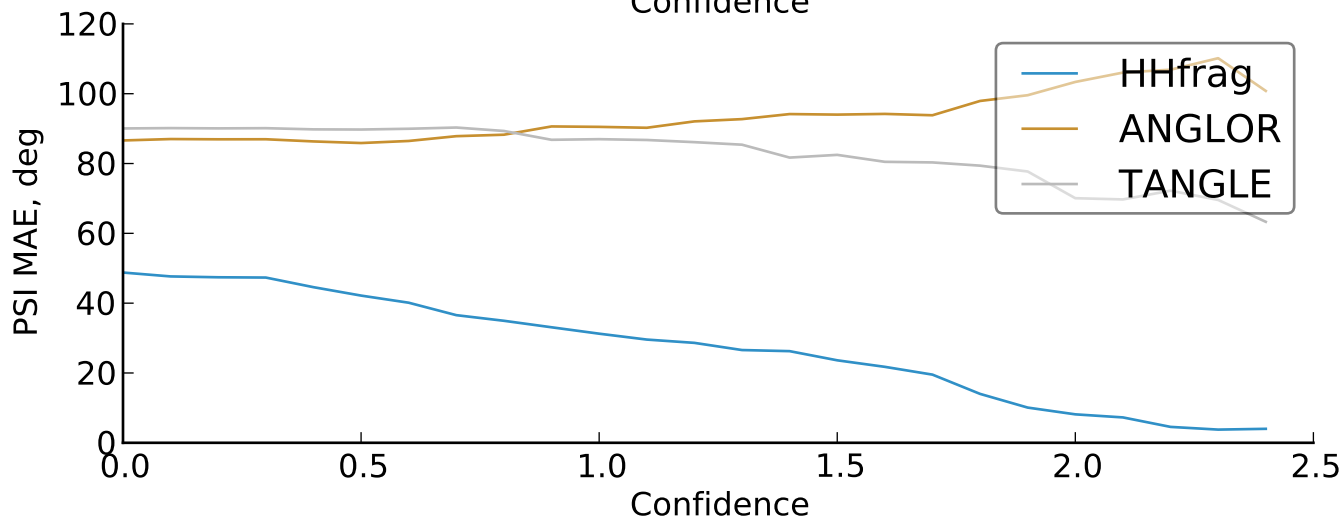

Supplement: Benchmark S1 — Local centroid precision for each target in the benchmark set and a breakdown of the torsion angle prediction performance by residue type and secondary structure. (ZIP) [file pone.0076512.s001.zip › MAE/GLY.pdf]

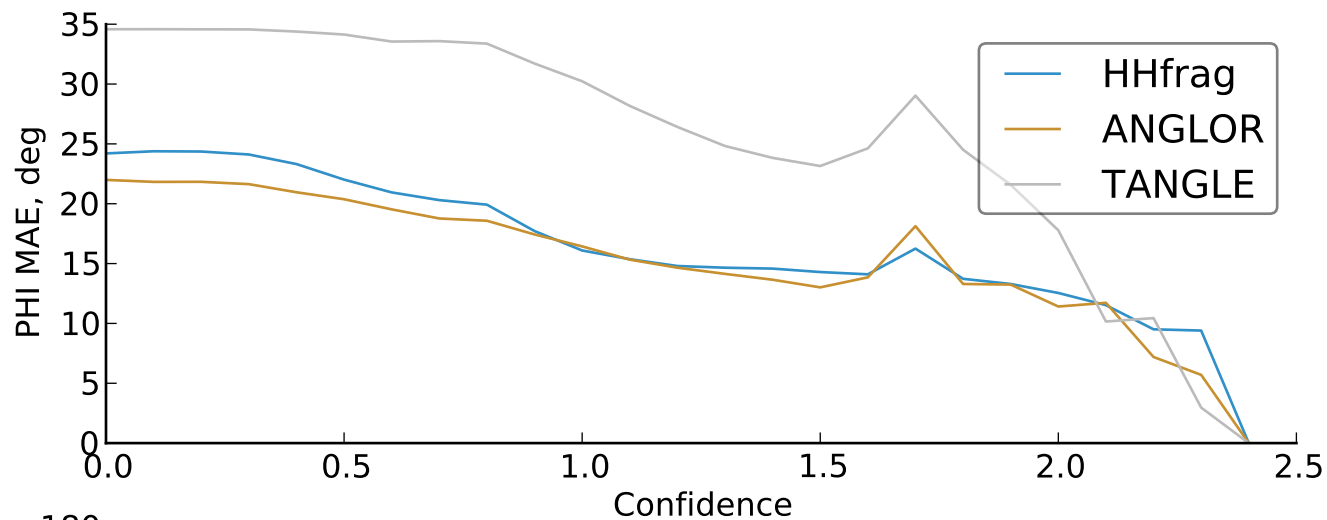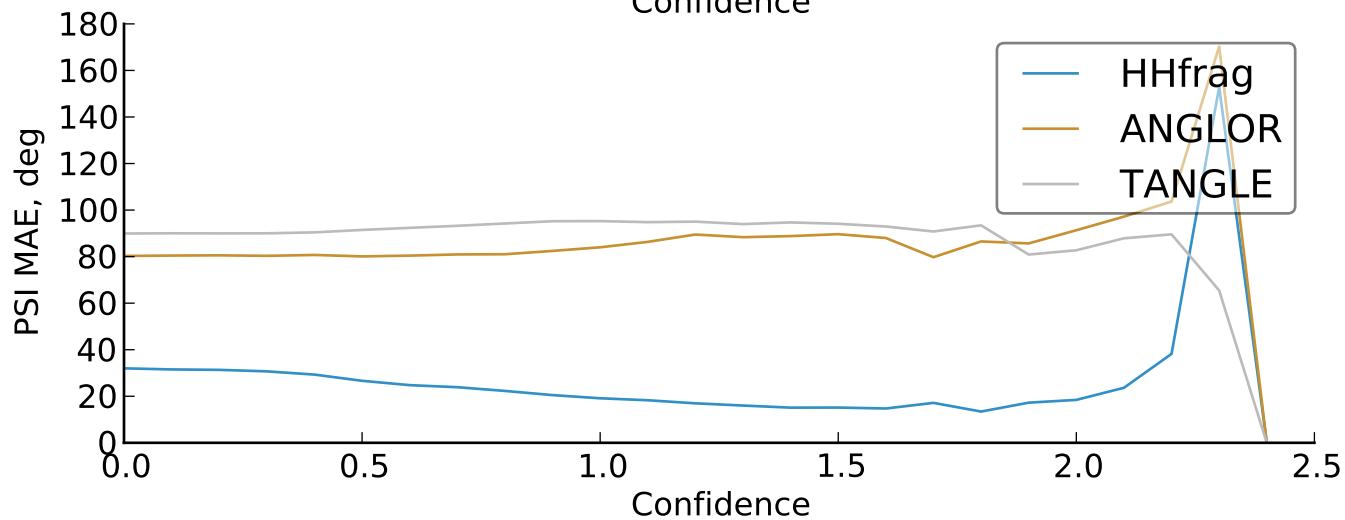

Supplement: Benchmark S1 — Local centroid precision for each target in the benchmark set and a breakdown of the torsion angle prediction performance by residue type and secondary structure. (ZIP) [file pone.0076512.s001.zip › MAE/PHE.pdf]

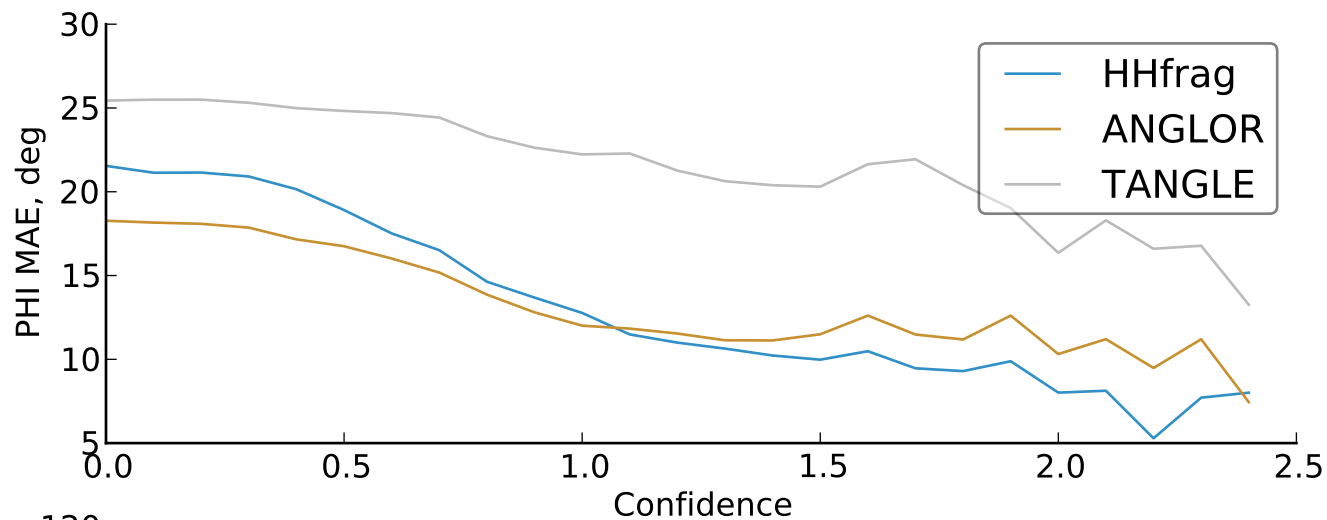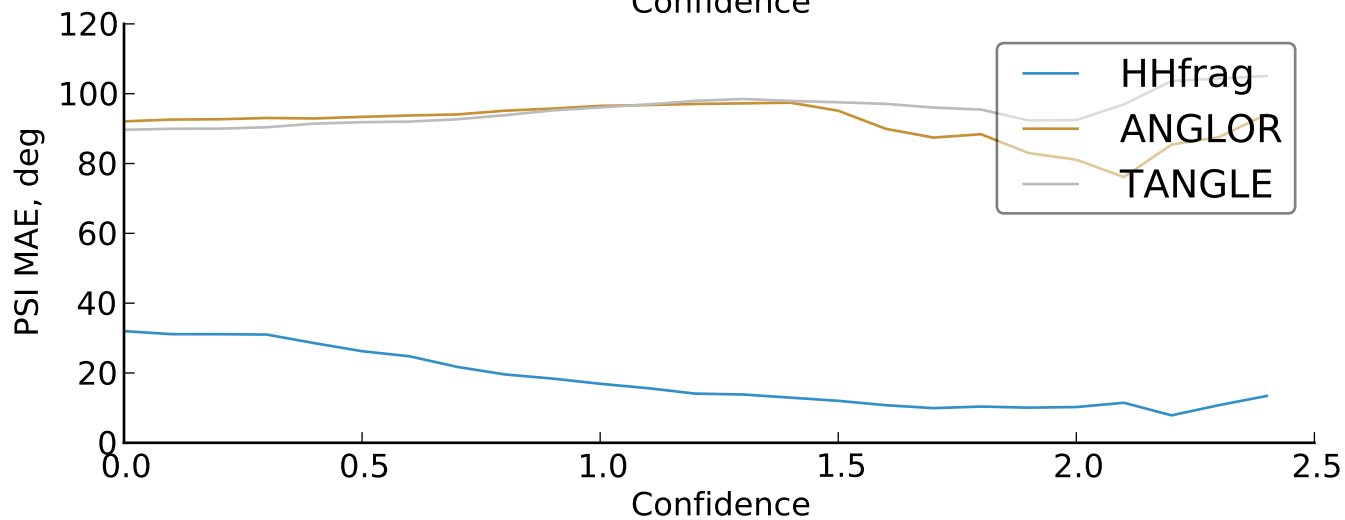

Supplement: Benchmark S1 — Local centroid precision for each target in the benchmark set and a breakdown of the torsion angle prediction performance by residue type and secondary structure. (ZIP) [file pone.0076512.s001.zip › MAE/GLU.pdf]

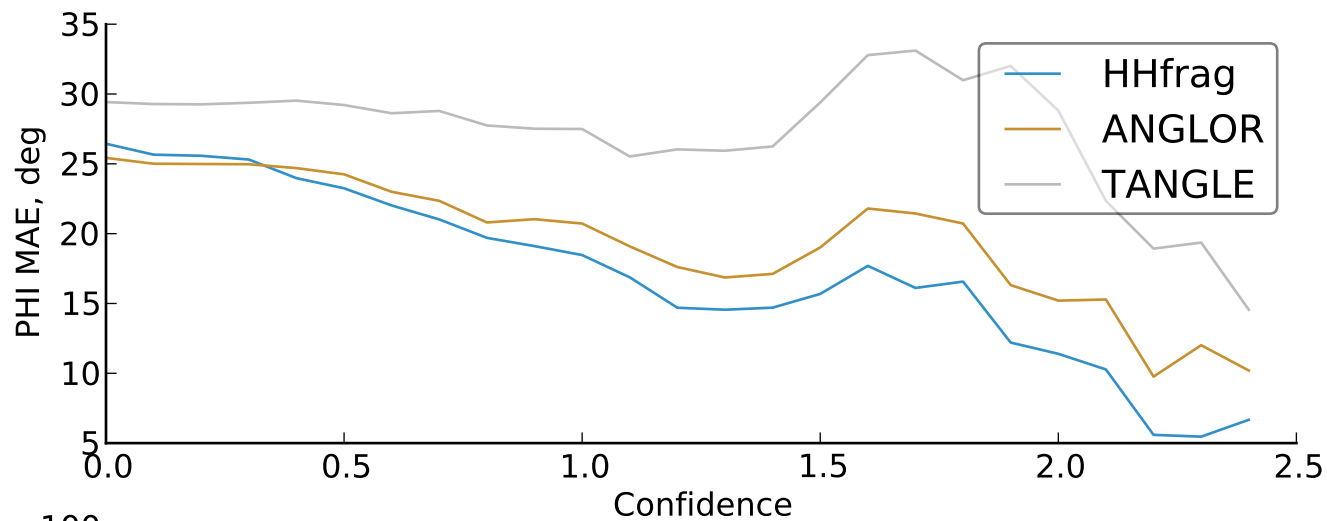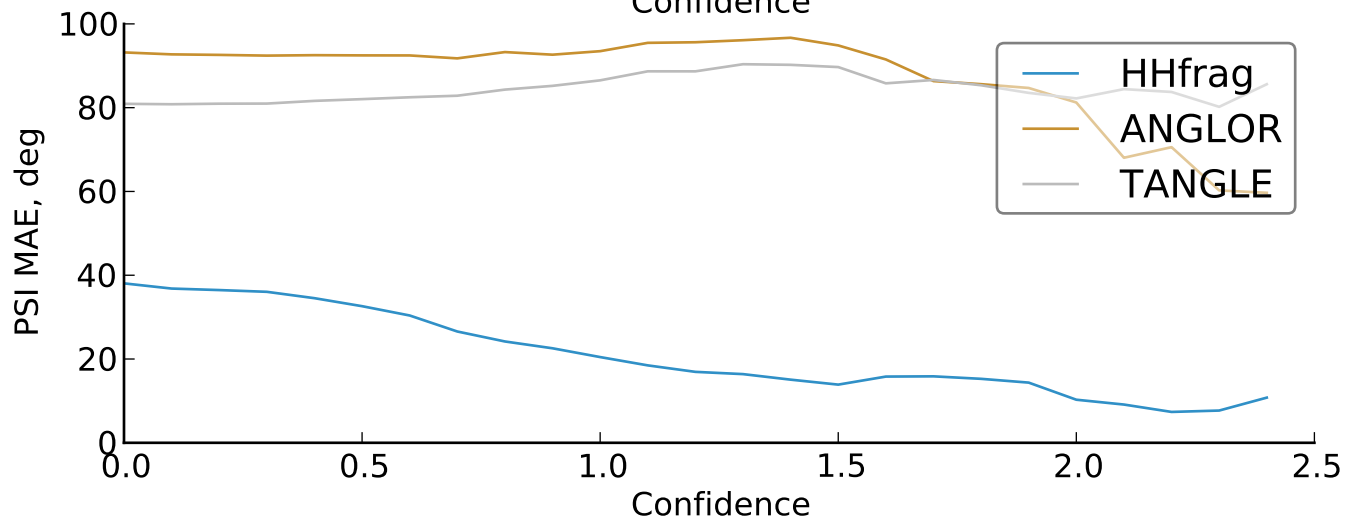

Supplement: Benchmark S1 — Local centroid precision for each target in the benchmark set and a breakdown of the torsion angle prediction performance by residue type and secondary structure. (ZIP) [file pone.0076512.s001.zip › MAE/ASP.pdf]

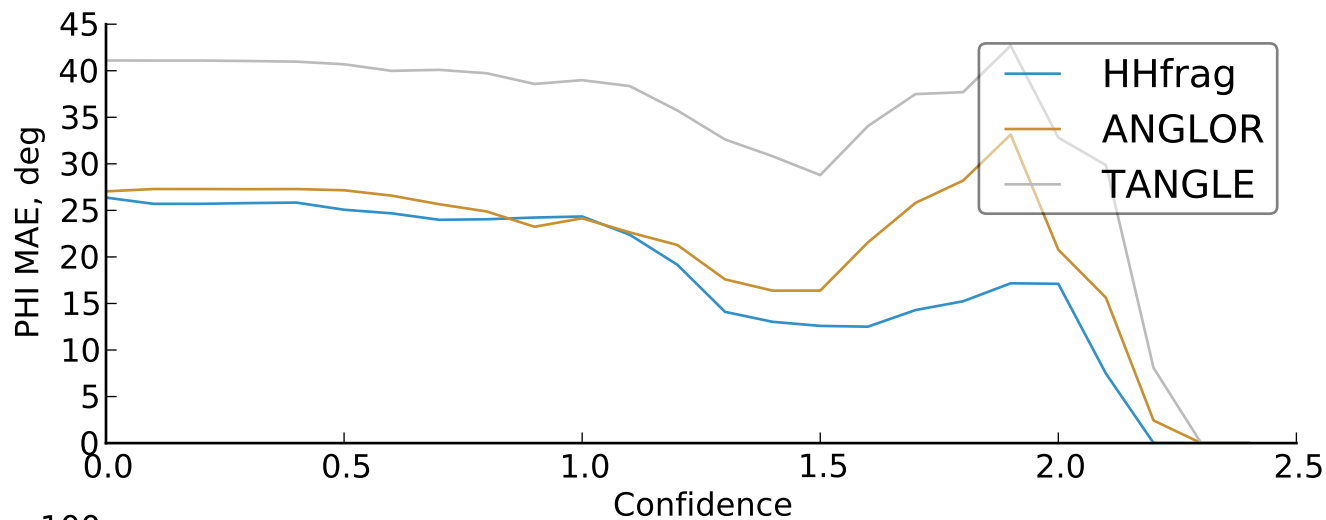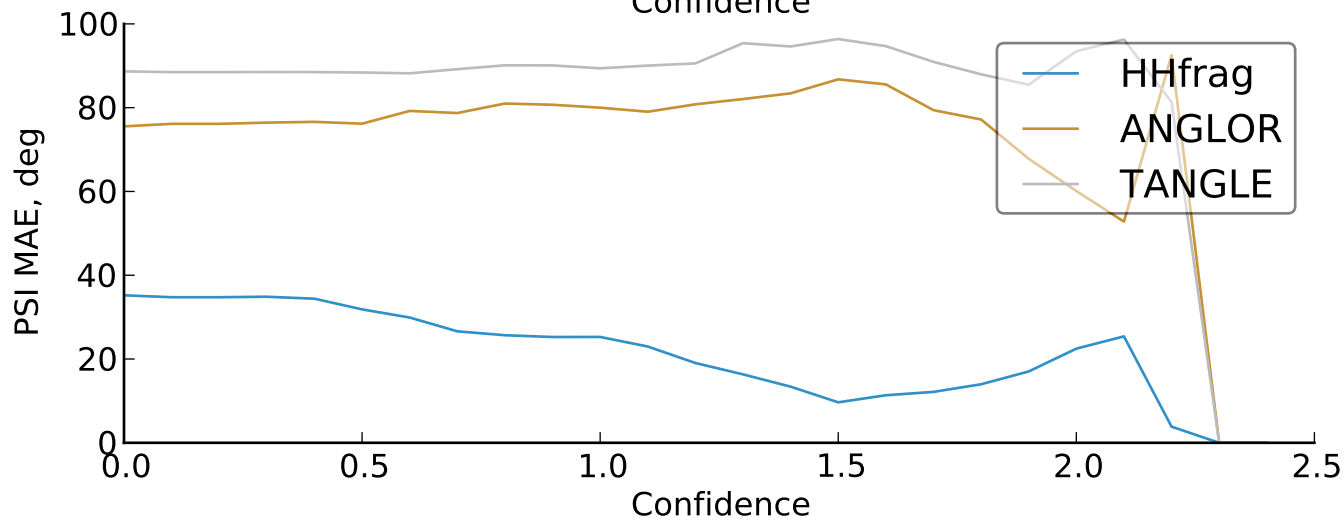

Supplement: Benchmark S1 — Local centroid precision for each target in the benchmark set and a breakdown of the torsion angle prediction performance by residue type and secondary structure. (ZIP) [file pone.0076512.s001.zip › MAE/CYS.pdf]

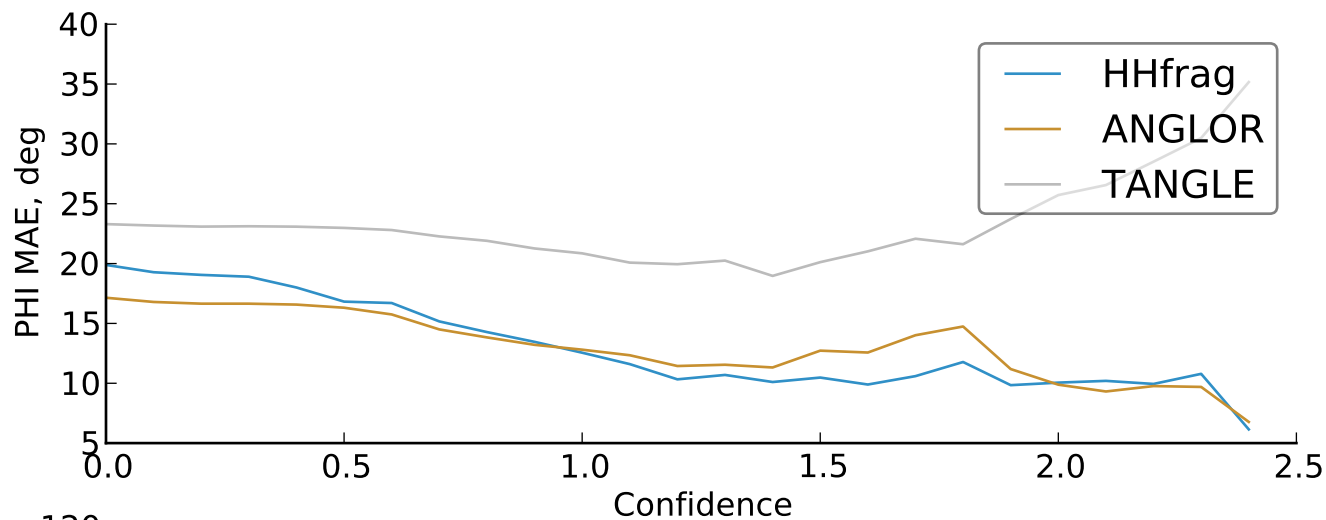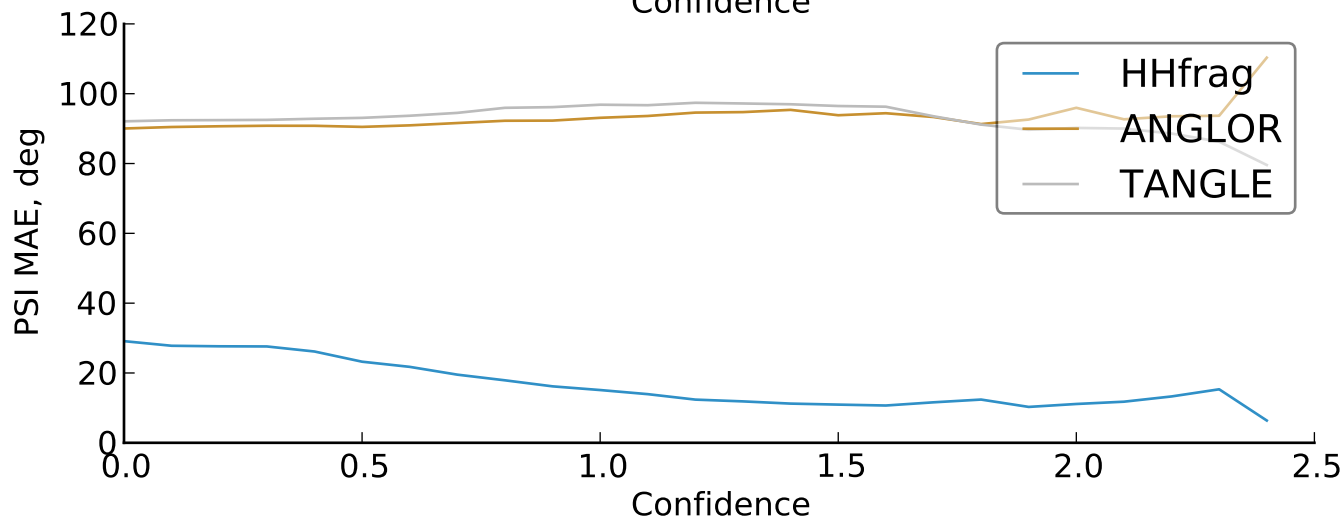

Supplement: Benchmark S1 — Local centroid precision for each target in the benchmark set and a breakdown of the torsion angle prediction performance by residue type and secondary structure. (ZIP) [file pone.0076512.s001.zip › MAE/ALA.pdf]

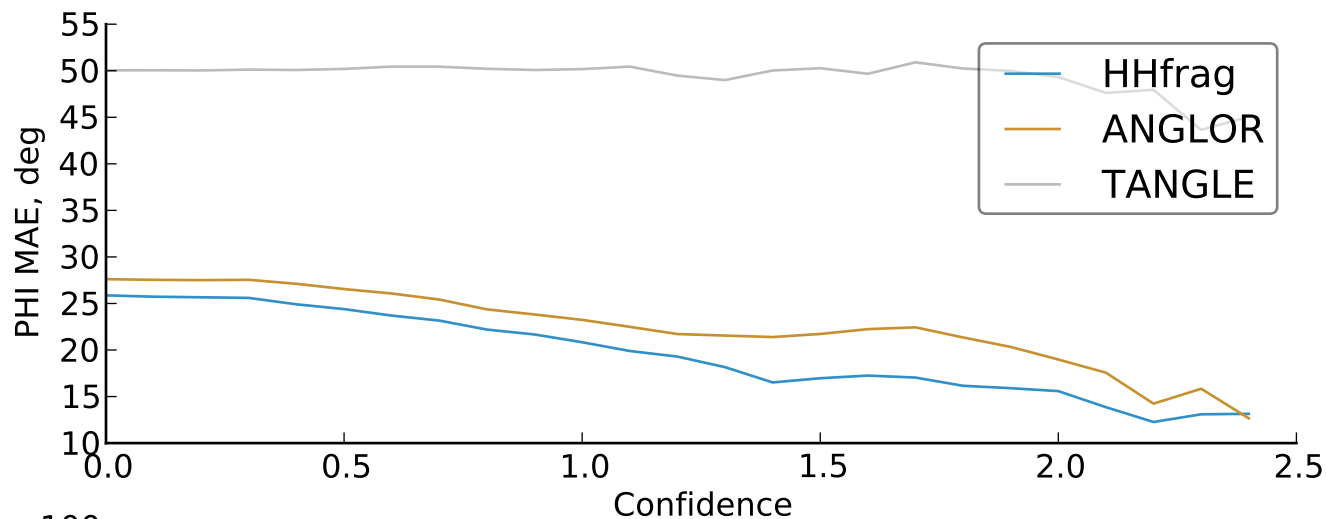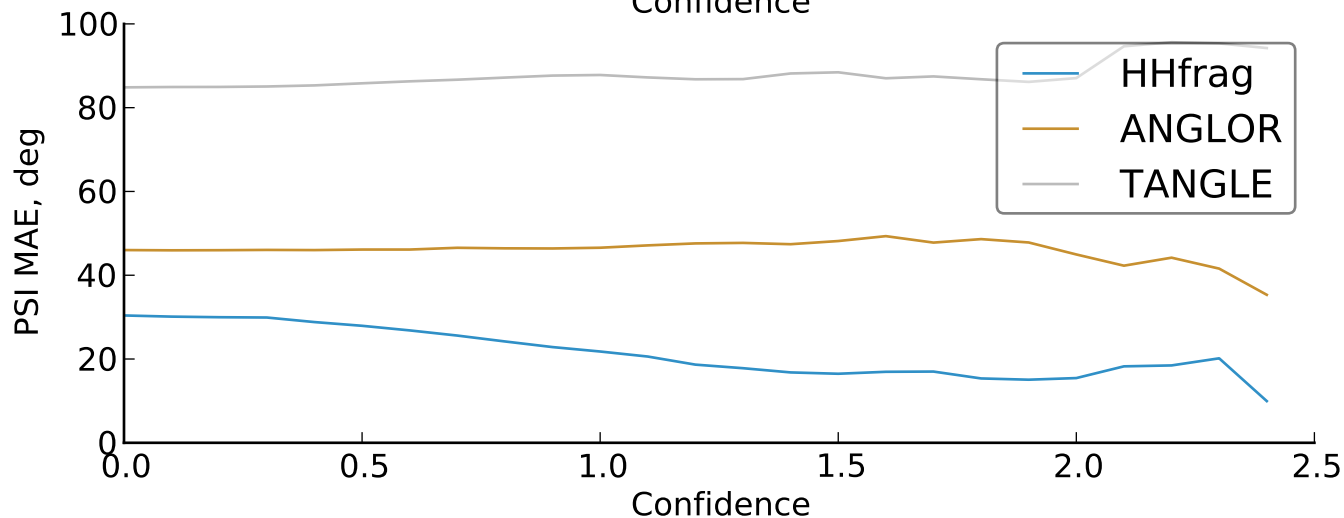

Supplement: Benchmark S1 — Local centroid precision for each target in the benchmark set and a breakdown of the torsion angle prediction performance by residue type and secondary structure. (ZIP) [file pone.0076512.s001.zip › MAE/Strand.pdf]

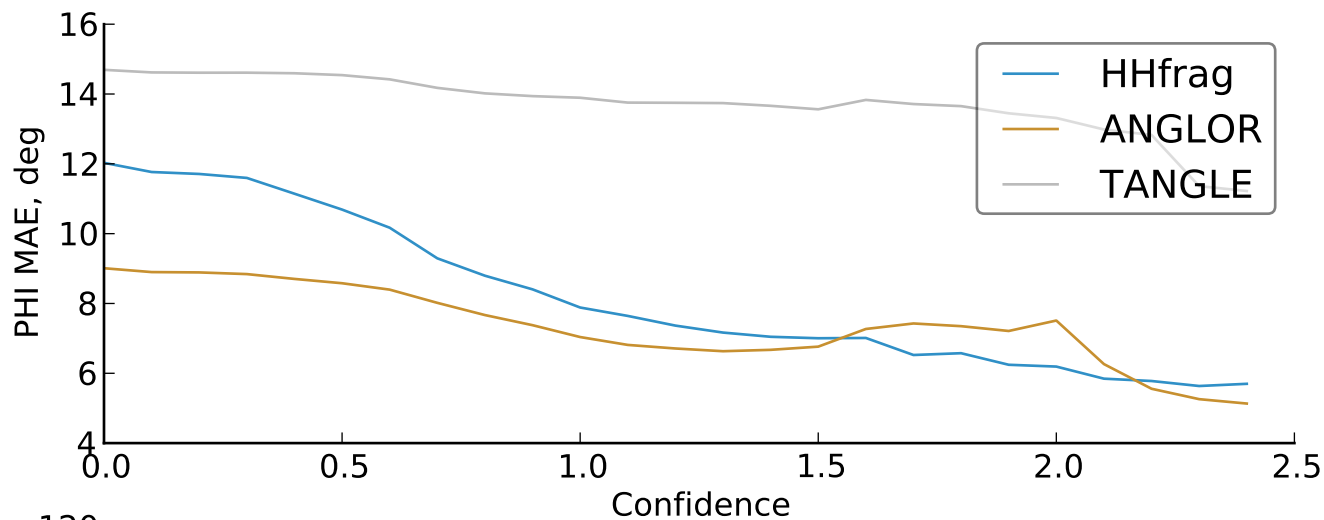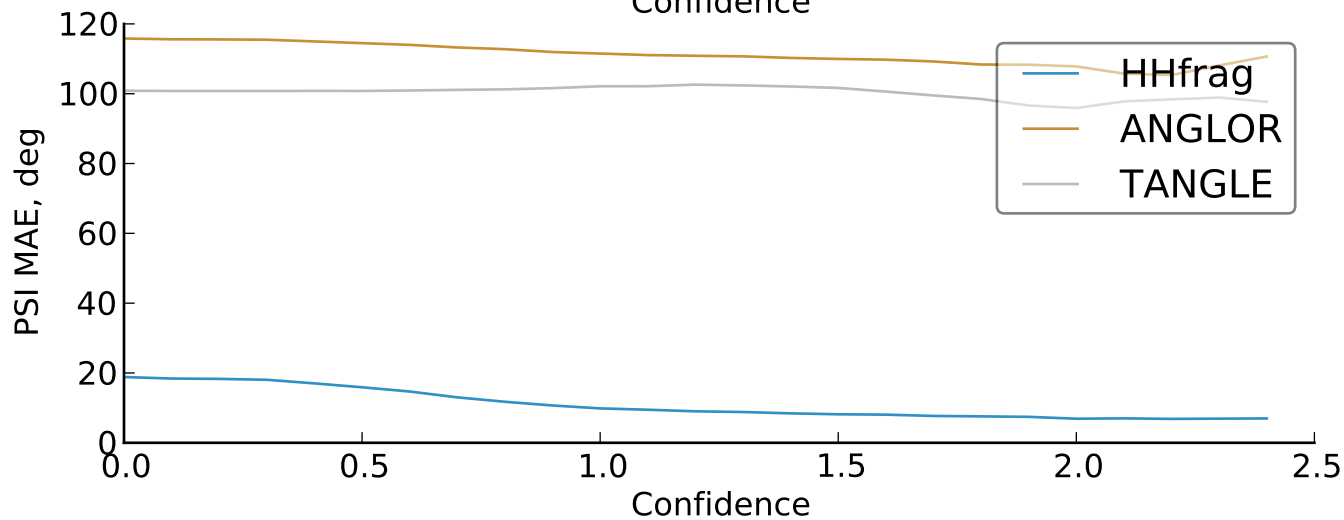

Supplement: Benchmark S1 — Local centroid precision for each target in the benchmark set and a breakdown of the torsion angle prediction performance by residue type and secondary structure. (ZIP) [file pone.0076512.s001.zip › MAE/Helix.pdf]

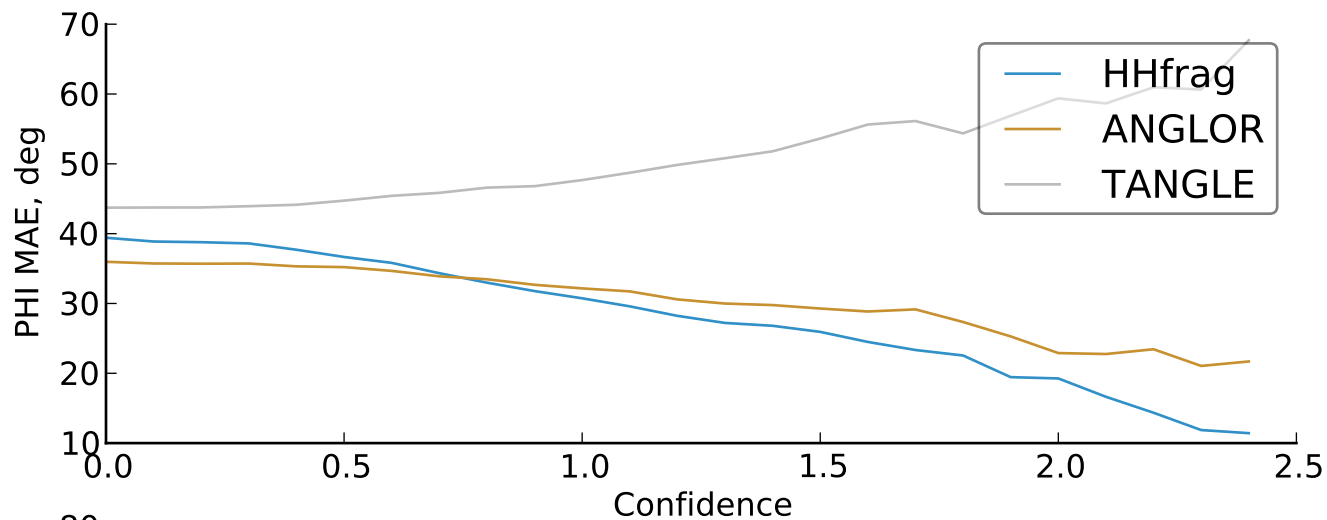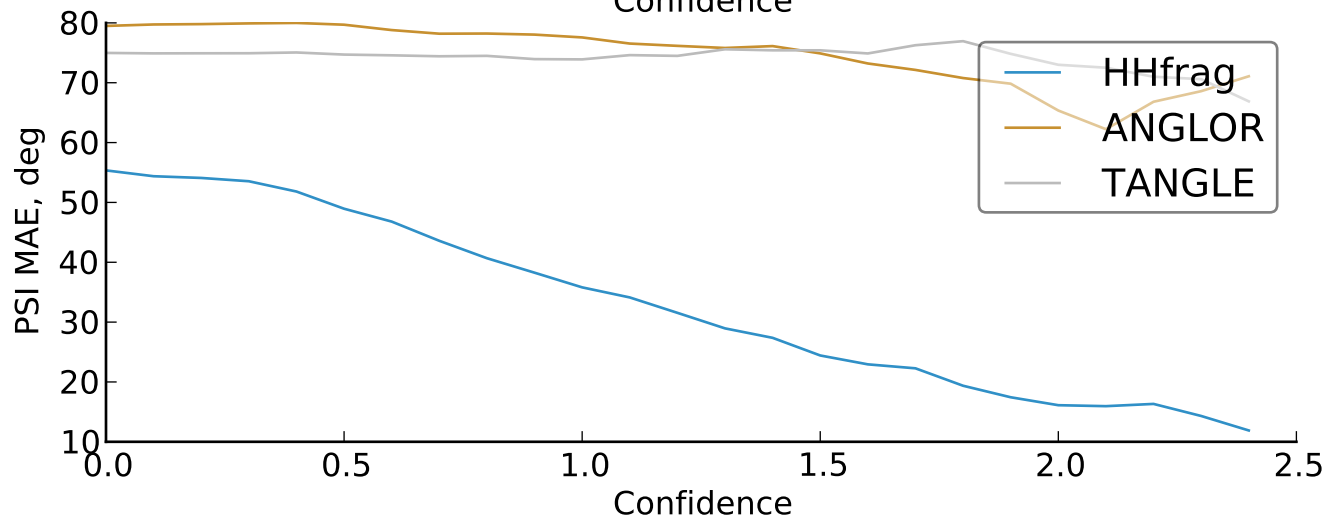

Supplement: Benchmark S1 — Local centroid precision for each target in the benchmark set and a breakdown of the torsion angle prediction performance by residue type and secondary structure. (ZIP) [file pone.0076512.s001.zip › MAE/Coil.pdf]

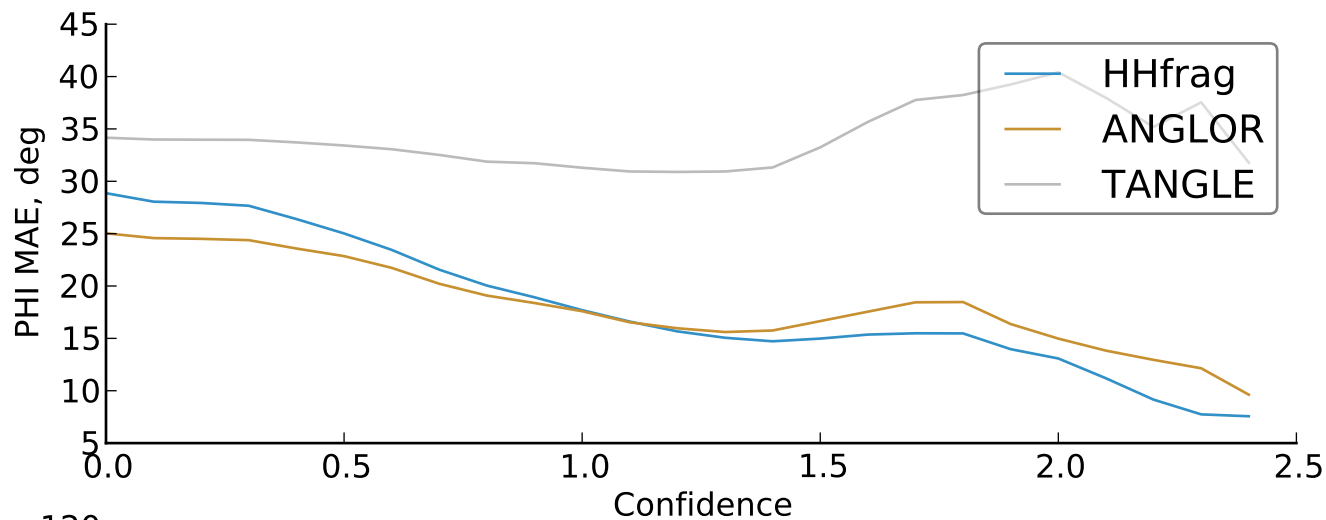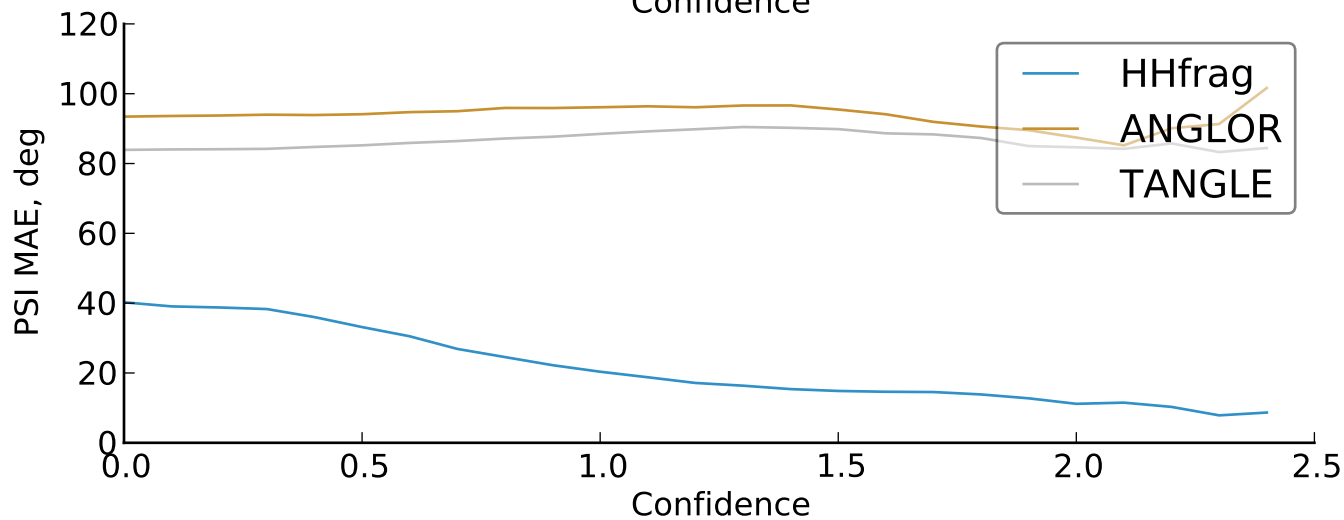

Supplement: Benchmark S1 — Local centroid precision for each target in the benchmark set and a breakdown of the torsion angle prediction performance by residue type and secondary structure. (ZIP) [file pone.0076512.s001.zip › MAE/Exposed.pdf]

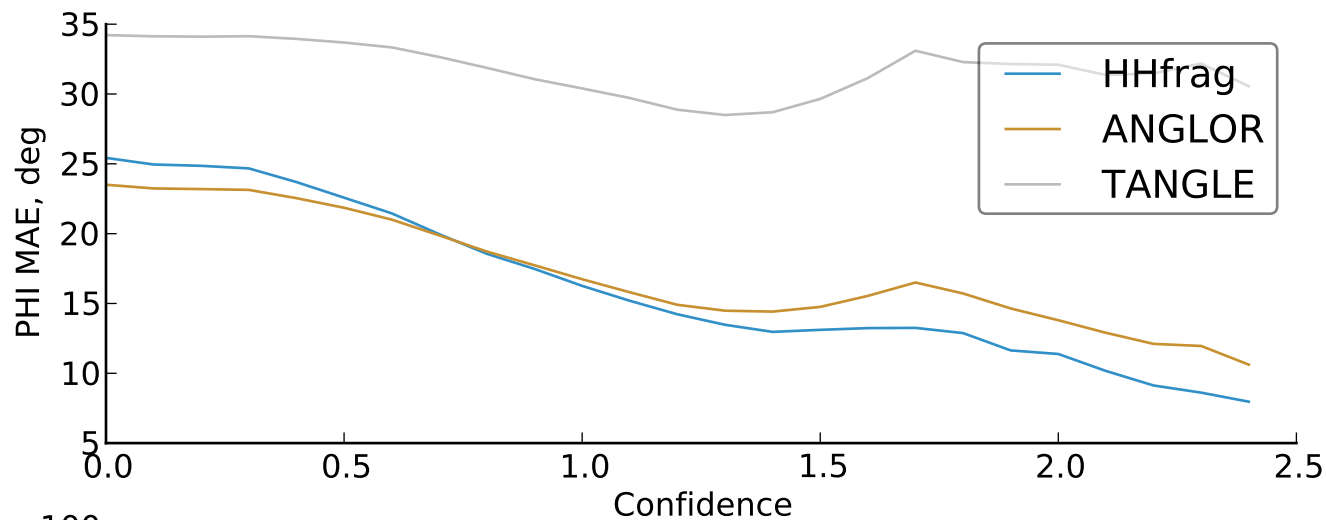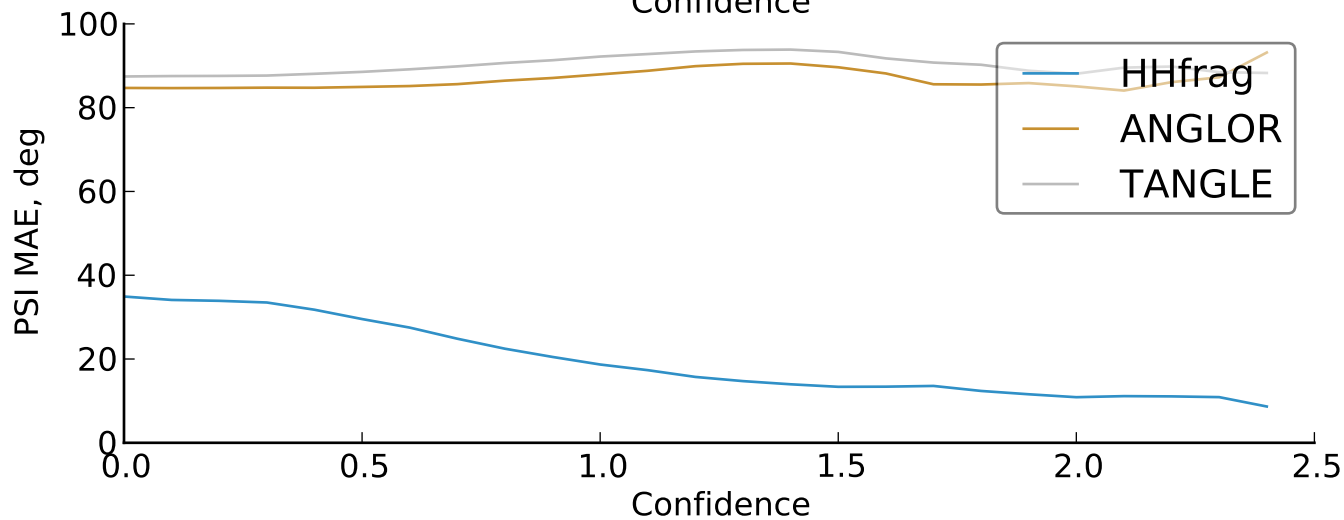

Supplement: Benchmark S1 — Local centroid precision for each target in the benchmark set and a breakdown of the torsion angle prediction performance by residue type and secondary structure. (ZIP) [file pone.0076512.s001.zip › MAE/Buried.pdf]

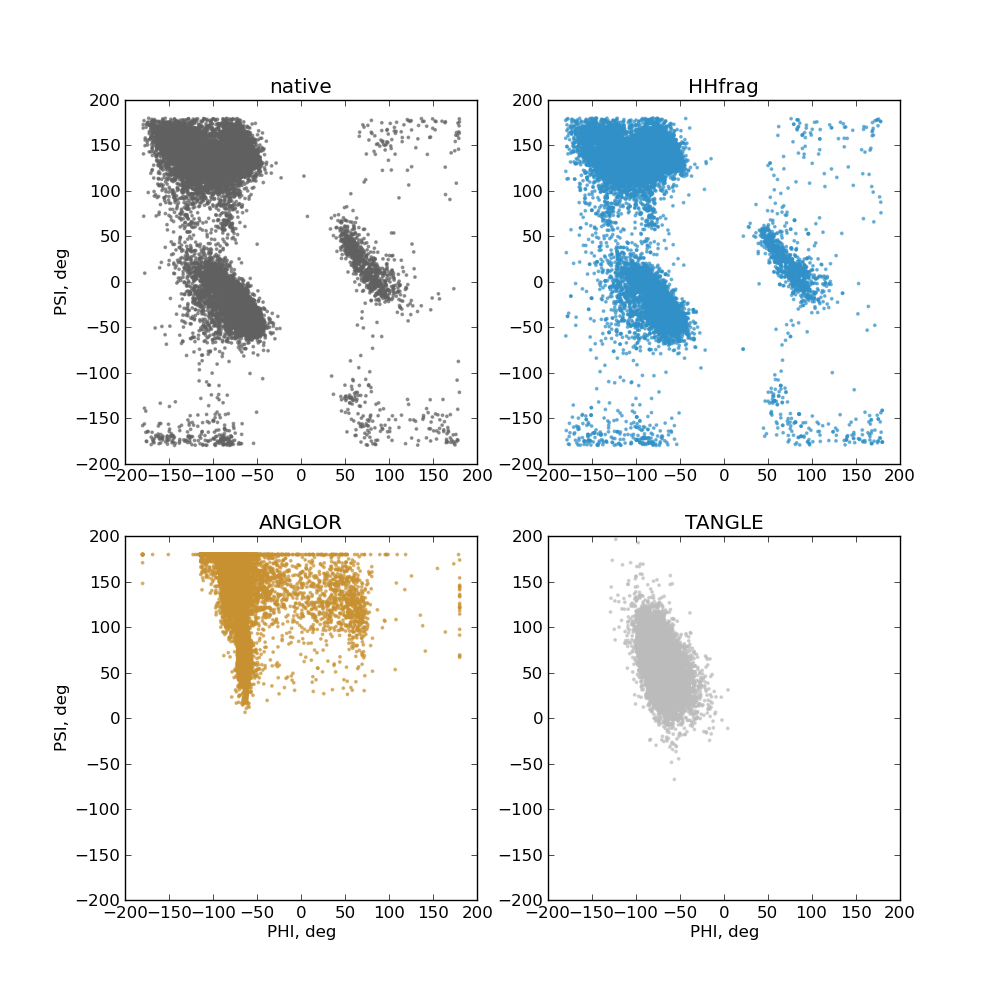

Supplement: Benchmark S1 — Local centroid precision for each target in the benchmark set and a breakdown of the torsion angle prediction performance by residue type and secondary structure. (ZIP) [file pone.0076512.s001.zip › Ramachandran/Ramachandran.png]
