# Supplementary figures and images for: Confidence-Guided Local Structure Prediction with HHfrag (part 1 of 2)
Source: PLoS One. 2013 Oct 16;8(10):e76512. doi: 10.1371/journal.pone.0076512 (PMC3797814; doi:10.1371/journal.pone.0076512)

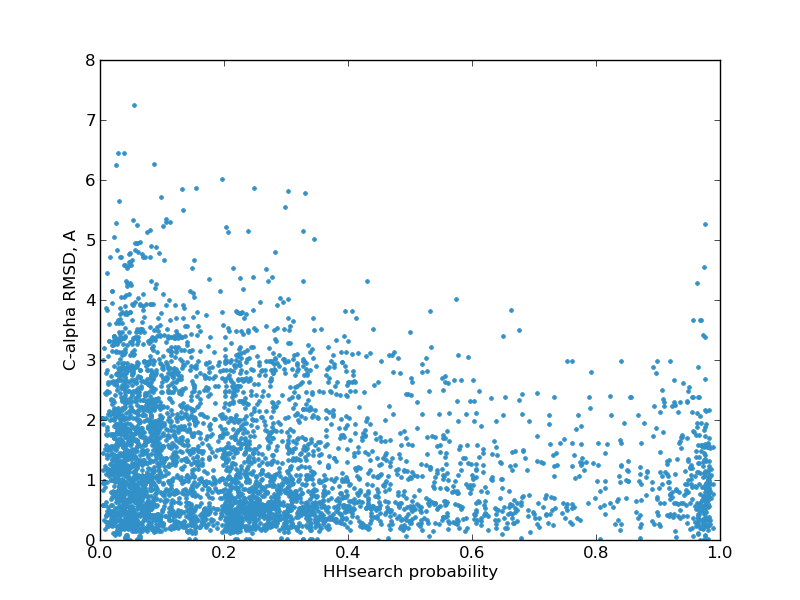

Supplement: Benchmark S1 — Local centroid precision for each target in the benchmark set and a breakdown of the torsion angle prediction performance by residue type and secondary structure. (ZIP) [file pone.0076512.s001.zip › Correlation/HHsearch.png]

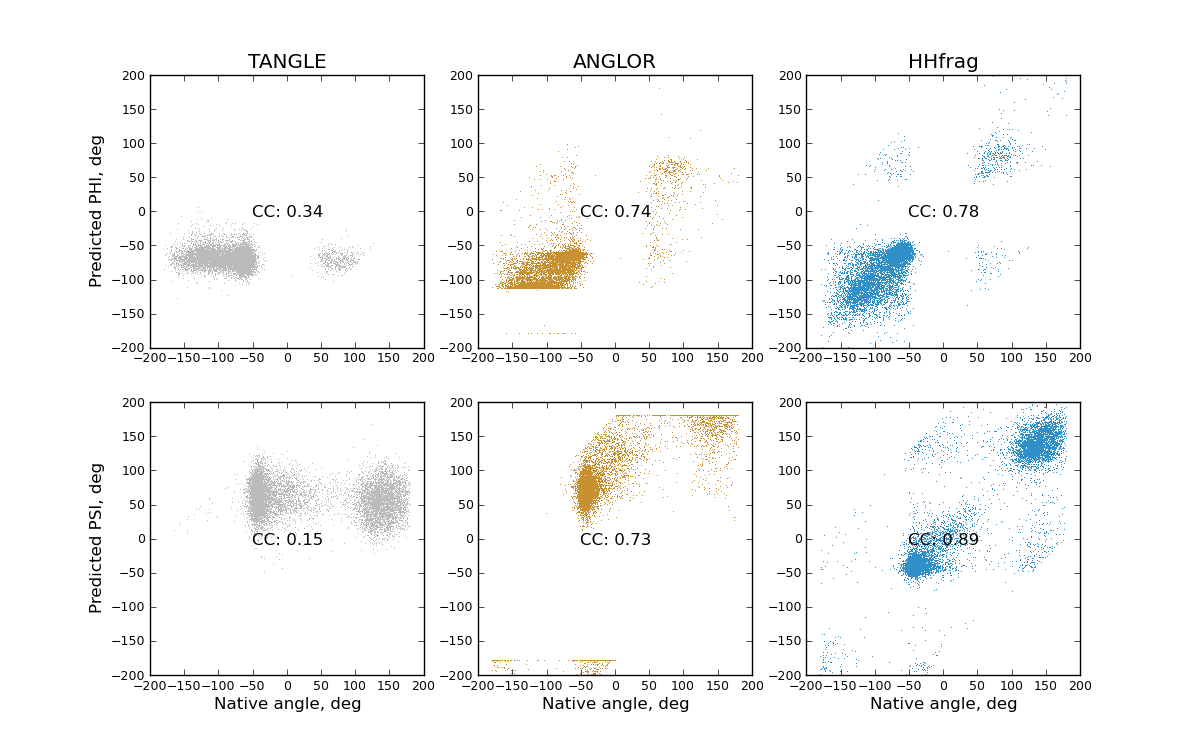

Supplement: Benchmark S1 — Local centroid precision for each target in the benchmark set and a breakdown of the torsion angle prediction performance by residue type and secondary structure. (ZIP) [file pone.0076512.s001.zip › Correlation/Angles.png]

3nz1A

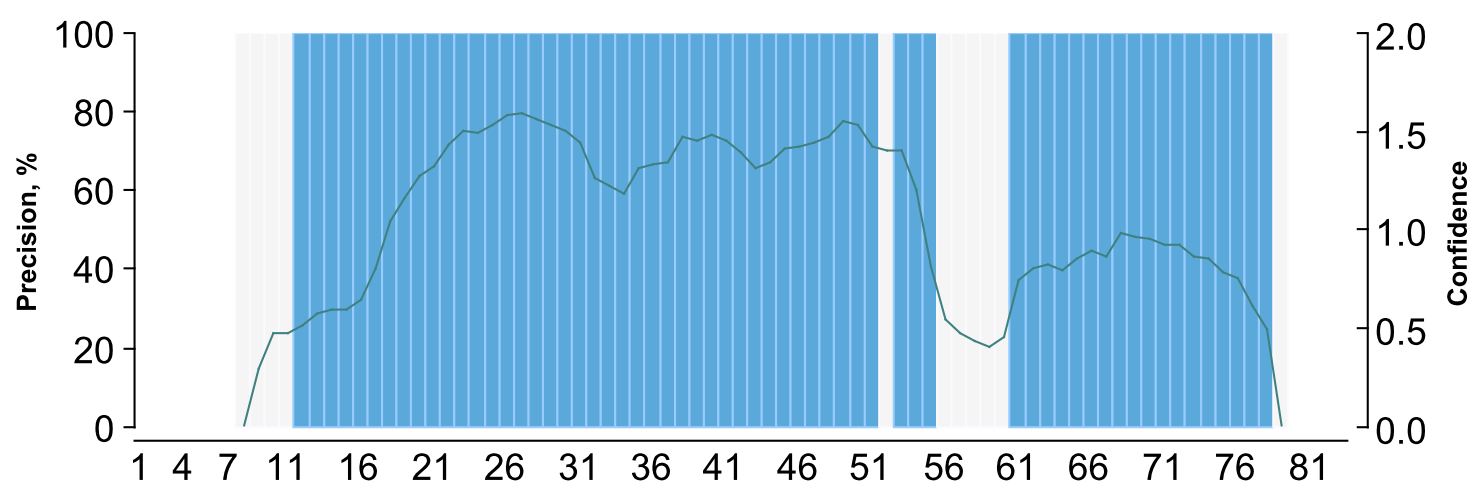

Supplement: Benchmark S1 — Local centroid precision for each target in the benchmark set and a breakdown of the torsion angle prediction performance by residue type and secondary structure. (ZIP) [file pone.0076512.s001.zip › Filtering/3nzlA.pdf]

# 3nyiA

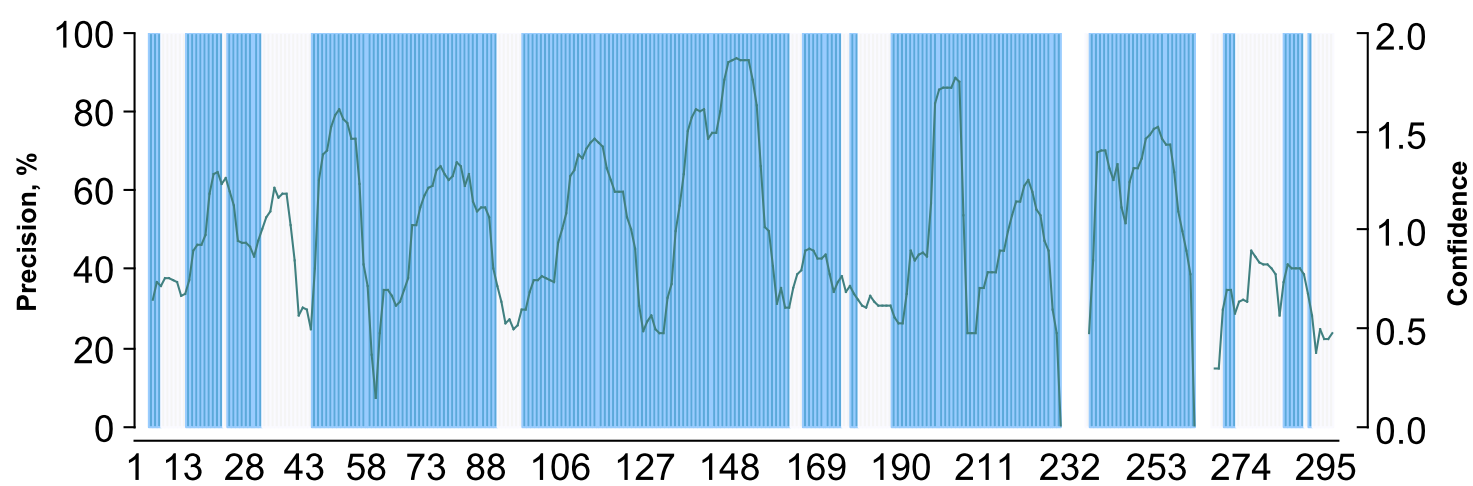

Supplement: Benchmark S1 — Local centroid precision for each target in the benchmark set and a breakdown of the torsion angle prediction performance by residue type and secondary structure. (ZIP) [file pone.0076512.s001.zip › Filtering/3nyiA.pdf]

3nywA

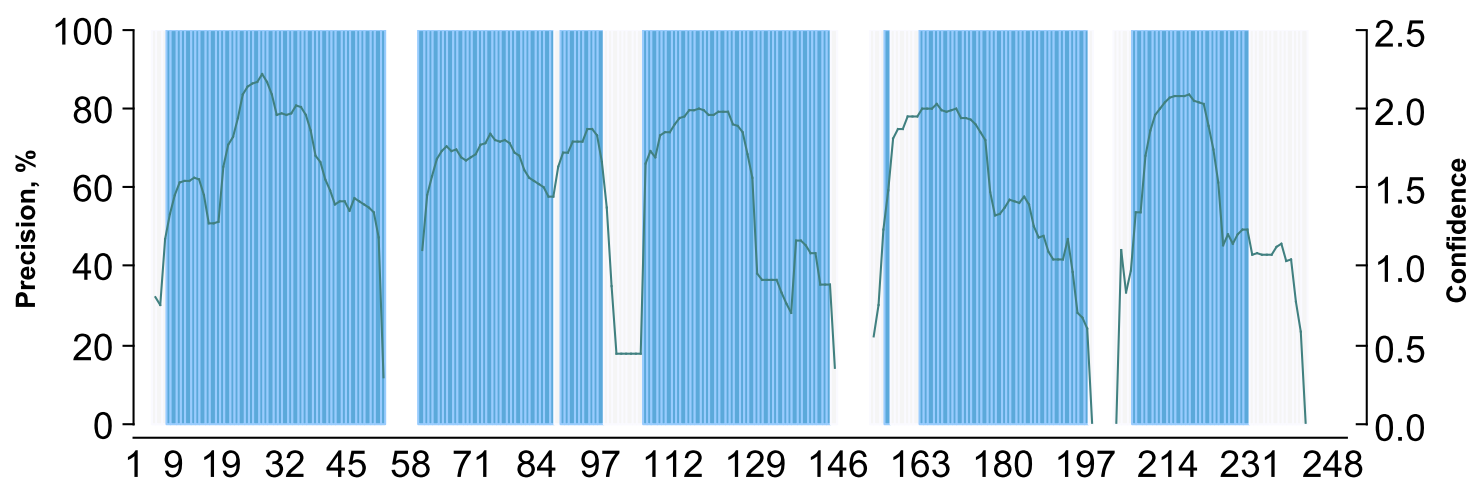

Supplement: Benchmark S1 — Local centroid precision for each target in the benchmark set and a breakdown of the torsion angle prediction performance by residue type and secondary structure. (ZIP) [file pone.0076512.s001.zip › Filtering/3nywA.pdf]

### 3nxA

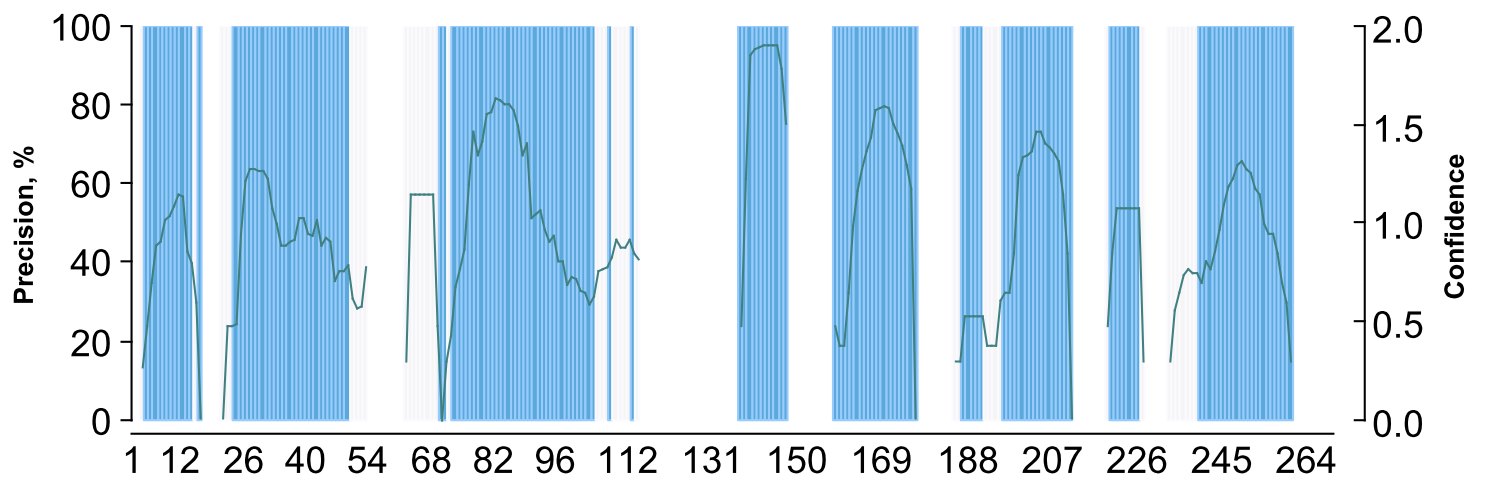

Supplement: Benchmark S1 — Local centroid precision for each target in the benchmark set and a breakdown of the torsion angle prediction performance by residue type and secondary structure. (ZIP) [file pone.0076512.s001.zip › Filtering/3nxhA.pdf]

3p1tA

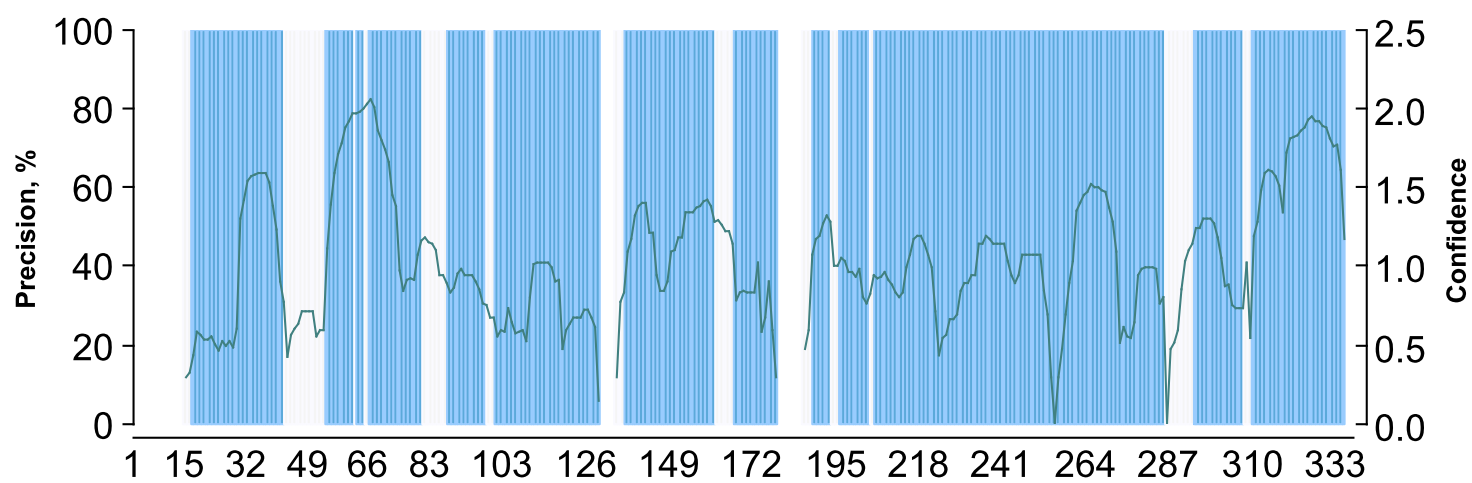

Supplement: Benchmark S1 — Local centroid precision for each target in the benchmark set and a breakdown of the torsion angle prediction performance by residue type and secondary structure. (ZIP) [file pone.0076512.s001.zip › Filtering/3p1tA.pdf]

3n1uA

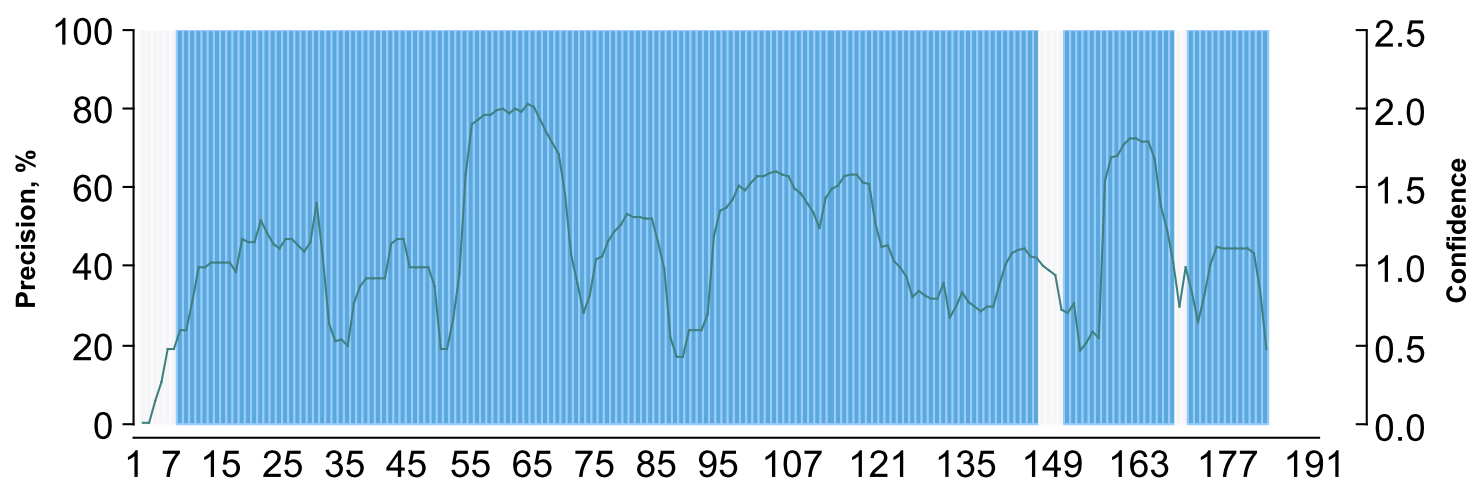

Supplement: Benchmark S1 — Local centroid precision for each target in the benchmark set and a breakdown of the torsion angle prediction performance by residue type and secondary structure. (ZIP) [file pone.0076512.s001.zip › Filtering/3n1uA.pdf]

3n53A

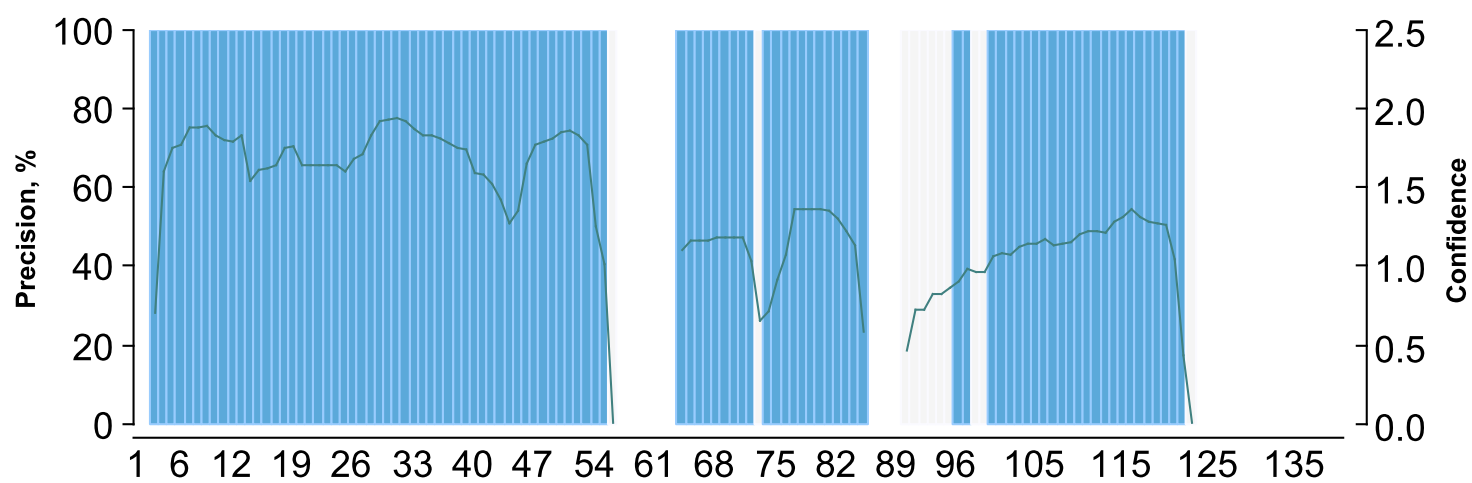

Supplement: Benchmark S1 — Local centroid precision for each target in the benchmark set and a breakdown of the torsion angle prediction performance by residue type and secondary structure. (ZIP) [file pone.0076512.s001.zip › Filtering/3n53A.pdf]

3nwzA

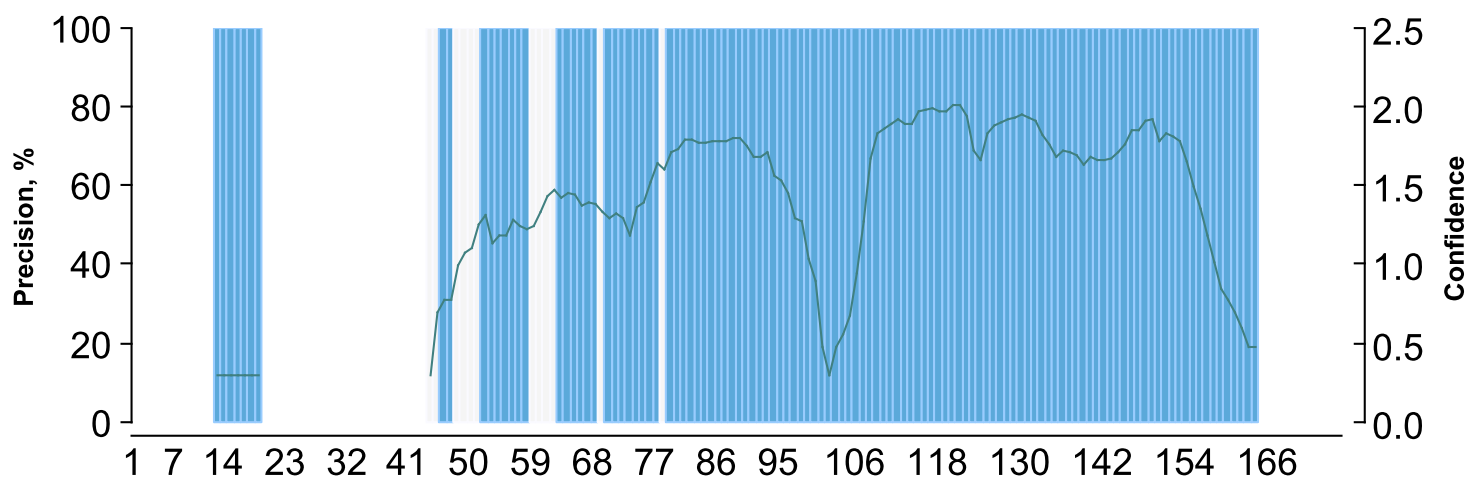

Supplement: Benchmark S1 — Local centroid precision for each target in the benchmark set and a breakdown of the torsion angle prediction performance by residue type and secondary structure. (ZIP) [file pone.0076512.s001.zip › Filtering/3nwzA.pdf]

2kytA

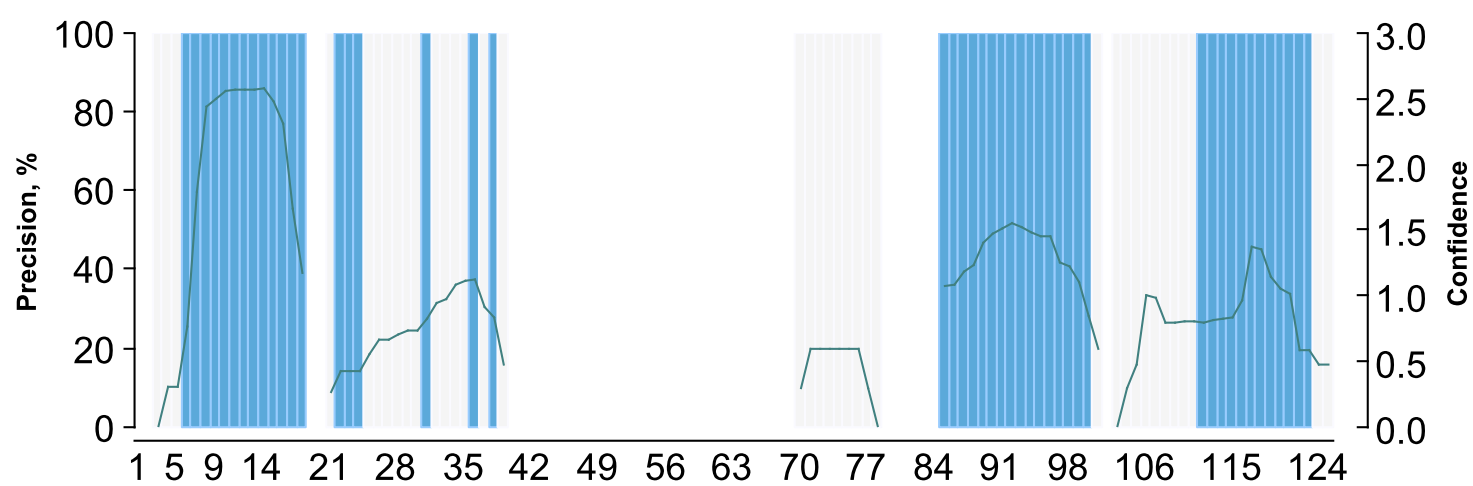

Supplement: Benchmark S1 — Local centroid precision for each target in the benchmark set and a breakdown of the torsion angle prediction performance by residue type and secondary structure. (ZIP) [file pone.0076512.s001.zip › Filtering/2kytA.pdf]

2xgfA

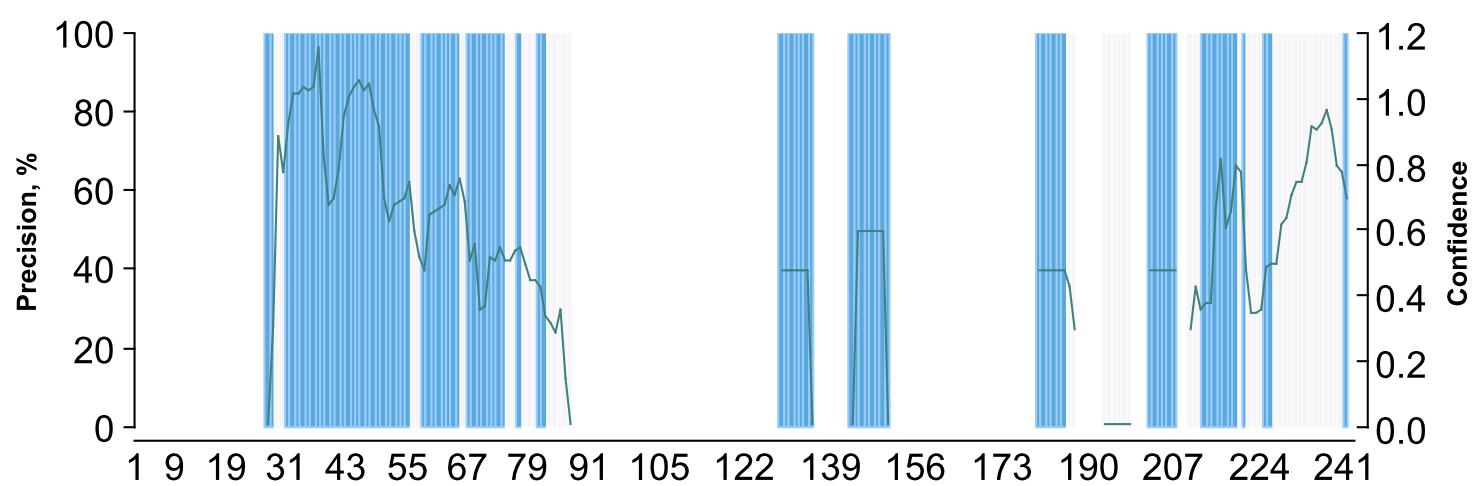

Supplement: Benchmark S1 — Local centroid precision for each target in the benchmark set and a breakdown of the torsion angle prediction performance by residue type and secondary structure. (ZIP) [file pone.0076512.s001.zip › Filtering/2xgfA.pdf]

3nuwA

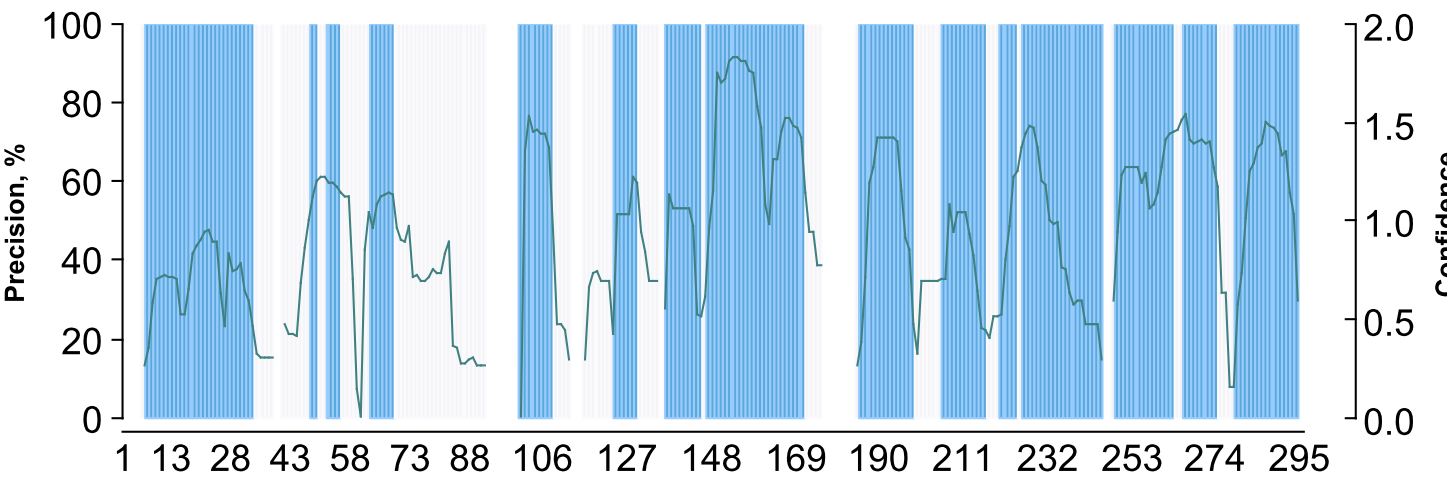

Supplement: Benchmark S1 — Local centroid precision for each target in the benchmark set and a breakdown of the torsion angle prediction performance by residue type and secondary structure. (ZIP) [file pone.0076512.s001.zip › Filtering/3nuwA.pdf]

# 3oqlA

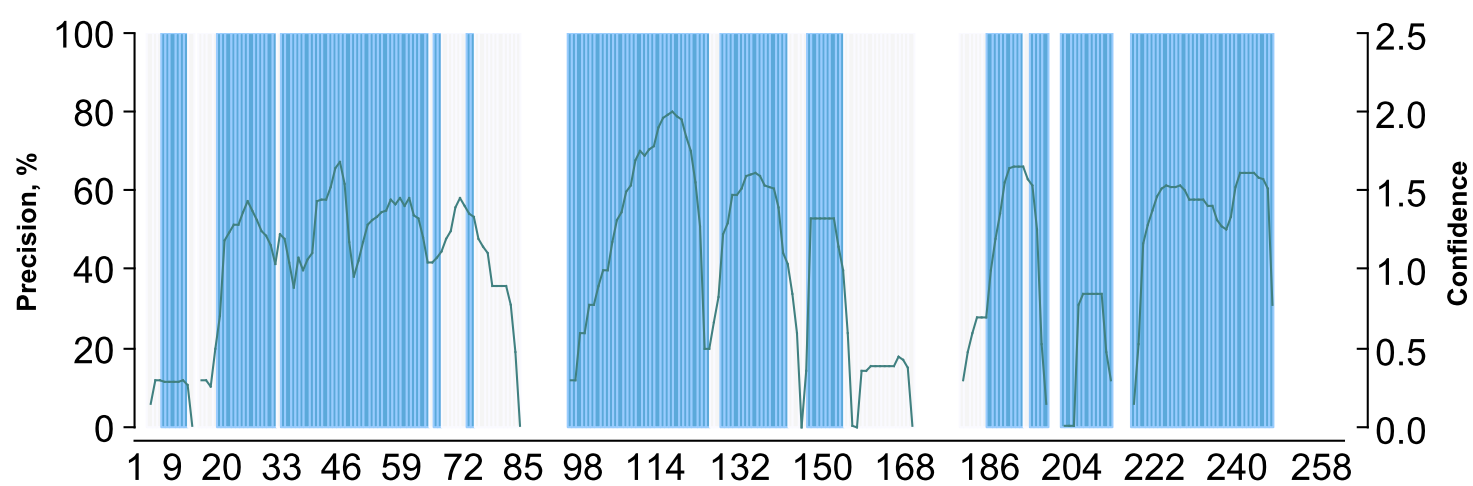

Supplement: Benchmark S1 — Local centroid precision for each target in the benchmark set and a breakdown of the torsion angle prediction performance by residue type and secondary structure. (ZIP) [file pone.0076512.s001.zip › Filtering/3oqlA.pdf]

# 3o11A

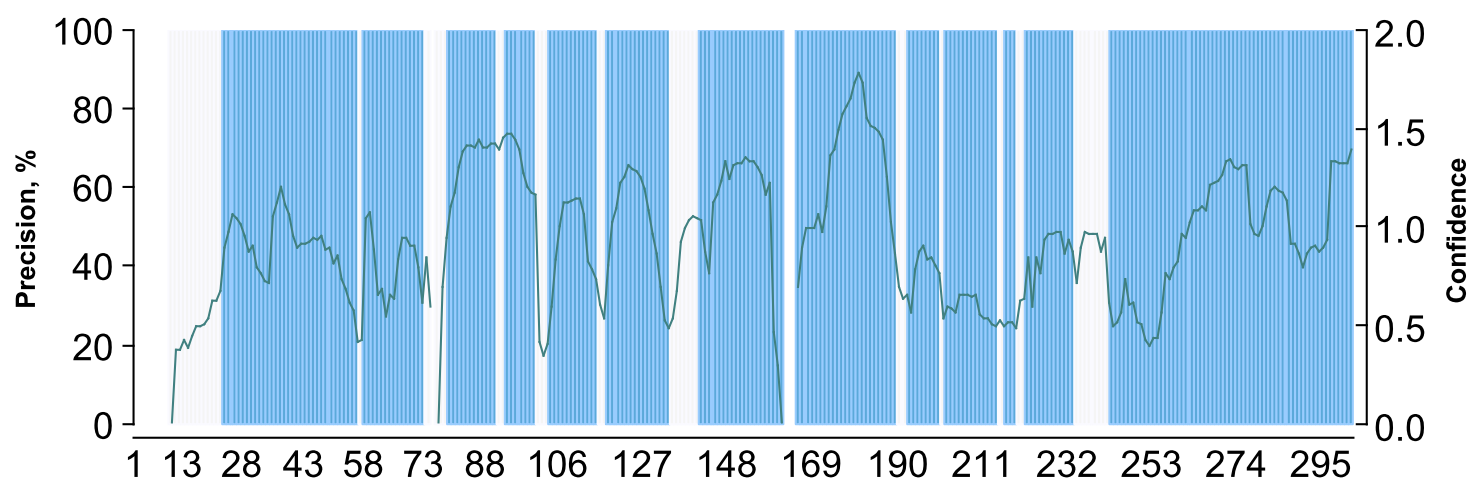

Supplement: Benchmark S1 — Local centroid precision for each target in the benchmark set and a breakdown of the torsion angle prediction performance by residue type and secondary structure. (ZIP) [file pone.0076512.s001.zip › Filtering/3o1lA.pdf]

# 3oruA

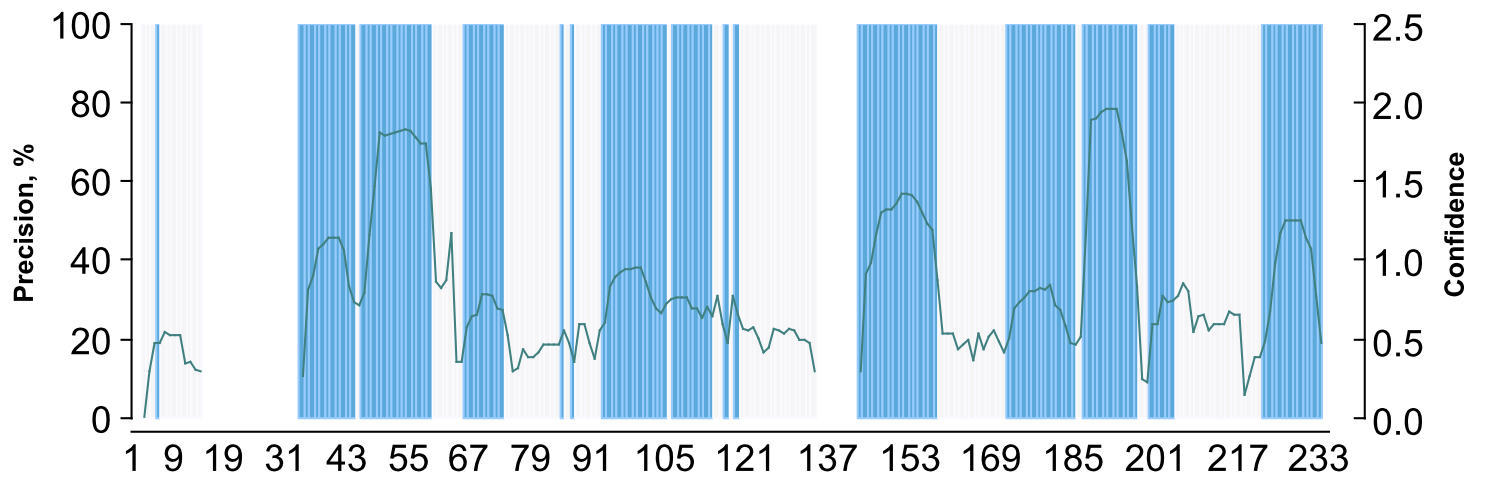

Supplement: Benchmark S1 — Local centroid precision for each target in the benchmark set and a breakdown of the torsion angle prediction performance by residue type and secondary structure. (ZIP) [file pone.0076512.s001.zip › Filtering/3oruA.pdf]

3nkhA

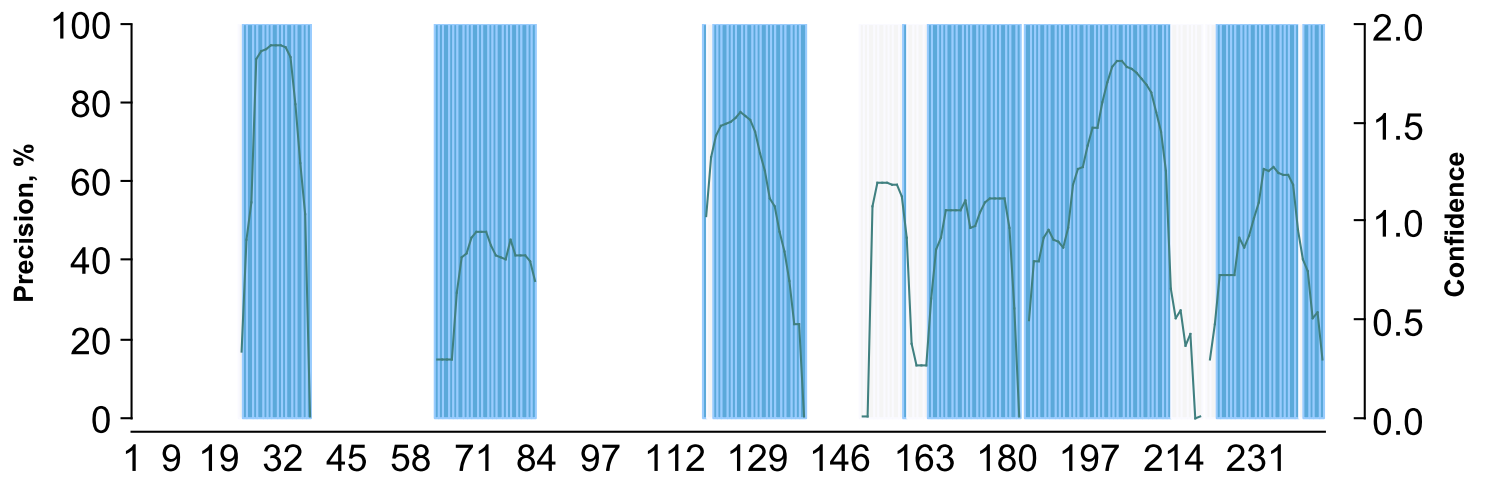

Supplement: Benchmark S1 — Local centroid precision for each target in the benchmark set and a breakdown of the torsion angle prediction performance by residue type and secondary structure. (ZIP) [file pone.0076512.s001.zip › Filtering/3nkhA.pdf]

# 3nklA

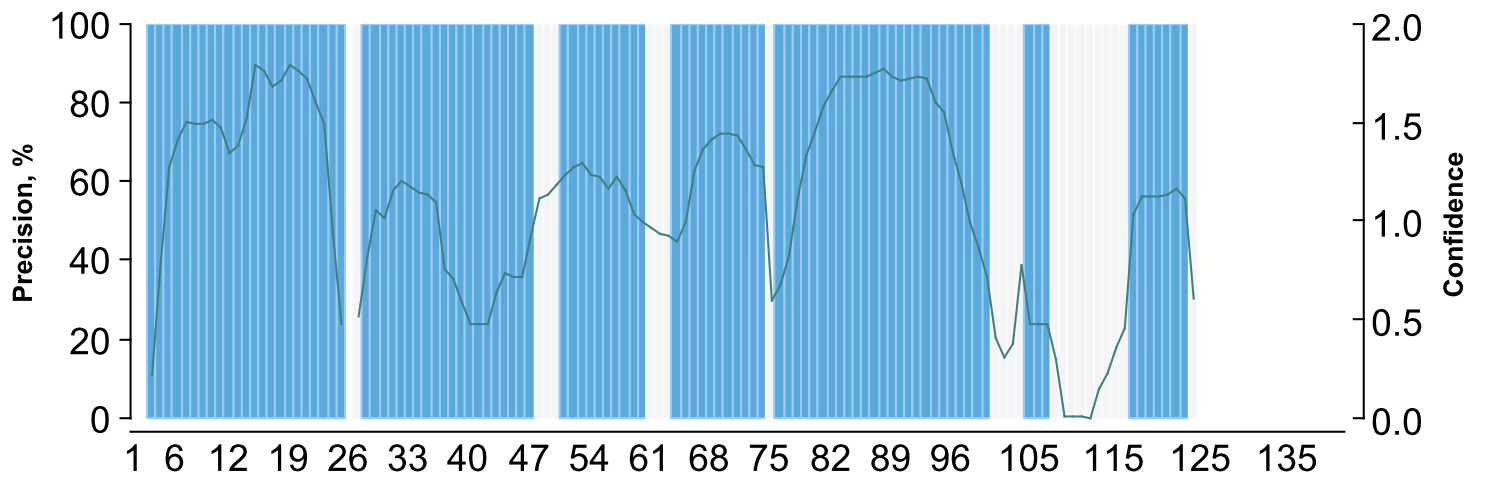

Supplement: Benchmark S1 — Local centroid precision for each target in the benchmark set and a breakdown of the torsion angle prediction performance by residue type and secondary structure. (ZIP) [file pone.0076512.s001.zip › Filtering/3nklA.pdf]

# 3nr8A

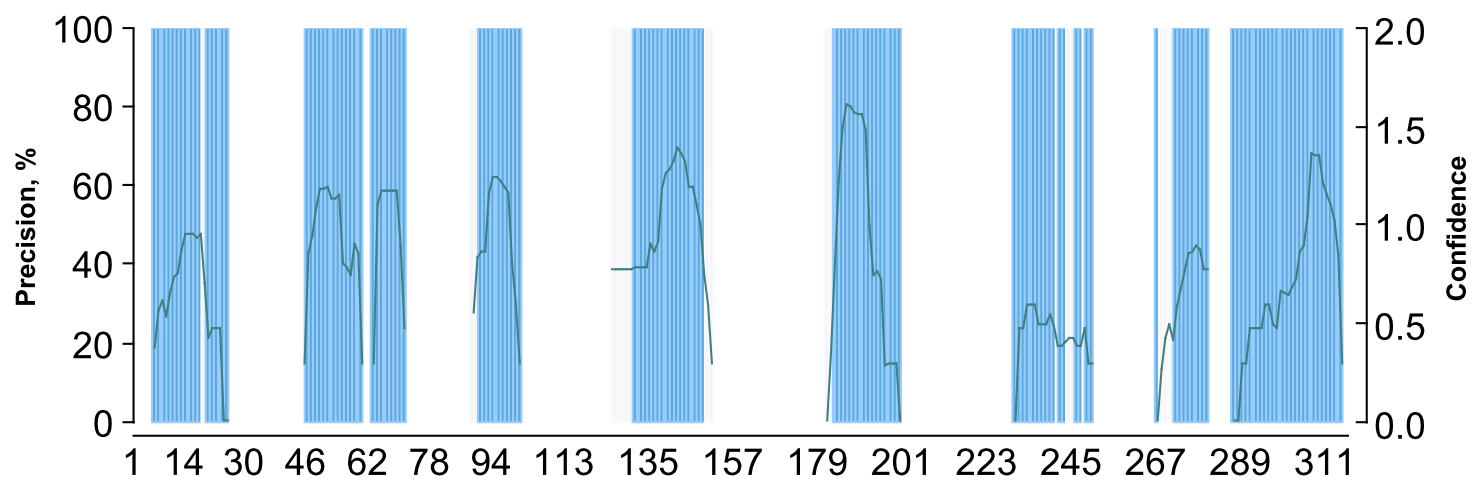

Supplement: Benchmark S1 — Local centroid precision for each target in the benchmark set and a breakdown of the torsion angle prediction performance by residue type and secondary structure. (ZIP) [file pone.0076512.s001.zip › Filtering/3nr8A.pdf]

3nrwA

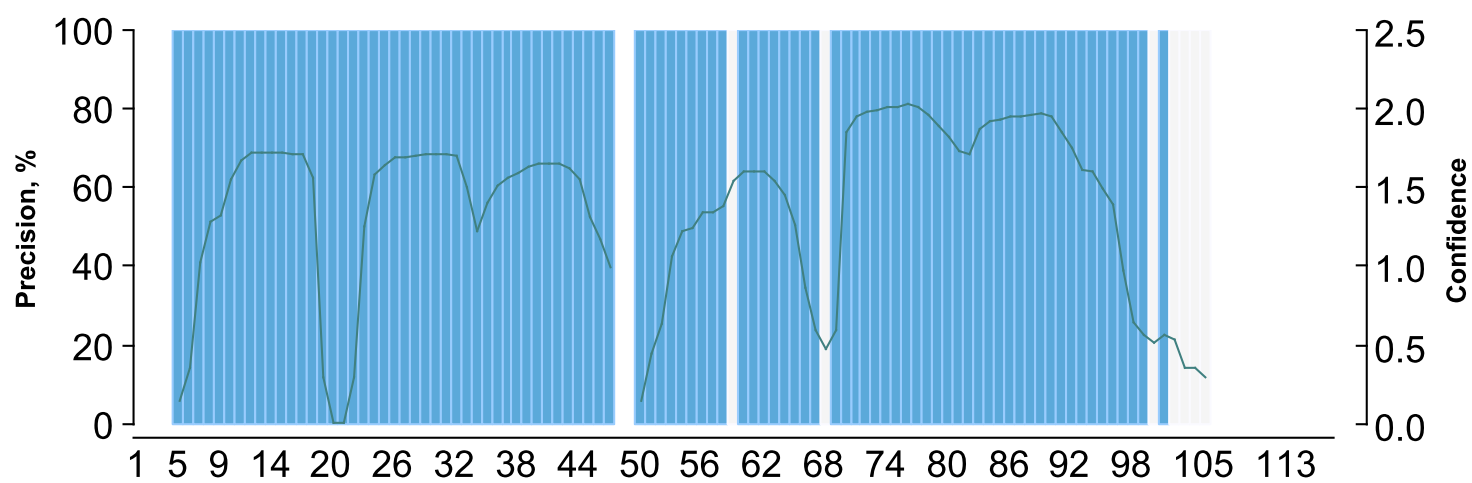

Supplement: Benchmark S1 — Local centroid precision for each target in the benchmark set and a breakdown of the torsion angle prediction performance by residue type and secondary structure. (ZIP) [file pone.0076512.s001.zip › Filtering/3nrwA.pdf]

### 3nrvA

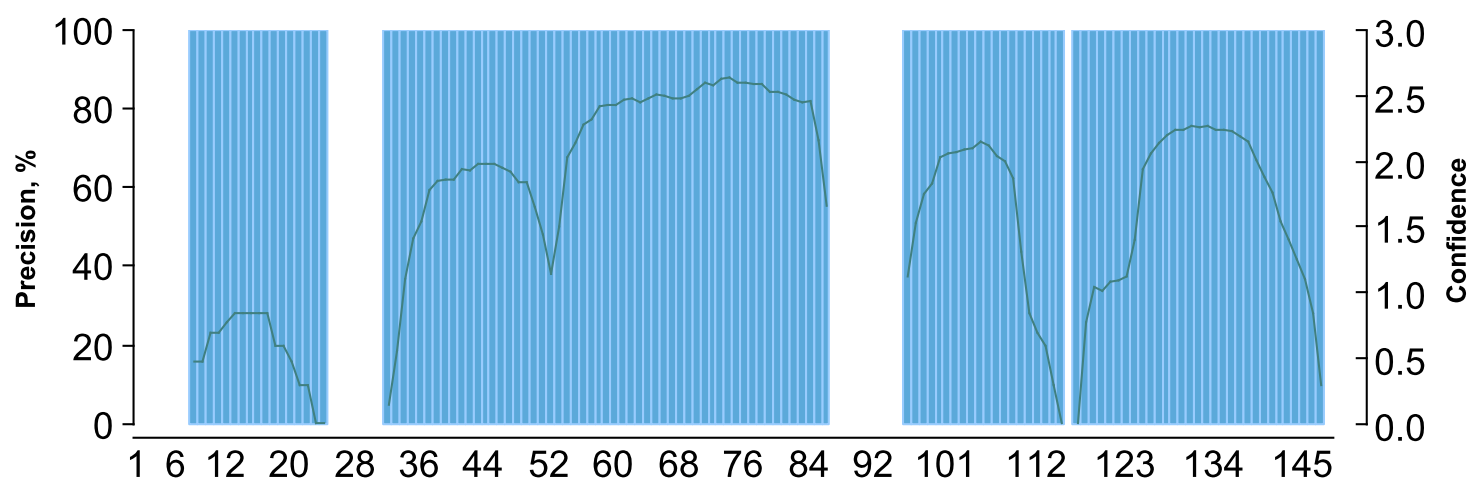

Supplement: Benchmark S1 — Local centroid precision for each target in the benchmark set and a breakdown of the torsion angle prediction performance by residue type and secondary structure. (ZIP) [file pone.0076512.s001.zip › Filtering/3nrvA.pdf]

# 3obiA

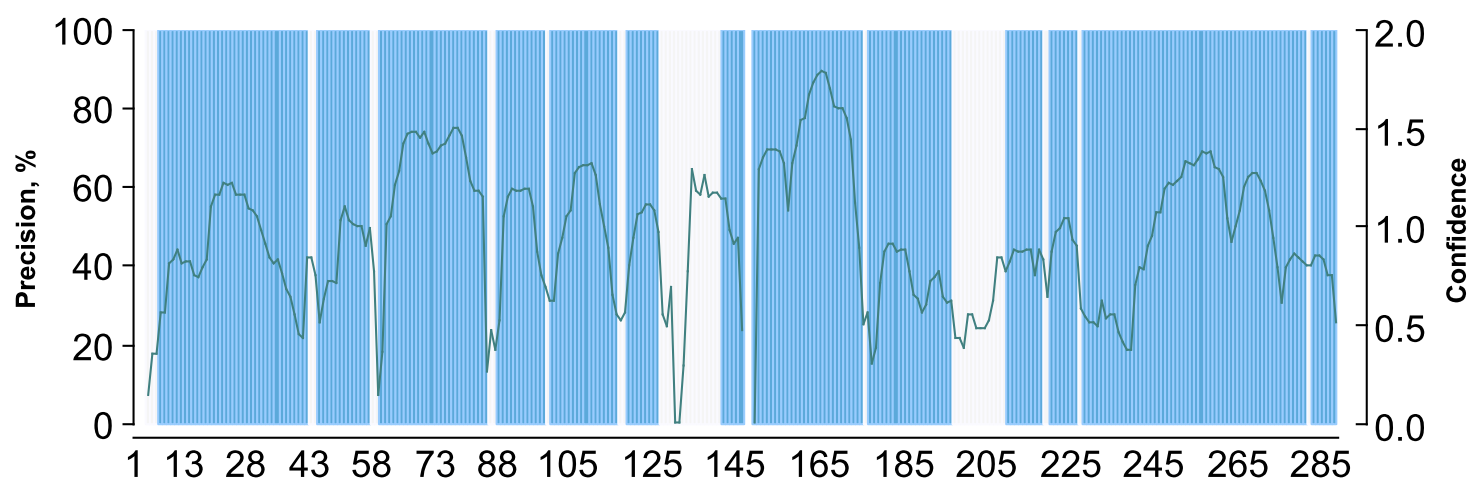

Supplement: Benchmark S1 — Local centroid precision for each target in the benchmark set and a breakdown of the torsion angle prediction performance by residue type and secondary structure. (ZIP) [file pone.0076512.s001.zip › Filtering/3obiA.pdf]

3o0lA

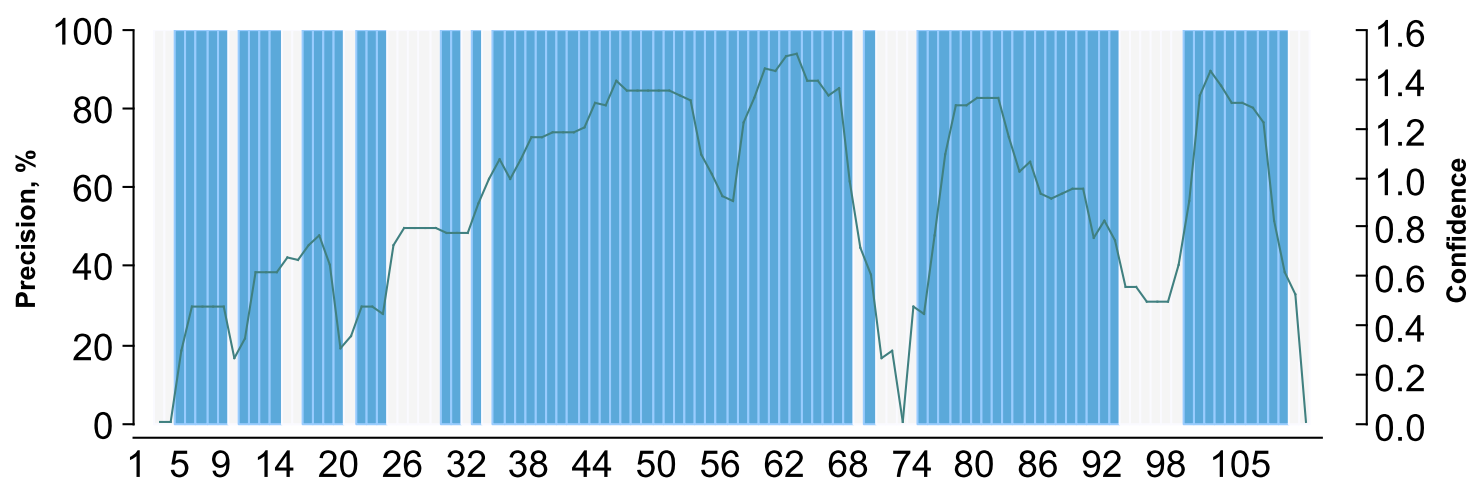

Supplement: Benchmark S1 — Local centroid precision for each target in the benchmark set and a breakdown of the torsion angle prediction performance by residue type and secondary structure. (ZIP) [file pone.0076512.s001.zip › Filtering/3o0lA.pdf]

### 3nnrA

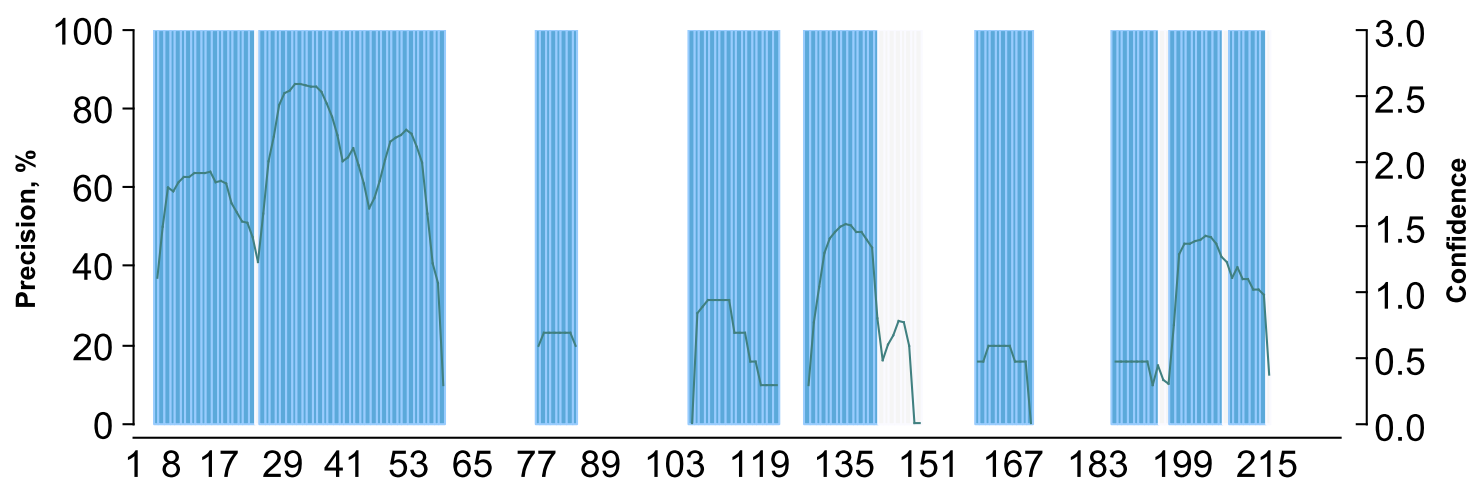

Supplement: Benchmark S1 — Local centroid precision for each target in the benchmark set and a breakdown of the torsion angle prediction performance by residue type and secondary structure. (ZIP) [file pone.0076512.s001.zip › Filtering/3nnrA.pdf]

# 3ot2A

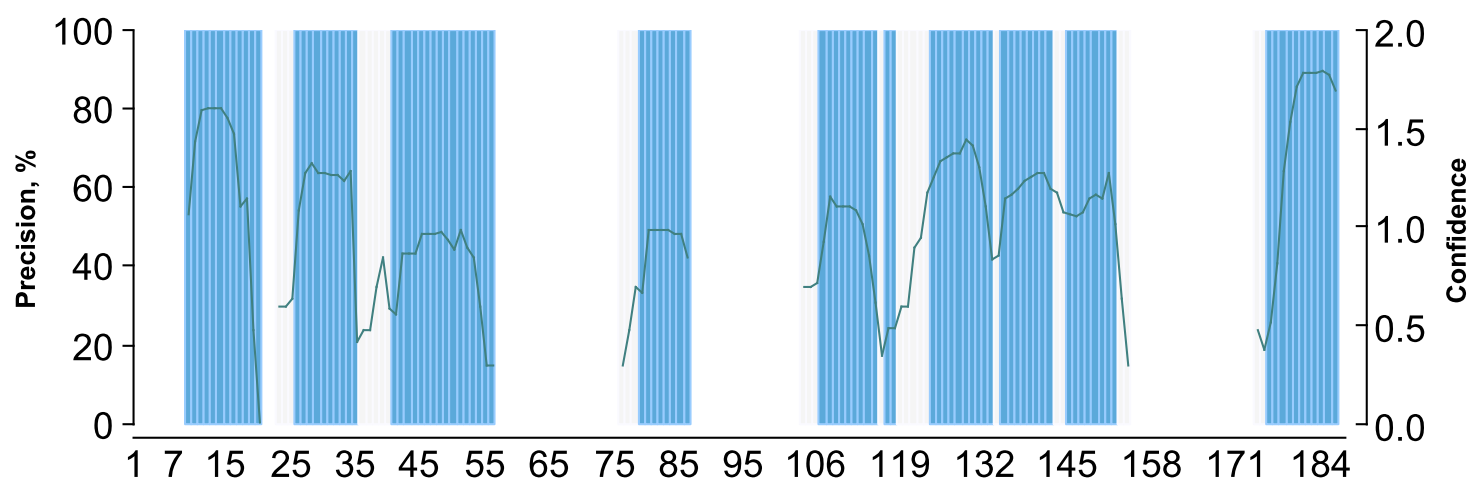

Supplement: Benchmark S1 — Local centroid precision for each target in the benchmark set and a breakdown of the torsion angle prediction performance by residue type and secondary structure. (ZIP) [file pone.0076512.s001.zip › Filtering/3ot2A.pdf]

# 3os7A

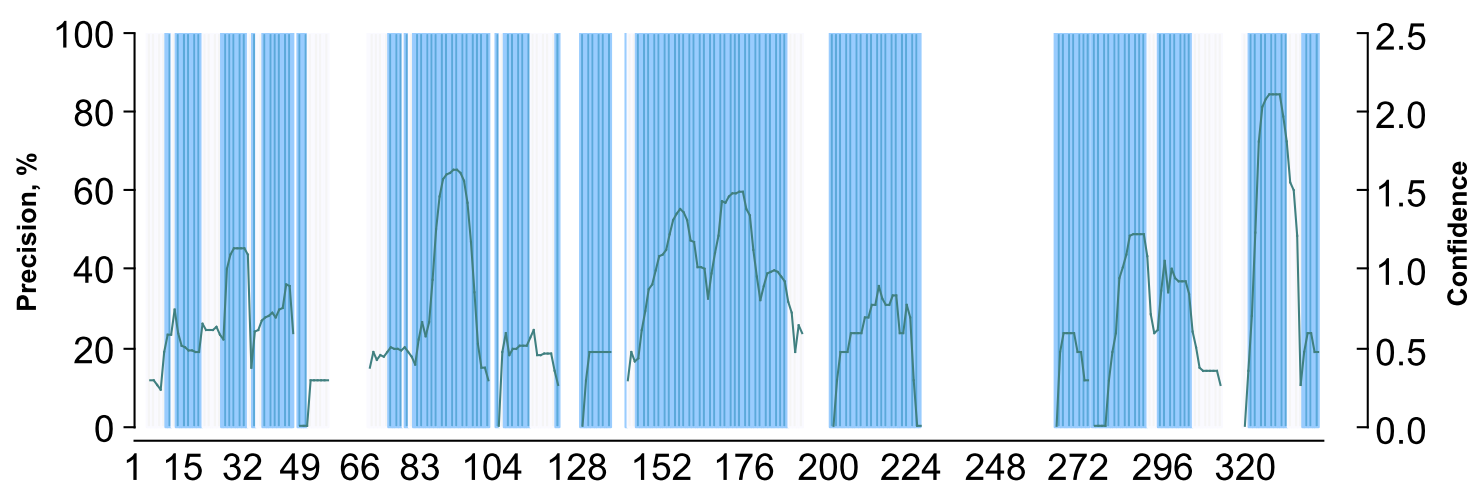

Supplement: Benchmark S1 — Local centroid precision for each target in the benchmark set and a breakdown of the torsion angle prediction performance by residue type and secondary structure. (ZIP) [file pone.0076512.s001.zip › Filtering/3os7A.pdf]

3nyyA

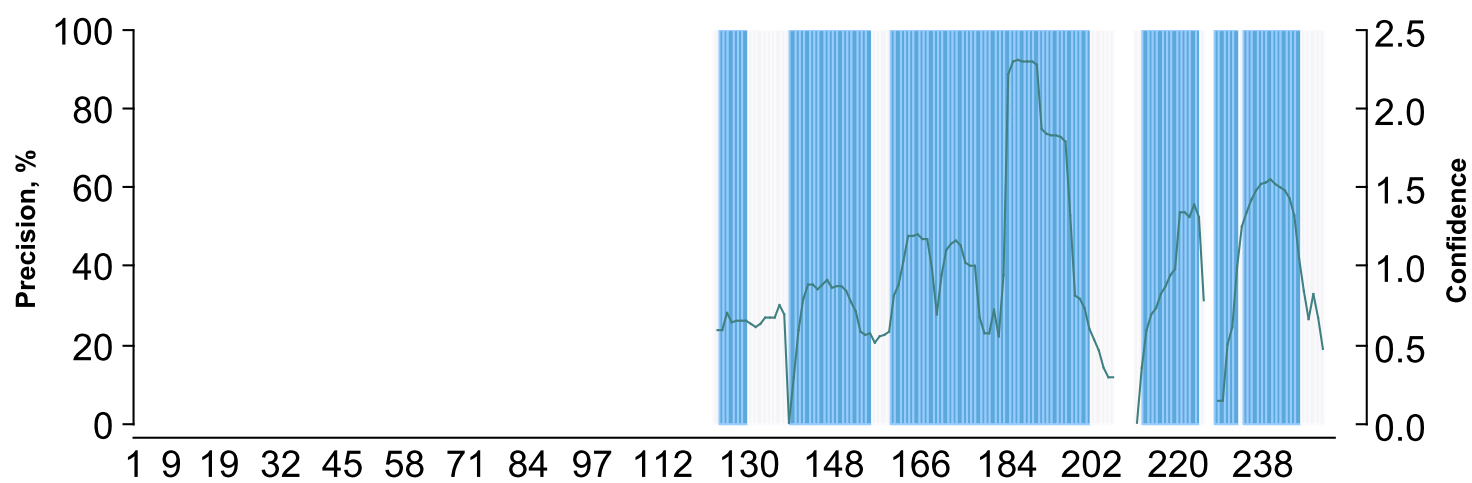

Supplement: Benchmark S1 — Local centroid precision for each target in the benchmark set and a breakdown of the torsion angle prediction performance by residue type and secondary structure. (ZIP) [file pone.0076512.s001.zip › Filtering/3nyyA.pdf]

3nmdA

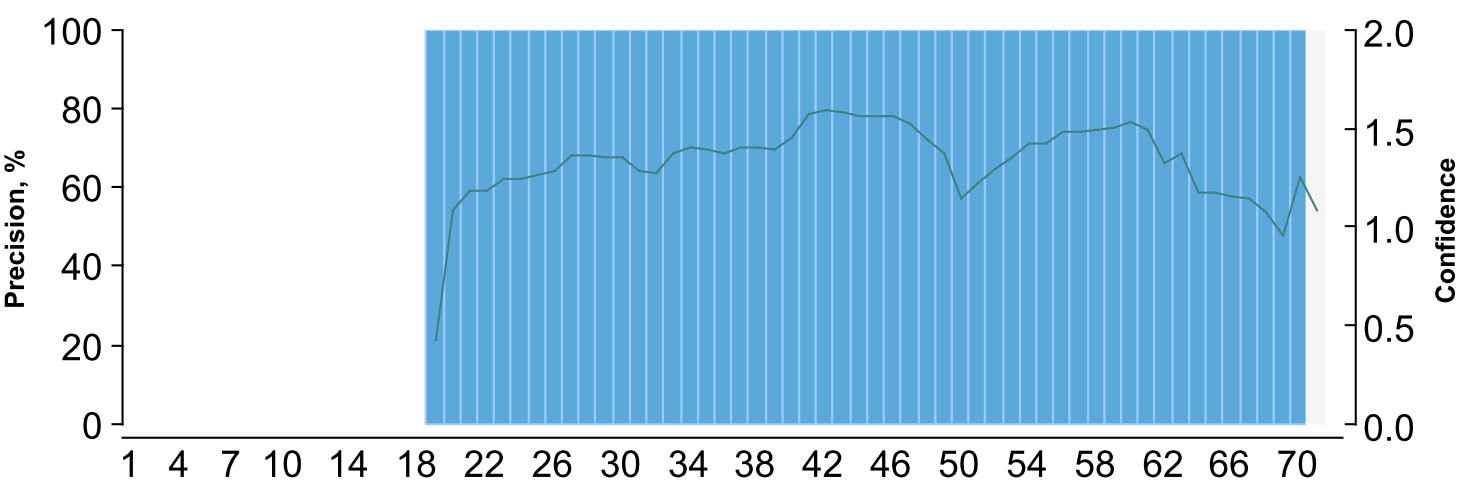

Supplement: Benchmark S1 — Local centroid precision for each target in the benchmark set and a breakdown of the torsion angle prediction performance by residue type and secondary structure. (ZIP) [file pone.0076512.s001.zip › Filtering/3nmdA.pdf]

# 3nlcA

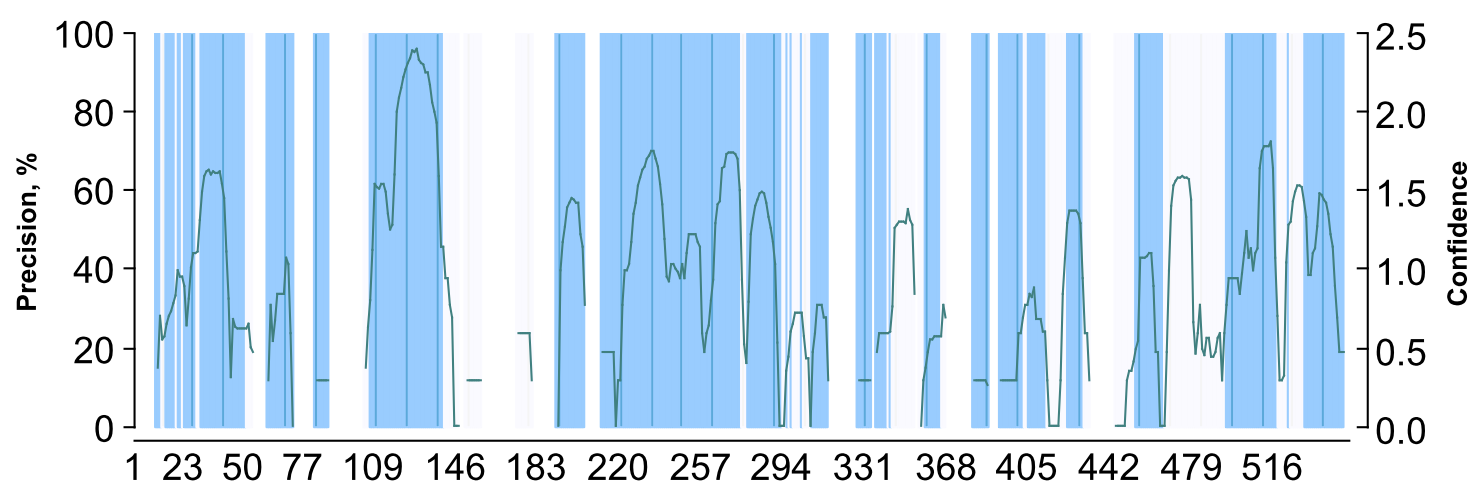

Supplement: Benchmark S1 — Local centroid precision for each target in the benchmark set and a breakdown of the torsion angle prediction performance by residue type and secondary structure. (ZIP) [file pone.0076512.s001.zip › Filtering/3nlcA.pdf]

# 3nkdA

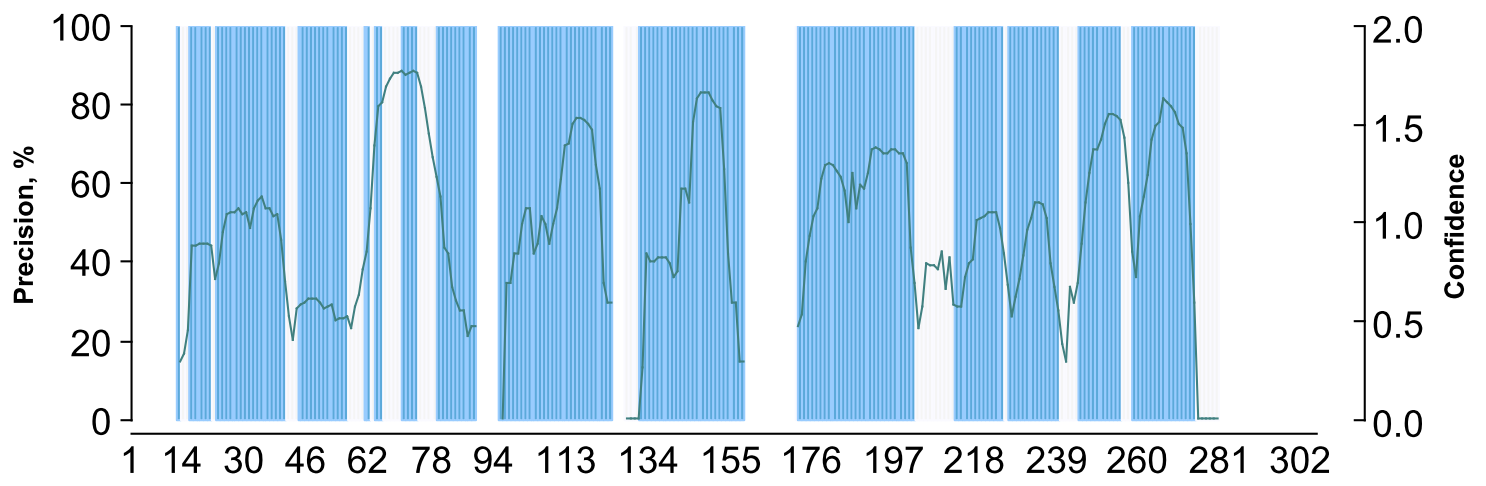

Supplement: Benchmark S1 — Local centroid precision for each target in the benchmark set and a breakdown of the torsion angle prediction performance by residue type and secondary structure. (ZIP) [file pone.0076512.s001.zip › Filtering/3nkdA.pdf]

3nkzA

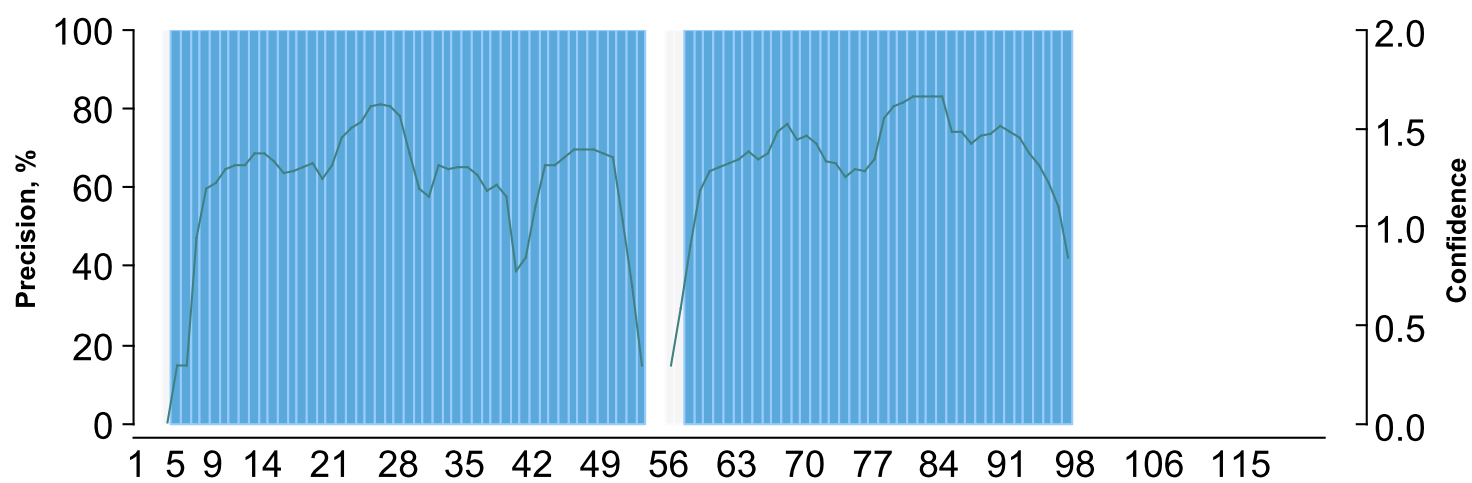

Supplement: Benchmark S1 — Local centroid precision for each target in the benchmark set and a breakdown of the torsion angle prediction performance by residue type and secondary structure. (ZIP) [file pone.0076512.s001.zip › Filtering/3nkzA.pdf]

# 3njaA

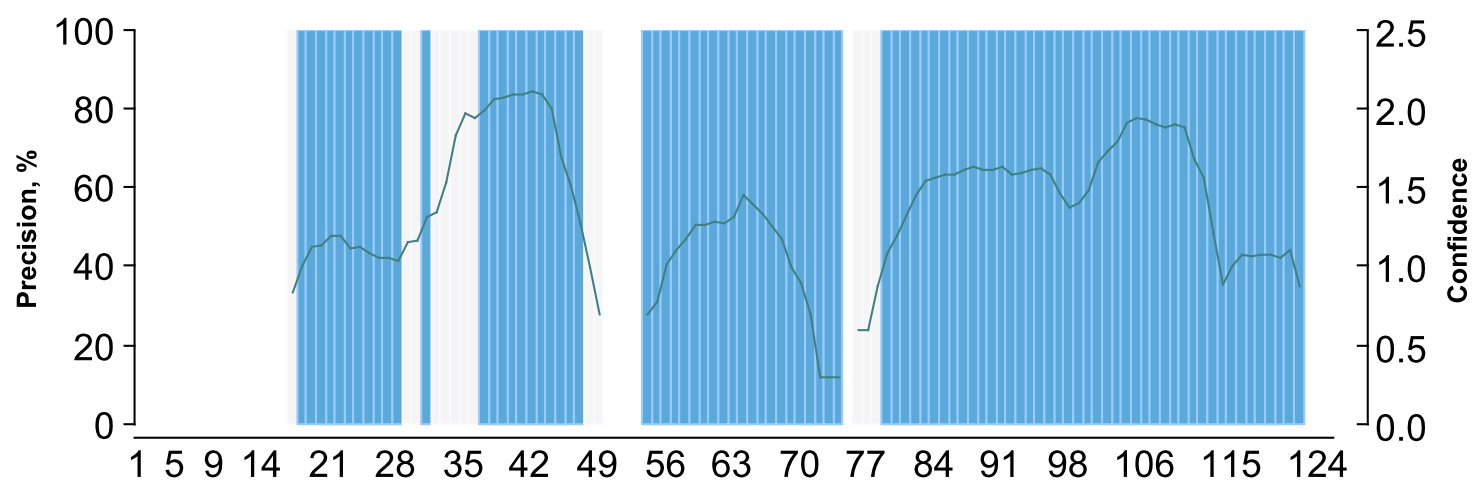

Supplement: Benchmark S1 — Local centroid precision for each target in the benchmark set and a breakdown of the torsion angle prediction performance by residue type and secondary structure. (ZIP) [file pone.0076512.s001.zip › Filtering/3njaA.pdf]

# 3os6A

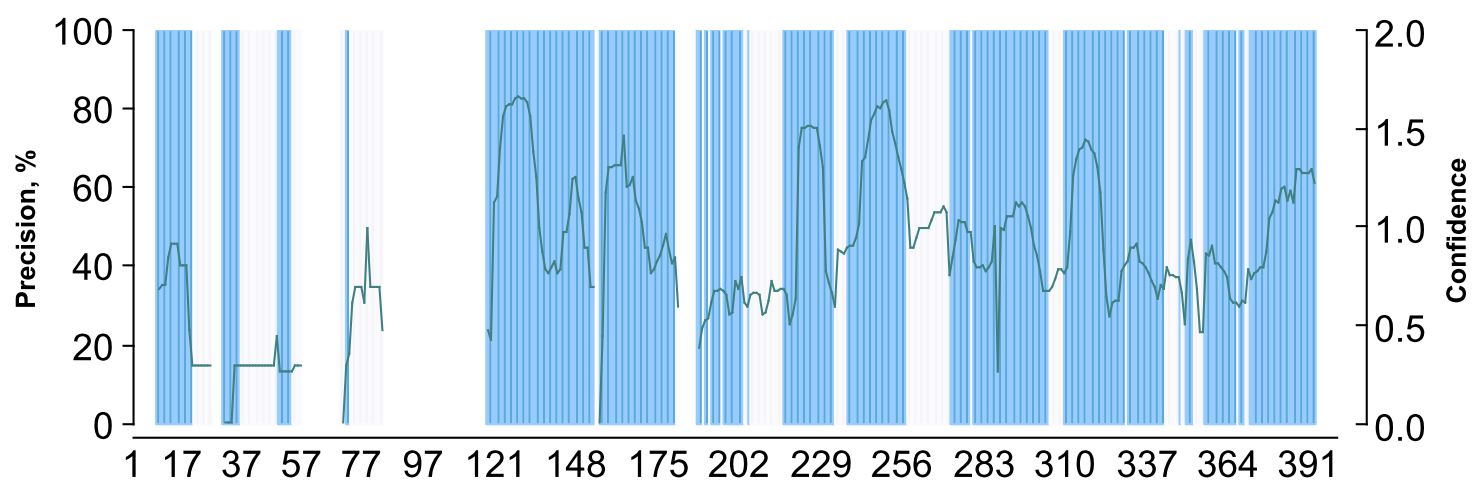

Supplement: Benchmark S1 — Local centroid precision for each target in the benchmark set and a breakdown of the torsion angle prediction performance by residue type and secondary structure. (ZIP) [file pone.0076512.s001.zip › Filtering/3os6A.pdf]

3nqwA

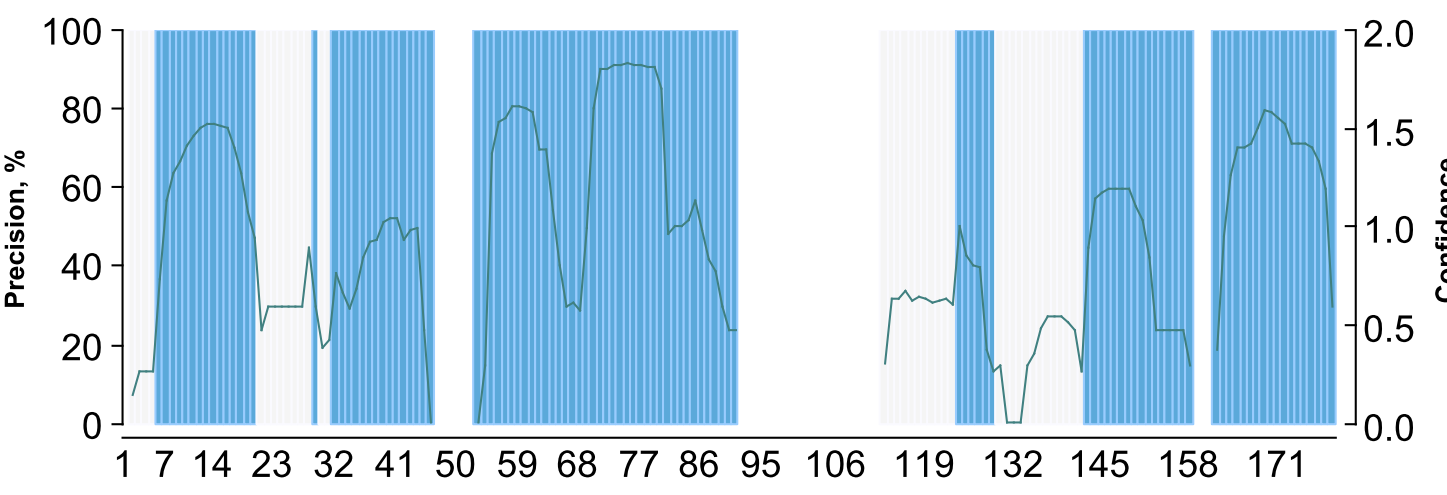

Supplement: Benchmark S1 — Local centroid precision for each target in the benchmark set and a breakdown of the torsion angle prediction performance by residue type and secondary structure. (ZIP) [file pone.0076512.s001.zip › Filtering/3nqwA.pdf]

# 3njcA

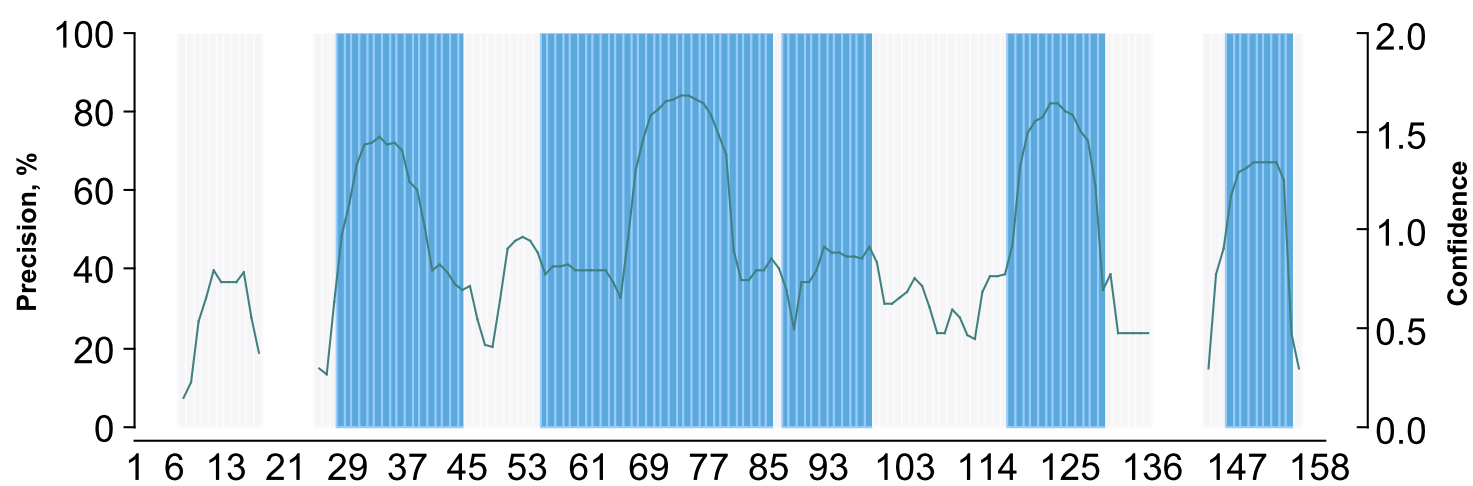

Supplement: Benchmark S1 — Local centroid precision for each target in the benchmark set and a breakdown of the torsion angle prediction performance by residue type and secondary structure. (ZIP) [file pone.0076512.s001.zip › Filtering/3njcA.pdf]

# 3nieA

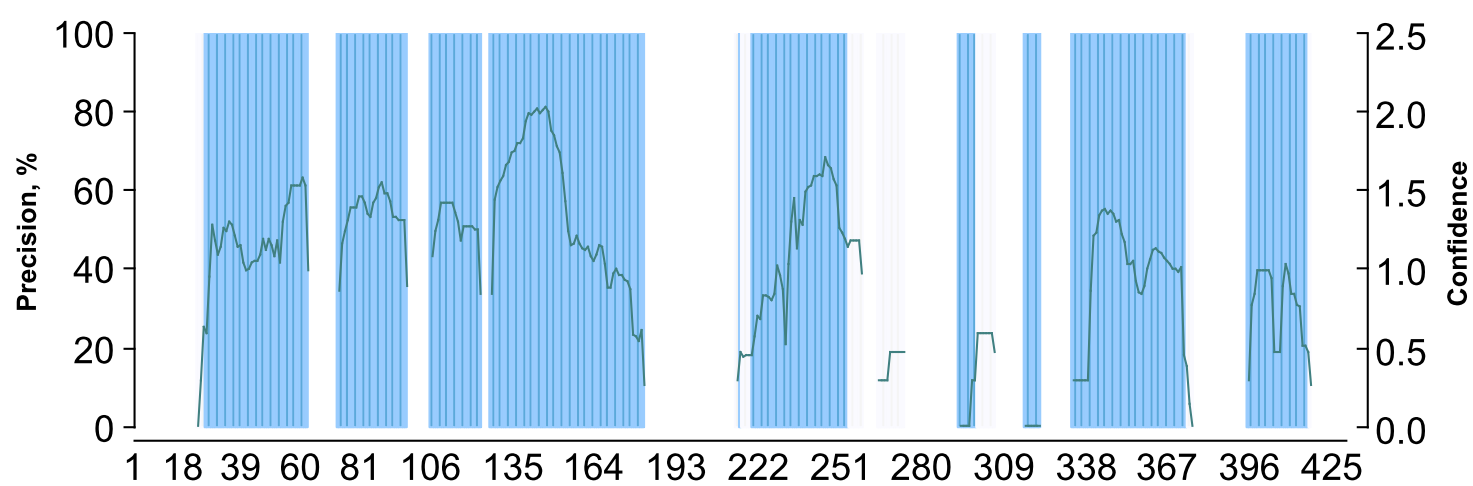

Supplement: Benchmark S1 — Local centroid precision for each target in the benchmark set and a breakdown of the torsion angle prediction performance by residue type and secondary structure. (ZIP) [file pone.0076512.s001.zip › Filtering/3nieA.pdf]

3ni7A

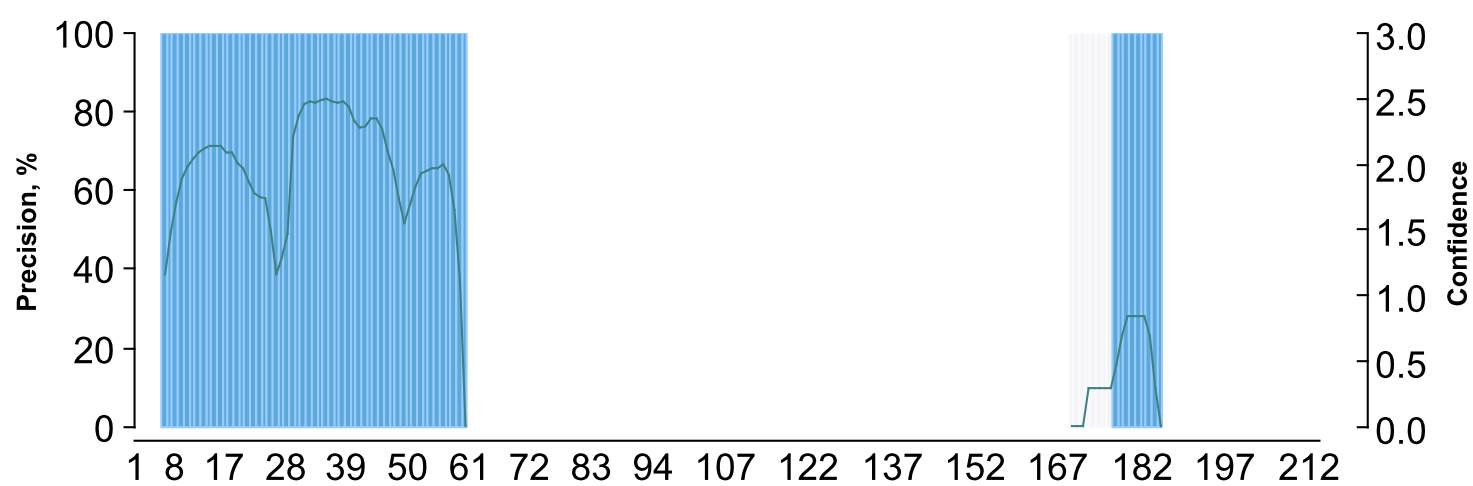

Supplement: Benchmark S1 — Local centroid precision for each target in the benchmark set and a breakdown of the torsion angle prediction performance by residue type and secondary structure. (ZIP) [file pone.0076512.s001.zip › Filtering/3ni7A.pdf]

# 3ni8A

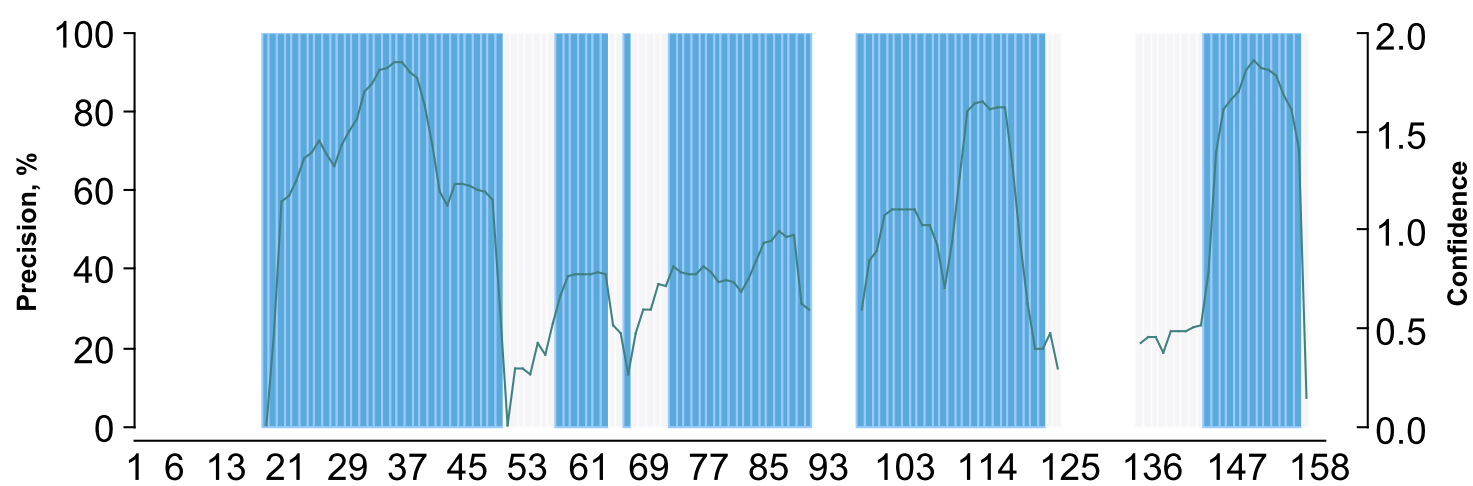

Supplement: Benchmark S1 — Local centroid precision for each target in the benchmark set and a breakdown of the torsion angle prediction performance by residue type and secondary structure. (ZIP) [file pone.0076512.s001.zip › Filtering/3ni8A.pdf]

3ngwA

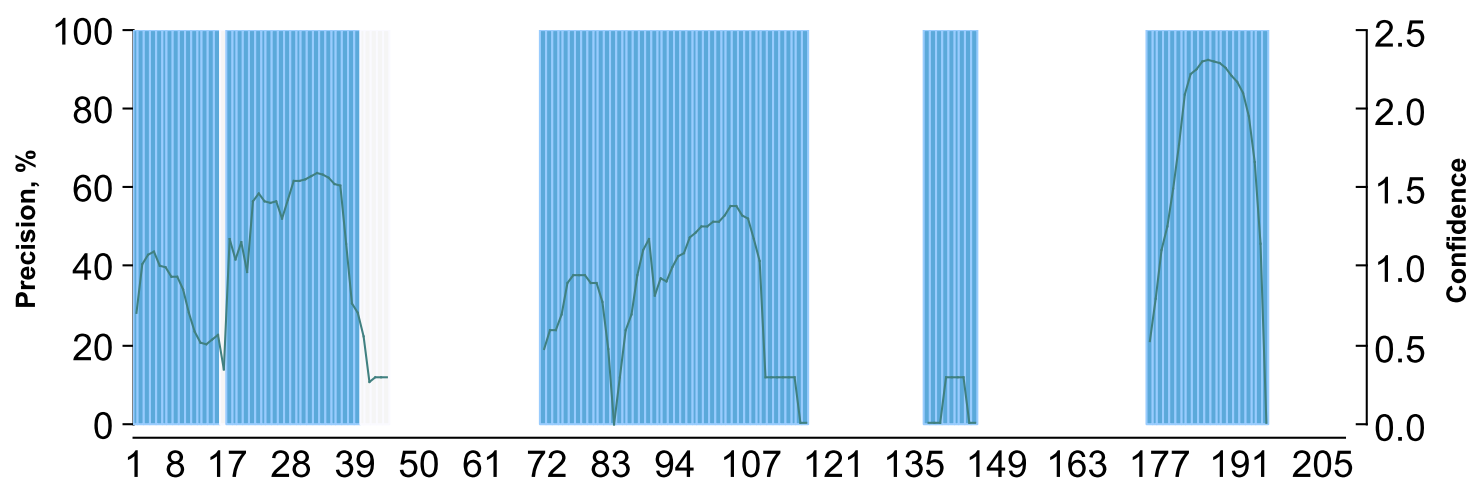

Supplement: Benchmark S1 — Local centroid precision for each target in the benchmark set and a breakdown of the torsion angle prediction performance by residue type and secondary structure. (ZIP) [file pone.0076512.s001.zip › Filtering/3ngwA.pdf]

# 3nohA

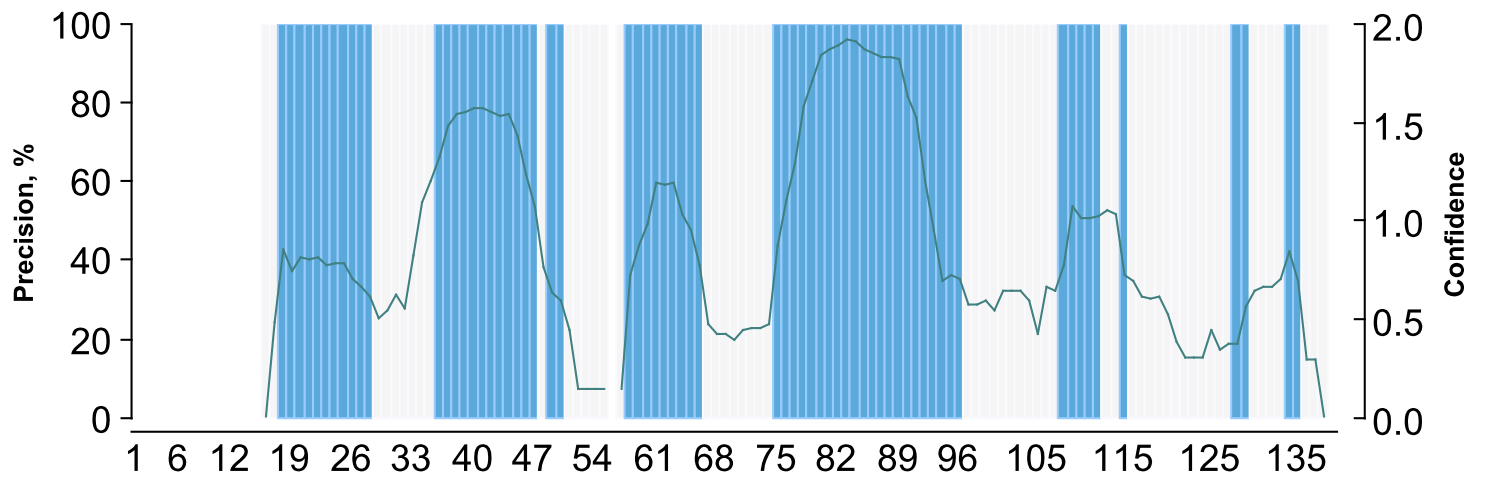

Supplement: Benchmark S1 — Local centroid precision for each target in the benchmark set and a breakdown of the torsion angle prediction performance by residue type and secondary structure. (ZIP) [file pone.0076512.s001.zip › Filtering/3nohA.pdf]

3nhvA

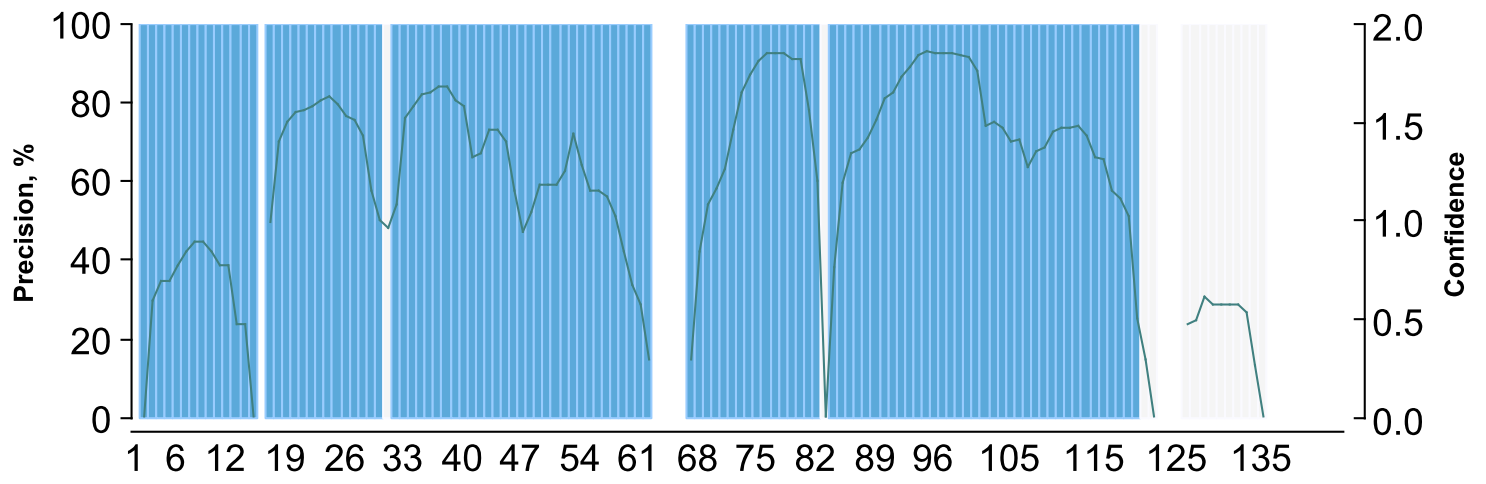

Supplement: Benchmark S1 — Local centroid precision for each target in the benchmark set and a breakdown of the torsion angle prediction performance by residue type and secondary structure. (ZIP) [file pone.0076512.s001.zip › Filtering/3nhvA.pdf]

# 3nraA

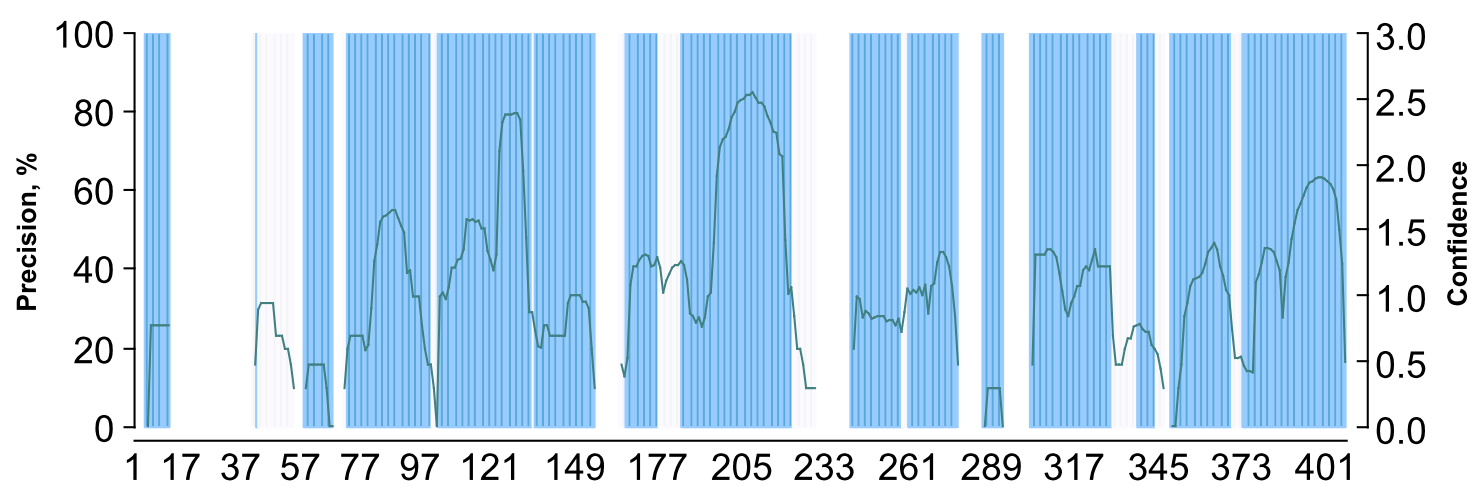

Supplement: Benchmark S1 — Local centroid precision for each target in the benchmark set and a breakdown of the torsion angle prediction performance by residue type and secondary structure. (ZIP) [file pone.0076512.s001.zip › Filtering/3nraA.pdf]

2kzwA

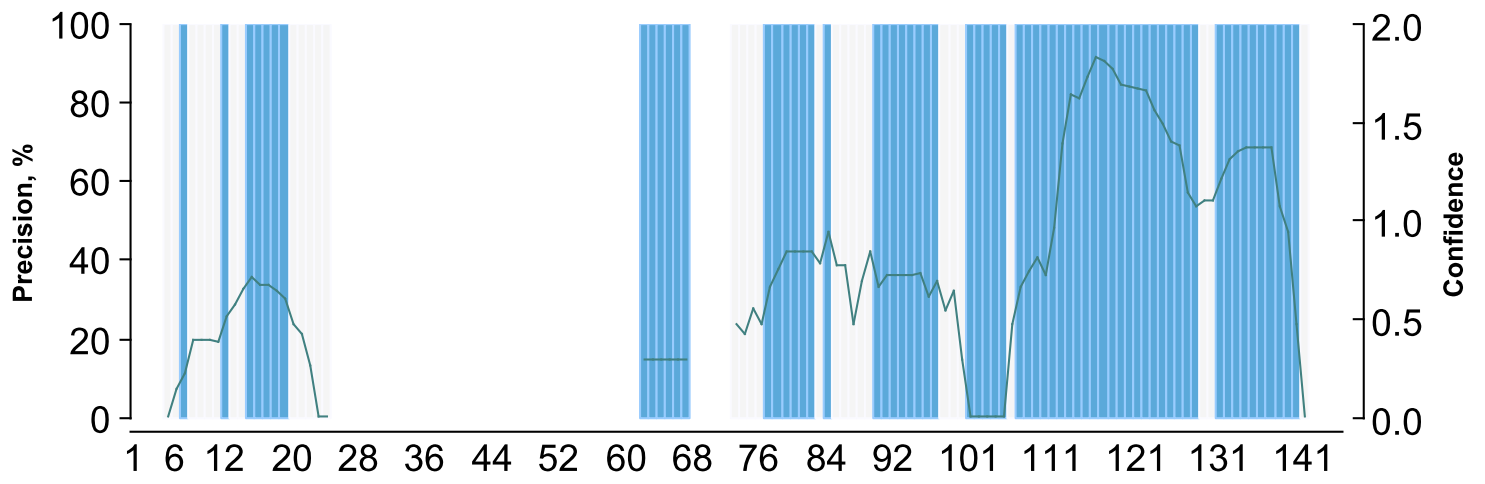

Supplement: Benchmark S1 — Local centroid precision for each target in the benchmark set and a breakdown of the torsion angle prediction performance by residue type and secondary structure. (ZIP) [file pone.0076512.s001.zip › Filtering/2kzwA.pdf]

### 3netA

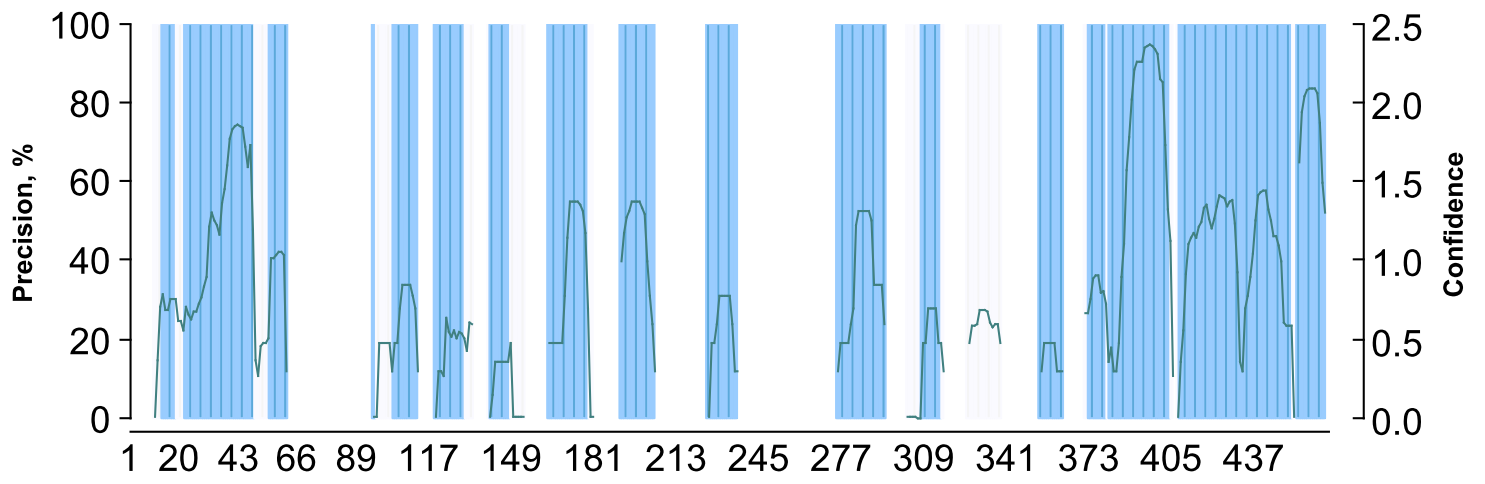

Supplement: Benchmark S1 — Local centroid precision for each target in the benchmark set and a breakdown of the torsion angle prediction performance by residue type and secondary structure. (ZIP) [file pone.0076512.s001.zip › Filtering/3netA.pdf]

### 3nfvA

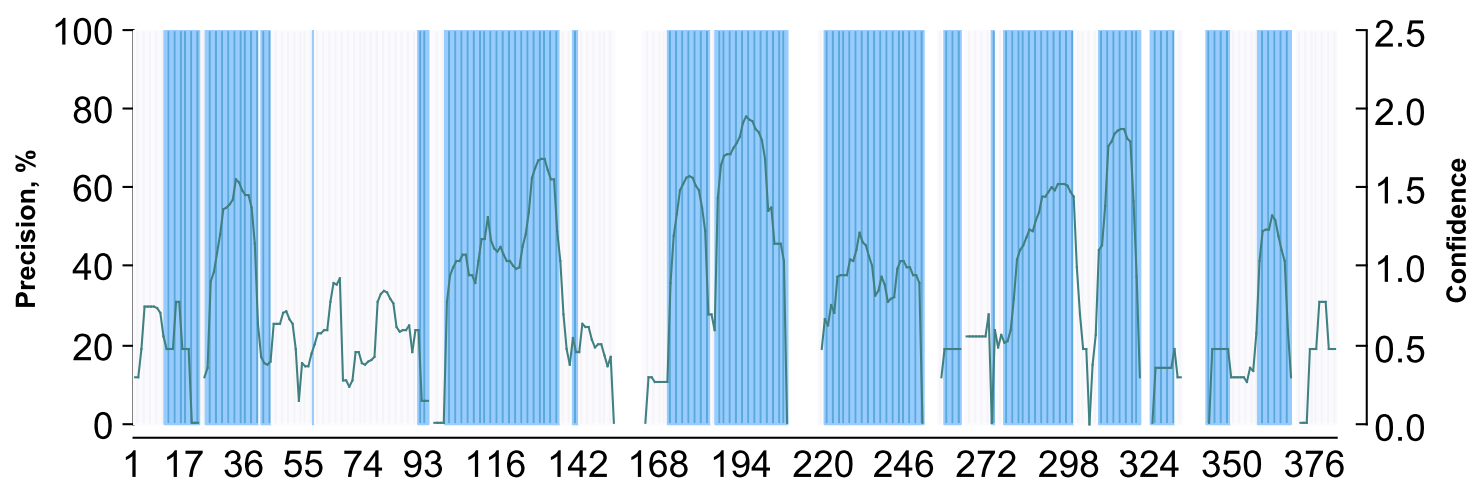

Supplement: Benchmark S1 — Local centroid precision for each target in the benchmark set and a breakdown of the torsion angle prediction performance by residue type and secondary structure. (ZIP) [file pone.0076512.s001.zip › Filtering/3nfvA.pdf]

### 3neuA

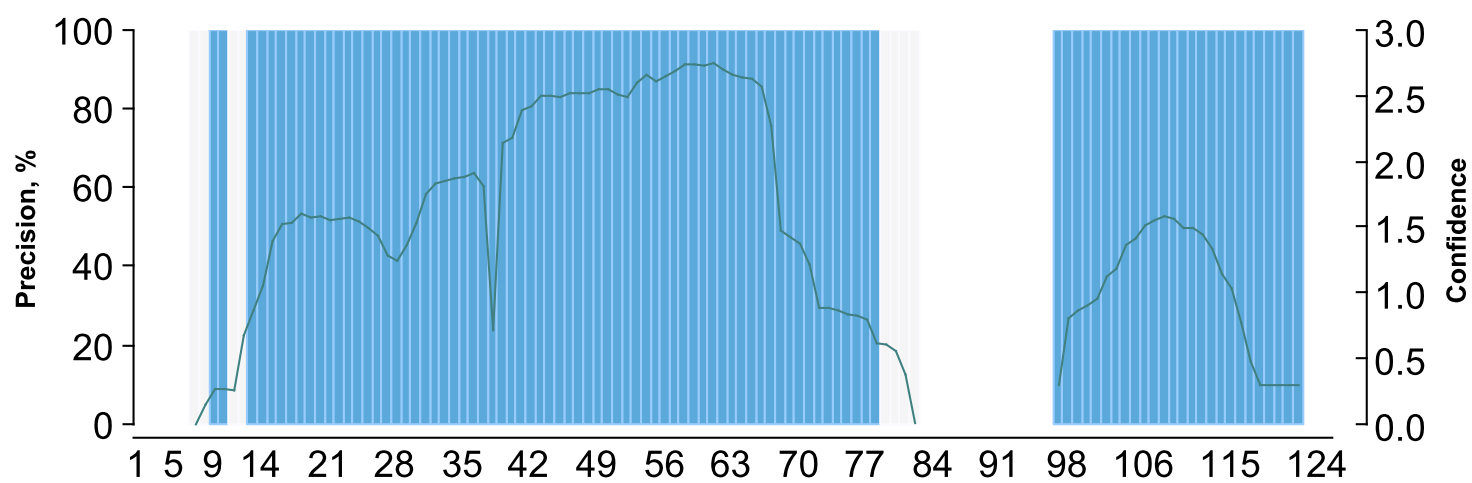

Supplement: Benchmark S1 — Local centroid precision for each target in the benchmark set and a breakdown of the torsion angle prediction performance by residue type and secondary structure. (ZIP) [file pone.0076512.s001.zip › Filtering/3neuA.pdf]

3ne8A

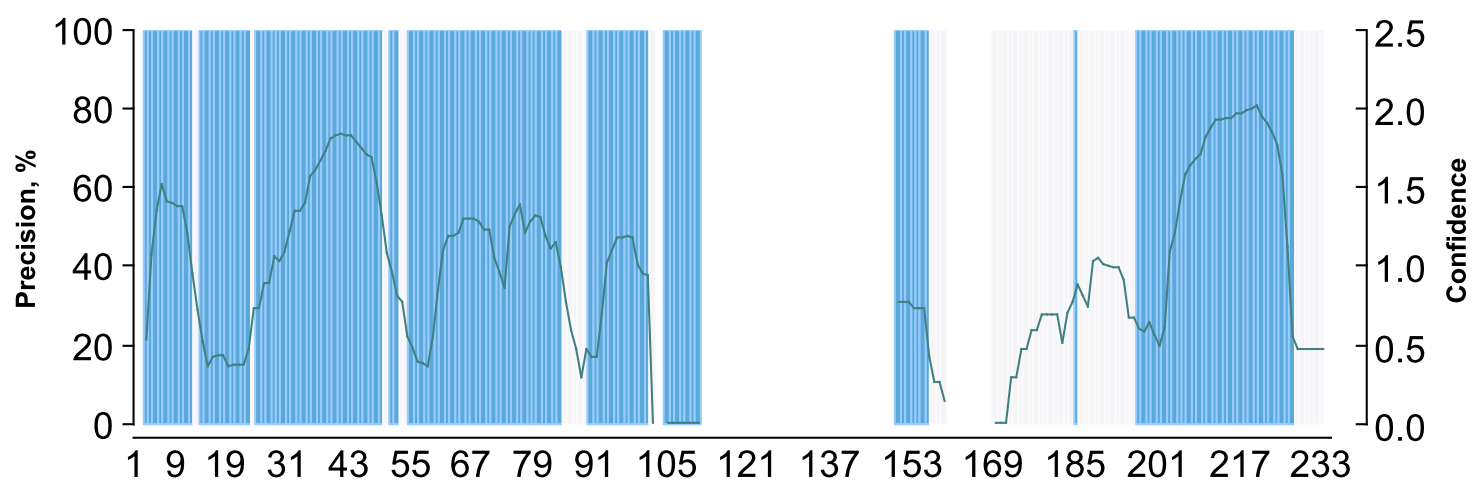

Supplement: Benchmark S1 — Local centroid precision for each target in the benchmark set and a breakdown of the torsion angle prediction performance by residue type and secondary structure. (ZIP) [file pone.0076512.s001.zip › Filtering/3ne8A.pdf]

# 3nf2A

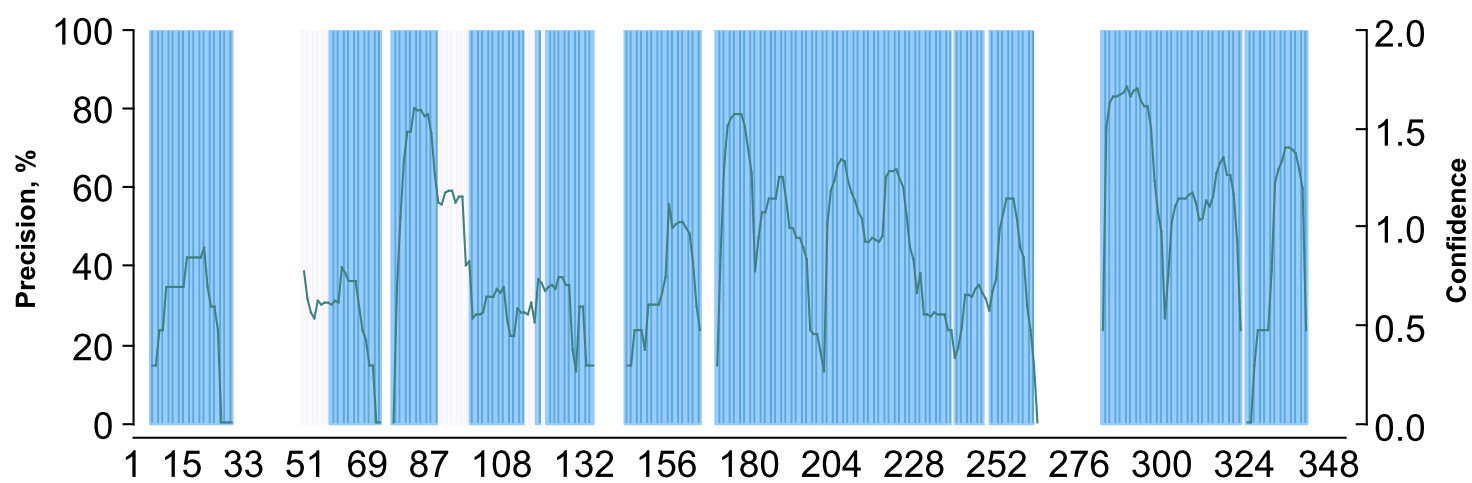

Supplement: Benchmark S1 — Local centroid precision for each target in the benchmark set and a breakdown of the torsion angle prediction performance by residue type and secondary structure. (ZIP) [file pone.0076512.s001.zip › Filtering/3nf2A.pdf]

3o14A

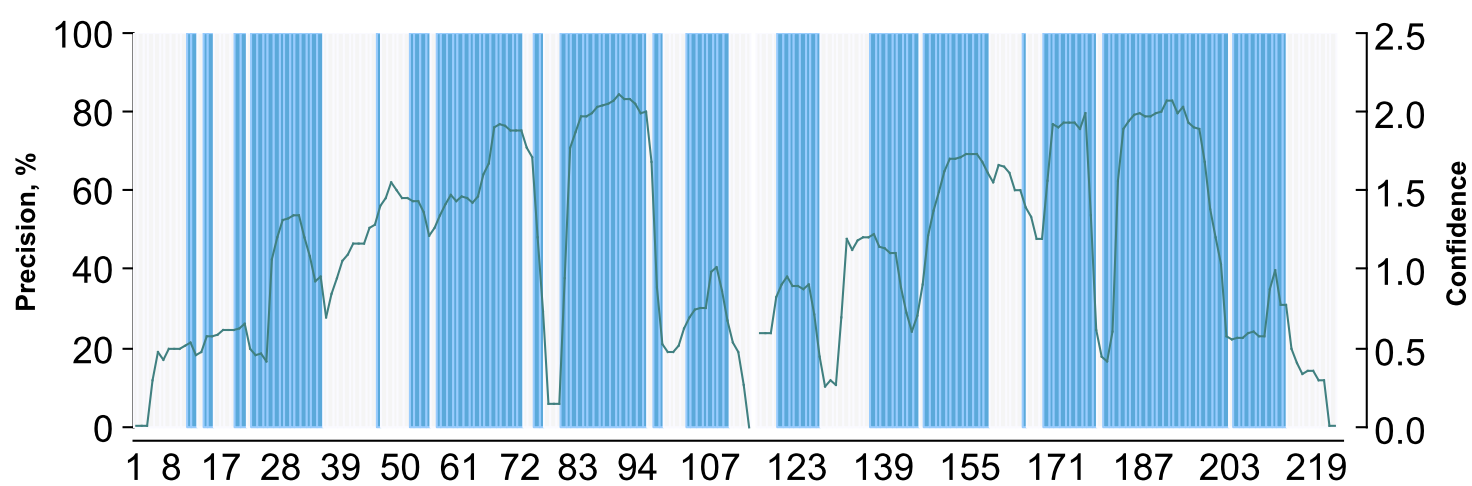

Supplement: Benchmark S1 — Local centroid precision for each target in the benchmark set and a breakdown of the torsion angle prediction performance by residue type and secondary structure. (ZIP) [file pone.0076512.s001.zip › Filtering/3o14A.pdf]

3nbmA

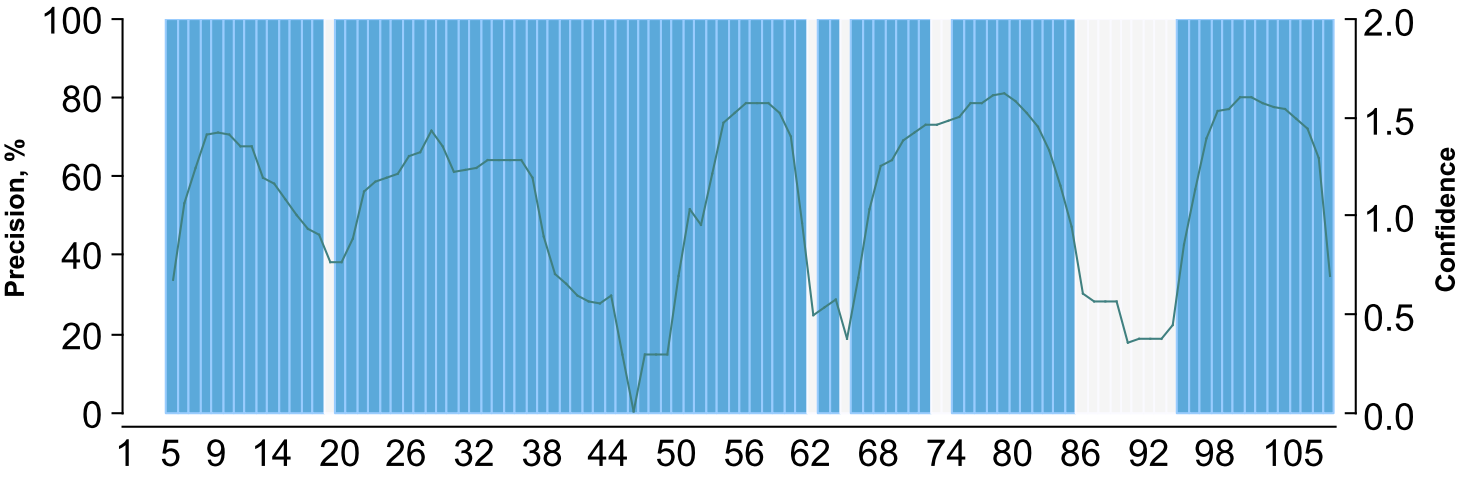

Supplement: Benchmark S1 — Local centroid precision for each target in the benchmark set and a breakdown of the torsion angle prediction performance by residue type and secondary structure. (ZIP) [file pone.0076512.s001.zip › Filtering/3nbmA.pdf]

2ky9A

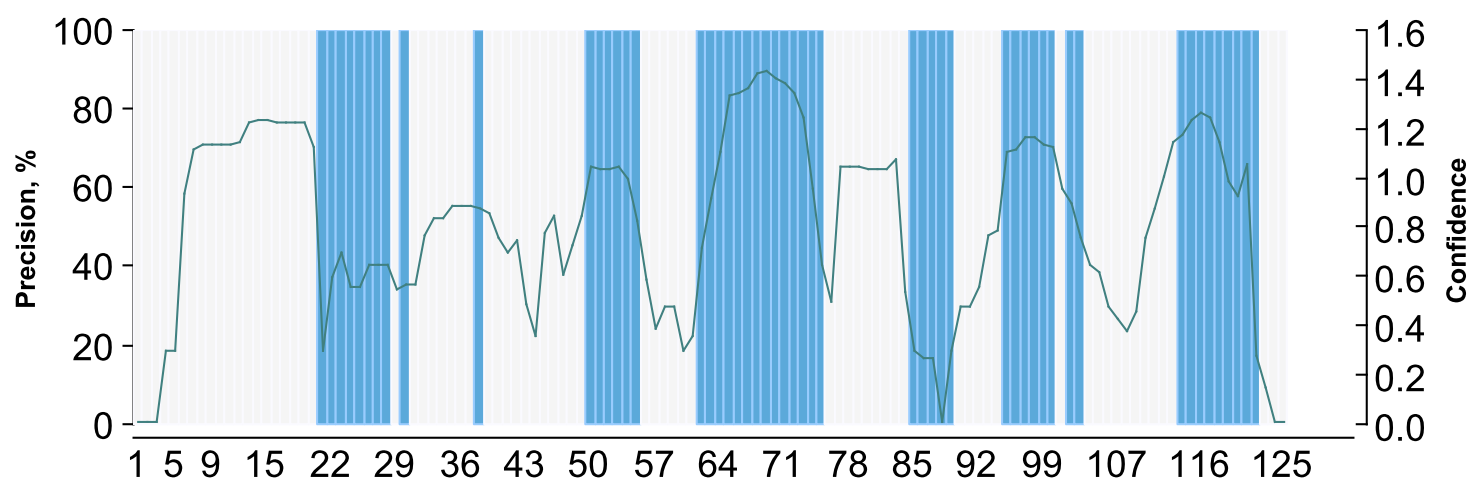

Supplement: Benchmark S1 — Local centroid precision for each target in the benchmark set and a breakdown of the torsion angle prediction performance by residue type and secondary structure. (ZIP) [file pone.0076512.s001.zip › Filtering/2ky9A.pdf]

# 3na2A

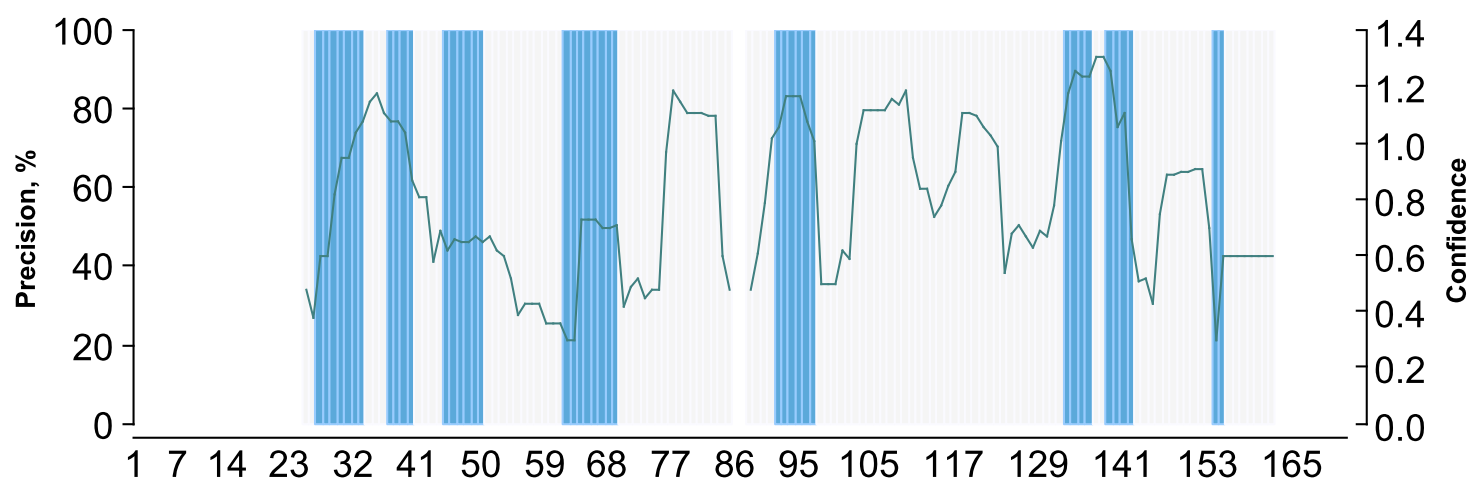

Supplement: Benchmark S1 — Local centroid precision for each target in the benchmark set and a breakdown of the torsion angle prediction performance by residue type and secondary structure. (ZIP) [file pone.0076512.s001.zip › Filtering/3na2A.pdf]

# 3nrgA

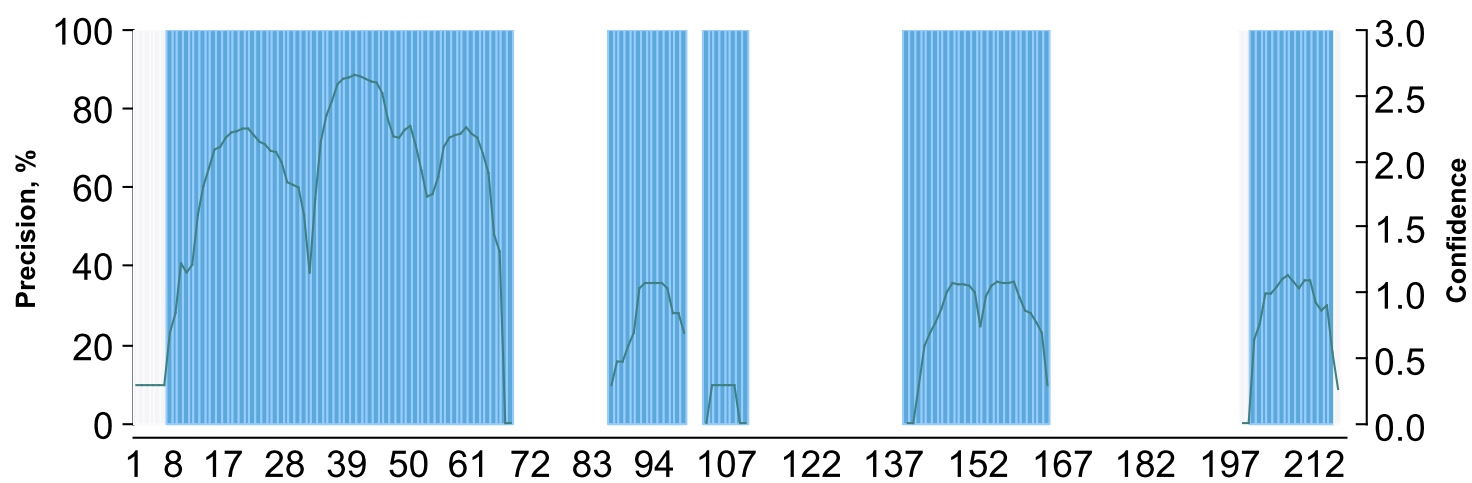

Supplement: Benchmark S1 — Local centroid precision for each target in the benchmark set and a breakdown of the torsion angle prediction performance by residue type and secondary structure. (ZIP) [file pone.0076512.s001.zip › Filtering/3nrgA.pdf]

### 3nrfA

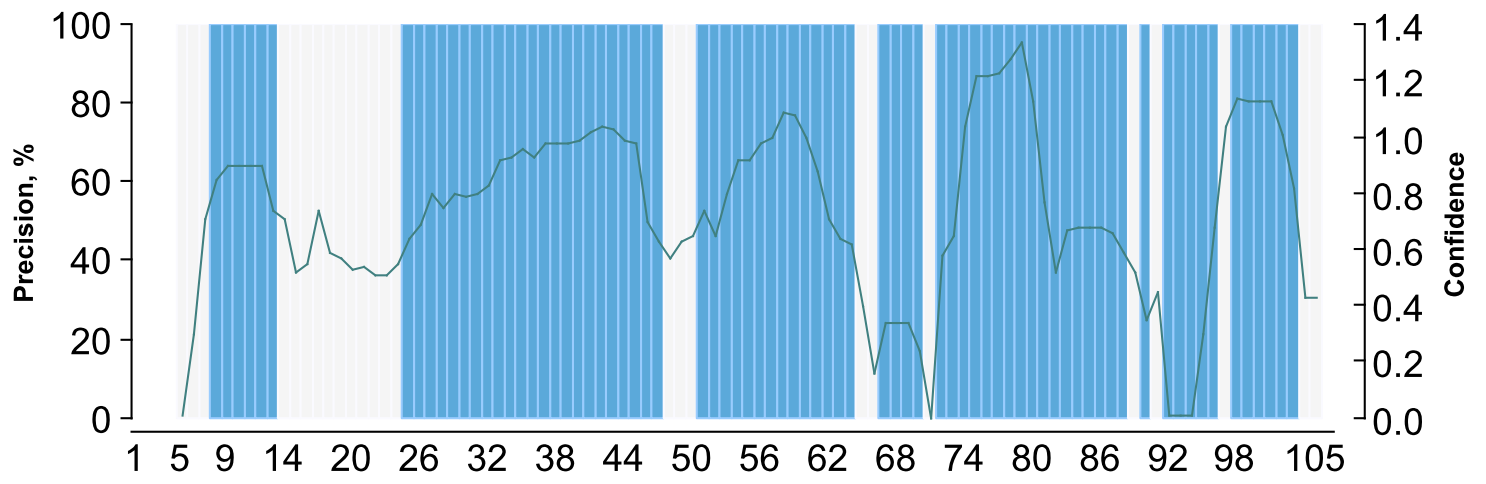

Supplement: Benchmark S1 — Local centroid precision for each target in the benchmark set and a breakdown of the torsion angle prediction performance by residue type and secondary structure. (ZIP) [file pone.0076512.s001.zip › Filtering/3nrfA.pdf]

# 2kxyA

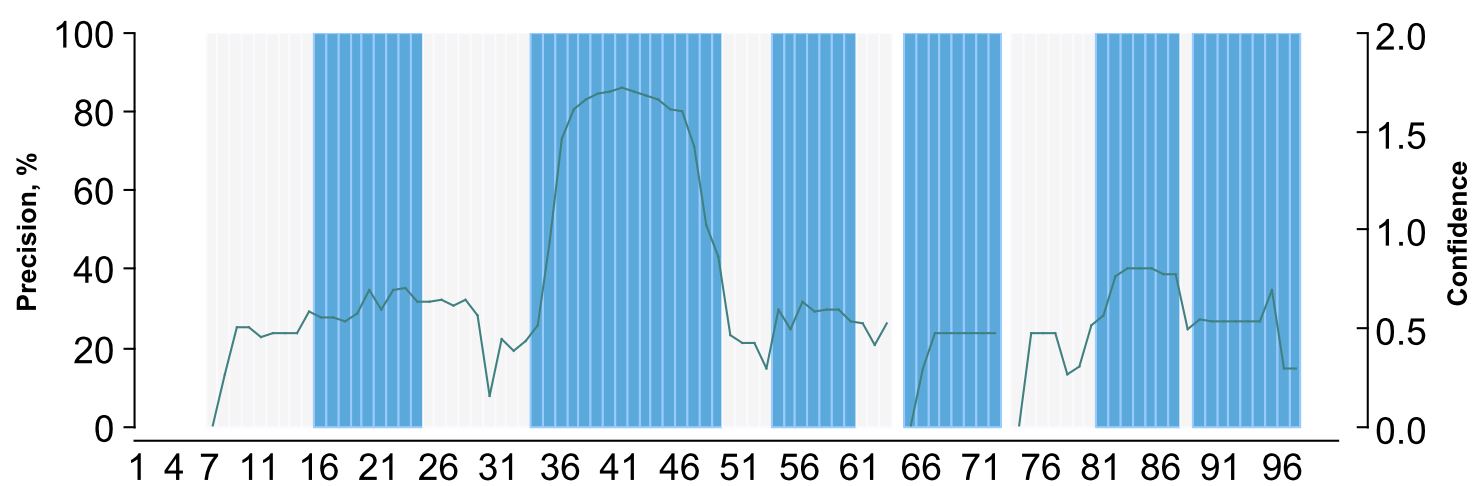

Supplement: Benchmark S1 — Local centroid precision for each target in the benchmark set and a breakdown of the torsion angle prediction performance by residue type and secondary structure. (ZIP) [file pone.0076512.s001.zip › Filtering/2kxyA.pdf]

### 3no3A

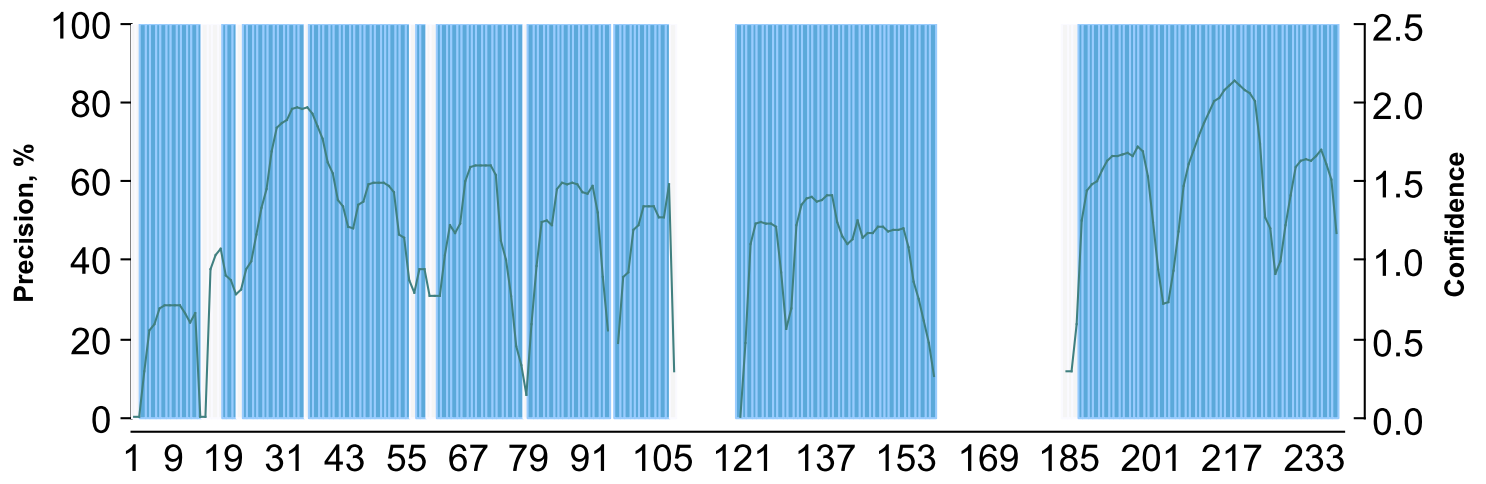

Supplement: Benchmark S1 — Local centroid precision for each target in the benchmark set and a breakdown of the torsion angle prediction performance by residue type and secondary structure. (ZIP) [file pone.0076512.s001.zip › Filtering/3no3A.pdf]

2kywA

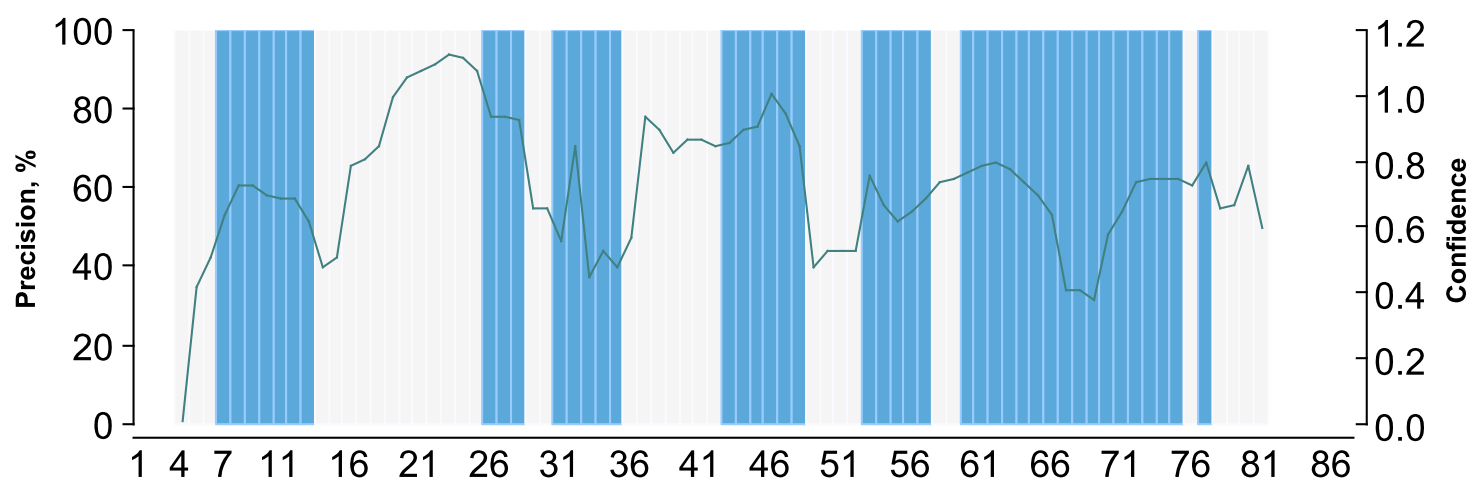

Supplement: Benchmark S1 — Local centroid precision for each target in the benchmark set and a breakdown of the torsion angle prediction performance by residue type and secondary structure. (ZIP) [file pone.0076512.s001.zip › Filtering/2kywA.pdf]

3n6yA

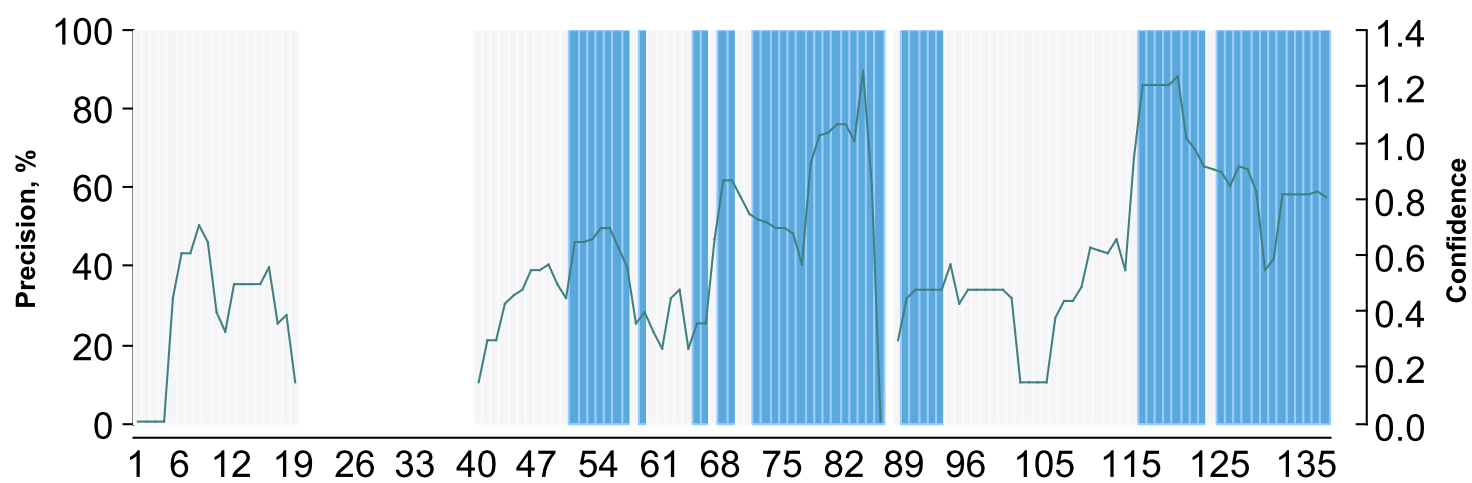

Supplement: Benchmark S1 — Local centroid precision for each target in the benchmark set and a breakdown of the torsion angle prediction performance by residue type and secondary structure. (ZIP) [file pone.0076512.s001.zip › Filtering/3n6yA.pdf]

3n70A

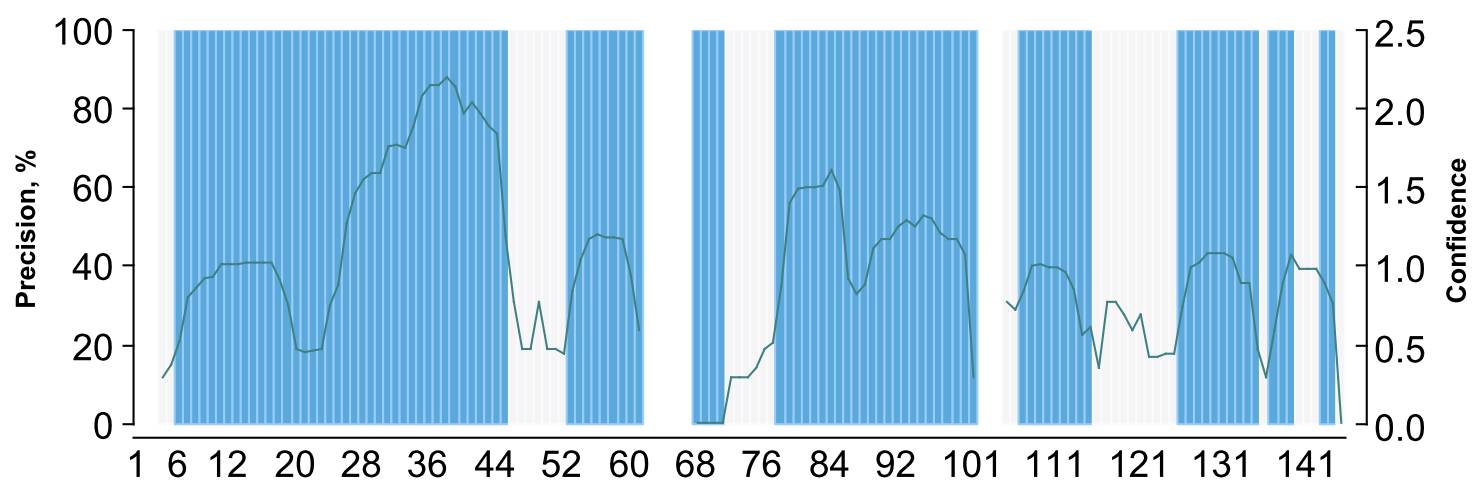

Supplement: Benchmark S1 — Local centroid precision for each target in the benchmark set and a breakdown of the torsion angle prediction performance by residue type and secondary structure. (ZIP) [file pone.0076512.s001.zip › Filtering/3n70A.pdf]

3n72A

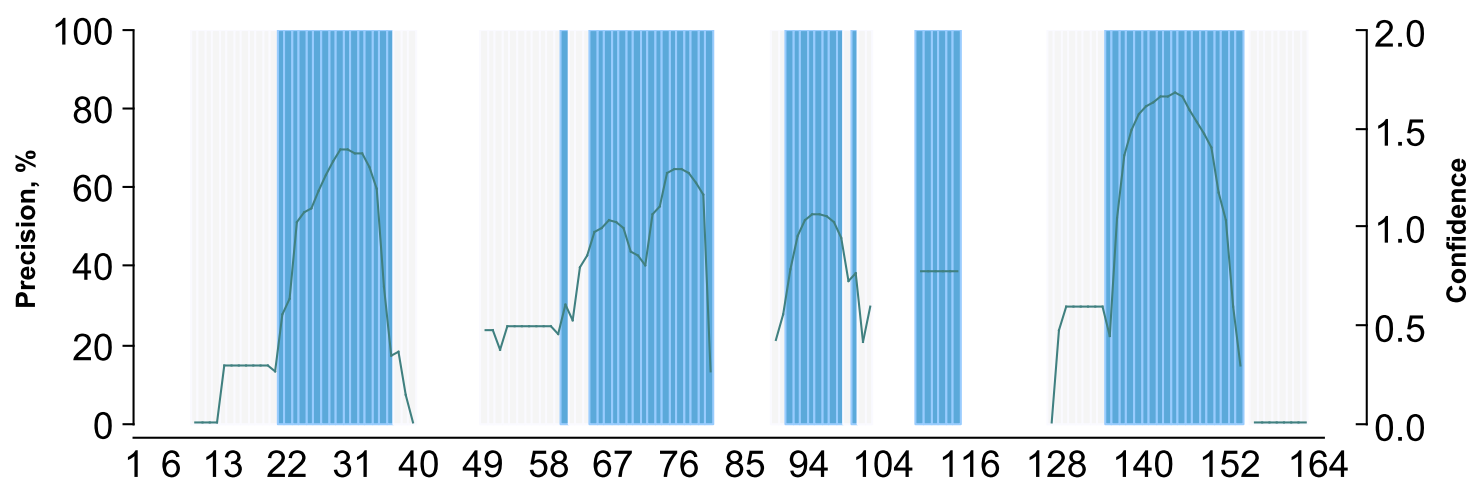

Supplement: Benchmark S1 — Local centroid precision for each target in the benchmark set and a breakdown of the torsion angle prediction performance by residue type and secondary structure. (ZIP) [file pone.0076512.s001.zip › Filtering/3n72A.pdf]

# 3npfA

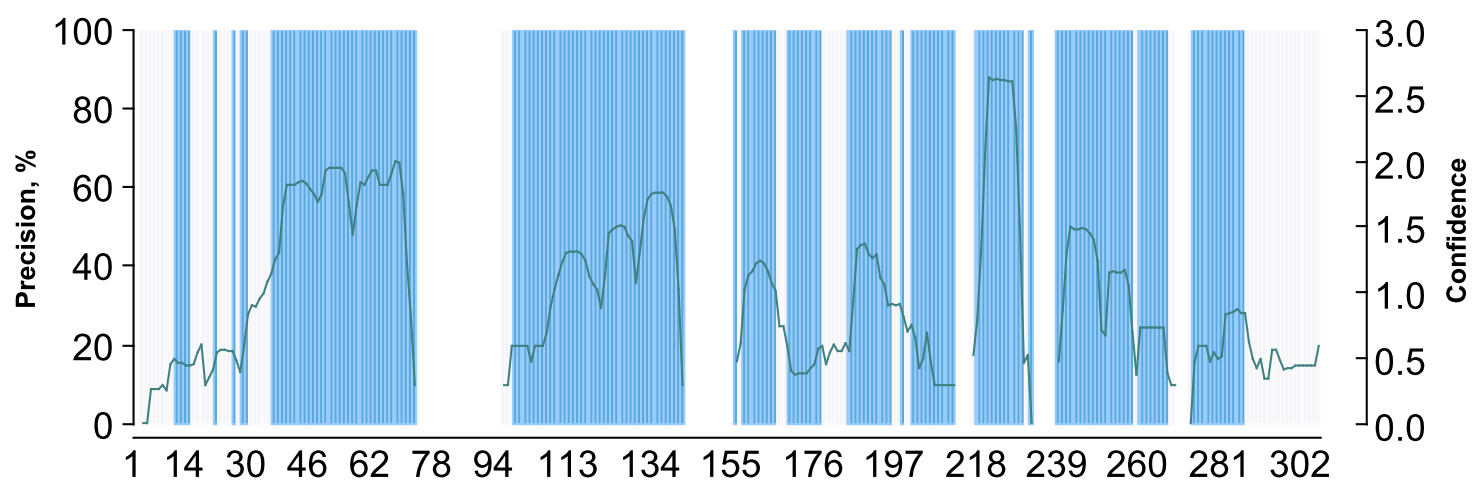

Supplement: Benchmark S1 — Local centroid precision for each target in the benchmark set and a breakdown of the torsion angle prediction performance by residue type and secondary structure. (ZIP) [file pone.0076512.s001.zip › Filtering/3npfA.pdf]

# 210cA

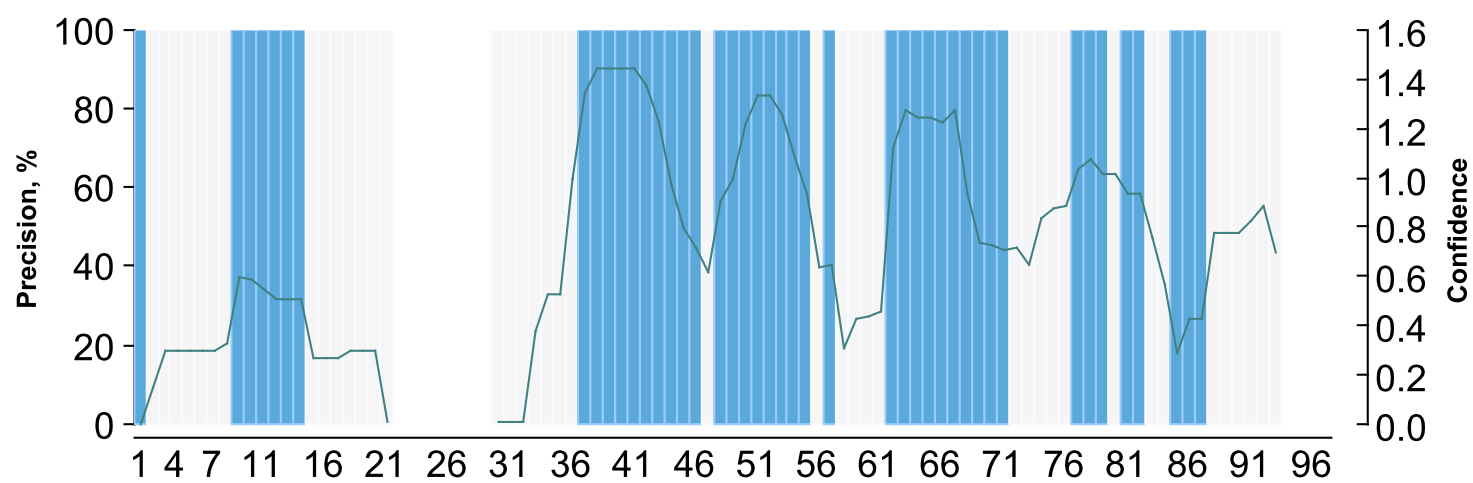

Supplement: Benchmark S1 — Local centroid precision for each target in the benchmark set and a breakdown of the torsion angle prediction performance by residue type and secondary structure. (ZIP) [file pone.0076512.s001.zip › Filtering/2l0cA.pdf]

3on7A

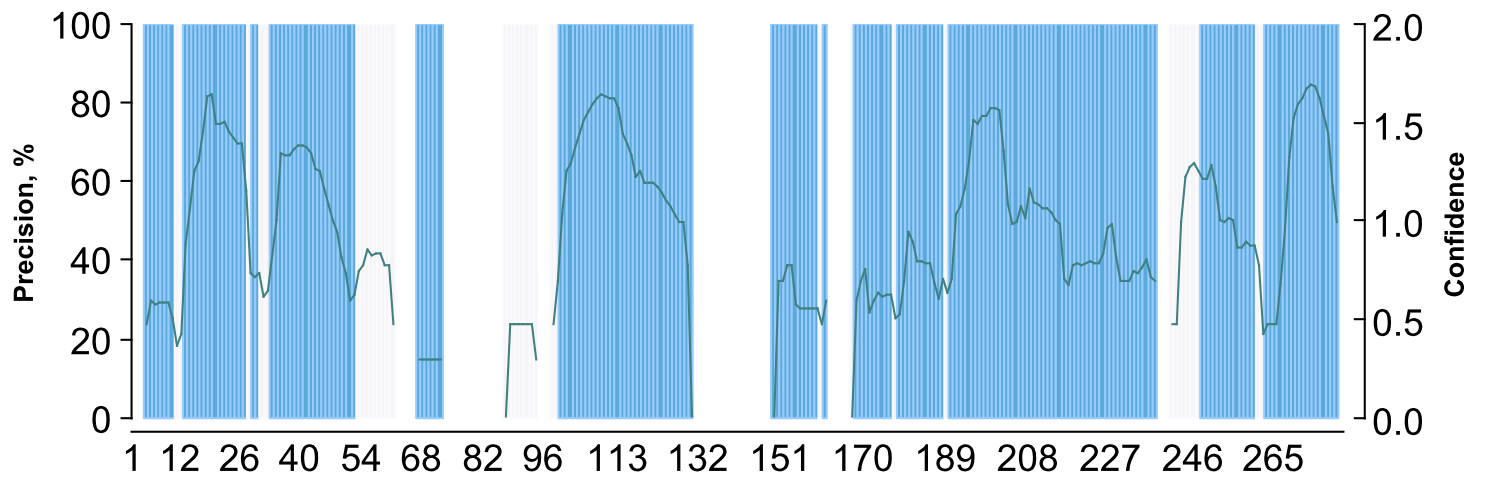

Supplement: Benchmark S1 — Local centroid precision for each target in the benchmark set and a breakdown of the torsion angle prediction performance by residue type and secondary structure. (ZIP) [file pone.0076512.s001.zip › Filtering/3on7A.pdf]

2l01A

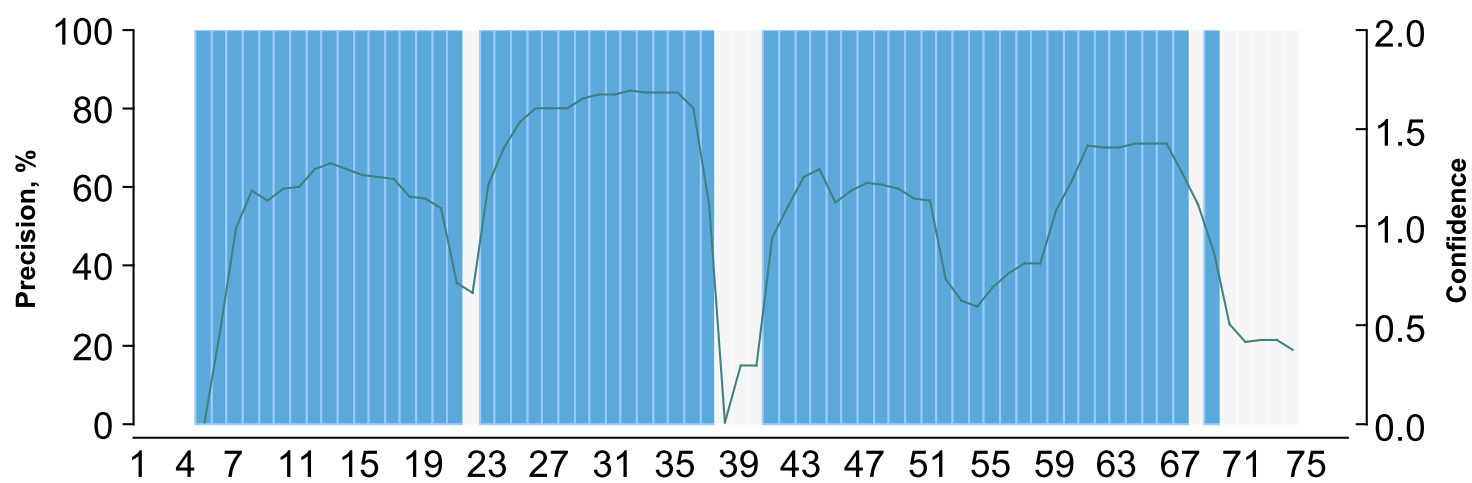

Supplement: Benchmark S1 — Local centroid precision for each target in the benchmark set and a breakdown of the torsion angle prediction performance by residue type and secondary structure. (ZIP) [file pone.0076512.s001.zip › Filtering/2l01A.pdf]

# 3no2A

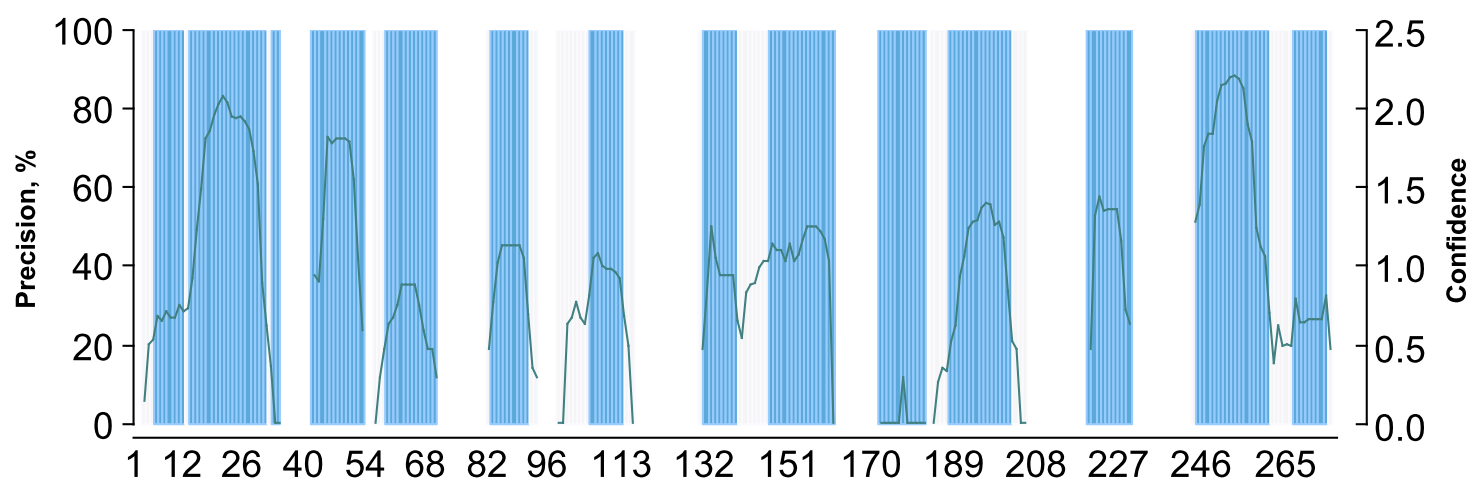

Supplement: Benchmark S1 — Local centroid precision for each target in the benchmark set and a breakdown of the torsion angle prediction performance by residue type and secondary structure. (ZIP) [file pone.0076512.s001.zip › Filtering/3no2A.pdf]

2l3bA

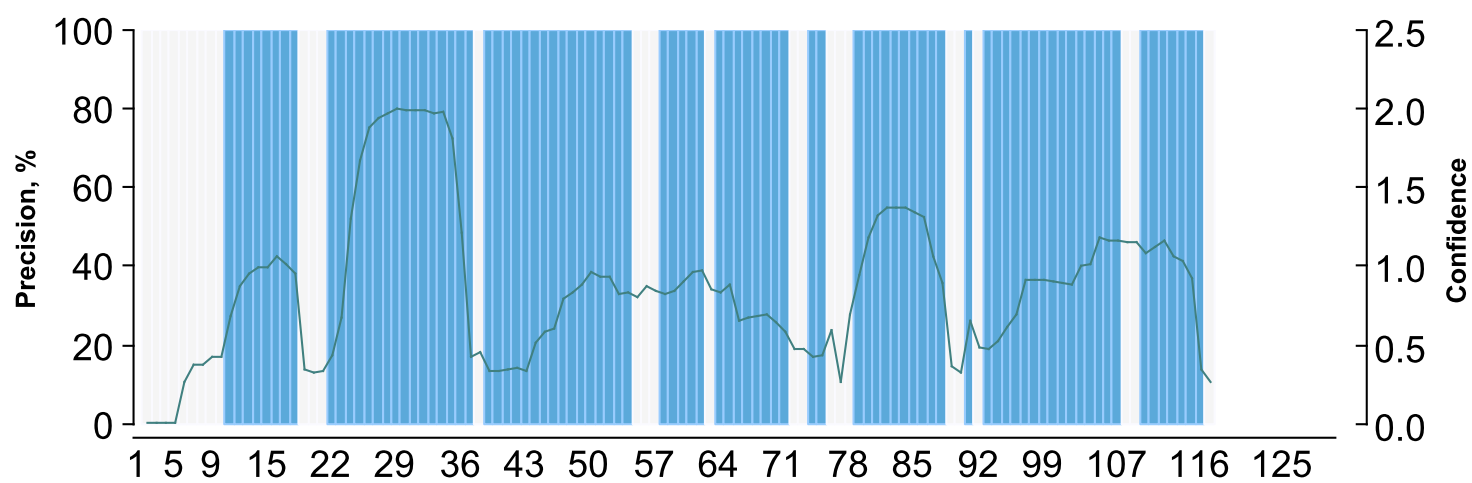

Supplement: Benchmark S1 — Local centroid precision for each target in the benchmark set and a breakdown of the torsion angle prediction performance by residue type and secondary structure. (ZIP) [file pone.0076512.s001.zip › Filtering/2l3bA.pdf]

3obhA

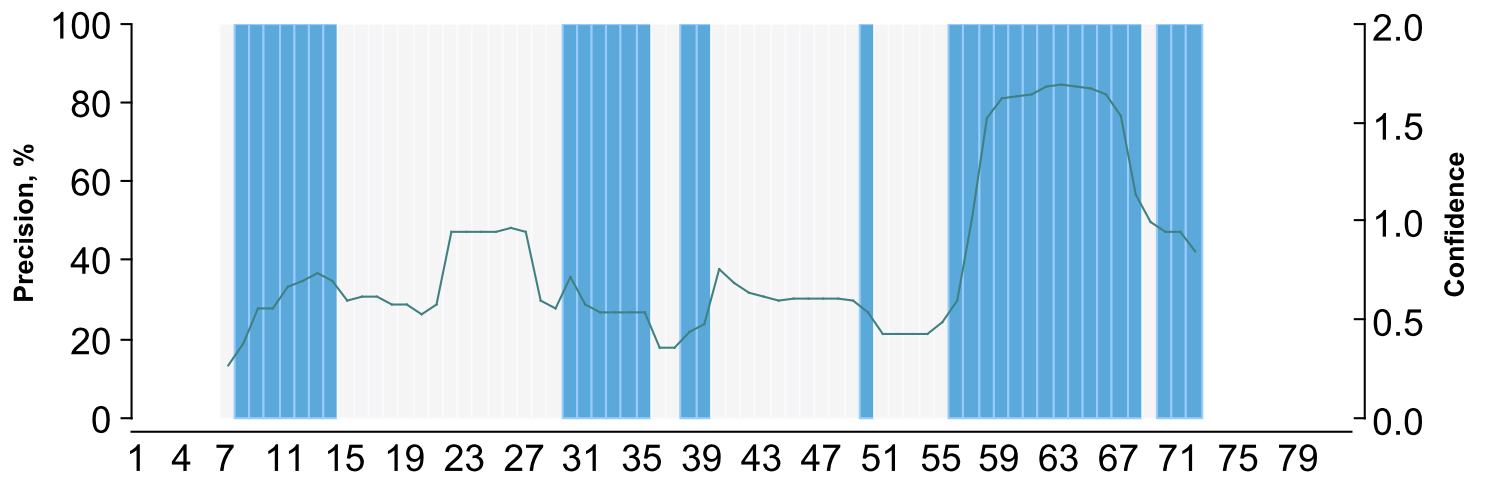

Supplement: Benchmark S1 — Local centroid precision for each target in the benchmark set and a breakdown of the torsion angle prediction performance by residue type and secondary structure. (ZIP) [file pone.0076512.s001.zip › Filtering/3obhA.pdf]

# 3nnqA

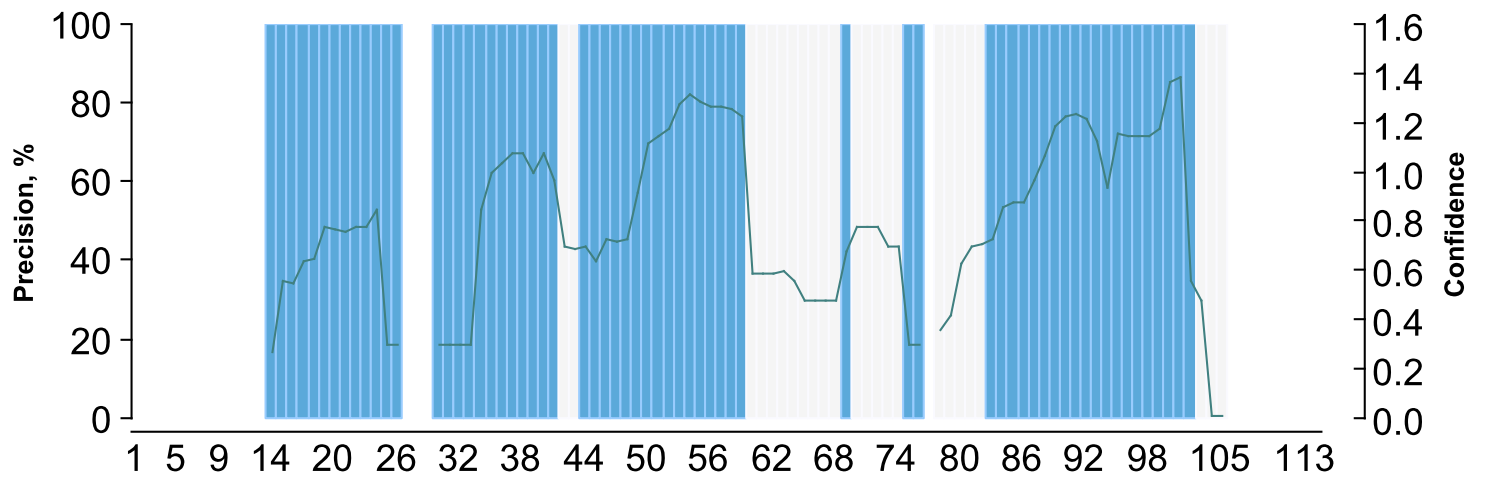

Supplement: Benchmark S1 — Local centroid precision for each target in the benchmark set and a breakdown of the torsion angle prediction performance by residue type and secondary structure. (ZIP) [file pone.0076512.s001.zip › Filtering/3nnqA.pdf]

3nzpA

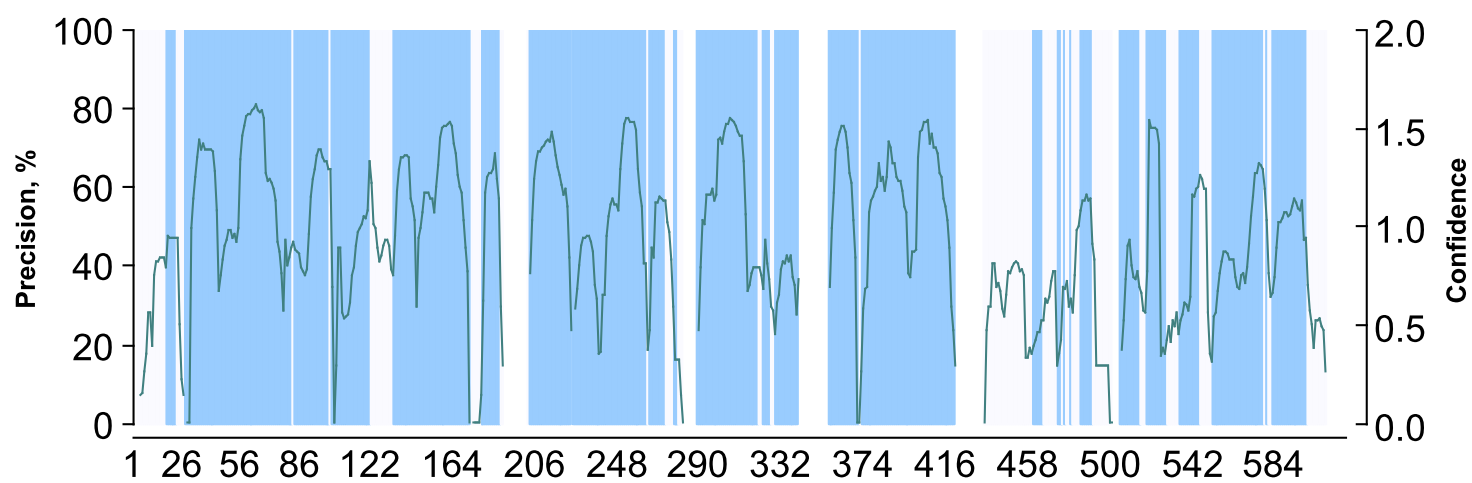

Supplement: Benchmark S1 — Local centroid precision for each target in the benchmark set and a breakdown of the torsion angle prediction performance by residue type and secondary structure. (ZIP) [file pone.0076512.s001.zip › Filtering/3nzpA.pdf]

2l3fA

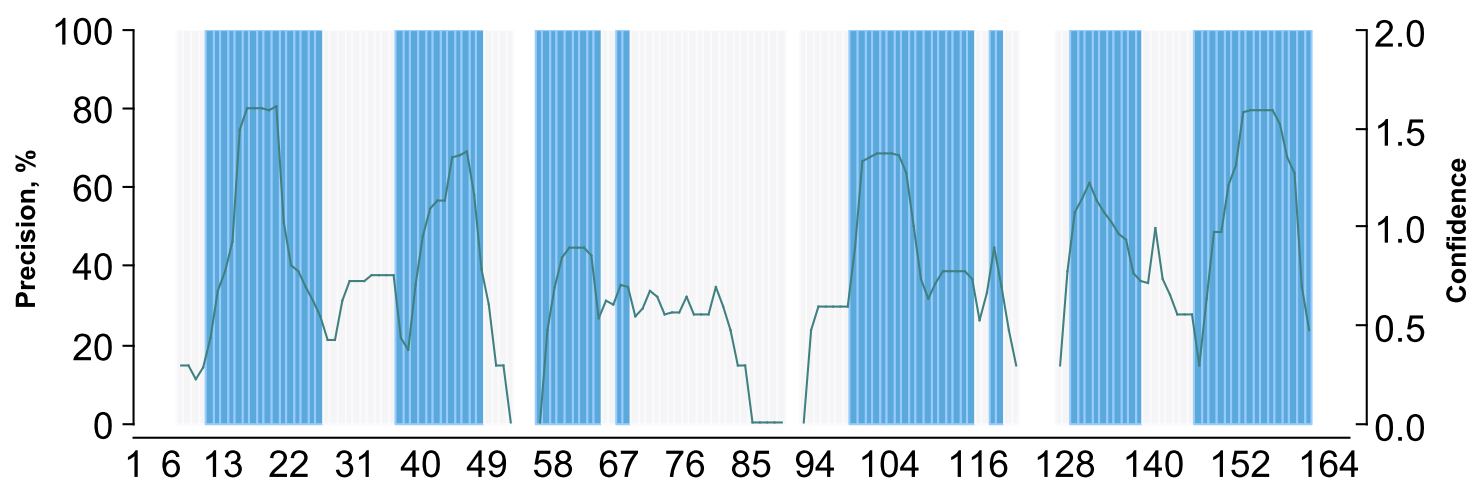

Supplement: Benchmark S1 — Local centroid precision for each target in the benchmark set and a breakdown of the torsion angle prediction performance by residue type and secondary structure. (ZIP) [file pone.0076512.s001.zip › Filtering/2l3fA.pdf]

3n05A

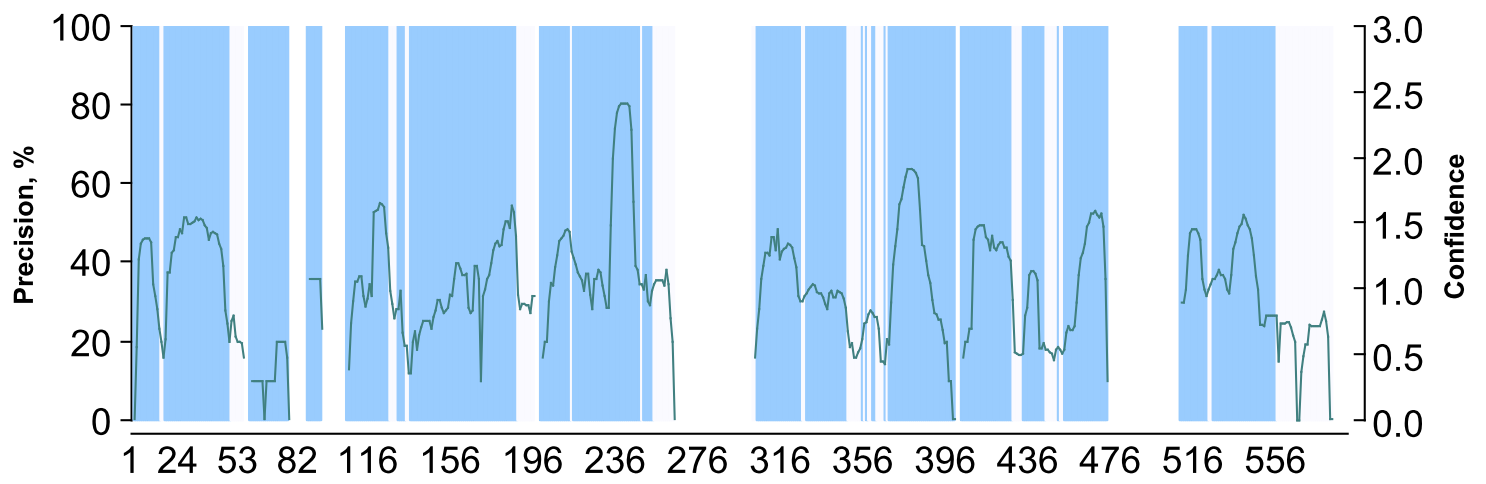

Supplement: Benchmark S1 — Local centroid precision for each target in the benchmark set and a breakdown of the torsion angle prediction performance by residue type and secondary structure. (ZIP) [file pone.0076512.s001.zip › Filtering/3n05A.pdf]

2kzxA

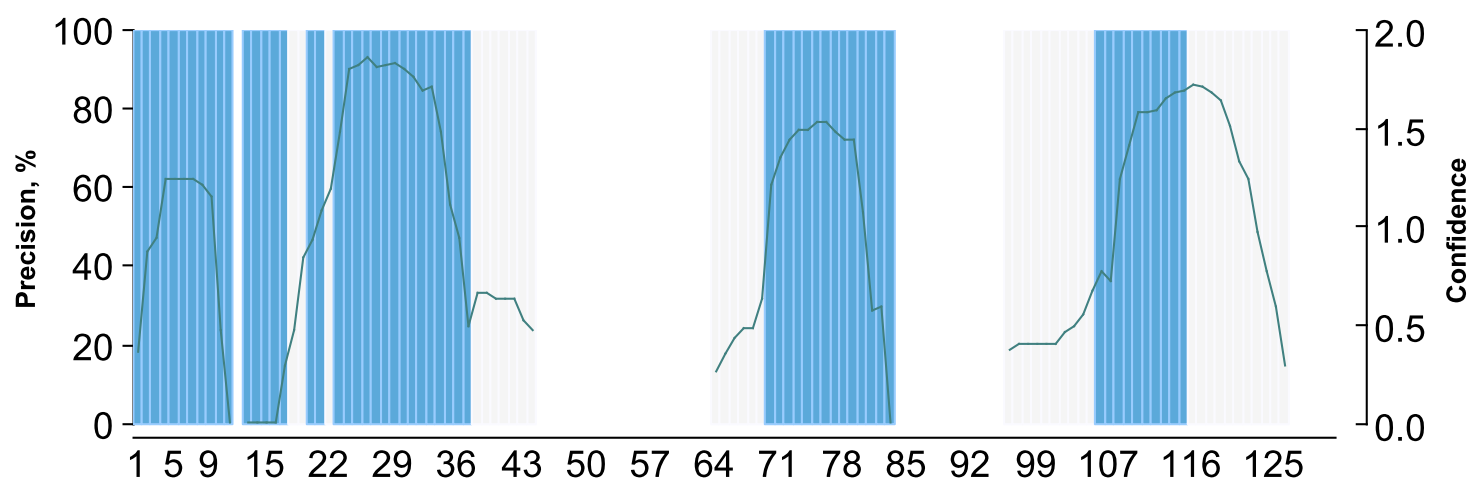

Supplement: Benchmark S1 — Local centroid precision for each target in the benchmark set and a breakdown of the torsion angle prediction performance by residue type and secondary structure. (ZIP) [file pone.0076512.s001.zip › Filtering/2kzxA.pdf]

210dA

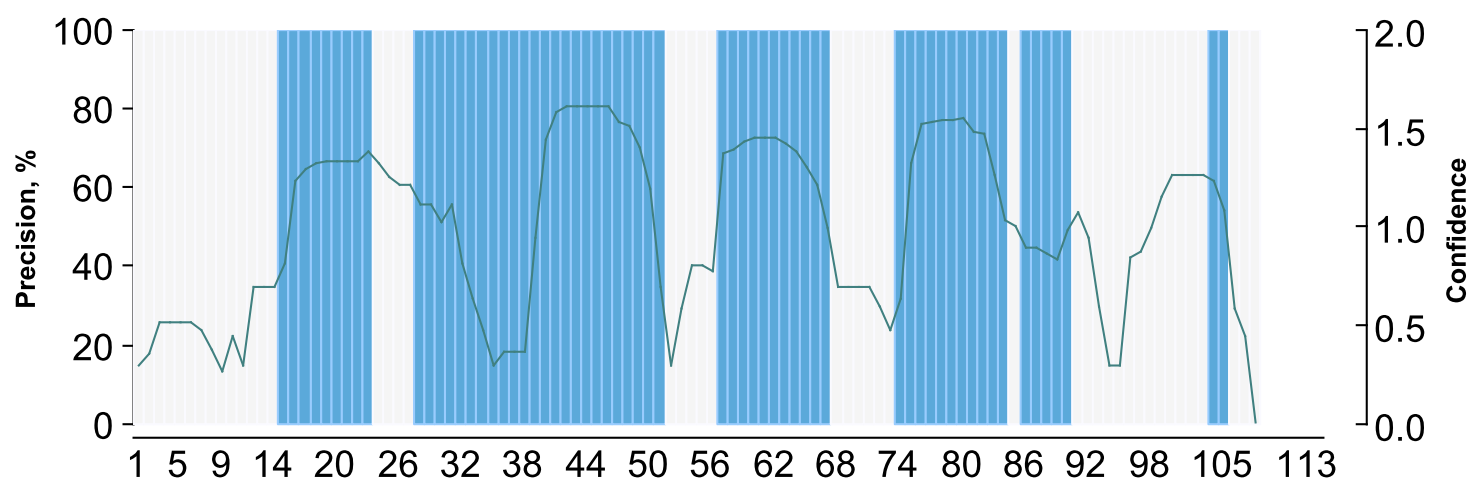

Supplement: Benchmark S1 — Local centroid precision for each target in the benchmark set and a breakdown of the torsion angle prediction performance by residue type and secondary structure. (ZIP) [file pone.0076512.s001.zip › Filtering/2l0dA.pdf]

2l0bA

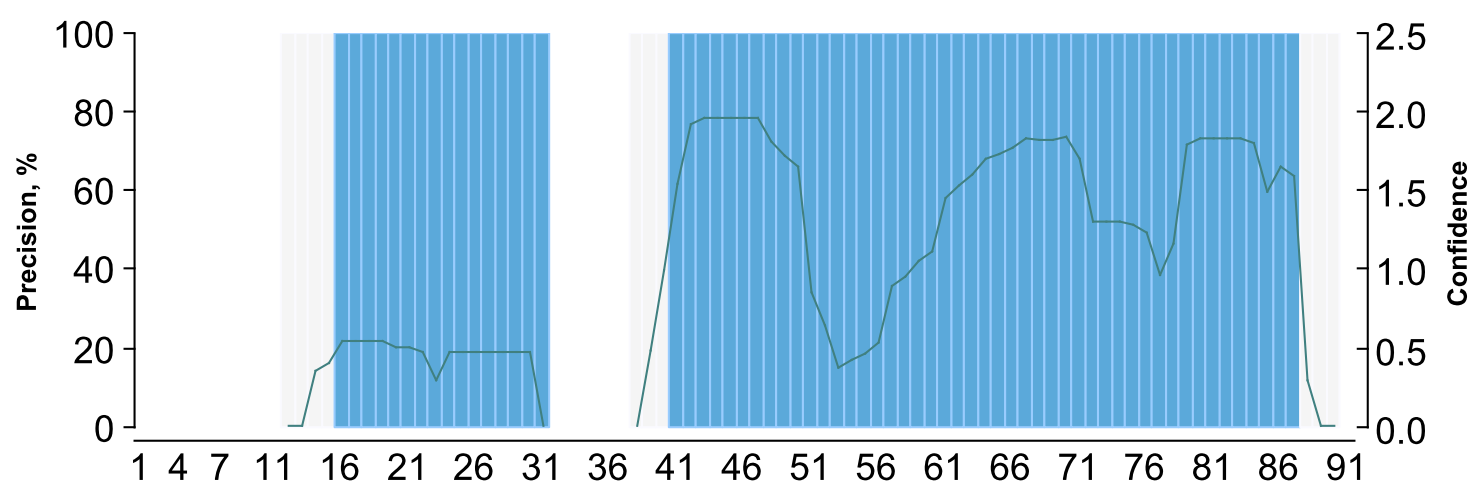

Supplement: Benchmark S1 — Local centroid precision for each target in the benchmark set and a breakdown of the torsion angle prediction performance by residue type and secondary structure. (ZIP) [file pone.0076512.s001.zip › Filtering/2l0bA.pdf]

2109A

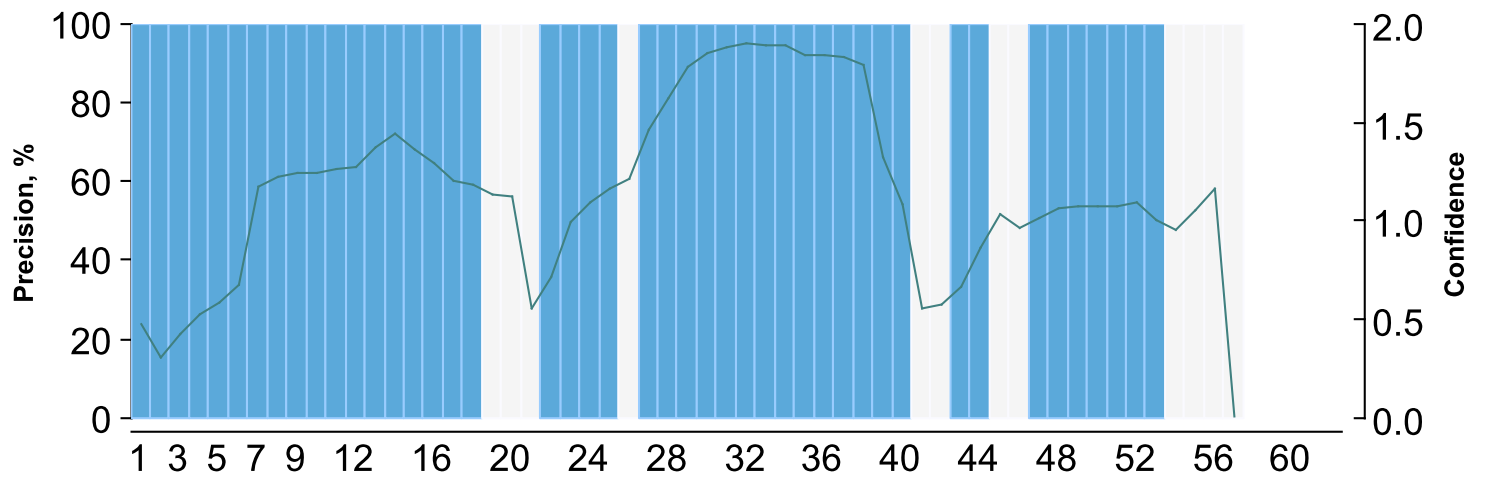

Supplement: Benchmark S1 — Local centroid precision for each target in the benchmark set and a breakdown of the torsion angle prediction performance by residue type and secondary structure. (ZIP) [file pone.0076512.s001.zip › Filtering/2l09A.pdf]

3mxqA

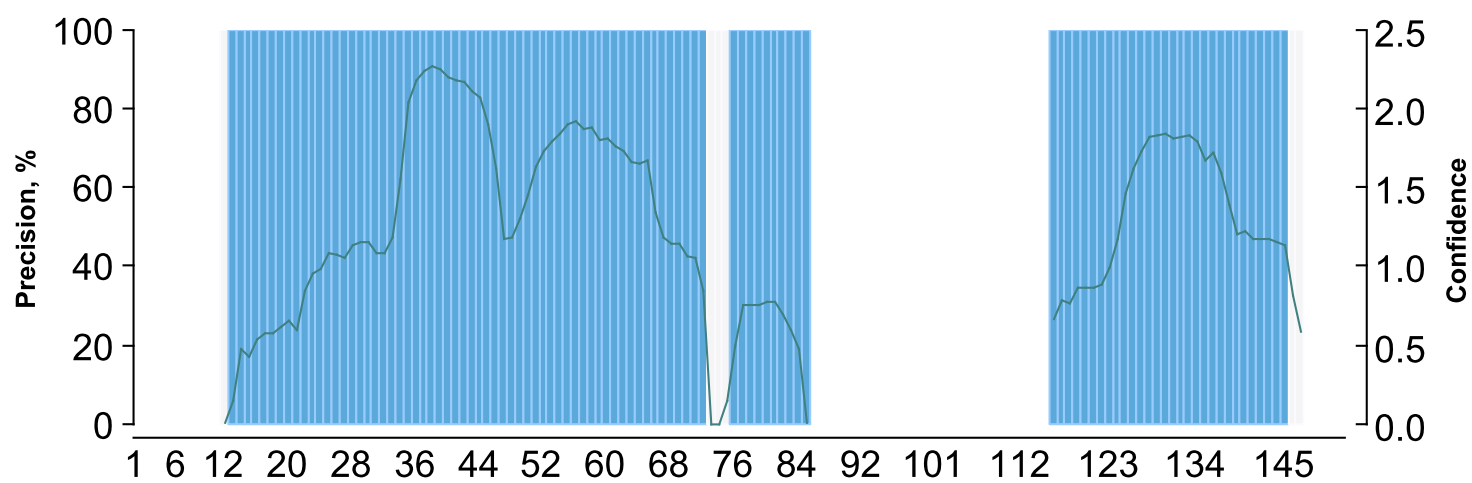

Supplement: Benchmark S1 — Local centroid precision for each target in the benchmark set and a breakdown of the torsion angle prediction performance by residue type and secondary structure. (ZIP) [file pone.0076512.s001.zip › Filtering/3mxqA.pdf]

# 3mwbA

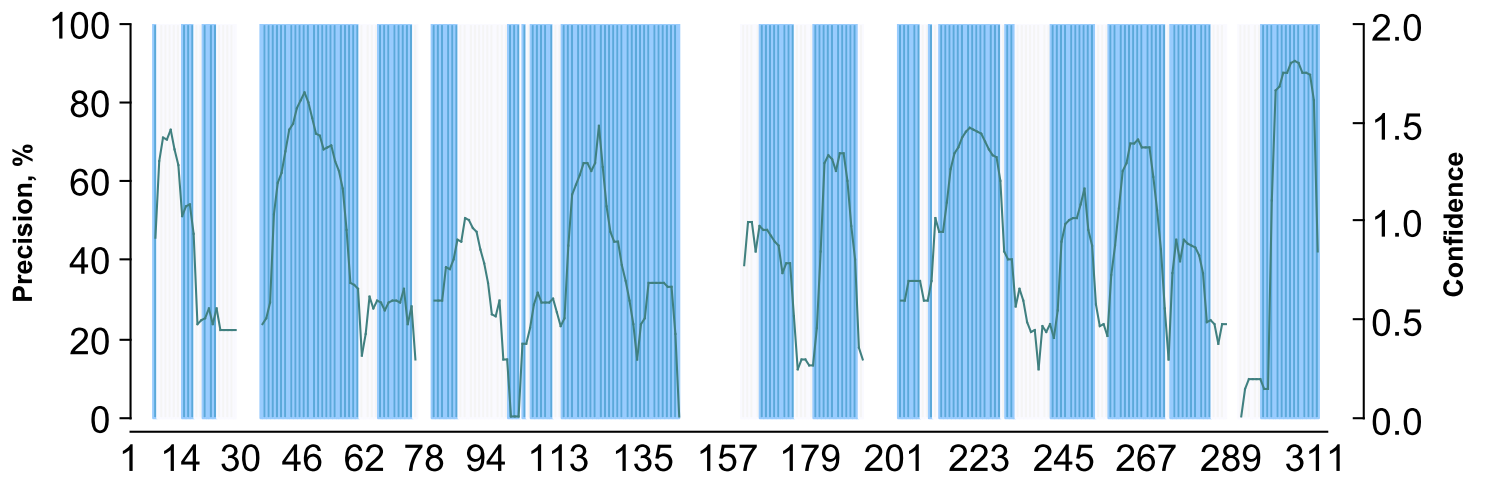

Supplement: Benchmark S1 — Local centroid precision for each target in the benchmark set and a breakdown of the torsion angle prediction performance by residue type and secondary structure. (ZIP) [file pone.0076512.s001.zip › Filtering/3mwbA.pdf]

3mx3A

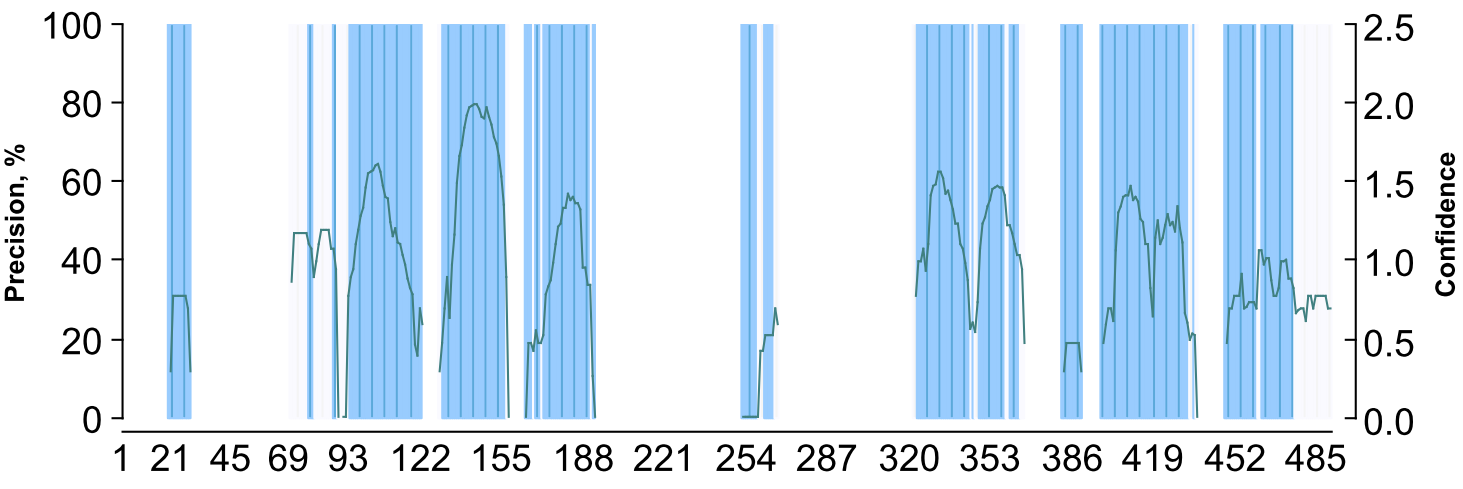

Supplement: Benchmark S1 — Local centroid precision for each target in the benchmark set and a breakdown of the torsion angle prediction performance by residue type and secondary structure. (ZIP) [file pone.0076512.s001.zip › Filtering/3mx3A.pdf]

3n0xA

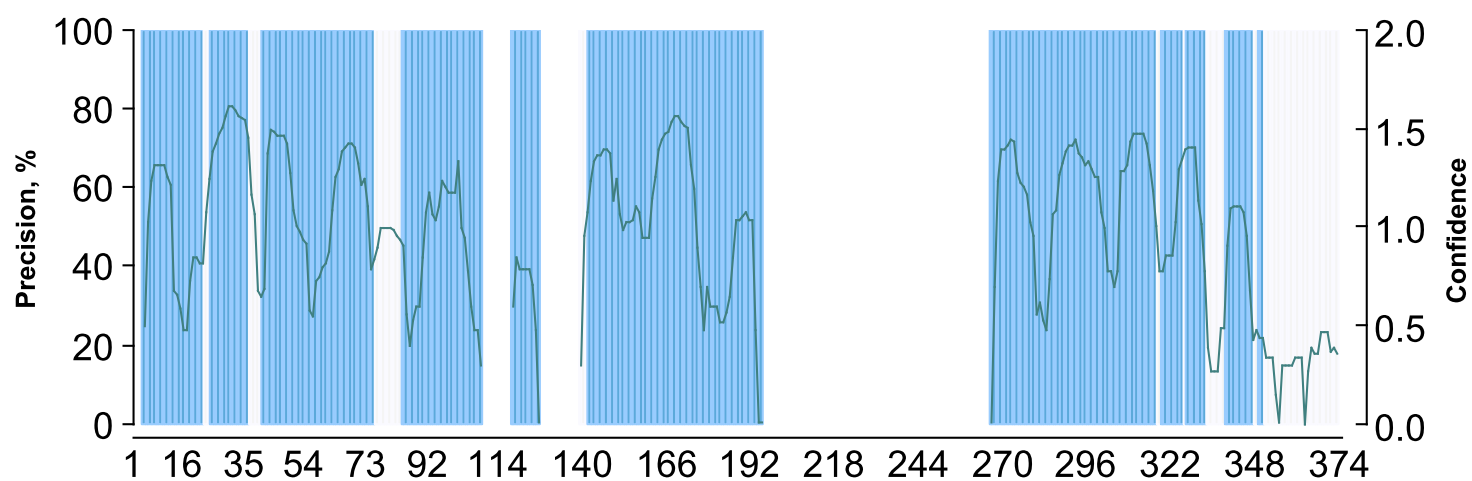

Supplement: Benchmark S1 — Local centroid precision for each target in the benchmark set and a breakdown of the torsion angle prediction performance by residue type and secondary structure. (ZIP) [file pone.0076512.s001.zip › Filtering/3n0xA.pdf]

3mr0A

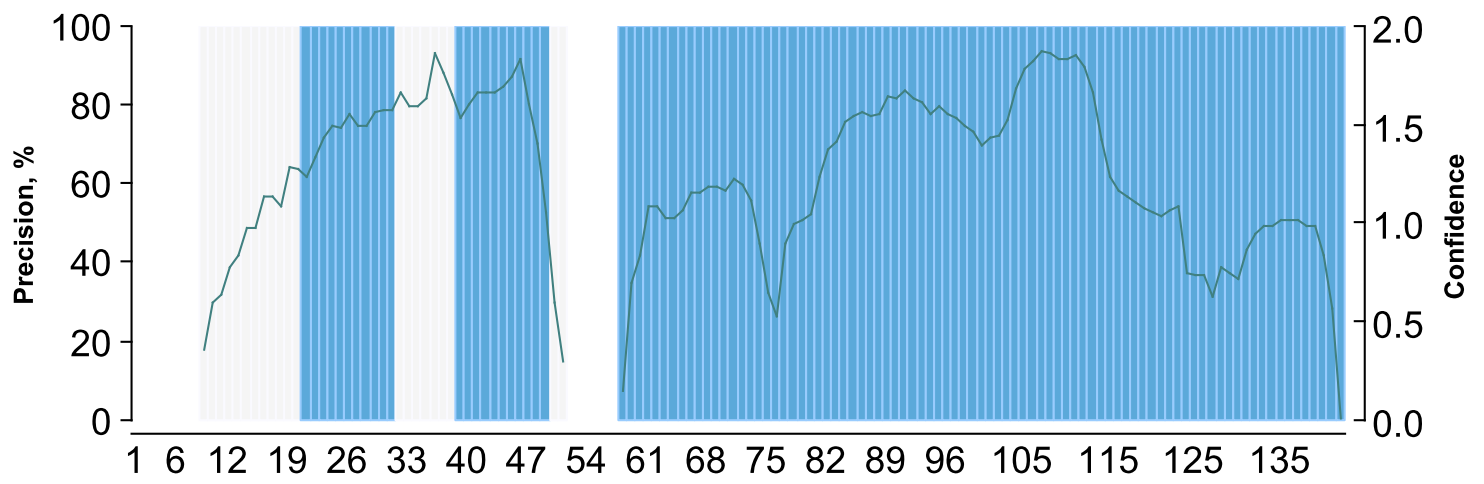

Supplement: Benchmark S1 — Local centroid precision for each target in the benchmark set and a breakdown of the torsion angle prediction performance by residue type and secondary structure. (ZIP) [file pone.0076512.s001.zip › Filtering/3mr0A.pdf]

# 3nreA

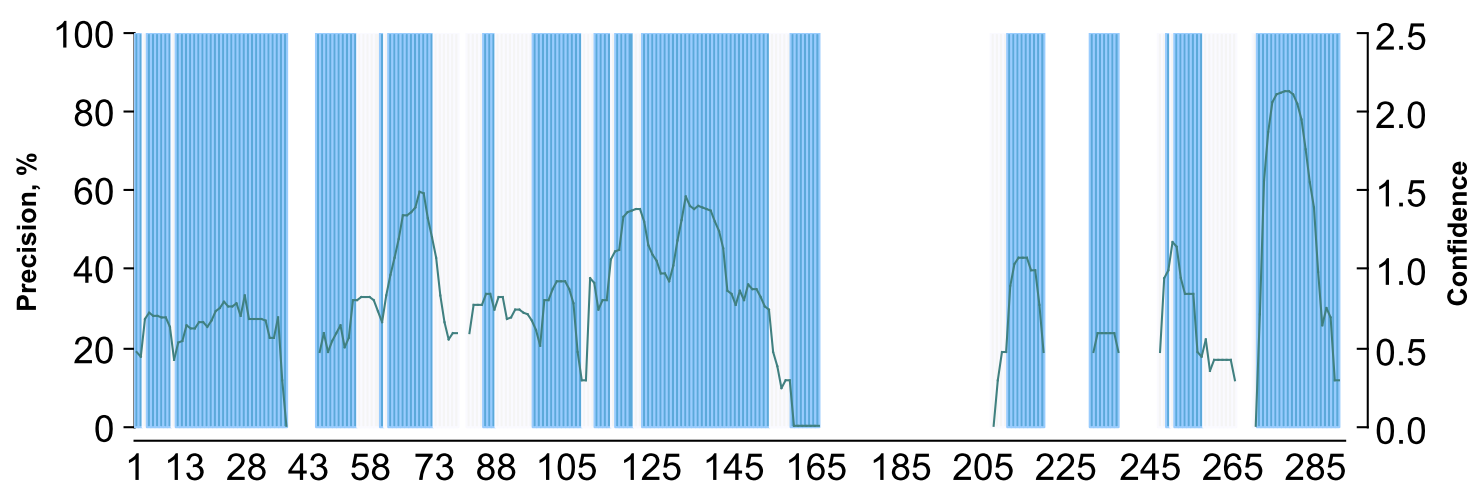

Supplement: Benchmark S1 — Local centroid precision for each target in the benchmark set and a breakdown of the torsion angle prediction performance by residue type and secondary structure. (ZIP) [file pone.0076512.s001.zip › Filtering/3nreA.pdf]

2102A

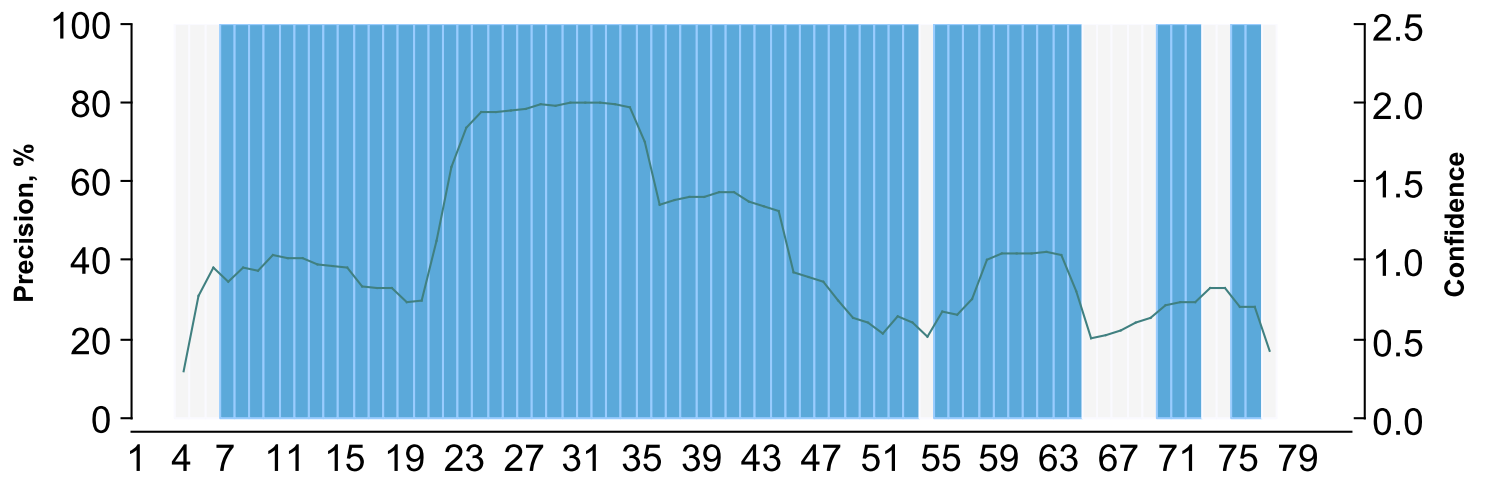

Supplement: Benchmark S1 — Local centroid precision for each target in the benchmark set and a breakdown of the torsion angle prediction performance by residue type and secondary structure. (ZIP) [file pone.0076512.s001.zip › Filtering/2l02A.pdf]

3mqzA

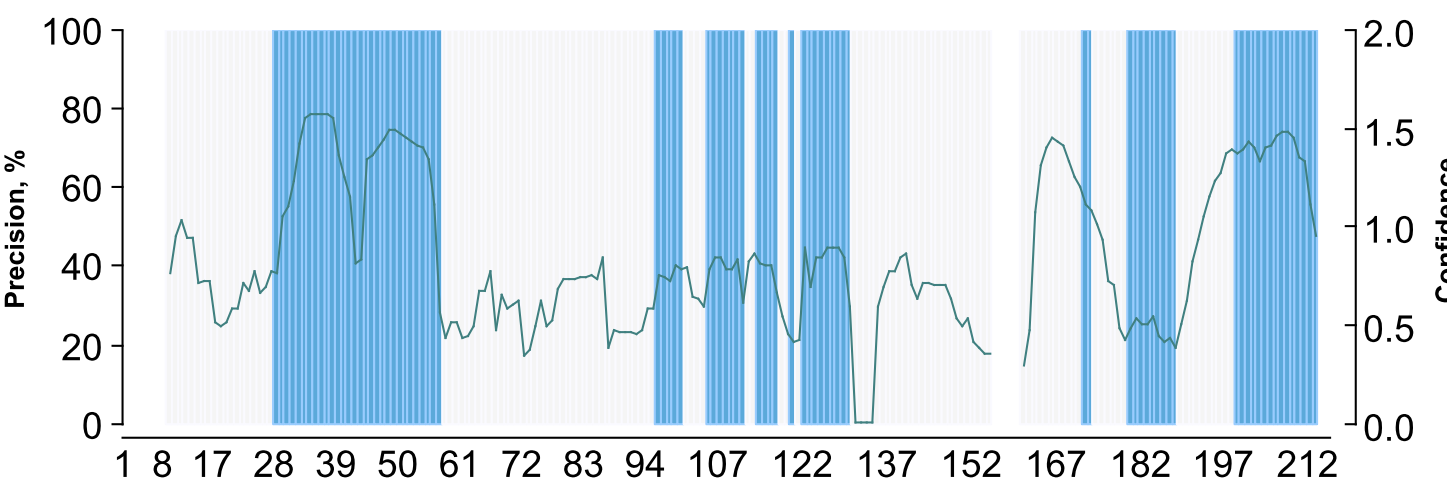

Supplement: Benchmark S1 — Local centroid precision for each target in the benchmark set and a breakdown of the torsion angle prediction performance by residue type and secondary structure. (ZIP) [file pone.0076512.s001.zip › Filtering/3mqzA.pdf]

# 3mwxA

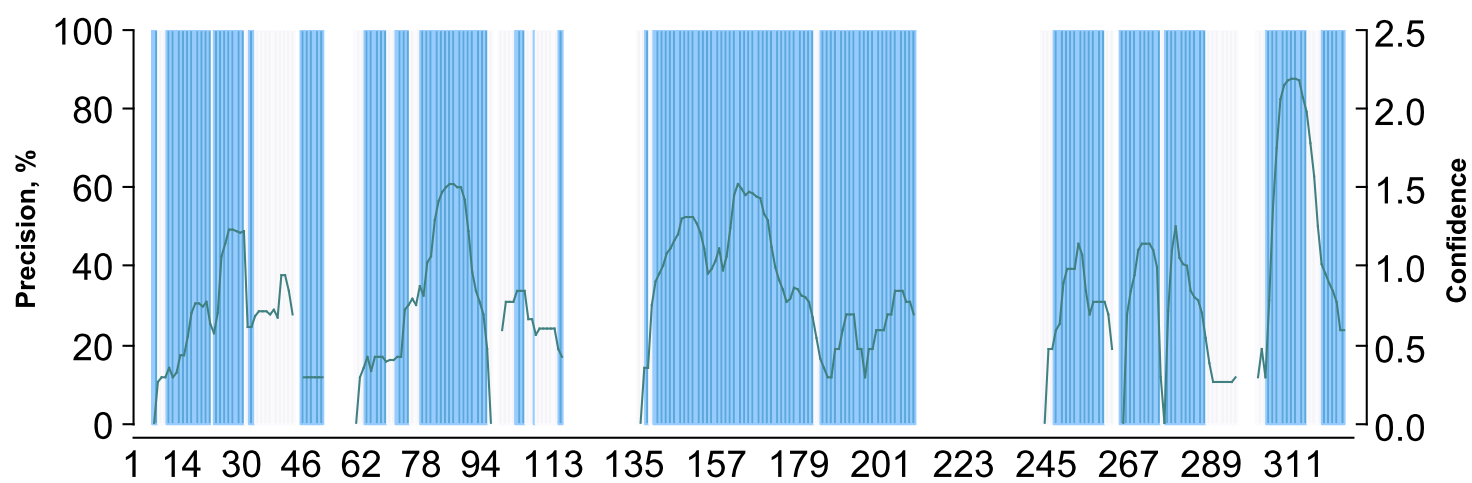

Supplement: Benchmark S1 — Local centroid precision for each target in the benchmark set and a breakdown of the torsion angle prediction performance by residue type and secondary structure. (ZIP) [file pone.0076512.s001.zip › Filtering/3mwxA.pdf]

3mqoA

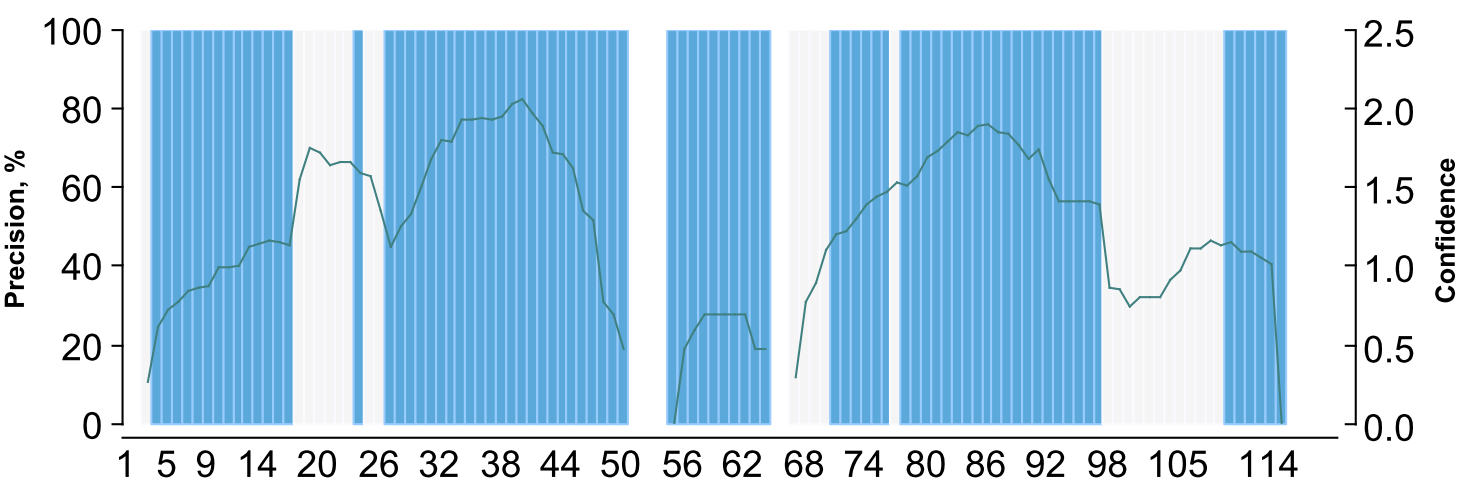

Supplement: Benchmark S1 — Local centroid precision for each target in the benchmark set and a breakdown of the torsion angle prediction performance by residue type and secondary structure. (ZIP) [file pone.0076512.s001.zip › Filtering/3mqoA.pdf]

### 3nrdA

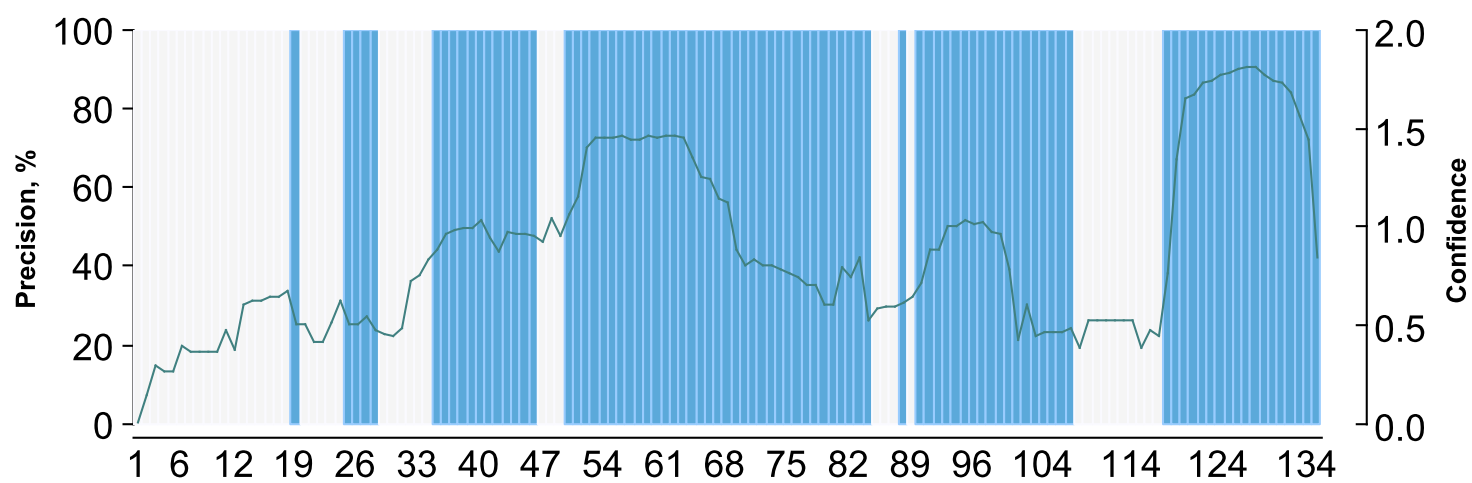

Supplement: Benchmark S1 — Local centroid precision for each target in the benchmark set and a breakdown of the torsion angle prediction performance by residue type and secondary structure. (ZIP) [file pone.0076512.s001.zip › Filtering/3nrdA.pdf]

3mseB

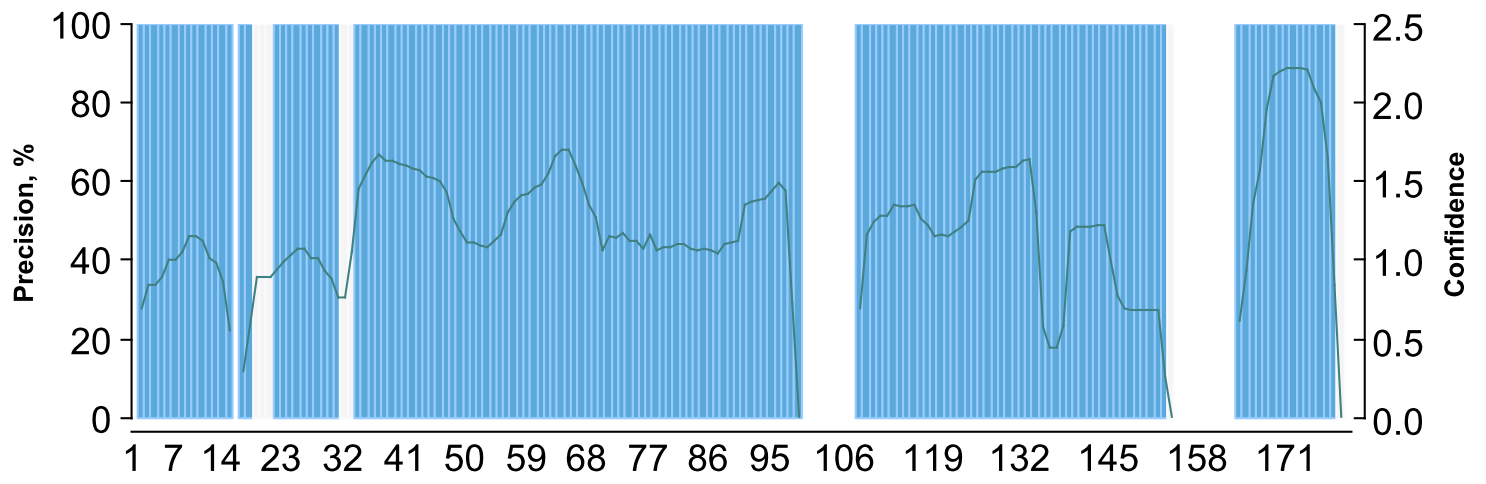

Supplement: Benchmark S1 — Local centroid precision for each target in the benchmark set and a breakdown of the torsion angle prediction performance by residue type and secondary structure. (ZIP) [file pone.0076512.s001.zip › Filtering/3mseB.pdf]

# 3mr7A

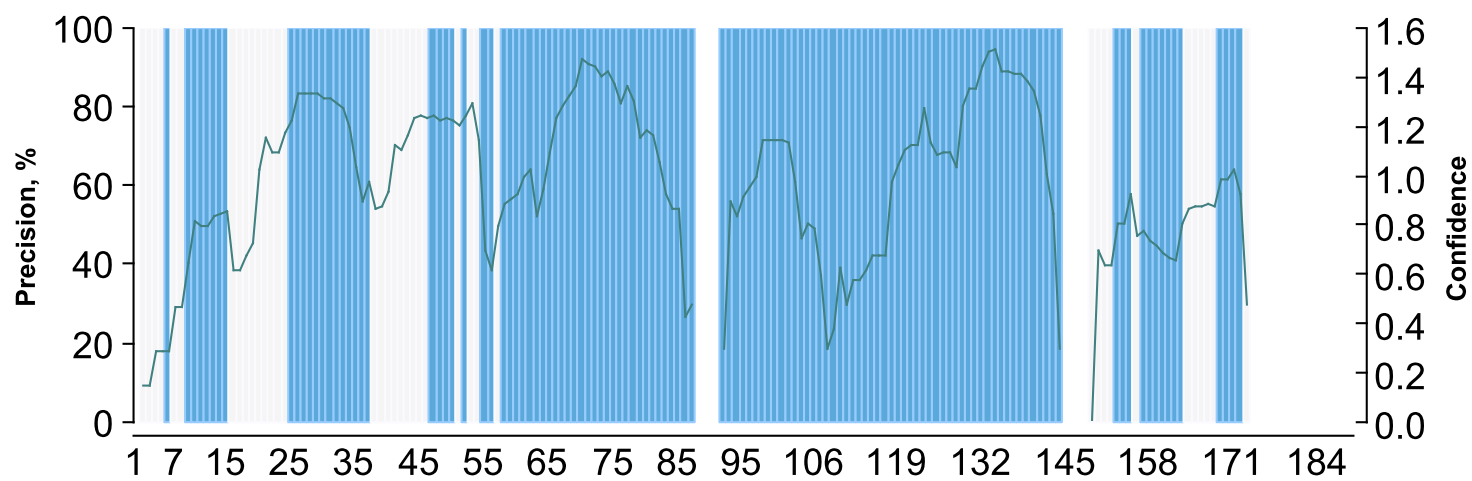

Supplement: Benchmark S1 — Local centroid precision for each target in the benchmark set and a breakdown of the torsion angle prediction performance by residue type and secondary structure. (ZIP) [file pone.0076512.s001.zip › Filtering/3mr7A.pdf]

3nmbA

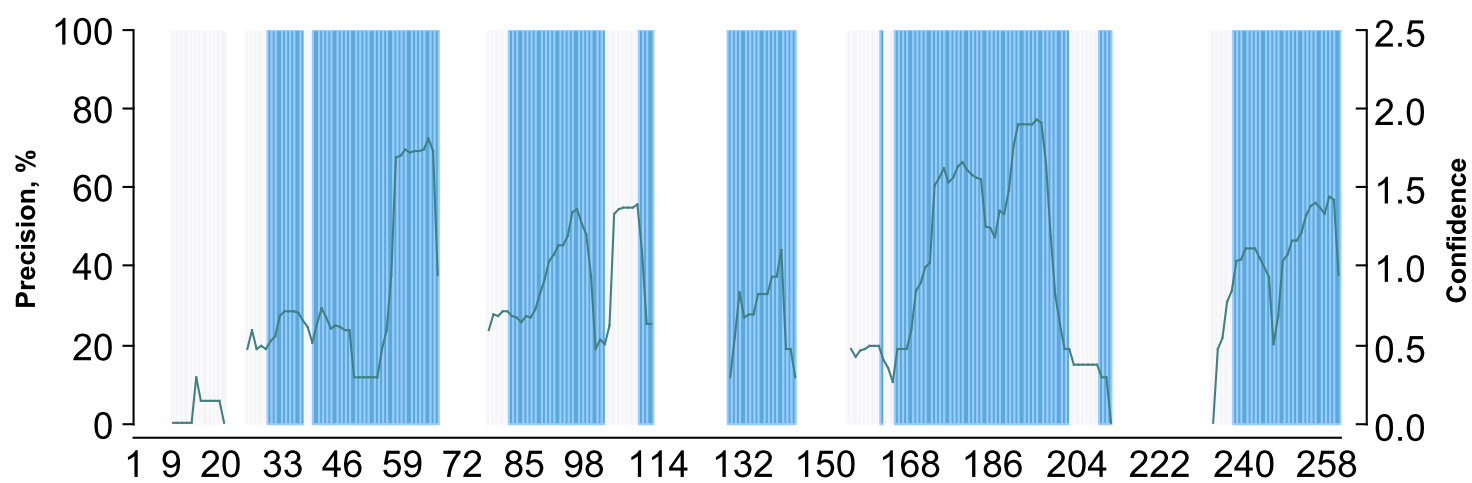

Supplement: Benchmark S1 — Local centroid precision for each target in the benchmark set and a breakdown of the torsion angle prediction performance by residue type and secondary structure. (ZIP) [file pone.0076512.s001.zip › Filtering/3nmbA.pdf]

# 3no6A

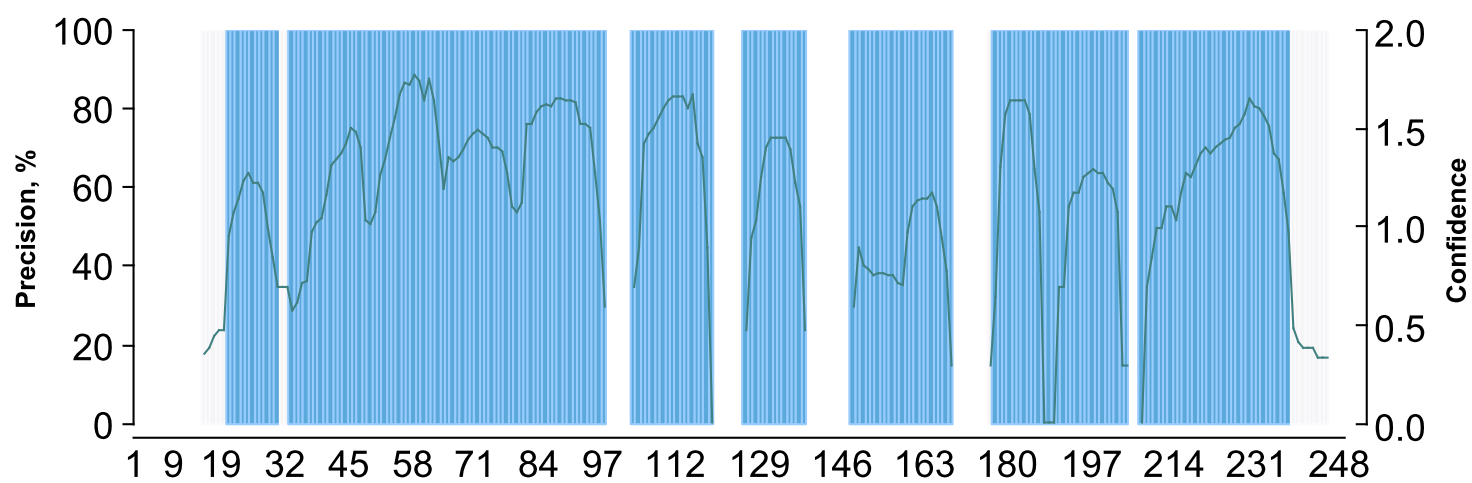

Supplement: Benchmark S1 — Local centroid precision for each target in the benchmark set and a breakdown of the torsion angle prediction performance by residue type and secondary structure. (ZIP) [file pone.0076512.s001.zip › Filtering/3no6A.pdf]

# 3mt1A

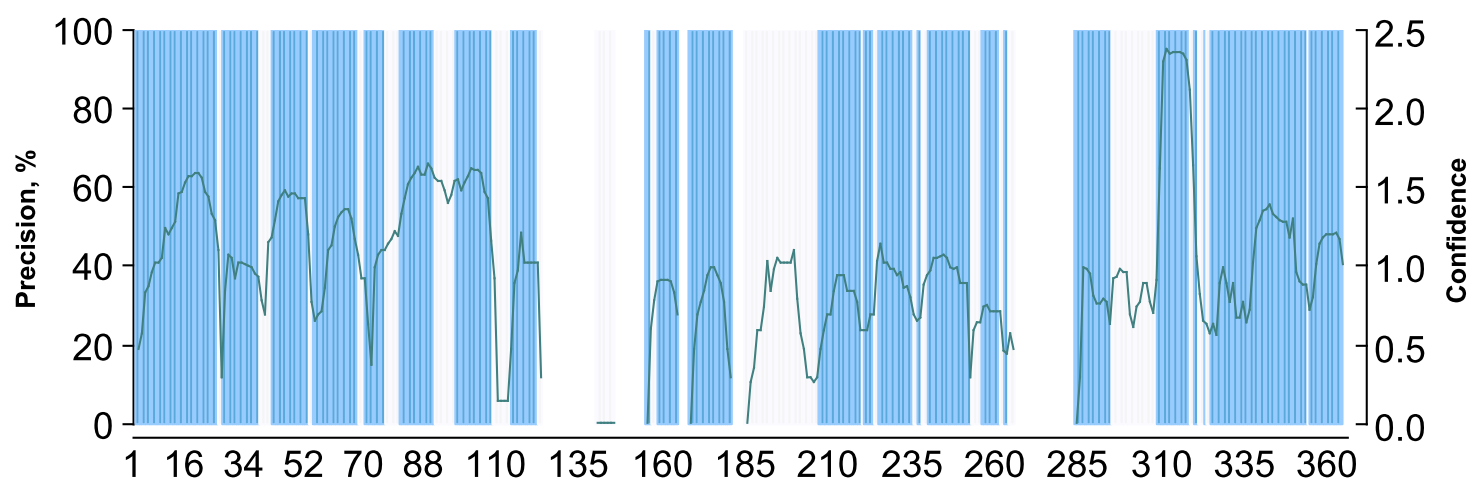

Supplement: Benchmark S1 — Local centroid precision for each target in the benchmark set and a breakdown of the torsion angle prediction performance by residue type and secondary structure. (ZIP) [file pone.0076512.s001.zip › Filtering/3mt1A.pdf]

2kyyA

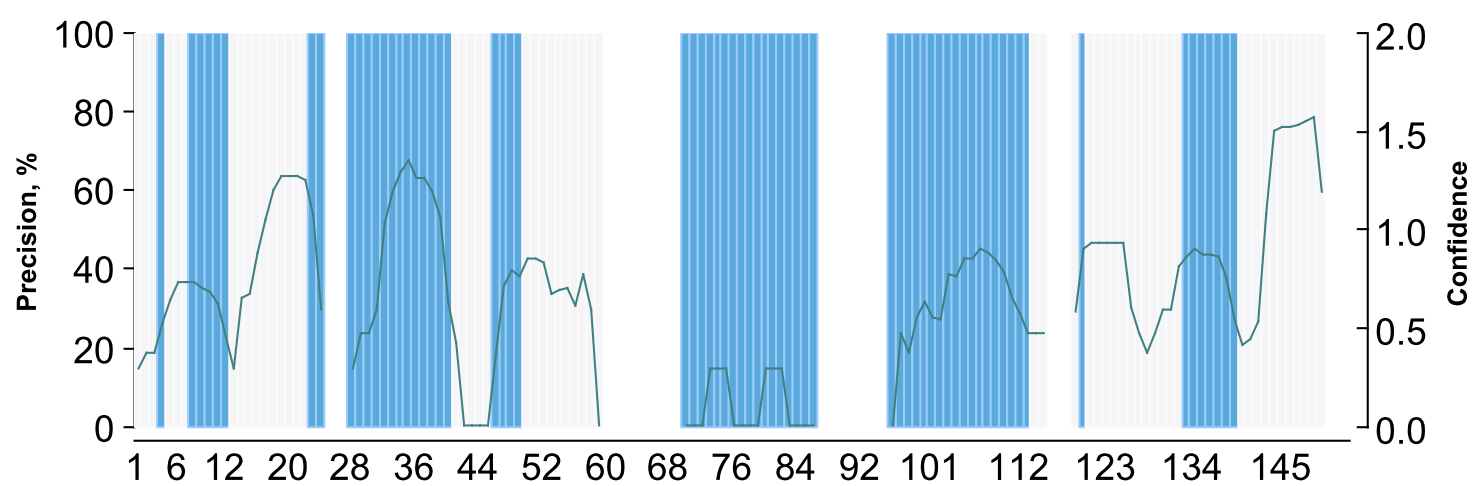

Supplement: Benchmark S1 — Local centroid precision for each target in the benchmark set and a breakdown of the torsion angle prediction performance by residue type and secondary structure. (ZIP) [file pone.0076512.s001.zip › Filtering/2kyyA.pdf]

# 3nrtA

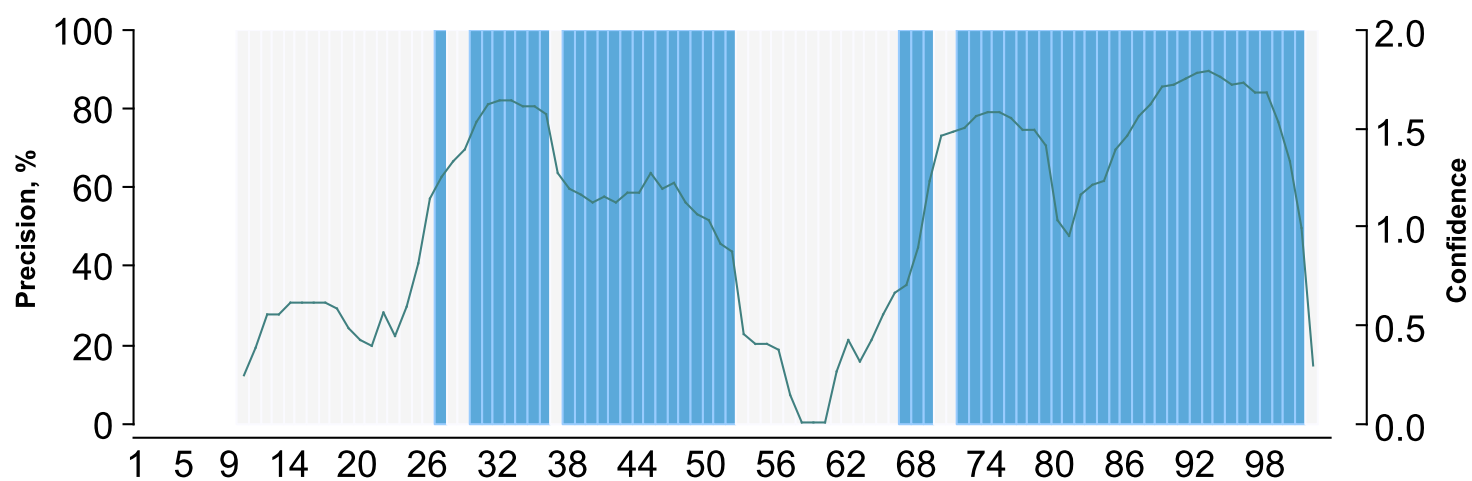

Supplement: Benchmark S1 — Local centroid precision for each target in the benchmark set and a breakdown of the torsion angle prediction performance by residue type and secondary structure. (ZIP) [file pone.0076512.s001.zip › Filtering/3nrtA.pdf]

# 3n91A

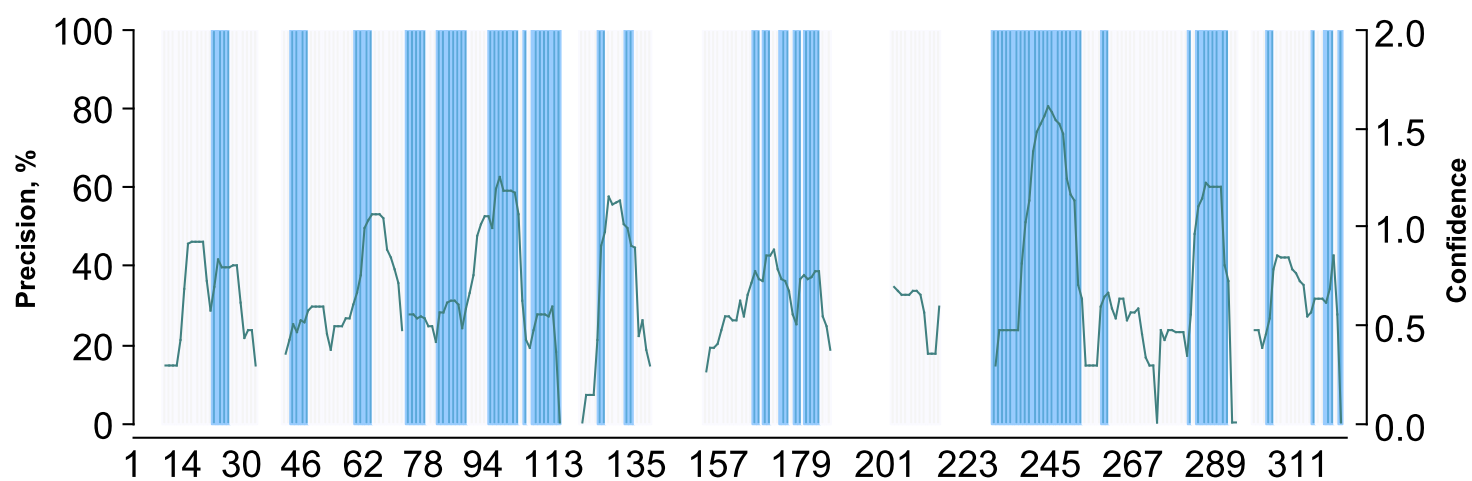

Supplement: Benchmark S1 — Local centroid precision for each target in the benchmark set and a breakdown of the torsion angle prediction performance by residue type and secondary structure. (ZIP) [file pone.0076512.s001.zip › Filtering/3n91A.pdf]

3nqkA

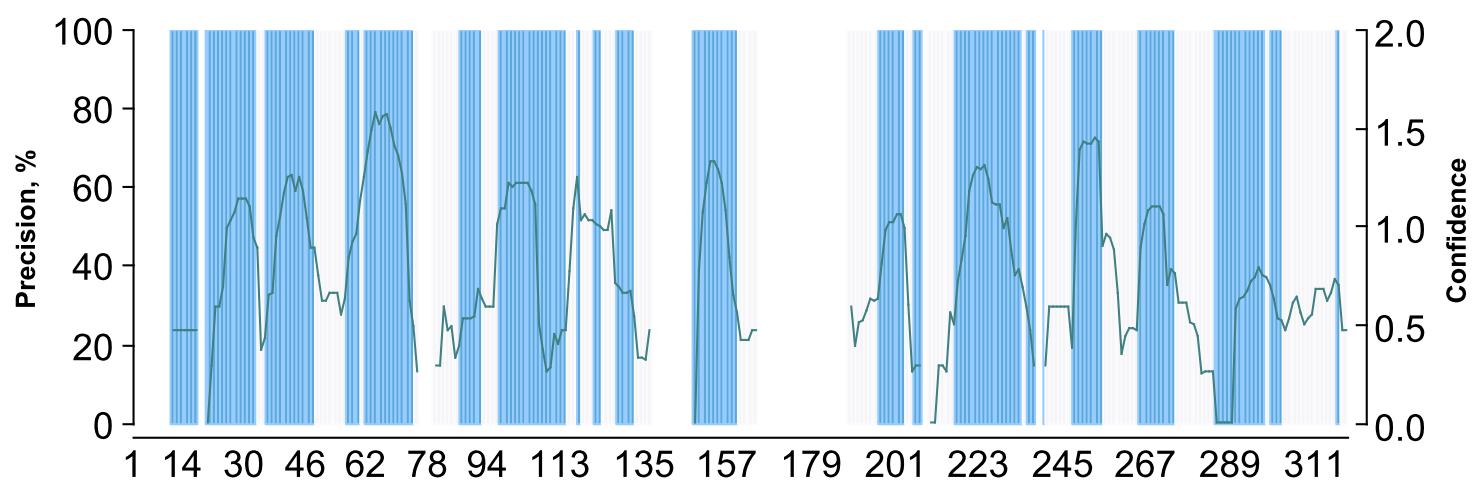

Supplement: Benchmark S1 — Local centroid precision for each target in the benchmark set and a breakdown of the torsion angle prediction performance by residue type and secondary structure. (ZIP) [file pone.0076512.s001.zip › Filtering/3nqkA.pdf]

3nkgA

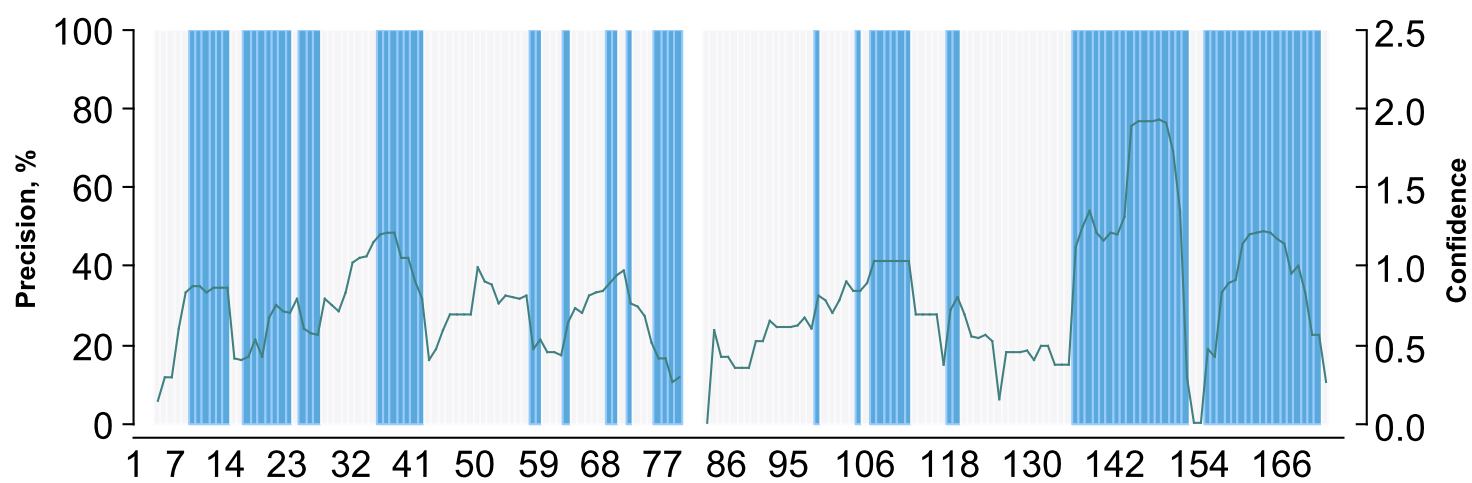

Supplement: Benchmark S1 — Local centroid precision for each target in the benchmark set and a breakdown of the torsion angle prediction performance by residue type and secondary structure. (ZIP) [file pone.0076512.s001.zip › Filtering/3nkgA.pdf]

### 3nrhA

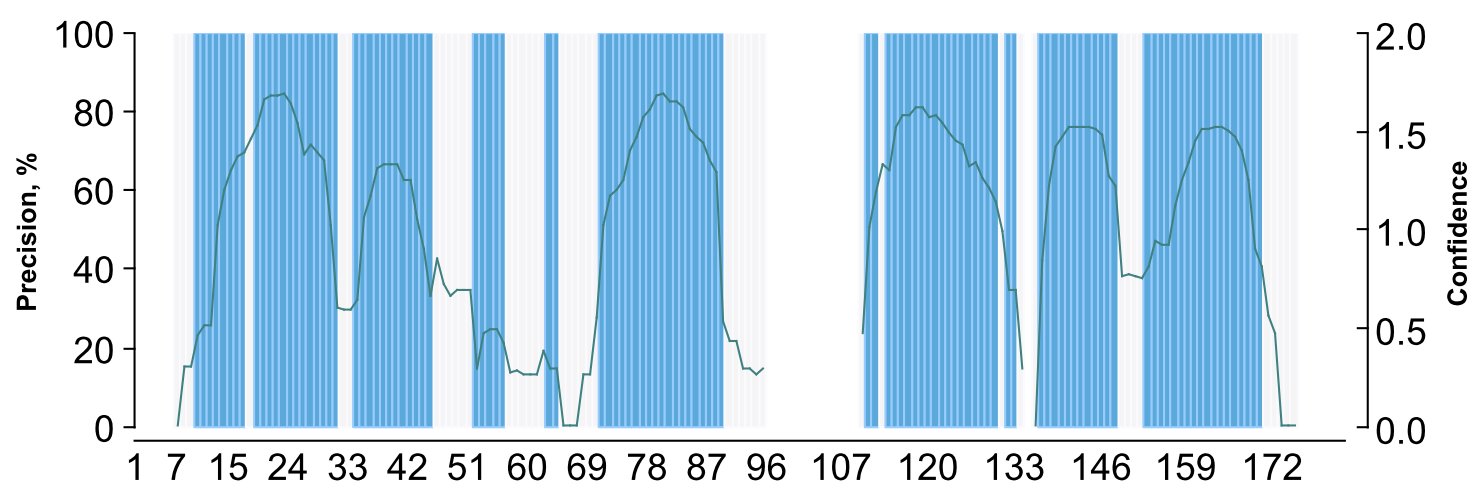

Supplement: Benchmark S1 — Local centroid precision for each target in the benchmark set and a breakdown of the torsion angle prediction performance by residue type and secondary structure. (ZIP) [file pone.0076512.s001.zip › Filtering/3nrhA.pdf]

### 3npdA

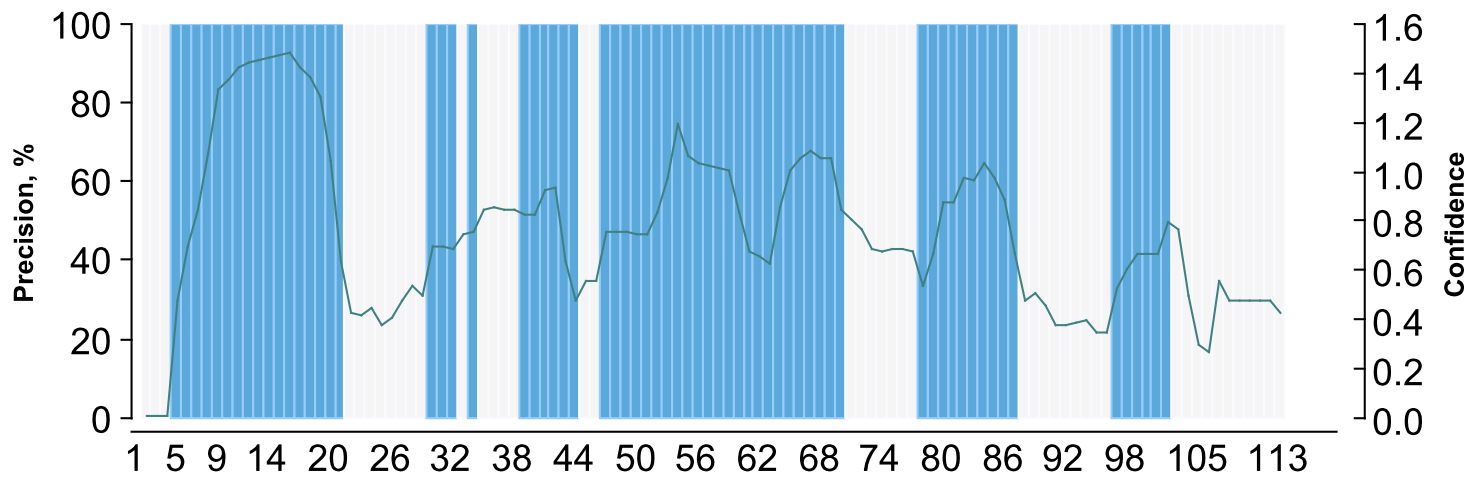

Supplement: Benchmark S1 — Local centroid precision for each target in the benchmark set and a breakdown of the torsion angle prediction performance by residue type and secondary structure. (ZIP) [file pone.0076512.s001.zip › Filtering/3npdA.pdf]

2106A

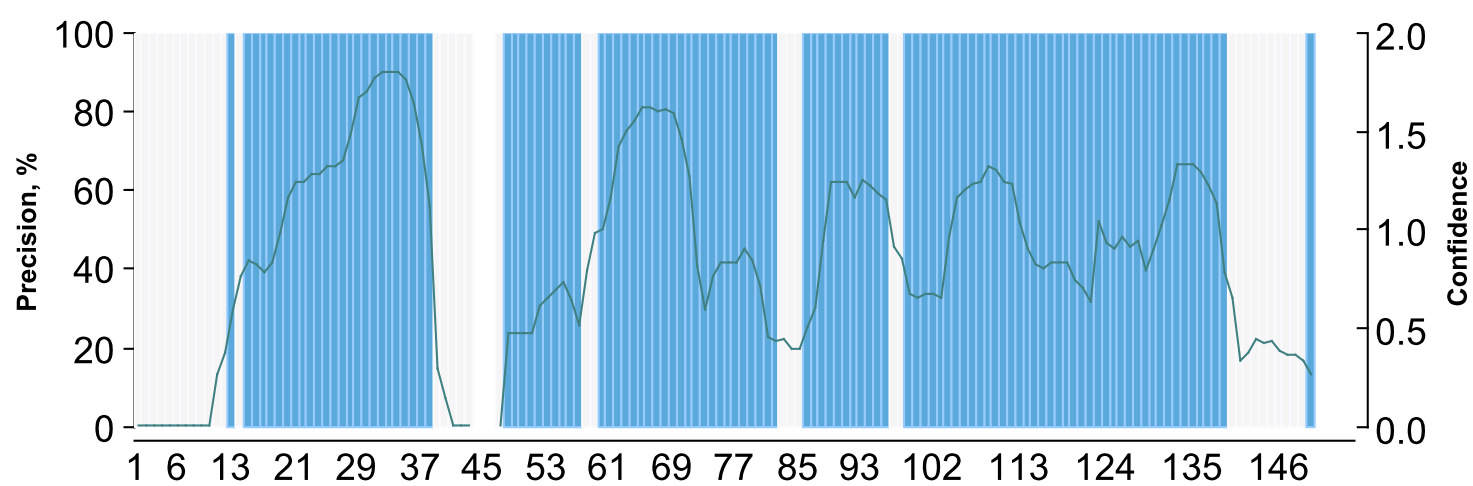

Supplement: Benchmark S1 — Local centroid precision for each target in the benchmark set and a breakdown of the torsion angle prediction performance by residue type and secondary structure. (ZIP) [file pone.0076512.s001.zip › Filtering/2l06A.pdf]

2ky4A

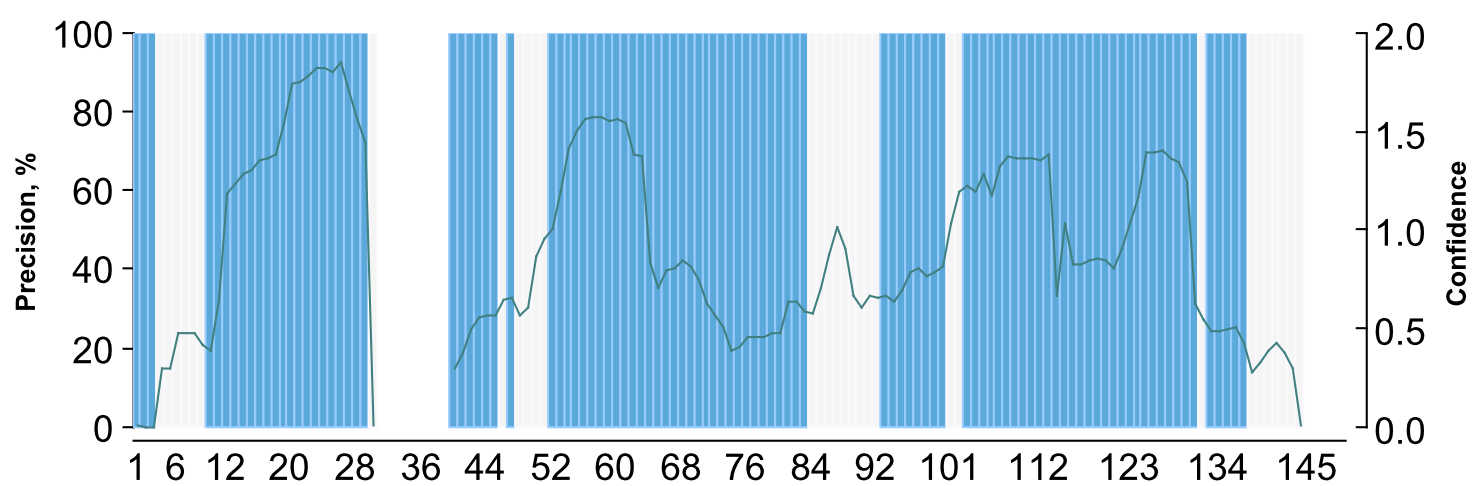

Supplement: Benchmark S1 — Local centroid precision for each target in the benchmark set and a breakdown of the torsion angle prediction performance by residue type and secondary structure. (ZIP) [file pone.0076512.s001.zip › Filtering/2ky4A.pdf]
